# Generalisation of automatic tumour segmentation in histopathological whole-slide images across multiple cancer types

**DOI:** 10.1038/s41698-026-01311-6

**Published:** 2026-02-04

**Authors:** Ole-Johan Skrede, Manohar Pradhan, Maria Xepapadakis Isaksen, Tarjei Sveinsgjerd Hveem, Ljiljana Vlatkovic, Arild Nesbakken, Kristina Lindemann, Gunnar B. Kristensen, Jenneke Kasius, Alain G. Zeimet, Odd Terje Brustugun, Lill-Tove Rasmussen Busund, Elin H. Richardsen, Erik Skaaheim Haug, Bjørn Brennhovd, Emma Rewcastle, Melinda Lillesand, Vebjørn Kvikstad, Emiel Janssen, David J. Kerr, Knut Liestøl, Fritz Albregtsen, Andreas Kleppe

**Affiliations:** 1 Institute for Cancer Genetics and Informatics, Oslo University Hospital, Oslo, Norway; 2 Department of Molecular Oncology, Oslo University Hospital, Oslo, Norway; 3 Institute of Clinical Medicine, University of Oslo, Oslo, Norway; 4 Department Gastrointestinal and Paediatric Surgery, Oslo University Hospital, Oslo, Norway; 5 Department of Surgical Oncology, Oslo University Hospital, Oslo, Norway; 6 Department of Gynaecological Oncology, Oslo University Hospital, Oslo, Norway; 7 Department of Gynecological Oncology, Centre for Gynecological Oncology Amsterdam, Amsterdam University Medical Centres, Amsterdam, The Netherlands; 8 Department of Obstetrics and Gynaecology, Innsbruck Medical University, Innsbruck, Austria; 9 Section of Oncology, Drammen Hospital, Vestre Viken Health Trust, Drammen, Norway; 10 Department of Medical Biology, UiT The Arctic University of Norway, Tromsø, Norway; 11 Department of Clinical Pathology, University Hospital of North Norway, Tromsø, Norway; 12 Department of Urology, Vestfold Hospital Trust, Tønsberg, Norway; 13 Department of Urology, Oslo University Hospital, Oslo, Norway; 14 Department of Pathology, Stavanger University Hospital, Stavanger, Norway; 15 Department of Chemistry, Bioscience and Environmental Engineering, University of Stavanger, Stavanger, Norway; 16 Department of Forensic Medicine, Oslo University Hospital, Oslo, Norway; 17 Nuffield Division of Clinical Laboratory Sciences, University of Oxford, Oxford, UK; 18 Department of Informatics, University of Oslo, Oslo, Norway; 19 Centre for Research-based Innovation Visual Intelligence, UiT The Arctic University of Norway, Tromsø, Norway

**Keywords:** Cancer, Computational biology and bioinformatics, Oncology

## Abstract

Deep learning is expected to aid pathologists in tasks such as tumour segmentation. We developed a general tumour segmentation model for histopathological images and examined its performance in different cancer types. The model was developed using over 20,000 whole-slide images from over 4000 patients with colorectal, endometrial, lung, or prostate carcinoma. Performance was validated in pre-planned analyses on external cohorts with over 3000 patients across six cancer types. Exploratory analyses included over 1500 additional patients from The Cancer Genome Atlas. Average Dice coefficient was over 80% in all validation cohorts with en bloc resection specimens and in The Cancer Genome Atlas cohorts. No performance loss was observed when comparing the general model with single-cancer models specialised in cancer types from the development set. In conclusion, extensive and rigorous evaluations demonstrate that generic tumour segmentation by a single model is possible across cancer types, patient populations, sample preparations and slide scanners.

## Introduction

The rapidly increasing adoption of digital pathology enables workflows that contribute towards the realisation of precision medicine^1^. In particular, the introduction of methods based on modern artificial intelligence (AI) promises for improved selection among therapeutic options^2^. Provided sufficient representative data, these AI-based methods outperform earlier automatic procedures, and may help ease routines, improving the precision in diagnostic tasks, and allowing the pathologists to focus on especially challenging problems. Advances in technology also enable efficient collection of multiple samples from each patient. However, the subsequent analyses of these samples will further increase the pressure on pathological services already affected by increasing cancer incidences and a general shortage of pathologists^3,4^. Thus, automatic procedures also in the analytic steps of the diagnostic processes may be vital to bring diagnostic advances to practical use in the clinic. The development of bright-field slide scanners enabled the production of high-quality whole-slide images (WSI) of tumour slides. A first step in many analyses of such WSIs, especially automatic analyses, is the segmentation of the tumour areas from the background. The modern deep learning AI-techniques have demonstrated high efficiency in recognising patterns in images, thus making automatic tumour segmentation an attractive and realistic alternative to manual tumour segmentation^5,6^. Such an automatic procedure may also produce heat maps highlighting regions of particular interest, assisting pathologists in their assessment.

In this study, we aimed to develop a universal deep learning model for automatic tumour segmentation in whole-slide images of haematoxylin and eosin (H&E) stained tissue sections from formalin-fixed, paraffin-embedded (FFPE) tissue blocks. Most previously published models are developed and tested using data from a single cancer type (e.g. lung, prostate or breast cancer). Some relevant studies present results from multiple cancer types, but none of them present performance estimates in cancer types different from the one used to train the model^7–12^. Recently, we have seen *foundation models*^13^ published for computational pathology, which are trained by self-supervised learning on histological images from multiple cancer types^14–20^. These models are pan-cancer by design, and can be utilised for tumour segmentation if additional segmentation-specific components that also need to be trained are attached.

Although focusing on a single cancer type can ease utilisation of features characteristic to the specific cancer type, this also limits the applicability of the resulting model. A pan-cancer model may tend to focus on more general characteristics in cancer tissues, can be trained and validated on larger data volumes, and can be applied to multiple cancer types, including rare cancers with insufficient amounts of available data to train a specialised model. A universal tumour segmentation model trained on much and varied data may also be expected to be more robust and generalise better than specialised models. There is, however, also a need to study the limits of these models in terms of elements such as the technical quality of the input, different tissue types, and sociodemographic variations.

To examine pan-cancer segmentation in WSIs of H&E-stained tissue sections, we here present the development and validation of a single segmentation model developed using cohorts from colorectal, endometrial, lung, and prostate carcinoma. This model is referred to as the *primary model* to distinguish it from other models presented in this study: two models obtained by replicating the training of the primary model, and four cancer-type specialised models only trained on a single cancer type.

**Fig. 1:**
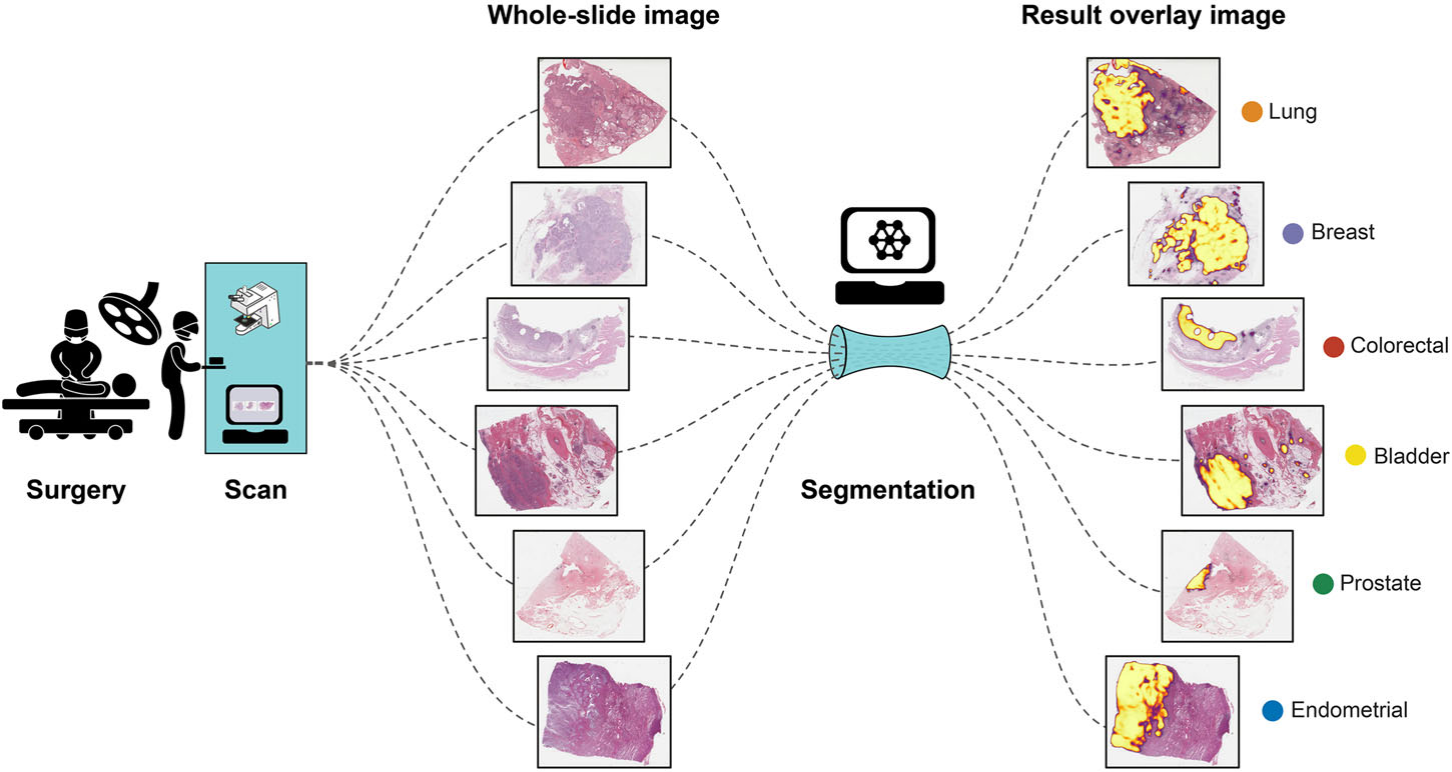
Overview of input and corresponding result. WSIs of H&E stained tissue from different cancer types are all segmented by the same deep learning-based segmentation method. This figure shows input images on the left and result heatmaps on the right overlain the corresponding input images. Heatmaps show the output of the segmentation network as a score image coloured as in Fig. 4b from transparent (value 0) to yellow (value 100%).

The performance of the primary model is validated in a pre-specified primary analysis using independent cohorts from the same four cancer types as well as from breast and bladder carcinoma (Fig. 1). Combined, the included cancer types represent about 40–45% of both new cancer cases and cancer deaths worldwide in 2020^21^. Pre-specified secondary analyses compare the primary model with cancer type specialised models and evaluate its robustness on images from different slide scanners. To illustrate the level of uncertainty in the segmentation task, we also report the intra- and inter-observer variability of two pathologists on a breast cancer cohort. Further exploratory analyses include performance evaluation in four different cohorts from The Cancer Genome Atlas (TCGA) and examination of factors that may lead to suboptimal segmentation results.

## Results

### Materials

**Fig. 2:**
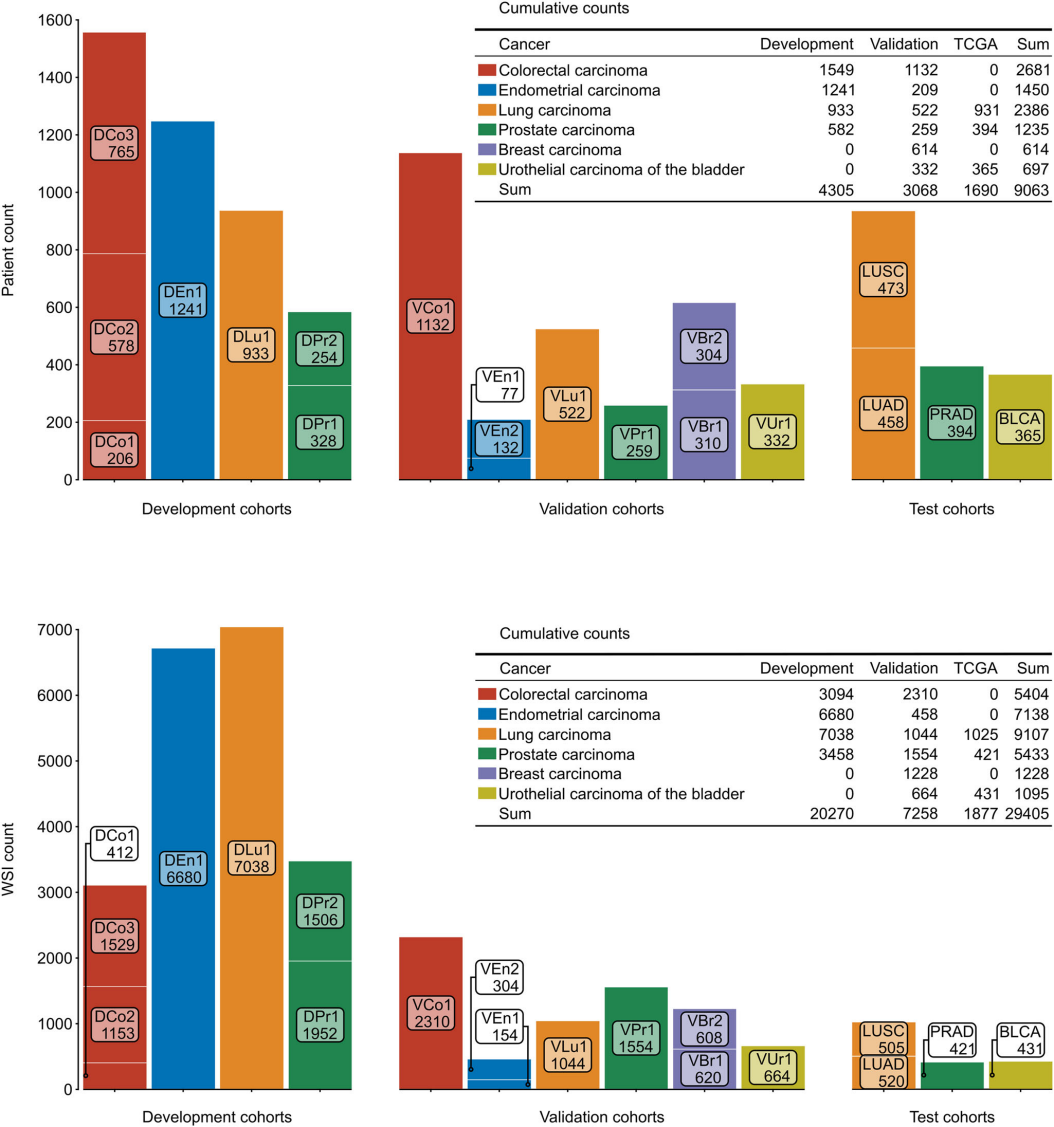
Included patient and WSI count. The charts show counts stratified by patient cohort and grouped by cancer type and cohort use (for method development, validation or test), for patients (top panel) and WSIs (bottom panel). The tables in the respective panels display cumulative counts aggregated by cancer type and cohort use.

To develop the primary segmentation model, we used 20,270 WSIs from 4305 patients encompassing four types of cancer obtained using two different microscope scanners. To mitigate the imbalance between the included cancer types in training, we oversampled the minority groups on a tissue section level, resulting in 3519 tissue sections sampled from each cancer type, or a total of 28,144 sampled WSIs when counting both scanners. The pre-planned primary analysis assessed the performance by comparing the automatic segmentation with a manual reference segmentation in 3629 WSIs from 3068 patients and six cancer types. Additional exploratory analyses included evaluating 1877 WSIs from 1690 patients and three cancer types from TCGA. See Fig. 2 for patient and WSI counts stratified by cohort and grouped by cancer type and use, and Methods for further details about the included patient cohorts.

### Primary analysis of model performance

**Fig. 3:**
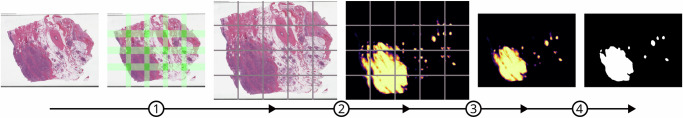
Segmentation method pipeline. Illustrated with example WSI TCGA-FD-A6TE-01Z-00-DX1 from BLCA, the same that is used in Fig. 4. 1: Downscale the input scan to spatial resolution 1 µm per pixel and partition it into tiles of size 7680 × 7680 pixels with minimum 1024 pixels overlap in each direction. In the second image from the left, green opacity signify overlap. 2: Process each tile with the segmentation network to produce score tiles. 3: Merge score tiles to score image with linear weight based on distance in overlapping regions. 4: Segment the score image into foreground and background regions.

**Fig. 4:**
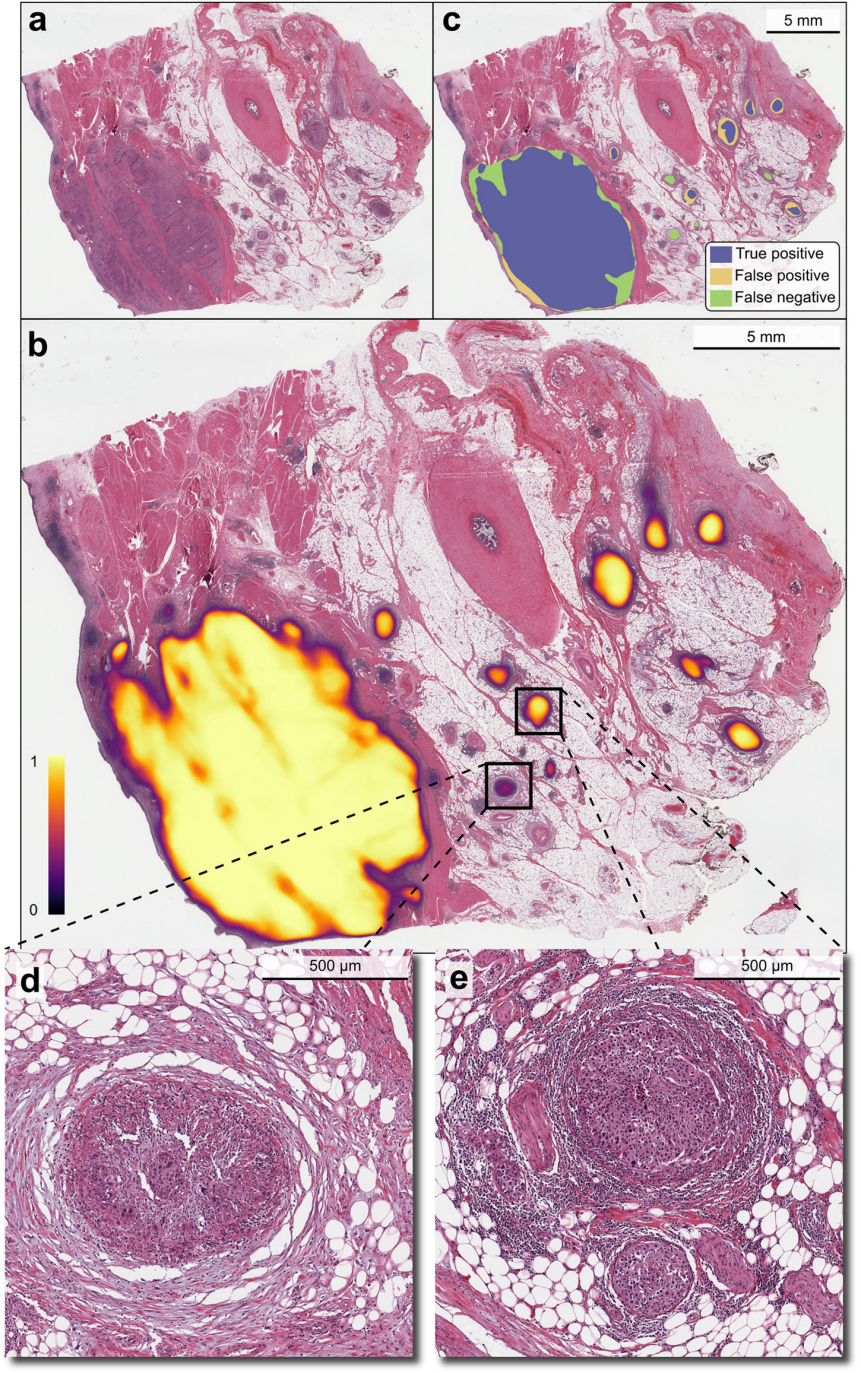
Example result in TCGA-FD-A6TE-01Z-00-DX1 from BLCA. Input WSI (**a**), annotated with the probability image (**b**) and with the segmentation result (**c**). The resulting DSC is 92.16% which is similar to the BLCA median DSC of 92.31%. With reference to **c**: the DSC is computed as two times the true positive area (blue) divided by the sum of the automatically segmented areas (blue and yellow) and the manually segmented areas (blue and green). The detailed crops show one false negative region (**d**) and one true positive region (**e**). We see that the false negative region (**d**) has a signal in the probability image (**b**), but that it is too weak to be included in the final segmentation (**c**). Comparing **(d)** with **(e)**, both show clusters of tumour cells and aggregates of lymphocytes surrounded by adipose tissue, but the area of the largest tumour cell cluster in **e** is about ten times larger than the area of the largest cluster in **d**, which might explain the weaker response.

Figure 3 illustrates how the method segments a WSI by creating an image indicating the probability of a pixel being part of a region displaying tumour, before the final dichotomous tumour segmentation is obtained through a thresholding procedure. The performance of the automatic segmentation is evaluated by comparing it with the manual segmentation using the Dice similarity coefficient (DSC), and the regions involved in this computation are illustrated by example in Fig. 4c.

**Fig. 5:**
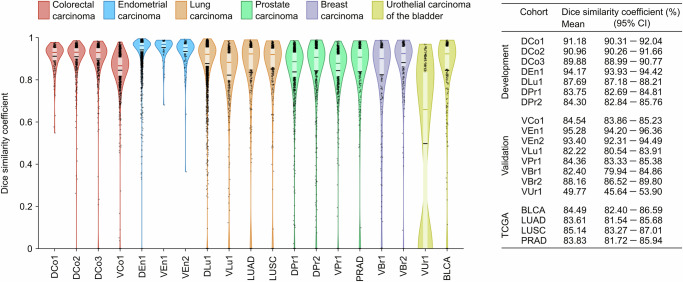
Primary model results in cohorts from development, validation and TCGA. Only scans from Aperio AT2 are included from the development and validation cohorts. For each cohort, the chart in the left panel shows the DSC for individual scans (black dots) and the approximate DSC distribution as a violin plot. It also summarises the DSC with interquartile range (light box), mean value (black horizontal line), and median value (coloured horizontal line). The table in the right panel shows the mean DSC per cohort and the corresponding 95% confidence interval (CI).

The primary segmentation model achieved a mean DSC of 82% to 94% in the validation cohorts with en bloc specimens of solid tumours imaged with the Aperio AT2 scanner (Fig. 5). This includes two cohorts from breast carcinoma, a cancer type not present in the development materials. Especially good performance was observed for endometrial carcinoma (cohorts VEn1 and VEn2) with DSC well over 90%. The exception from these satisfactory results was for the VUr1 cohort with transurethral resection (TUR) specimens from early-stage urothelial carcinoma of the bladder.

Additional performance evaluation metrics were also computed (see Supplementary Table S1 and Supplementary Fig. S1). Except for the TUR-sample bladder cohort, the proportion of tissue marked as tumour was similar when segmented automatically and manually, with only a slight tendency for the automatic procedure to mark more as tumour in colorectal (VCo1) and lung cancer (VLu1). For VLu1, this tendency is also reflected in a high true positive rate (sensitivity) of 91% compared with a slightly lower true negative rate (specificity) of 88%, indicating some over-segmentation in this cohort. A difference between sensitivity and specificity is also seen in the cohorts from endometrial cancer, where (sensitivity, specificity) are (96%, 96%) and (92%, 93%) for VEn1 and VEn2, respectively. Conversely, the automatic segmentation displays higher specificity than sensitivity in the prostate and breast cancer cohorts, with differences ranging from 0.08 to 0.15.

The area of every segmented region in the validation cohorts was measured, and the distributions are presented in Supplementary Fig. S18. The manually annotated tumour region size distribution is similar in all validation cohorts, except for the lung cohort (VLu1) where there are more small regions (area less than 1 mm^2^) per WSI, and in the bladder cohort (VUr1) where there are even more small regions per WSI. The size distributions for the regions automatically segmented by the primary model are similar in all validation cohorts, with a tendency towards more small regions in the prostate, breast and bladder cohorts. This results in many small false negative regions in VLu1 and VUr1, and some more small false positive regions in the prostate and breast cohorts compared with the other validation cohorts. The average DSC is high in all validation cohorts when only considering true positive regions, but the fraction of images containing true positive regions is considerably lower in the bladder cancer cohort than in the other validation cohorts (Supplementary Table S9).

### Factors affecting segmentation performance

The DSC of the primary model correlated positively with both the manually segmented tumour area and prevalence of tumour in all validation cohorts with Spearman’s rank correlation coefficient *ρ* > 0.36 and *p* < 0.0001, except for in endometrial carcinoma, where only VEn2 was significantly correlated with area (*ρ* = 0.29, *p* = 0.0004) and prevalence (*ρ* = 0.38, *p* < 0.0001). DSC also showed some correlation with known risk predictors, e.g. with pathological T stage (pT) in VLu1 (*ρ* = 0.10, *p* = 0.017), VPr1 (*ρ* = 0.11, *p* = 0.021) and VBr1 (*ρ* = 0.28, *p* < 0.0001), with Nottingham prognostic index in the breast cancer cohorts VBr1 (*ρ* = 0.18, *p* = 0.0017) and VBr2 (*ρ* = 0.25, *p* < 0.0001), and with Gleason score in VPr1 (*ρ* = 0.12, *p* = 0.0010). See Supplementary Section 2.1 for these and additional correlations between the DSC of the primary model and selected variables in the validation cohorts.

### Comparison with models specialised on single cancer types

**Fig. 6:**
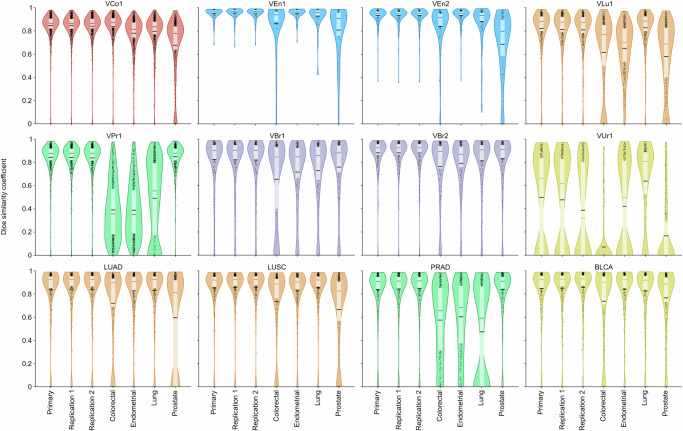
Performance comparison of all presented models. Results from test and validation cohorts with the Aperio AT2 scanner. See Fig. 5 for the display legend.

Four cancer type specialised models were developed on subsets of the full training data; the specialised colorectal model was trained using only the cohorts from colorectal carcinoma (DCo1, DCo2 and DCo3), and vice versa for the specialised endometrial, lung, and prostate models. The four cancer type specialised models achieved similar results as the primary model when tested on validation cohorts from the same cancer types they were trained on (see performance overview in Fig. 6 with details in Supplementary Section 1.3), with a mean difference in DSC below 0.007 between the primary model and the specialised model for all specialised models (Supplementary Fig. S17). Thus, the larger and more varied training data of the pan-cancer primary model seems to have compensated for the specific features that the specialised models may utilise. In general, the cancer type specialised models failed to generalise beyond their respective cancer types, with exceptions including the lung model that performed well in cohorts from endometrial carcinoma.

### Robustness to variations in sample origin, preparation and imaging

**Fig. 7:**
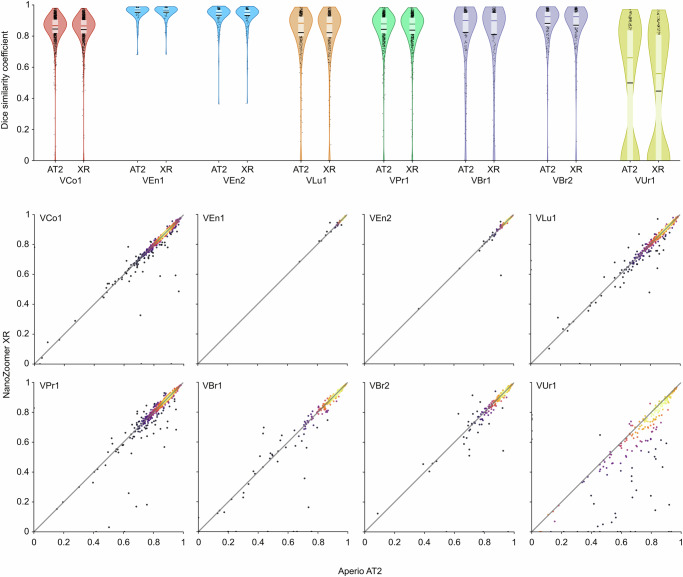
Performance comparison between Aperio AT2 and NanoZoomer XR. The results show the DSC of the primary model evaluated on all validation cohorts. In the top panel, results are summarised in violin plots (see Fig. 5 for display legend), while the bottom panel shows scatter plots where the diagonal line traces equal scores in scans from Aperio AT2 and NanoZoomer XR. Markers in the scatter plots are coloured by estimated density using the same colourmap as in Fig. 4b, using Gaussian kernel density estimation from skipy.stats.gaussian_kde in Python.

**Fig. 8:**
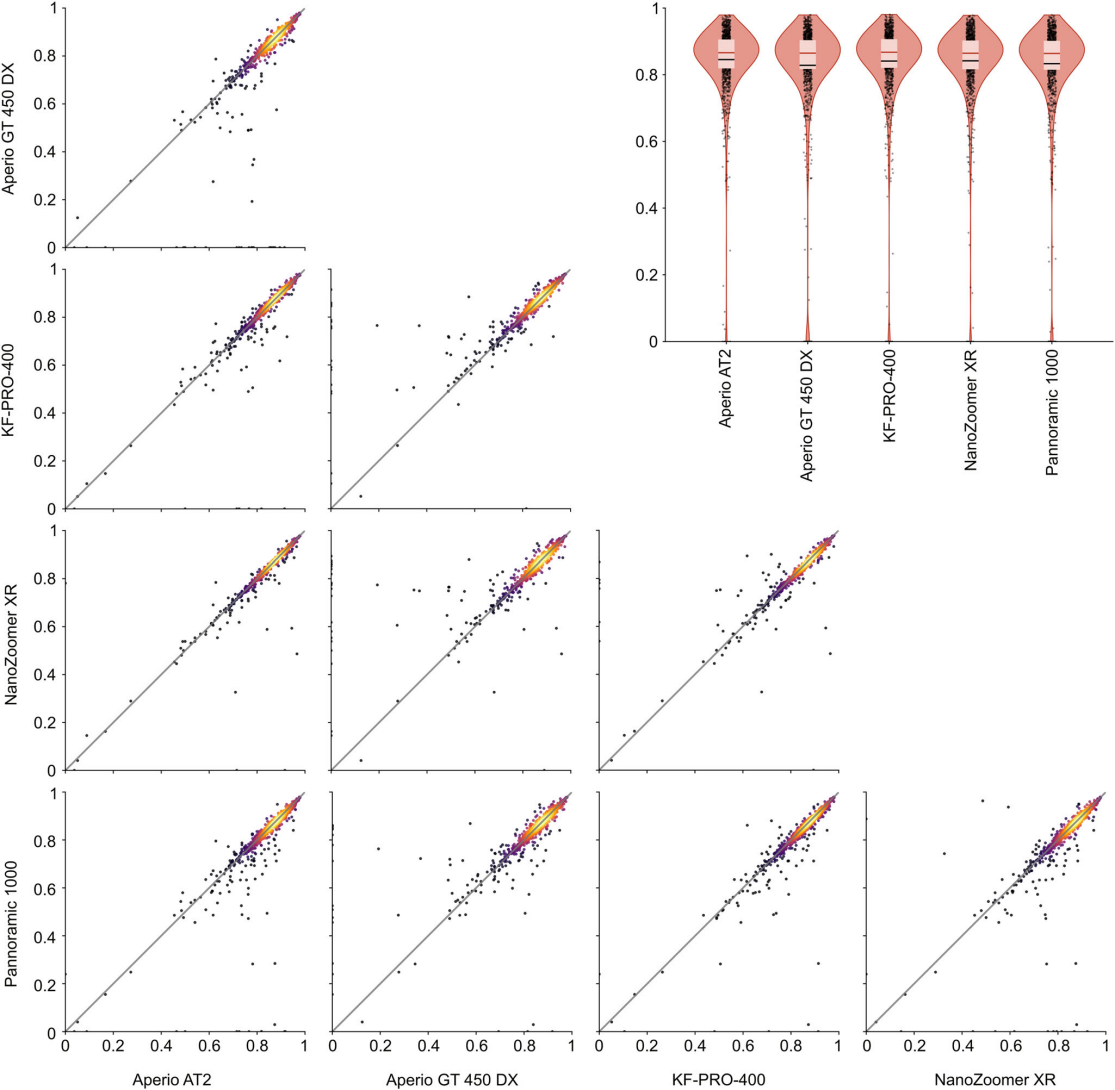
Performance comparison on slides scanned with five different scanners. DSC of the primary model evaluated on the VCo1 validation cohort with five different scanners. See Fig. 5 for violin plot legend and Fig. 7 for scatter plot legend.

The performance of the primary model in images from the Aperio AT2 scanner was preserved in the images from the NanoZoomer XR scanner (Fig. 7). Scan-by-scan comparisons reveal no particular shift between scans from Aperio AT2 and NanoZoomer XR, with a mean difference DSC below 0.006 in cohorts from colorectal, endometrial, and lung cancer, and a mean difference DSC below 0.014 in cohorts from breast cancer (Supplementary Fig. S17). Moreover, from Fig. 8, we see that the evaluation of the primary model in VCo1 scanned with five different scanners shows no substantial performance difference between the scanner models; the mean DSC ranging from 82.9% in Aperio GT 450 DX to 84.6% in Aperio AT2 (more details in Supplementary Section 2.6). Robustness to both external laboratory sample preparation and imaging was demonstrated by achieving a mean DSC over 83% in all included TCGA cohorts: BLCA, LUAD, LUSC and PRAD (Fig. 5).

When comparing the performance in the validation cohorts with the cohorts used to develop the primary model, we see from Fig. 5 that the performance is better in the development cohorts from colorectal (91.18%, 90.96% and 89.88% vs. 84.54%) and lung cancer (87.69% vs. 82.22%), while it is more similar in the cohorts from endometrial (94.17% vs. 95.28% and 93.40%) and prostate cancer (83.75% and 84.30% vs. 84.36%).

Finally, the performance of the primary model in all validation and test cohorts was similar in the two models trained with an identical setup as the primary model, only differing by using different random seeds, which affects model weight initialisation and input order (see result overview in Fig. 6 with details in Supplementary Section 1.4). Per-scan comparisons show an absolute mean difference in DSC between the primary and the replication model’s results below 0.005 in all non-TUR validation cohorts except for VLu1, where the difference is 0.009 and 0.013 for the first and second replication, respectively (Supplementary Fig. S17).

### Intra- and inter-pathologist variability

All 304 Aperio AT2 scans in the breast carcinoma validation cohort VBr2 were annotated for tumour a second time by pathologists MP and LV, about two years after this cohort had been annotated the first time by MP. The mean DSC between the first and second segmentations of MP was 91%, while the mean DSC between LV and the second segmentation of MP was 77%. For comparison, the mean DSC between the primary automatic segmentation and both the first and the second segmentations by MP was 88% (Supplementary Fig. S19). MP and LV had segmented overlapping regions in all 304 scans in VBr2, while the automatic primary model did not segment any regions in four scans (1.32%).

### Failure to segment fragmented samples from early-stage tumours in the bladder

The primary model did not segment any regions in 108 (33%) of the 332 Aperio AT2 scans in the bladder validation cohort (VUr1), even though all of them show tumour tissue. When only considering the 217 (65%) scans with true positive segmented regions, the DSC is 87% (Supplementary Table S9). A likely reason for this failure is that the tumour samples obtained through TUR are generally small and from early-stage tumours, which results in low-valued regions in the probability images from the segmentation network that are ultimately erased by the post-processing.

DSC for the primary model is generally correlated with tumour size, and for VUr1 we observe a Spearman’s rank correlation *ρ* = 0.65, *p* < 0.0001 (Supplementary Fig. S16).

In the 342 (79%) scans without fragmented tissue in the TCGA bladder cohort BLCA, the primary model failed to detect any cancerous regions in only 8 scans (2%), with a mean DSC of 91% in the 334 (98%) scans with predictions. The performance degraded when considering the 87 (20%) scans with fragmented tissue: 12 (14%) scans had no predicted cancerous regions, and the mean DSC was 77% in the 75 (86%) scans with predictions.

In VUr1, 255 (77%) scans are from pTa, 1 (less than 1%) scan is from pTis, and 76 (23%) are from pT1 (Protocol Table 8, Supplementary Section 6). In the pTa or pTis group, no regions were segmented in 38% of the scans, but this proportion decreased to 13% in pT1. For the BLCA cohort with more advanced stage cancers (pT2: *n* = 112 (26%), pT3: *n* = 203 (47%), pT4: *n* = 58 (13%)), results were markedly better with a mean DSC of 84% in the whole cohort (Supplementary Table S10).

When lowering the hysteresis threshold values in the segmentation post-processing, we obtain higher performance in VUr1 (Supplementary Fig. S23). The highest mean DSC of 89% was observed when using thresholds of 5% and 10% (Supplementary Table S14).

### Performance comparison with MedSAM

To provide context for our results, we evaluated the performance of MedSAM in all validation datasets^22^. MedSAM is presented as a foundation model for medical image segmentation, developed by finetuning the segment anything model (SAM) on a large dataset of medical images^23^. MedSAM requires prompting, that is, some marker in the image to indicate where the regions to segment are located. For this reason, we evaluated two versions of MedSAM, one where the prompt was the bounding box of the tissue foreground region, and another where we used the bounding box of the manually segmented tumour areas as prompts.

MedSAM prompted by a bounding box of the manually segmented tumour achieved a mean DSC of 79%, 89%, 87%, 72%, 66%, 81%, 83% and 75% when applied on the Aperio AT2 scans from validation cohorts VCo1, VEn1, VEn2, VLu1, VPr1, VBr1, VBr2 and VUr1, respectively (Supplementary Fig. S22). When prompted by a bounding box of the whole tissue foreground, MedSAM achieved a mean DSC of 48%, 63%, 53%, 47%, 28%, 34%, 42% and 64% in the same datasets. The bounding boxes of the manually segmented tumour without refined segmentation by MedSAM achieved a mean DSC of 74%, 82%, 78%, 67%, 60%, 70%, 73% and 74%.

## Discussion

A deep learning model developed to automatically delineate cancerous regions in WSIs of conventionally H&E-stained tissue sections demonstrated good overall performance in external validation cohorts from different cancer types, including breast cancer, not represented in the development set. Comparing the primary pan-cancer model to specialised models developed and tested on cohorts of one cancer type indicated no loss of performance, neither overall nor scan-by-scan. This might be because the specialised models have been trained on a subset of the general primary model’s training data, and the network has sufficient capacity to make efficient use of the more comprehensive data. It should also be noted that this experiment only compared the primary model with models specialised in cancer types present in the training set of the primary model. However, the good performance of the primary model also extends to cancer types not present in the training set (an ability we did not generally observe in the specialised models), indicating that the primary pan-cancer model can also utilise more general features to distinguish between cancerous and non-cancerous tissue.

Our primary performance evaluation metric, DSC, is a purely overlap-based metric that is independent of true negative counts, and also invariant to a reference-prediction swap. To further examine nuances in the behaviour of the primary segmentation model, we included additional analyses and performance metrics. All additional statistics included in the first secondary analysis are derived from the pixel overlap contingency table. This provides insight into the kind of overlap (e.g. over-segmentation or under-segmentation), but does not distinguish between disconnected regions in an image, nor does it consider the shape of the regions. Shape similarity between reference and predicted regions is not explicitly evaluated in this study, but we designed the method to produce visually similar results to the reference segmentation.

Another perspective of the segmentation performance is provided by the analysis of individual segmented regions (connected pixels annotated as tumour). This reveals that the size distributions of manual segmentations are different between the cancer types, which, to a lesser extent, is also observed in the automatic segmentations. In particular, the behaviour of the automatic segmentation in cohorts from prostate and breast carcinoma is similar, which might be explained by the similarities of the two diseases^24^.

From Fig. 5, we see that the mean DSC is determined by a majority of scans with high DSC and a small minority with very low DSC, and that the median DSC is substantially higher than the mean. Related to this is the high DSC when only considering true positive regions (Supplementary Table S9). This can suggest that the dominant failure type is a few completely failed segmentations, rather than many partly failed segmentations.

The primary model’s performance is highly correlated with the annotated tumour area size in most cohorts. Moreover, results with DSC less than 50% (and in particular 0%) mostly appear in scans with an aggregated tumour area less than (10 mm)^2^. Inspection of the prediction probability images shows that these regions often have a positive signal which is discarded in the final dichotomisation into tumour and background (see Fig. 4d). In general, if a more sensitive model is desired, one can lower the post-processing thresholds without requiring retraining of the underlying neural network. From the experiment where we varied the hysteresis threshold values (Supplementary Section 2.8), we see that this approach would have helped in the challenging VUr1 dataset.

Image preparation is a source of variation that might cause worse performance in settings external to the development settings. The primary segmentation model is developed and externally validated on scans from samples originating from many institutions in many different countries and has been shown to perform consistently across the differences in sample preparation and patient population. Since both development and validation cohorts in lung and prostate are from Norwegian hospitals, we evaluated the primary model on lung and prostate cohorts from TCGA (LUAD, LUSC and PRAD). The performance was maintained, increasing our confidence that the primary model generalises well. The primary model behaved similarly in WSIs acquired from the two different scanners used to train the model, both when considering individual slides and performance averaged over cohorts. This result is corroborated by the similar performance of the model when evaluated on the VCo1 cohort scanned with five different scanners. This suggests that the performance of the primary model also generalises to scanners not present in the training data.

We evaluated the primary model on all scans from the development cohorts to check if any substantial over-fitting had occurred. Even though the performance is good in these cohorts, they are not out of line compared with the results in the validation cohorts, suggesting that over-fitting is limited.

For simplicity, we did not employ any hyperparameter tuning or model selection, nor did we combine models to form an ensemble model. This can come with a cost of repeatability, but when comparing the primary model with the replication models, both overall performance and scan-by-scan comparisons show that the performance of models resulting from our method is stable.

In the experiment where VBr2 was manually annotated a second time, the intra-observer similarity was greater than the inter-observer variability, which we find reasonable. What is perhaps more surprising is that the agreement (in terms of average DSC) was higher between the automatic segmentation and pathologist MP, than between pathologists LV and MP, which might indicate that the automatic segmentation model is somewhat adapted to MP’s segmentation ‘style’ in addition to high agreement on what regions to annotate as tumour.

MedSAM, prompted by tissue bounding boxes, performs substantially worse than the tumour prompted version, which again performs substantially worse than our primary model on VLu1 and VPr1. The tissue-prompted version is an example of a truly automatic method, while the tumour-prompted version could represent a scenario where a human expert uses MedSAM to segment areas of interest. In the other validation cohorts, its performance is lower but comparable to our primary model, except for in VUr1, where its performance is substantially better. However, the performance of the tumour prompted by MedSAM in VUr1 can largely be explained by the prompting bounding boxes, which, without MedSAM, achieves almost the same performance.

The poor performance in the validation set from urothelial carcinoma (VUr1) was probably related to its origin from the TUR procedure, often resulting in scans of small, fragmented samples. Regions where manual and automatic segmentations overlap are often correctly segmented, and the poor general performance is dominated by regions completely missed by the automatic segmentation. Note that the underlying probability image often contains the correct signal, but that this is too low to survive the thresholding and pruning in the post-processing. Challenges with fragmented tumours and small fragments were also seen for the TCGA urothelial carcinoma cohort BLCA. The observed correlation between the size of the tumour region and DSC confirms the problems with detecting small areas of the tumour, which partly explains the poor performance in VUr1, since it contains many small annotated tumour regions compared with the other cohorts. Additionally, VUr1 contained many scans from pTa or pTis cancers where the primary model failed to detect any tumour. This improved in the pT1 cancers, in line with the observation in other cancer types, indicating more challenges with segmenting early-stage cancers. It is, therefore, reasonable to conclude that the primary model is not inferior in samples from bladder carcinoma as such, but that the poor performance in VUr1 is rather explained by its fragmented tissue samples and the high proportion of early-stage cancer, which results in weak probability regions by the segmentation network that are zeroed in the post-processing.

This study relates explicitly to the segmentation of images into regions with and without predicted tumours. This dichotomisation is useful for evaluating the model’s performance, and the resulting masks can readily be used in subsequent analyses. However, as a visual aid for pathologists in the clinic, the non-dichotomised probability image displayed as a heat map might be more useful (see Fig. 4b).

A possible limitation of this study is that all scans included were manually annotated by the same pathologist (MP), potentially biasing the reported performance compared to the performance in cohorts annotated by other pathologists. All scans in the development and validation materials were scanned at the same laboratory at the Institute for Cancer Genetics and Informatics in Oslo, Norway, which might also impose a systematic bias that could cause results to be overoptimistic. However, the results in TCGA cohorts scanned elsewhere suggest that this is not a substantial issue.

It should be noted that the model has been developed and mainly validated in materials from resections, and that we can not anticipate how it will behave in biopsy samples. The performance in the validation cohort with TUR samples, and the suggested causes of this problem, might indicate that the method with the presented settings is not suited for biopsies.

The method was designed for tumour segmentation and mainly validated in samples containing cancers, and we can not recommend its use in settings where detecting small early-stage cancers or precancerous conditions is very important. Limited exploratory analyses suggest that we can increase the sensitivity of the method simply by relaxing the post-processing, with the likely side effect of increasing false positive detections. We decided to present the results of a fully automatic method with pre-determined segmentation thresholds, and extensive analyses of different post-processing methods were not in the scope of this study.

All samples are from carcinomas, and we have not evaluated the performance in other histological super-categories such as sarcoma. Exploring this is required before this primary model is applied to cancers other than carcinomas.

This study lacks comprehensive comparisons with other segmentation models, which would have contextualised the presented results and could have provided further insight into their relative performance. It was challenging to find published, fully automatic tumour segmentation models that we could apply directly without additional training. We did include comparisons with MedSAM, but this is not ideal since MedSAM is interactive, and is not developed primarily for tumour segmentation in histological images. A more relevant approach to compare against would be a computational pathology foundation model (such as those presented in refs. ^14–20^) adapted for segmentation using methods such as ViT-Adaptor and Mask2Former, but this would have required additional training^25,26^. We attempted another option, namely PathoSAM, which is similar to MedSAM except it is specialised to histopathology^27^. Unfortunately, PathoSAM is finetuned to nucleus segmentation, and the results it produced were not suited as a comparison with our tumour segmentation model.

With the advent of publicly available foundation models for computational pathology which are pan-cancer in nature, it would be natural to adapt them to tumour segmentation and evaluate their performance on the materials included in this study. Another useful benchmark in this regard would have been to train well-established segmentation networks, such as U-net or the more recent nnU-net^28,29^. However, since this study was not primarily about the segmentation network, it was considered out of scope to train and compare multiple network architectures.

We emphasise our use of pre-planned validation in external cohorts and our extensive performance evaluation. All planned analyses together with information required to define these analyses were specified in a study protocol that adheres to the PIECES (*Protocol Items for External Cohort Evaluation of a deep learning System*) recommendations, and this protocol was fixed prior to validation (Supplementary Section 6)^30^. That no adjustments were made to the primary model after validation, and that the validation was pre-specified and performed only once, means that we can trust that the primary analysis gives an unbiased and realistic assessment of the model’s performance that is not overly optimistic and actually reflects how the model will perform in real usage on new data^30,31^.

We conclude that it was possible to develop an automatic segmentation model that performs well in multiple cancer types, without sacrificing performance compared with specialised models only trained on single cancer types from the development set. Small, fragmented tumours are a challenge, but otherwise the primary model was observed to perform well on tumour types not present in the development cohorts, on different scanners, on slides prepared at different laboratories and in patients from different countries. Thus, we conclude that such pan-cancer segmentation models can serve as a first step for subsequent automatic analyses of tumour areas and be implemented in digital pathology platforms for a more streamlined and effective diagnostic pipeline.

## Methods

A study protocol (Supplementary Section 6) was written following our previously published PIECES recommendations and fixed prior to all investigations that could reveal associations between the predicted and target segmentation masks in the validation cohorts^30^. It includes a description of included materials (Protocol Section 1), a detailed technical account of the method (Protocol Section 2), and the set of analyses we commit to perform and report the results of (Protocol Section 3).

The study protocol is appended unedited in Supplementary Section 6, with amendments in Supplementary Section 5. This Methods section contains a brief overview over the included materials, a complete method description, a brief overview over pre-planned analyses, and a complete description of exploratory analyses.

### Materials

In the following, a brief description of all included cohorts is presented. A detailed description of the development and validation cohorts, including acquisition flow diagrams and baseline characteristics is available in Protocol Section 1 (Supplementary Section 6). Development cohorts are used to develop the method, and the validation cohorts are used for validation according to our predefined protocol. In addition, we have included TCGA cohorts for some post-hoc analyses that are not stated in the protocol.

A simple naming scheme is used for the development and validation cohorts. The first letter is either *D* or *V*, signifying whether the cohort was used for development or validation, respectively. Then, two letters identify the type of cancer: *Co* for colorectal carcinoma, *En* for endometrial carcinoma, *Lu* for lung carcinoma, *Pr* for prostate carcinoma, *Br* for breast carcinoma, and *Ur* for urothelial carcinoma of the bladder. A final integer distinguishes cohorts of the same kind. The TCGA cohorts retain their original abbreviations.

The development and validation cohorts were chiefly acquired for other historic or present projects at our institute, and not this segmentation study. Whether a cohort was included in this study depended on whether we had available WSIs for that cohort with corresponding appropriate manual tumour segmentations. For TCGA, we included LUAD, LUSC, and PRAD to supplement the lung and prostate validation cohorts which were from Norwegian hospitals, and BLCA was included to further examine the performance in urothelial carcinoma of the bladder. A cohort is determined by organ and the acquisition site, and in the case for VBr1 and VBr2, also by date of surgery. Each cohort were used exclusively for either development or validation, generally depending on how we had used, or planned to use, this cohort in other studies at our institute.

The included cohorts were approved for use in this study by the Regional Committees for Medical and Health Research Ethics in Norway. DCo1, DCo2, DCo3, and VCo1 are covered by number 747764. DEn1, VEn1, and VEn2 are covered by number 24969. DLu1 and VLu1 are covered by number 880608. DPr1, DPr2 and VPr1 are covered by number 489722. VBr1 and VBr2 are covered by number 17865. VUr1 is covered by number 172005.

### Development cohorts

We used seven cohorts from four cancer types for method development. DCo1 is based on a consecutive series of patients with colonic adenocarcinoma treated between 1988 and 2000 at Akershus University Hospital, Norway^32^. DCo2 is based on a consecutive series of patients with stage I to III colorectal carcinoma treated between 1993 and 2003 at Aker University Hospital (now part of Oslo University Hospital (OUH)), Norway^33^. DCo3 originates from the VICTOR trial (ISRCTN registry, ISRCTN98278138) which recruited patients with stage II and III colorectal cancer from 151 hospitals in the UK between 2002 and 2004^34^. DEn1 comprises patients referred to the Department of Gynaecological Oncology at OUH, Norway, and diagnosed or operated for endometrial carcinoma between 2006 and 2017. DLu1 consists of patients resected for primary lung cancer as part of primary treatment between 2006 and 2018 at OUH, Norway^35^. DPr1 comprises patients who underwent radical prostatectomy (RP) between 1999 and 2010 at Vestfold Hospital Trust, Norway. DPr2 consists of patients who underwent RP between 1987 and 2005 at the Norwegian Radium Hospital (now part of OUH), Norway^36^.

### Validation cohorts

We used eight cohorts from six cancer types for the pre-planned method validation. VCo1 comprises patients with stage II and III colorectal carcinoma enroled between 2005 and 2010 from 170 hospitals in seven countries for the QUASAR 2 trial (ISRCTN registry, ISRCTN45133151)^37^. VEn1 consists of patients with endometrial carcinoma collected between 2001 and 2016 at Amsterdam Medical Center, The Netherlands. VEn2 comprises patients with endometrial carcinoma collected between 1999 and 2018 at the Department of Obstetrics and Gynaecology, Innsbruck Medical University, Austria. VLu1 includes a consecutive series of patients with stage I to III non-small cell lung carcinoma operated between 1990 and 2010 at the University Hospital of North Norway and Nordland Hospital Trust, Norway^38^. VPr1 consists of patients who underwent RP between 2001 and 2006 at the Norwegian Radium Hospital, Norway^39^. Note that although DPr2 and VPr1 both originates from the Norwegian Radium Hospital and have some overlap in time, they comprise a disjoint set of patients with different responsible surgeons. VBr1 are patients registered with lymph node negative breast cancer between 1990 and 1998 at Stavanger University Hospital, Norway, while VBr2 are patients from the same hospital registered with breast cancer between 2000 and 2004^40,41^. VUr1 comprises patients diagnosed with early-stage non-muscle invasive urothelial carcinoma of the bladder and without upper urinary tract urothelial carcinoma between 2002 and 2010 at Stavanger University Hospital, Norway^42^. All samples in VUr1 are from TURs, which result in glass slides typically containing fragmented tissue sections rather than a larger single tissue section, typical of the other development and validation cohorts.

### Test cohorts

We used four cohorts from three cancer types from TCGA for additional exploratory analyses: from lung (LUAD and LUSC), prostate (PRAD) and bladder carcinoma (BLCA)^43–46^. See Supplementary Section 4 for acquisition flow diagrams and baseline characteristics.

### Sample acquisition and preparation

A 3 µm section is cut from a FFPE tumour tissue block, mounted on a glass slide and stained with H&E before imaging with a microscope scanner to form a WSI. For some cohorts (DCo1, DCo2, DEn1, DLu1, DPr1, DPr2, VCo1, VEn1, VEn2 and VPr1), we received FFPE blocks and prepared tissue slides locally. For the rest of the cohorts (DCo3, VLu1, VBr1, VBr2 and VUr1), we received glass slides with H&E-stained tissue. All cohorts, except those from TCGA, were scanned locally using the highest available magnification (×40) in two scanners, an Aperio AT2 (Leica Biosystems, Germany) and a NanoZoomer XR (Hamamatsu Photonics, Japan), resulting in WSIs with a size on the order of 100,000 × 100,000 pixels with a spatial resolution of about 0.24 µm per pixel. WSIs from TCGA were downloaded from the TCGA Research Network (https://www.cancer.gov/tcga). For TCGA, we don’t know how samples were prepared, nor which scanner models were used for the original imaging, and the highest available magnification varied between ×20 and ×40 (Supplementary Section 4.3). Clinical data are from the TCGA Pan-Cancer Clinical Data Resource, whose publication should be consulted when interpreting the included variables and their values^47^. Manual tumour annotations were created by a pathologist (MP) for all included WSIs.

### Programming environment

Most programmes used in this project are implemented in the Python programming language. For method validation, programmes were run in a Docker container based on the pytorch/pytorch:1.11.0-cuda11.3-cudnn8-runtime image. The network optimisation was run in a Docker container based on the image nvcr.io/nvidia/pytorch:22.02-py3. Additional python packages used are listed in Protocol Table 9 (Supplementary Section 6).

Segmentation network processing was done on graphical processing units (GPUs). We used an Nvidia DGX machine with 8 A100 40 GB SXM GPUs, driver version 470.57.02 and CUDA version 11.4 for the network optimisation. For the validation, we used computers with Nvidia Titan RTX 24 GB GPU cards with driver version 465.19.01 and CUDA version 11.3.

### Method development

The segmentation method uses a convolutional neural network which needs to be optimised to this particular task of tumour segmentation. All necessary steps needed for preparation are described in this section and are summarised next:

1. Read input scans and downsample them to spatial resolution 1 µm per pixel (*Downsampling*)
2. Partition each scan into tiles with 2048 × 2048 pixels (*Tiling*)
3. Balance the development dataset (*Dataset balancing*)
4. Exclude background tiles (*Background tile exclusion*)
5. Augment the development dataset (*Dataset augmentations*)
6. Standardise input images (*Image value standardisation*)
7. Optimise the segmentation network (*Segmentation network*)

### Downsampling

Each scan used in this study is downsampled to a target spatial resolution of 1 µm per pixel (MPP). For reference, the highest magnification of many scanners is labelled ×40 magnification which corresponds to a spatial resolution of about 0.25 MPP depending on the scanner vendor and model. As an example, we have scans from Aperio AT2 with a ×40 magnification with spatial resolution 0.2530 MPP and scans from NanoZoomer XR with a ×40 magnification with spatial resolution 0.2267 MPP (rounded to four decimal places). Single nuclei are easily distinguished at this magnification, and even the nucleolus can be visible (see Fig. 4d, e).

The target downsampling factor is found by dividing the target MPP by the MPP at the highest magnification level of the scan (level 0). The level 0 MPP is accessed from the scan by OpenSlide using the PROPERTY_NAME_MPP_X and PROPERTY_NAME_MPP_Y. In case the directional level 0 MPP are different, the target downsampling factor will also be different in the two directions. If these two properties are not available in the scan, it is not included in the study.

For neural network optimisation and application, we read tile regions from the scan file one by one rather than the entire scan. Each tile is read from the scan at the pyramid level with a corresponding downsampling factor smaller than or equal to the target downsampling factor (or the smallest of the two directional target downsampling factors if they are different). Unless the target downsampling factor is equal to the reading downsampling factor, the size of the read tile will be larger than the target size. The enlarged tile is therefore downsampled to the target size so that the resulting spatial resolution is equal to the target spatial resolution. Downsampling to a target size (instead of to a target factor) also ensures that the resulting tile has the exact height and width we desire (and not e.g. off-by-one due to rounding). This final resizing is performed using OpenCVs resize function with the INTER_AREA interpolation option. This ensures that no upsampling is performed, but may result in tiles being read from the scan at different spatial resolutions depending on the scanner model and settings.

For background exclusion, performance evaluation and display purposes, we use the downsampled scan as a single image, and in these cases the scan is downsampled to a spatial resolution of 5 MPP (about ×2 magnification). Extracting the image from the scan file is done as for the tiles explained in the previous paragraph, with the exception that the target spatial resolution is different and that the entire scan is read all at once instead of in smaller regions.

### Tiling

Because it is important to capture both smaller details and larger context when segmenting tumours, deep learning models utilising tiles at different magnifications have been described previously^7,9^. Instead, we use one set of large tiles sampled at high resolution which allows for a simpler network architecture while still including both details and context. The tile size was determined by hardware constraints and the sampling magnification was chosen to balance high resolution and large physical area.

The horizontal and vertical spatial dimensions are split in the same way, and the procedure for computing tile start and end coordinates is listed as python code in Protocol Listing 1 (Supplementary Section 6). The scan is partitioned into overlapping tiles if the scan dimension is not an integer multiple of the tile dimension and the minimum overlap is not specified to be 0. The amount of overlap is equal between all tile columns in the horizontal direction, except for between the rightmost tile columns which may overlap more, so that the rightmost tile column aligns with the right scan boundary. The same is true in the vertical direction where tile rows overlap with the same amount except perhaps for between the bottommost tile rows. With the procedure shown in Protocol Listing 1 (Supplementary Section 6), we can also specify the minimum number of overlapping pixels along a dimension.

Tiles used for network optimisation have a target spatial dimension of 2048 × 2048 pixels and are sampled from the scan with a minimum overlap of 0 pixels. Tiles used for network inference have a target spatial dimension of 7680 × 7680 pixels with a minimum overlap of 1024 pixels. Inference tiles cover a physical area of 7.68 × 7.68 mm^2^, where 7.68 mm is about one-third of the width of a typical glass slide.

Although the image size difference between tiles used for training and tiles used for inference is quite large in our study, we did not notice any performance issues, which is in line with what is indicated in other studies^10,48^.

Scan tiles are written as JPG files with 95% quality, while annotation mask tiles are written as PNG files. Full scans at 5 MPP are written as PNG files.

### Dataset balancing

The development set was balanced w.r.t. cancer type by oversampling the minority groups on a tissue slide level. Tissue slides were selected multiple times at random without replacement so that no slides were selected *n* + 1 times before all slides had been selected *n* times. This resulted in 3519 sections sampled from each cancer type (the same number of sections included in lung carcinoma, which was the majority group). Counting scans from both scanners, the result was 7030 scans from colorectal carcinoma and 7038 scans from each of the other cancer types. See Protocol Table 10 (Supplementary Section 6) for an overview of the number of scans for each cohort. Note that since the selection was done on a slide level, and cohorts DCo2 and DCo3 had fewer NanoZoomer XR scans than Aperio AT2, there are slightly fewer scans from NanoZoomer XR than from Aperio AT2 in Protocol Table 10 (Supplementary Section 6) for these two cohorts.

### Background segmentation

A simple method is employed to segment the white background in an image from the rest. This background mask is used to alter both predicted and reference segmentation masks. This is useful when large background regions are inside the annotated region (one example being holes from tissue microarray acquisition) without being manually annotated as background. These regions are clearly not cancerous tissue, and should not be annotated as such neither by the reference nor by the prediction.

Note that this segmentation is quite sensitive in that it will mark most tissue as foreground, also adipose tissue that is often left out when applying threshold methods based on image brightness or saturation or similar. But it may also include artefacts such as pen markings, air bubbles, dust, glass cracks, etc. But since the mask is used to exclude white background tiles used in training, it can be an advantage that foreground elements other than tissue is included. The method with the stated parameter values assumes images of H&E-stained tissue with 5 MPP spatial resolution.

Canny edge detection is performed on the input colour image, using the OpenCVs Canny implementation^49^. We use a 3 × 3 Sobel filter for the gradient computation, and thresholds of 10 and 50 for the lower and upper thresholds in the hysteresis. This produce a mask with lots of foreground pixels in regions with structure and lots of background pixels in homogeneous regions.

This foreground mask is refined by first removing small background regions. The mask first undergoes morphological closing (openCV morphologyEx) with a square 9 × 9 structure element before background regions with an area smaller than 10,000 pixels are filled in with the function remove_small_holes from the scikit-image python library. An area of 10,000 pixels at 5 MPP spatial resolution corresponds to a square region of 0.5 × 0.5 mm^2^.

Finally, small foreground regions are removed from the mask. Morphological opening is applied on the mask using the openCV function morphologyEx with the same 9 × 9 structure element, before foreground regions with an area smaller than 1600 pixels are erased using the function remove_small_objects from the scikit-image python library. An area of 1600 pixels corresponds to a square region of 0.2 ×0.2 mm^2^ at 5 MPP spatial resolution.

This method is simple to implement, very robust, and quite fast, spending around one second per image on a single CPU core on consumer-grade hardware. An example of a downscaled scan from colorectal carcinoma scanned with Aperio AT2 and manually annotated is shown in Protocol Fig. 26 (Supplementary Section 6).

With this we can classify every pixel as either white background, foreground without annotation and foreground with annotation. This content classification is summarised in Supplementary Section 5.4 for all scans in all development and validation cohorts.

### Background tile exclusion

Tiles containing too much white background are removed from the development set. The background segmentation is performed on 5 MPP full images as described in *Background segmentation* and transferred to the 1 MPP tiles. Specifically, we include all tiles that contain some tumour annotated regions, and for those that don’t, we keep those with a background fraction smaller than 50%.

In total this reduces the number of unique tiles from 3080330 to 2144651 or from 4233081 to 2902032 non-unique tiles in the balanced dataset (see Protocol Table 11 and Protocol Fig. 31 in Supplementary Section 6).

### Dataset augmentations

Image tiles are read as RGB with 8 bits values per channel, cast to 32 bits floating point values, and then preprocessed before they enter the segmentation network. We artificially augment the training dataset by distorting images using the albumentations library^50^. The operations are listed in Protocol Listing 2 (Supplementary Section 6) in the order they are applied. Note that while the tiles are sampled at a size of 2048 × 2048 pixels, they are cropped to a size of 1536 × 1536 pixels before they enter the network.

Image distortions are only applied during network optimisation, and not when the fixed network is applied.

### Image value standardisation

Before the image enters the network, the image values are divided by 255 before the image is centred around the development dataset mean value and scaled with the development dataset standard deviation. This standardisation is applied both during network optimisation and inference.

The dataset mean value for an image channel is computed as

1 $$\begin{array}{cc}{{\mu }} & =\frac{1}{m}{\sum }_{i=1}^{m}{{{\mu }}}_{i}\\ & =\frac{1}{m}{\sum }_{i=1}^{m}\frac{1}{{n}_{i}}{\sum }_{j=1}^{{n}_{i}}{x}_{{ij}}\end{array}$$

where *x*_*ij*_ is the value at pixel *j* in image *i* for the image channel and *µ*_*i*_ is the mean value in image *i*. *n*_*i*_ is the number of pixels in image *i*, and *m* is the number of images in the dataset. Similarly, the dataset variance for a single channel is estimated as

2 $$\begin{array}{ccc}{{{\sigma }}}^{2} & =\frac{1}{m}{\sum }_{i=1}^{m}{{{\sigma }}}_{i}^{2}\\ & =\frac{1}{m}{\sum }_{i=1}^{m}\frac{1}{{n}_{i}-1}{\sum }_{j=1}^{{n}_{i}}{\left({x}_{{ij}}-{{\rm{\mu }}}_{i}\right)}^{2}.\end{array}$$

We use $\sigma =\sqrt{{\sigma }^{2}}$ as the estimate for the dataset standard deviation. For both estimates *µ* and *σ*, the final result is divided by 255 before it is applied.

When applied on all unique 2048 × 2048-sized tiles in the development dataset at spatial resolution 1 MPP without distortions, we get the result shown in Protocol Table 12 and Protocol Figs. 32 and 33 (Supplementary Section 6). Colour mean and standard deviation distributions for the all scans at spatial resolution 5 MPP are shown in Protocol Figs. 22 to 25 (Supplementary Section 6).

### Segmentation network

The segmentation network is an encoder-decoder network developed using the PyTorch v1.11 machine learning framework^51^.

For the encoder we used a Normalising-free Network (NFNet)^52,53^, a modern classification network designed to achieve state-of-the-art performance without using batch normalisation^54^. More specifically, we use the eca_nfnet_l3 implementation provided by the timm version 0.4.12 package^55^.

This implementation differs from the one described by Brock and colleagues in that it has 4, 8, 24 and 12 blocks for the four stages, respectively. The Squeeze and Excitation module is replaced by the Efficient Channel Attention module^56,57^. It also uses SiLu activation functions instead of GeLu^58^.

The decoder is the decoder from the DeepLabV3+ segmentation network, and the implementation is from the segmentation_models_pytorch python package^59,60^. We modified the DeeplLabV3+ decoder to be free of batch normalisation, following the NFNet encoder. To achieve this, we simply replaced every batch norm layer with a group norm layer with groups of size 8^61^.

The network consists in total of 7,347,2403 adjustable parameters to be optimised (computed by torchinfo). An overview of the architecture can be seen in Protocol Fig. 34 (Supplementary Section 6).

The Normalising-free Network was selected as a candidate encoder because it was advertised to work well without batch normalisation, which we hypothesised would allow for easier optimisation with small batch size. The DeepLabV3+ was selected as a candidate encoder because it is well-known, and because we were able to modify the original network to work well without batch normalisation. Initially, several different encoders and decoders were tested before we arrived at the final network.

### Network optimisation

Below we describe how the network was optimised, but in general we follow the procedure described in by Brock and colleagues in their NFNet paper, with some exceptions^52^. We do not use Adaptive Gradient Clipping as we did not see any benefit for it in our case, perhaps because of our small batch size (24 images). We also do not use moving averages of the model parameters.

The objective is to minimise the difference between the output of the segmentation network and the reference segmentation by iteratively modifying the adjustable parameters of the segmentation network. The difference to be minimised is captured by the loss function *l* = *l*_1_ + *l*_2_, where *l*_1_ is the so-called Dice-loss (DiceLoss from segmentation_models_pytorch with mode = ‘multiclass’), and *l*_2_ is a so-called top-90 Cross Entropy loss function. The top-90 Cross Entropy at a particular step is computed by first computing the per-pixel cross entropy for all pixels in the mini batch of this step and then averaging the cross entropy value over pixels in the top 90 percentile. That is, when computing the mean cross entropy, we are ignoring 10% of pixels with the lowest cross entropy value.

We predict three classes, and the reference is segmented into background, non-annotated foreground, and tumour-annotated foreground. We also experimented with using just two classes, tumour-annotated foreground and everything else, but we did not notice any important difference in performance.

The convolution weights in the encoder are initialised with normal initialisation, while the biases are initialised to zero.

3 $$X{\mathscr{\sim}}{\mathscr{N}}\left(0,{{\rm{\sigma}}}^{2}\right),{\text{ where }}\,\sigma =\sqrt{\frac{1}{{c}_{i}{hw}}}.$$

The convolution weights and biases used in the decoder and segmentation head are initialised with uniform initialisation

4 $$X{\mathscr{\sim }}{\mathscr{U}}\left(-a,a\right),{\text{ where }}\,a=\sqrt{\frac{3}{{c}_{i}{hw}}}.$$

In the above equations, *c*_*i*_*hw* is the volume of the input feature maps in the convolutional layer (number of input channels times the height times the width), often called fan in ref.^62^.

At each iteration (or step), the adjustable network parameters are updated according to the Stochastic Gradient Descent optimisation method with Nesterov momentum 0.9^63^. The optimisation is regularised with a weight decay value of 2 × 10^−5^ with the exceptions described by Brock and colleagues^52^.

A batch of 24 images is randomly selected without replacement from the development dataset and processed at each step. When the dataset is exhausted we say that an epoch is complete, and the selection is reset. The whole batch is processed by the segmentation network before the output is compared with the corresponding reference segmentation batch with the objective function. The batch of 24 is distributed on 8 GPUs with 3 tiles per GPU using pytorchs DistributedDataParallel.

The step length is initialised to 1.0 × 10^−4^ and incremented by 1.0 × 10^−4^ every 10th step until step 1000 when the step length has reached 1.0 × 10^−2^. After this warm up period, the step length follows a cosine annealing schedule until termination (see Protocol Fig. 35, Supplementary Section 6)^64^.

The optimisation is carried out for 500,000 steps (or 4.14 epochs) before termination. Since we have 2,902,032 tiles in the dataset and 24 tiles per batch, we have 120,918 steps per epoch. The model at step 500,000 is selected as the model used in the segmentation method.

We employ Automatic mixed precision both during optimisation of the network and when applying it. This is provided by the torch.cuda.amp module in the pytorch python package.

Loss curves from the network optimisation are displayed in Supplementary Section 3. Training one network took around 150 h of elapsed real time.

### Method application

Application of the method on a single input scan is illustrated in Fig. 3 and can be summarised as

1. Read the input scan at 1 MPP spatial resolution
2. Partition the downsampled scan into overlapping tiles
3. Apply the optimised segmentation network on each tile
4. Construct a probability image from the segmentation network tiles (*Reconstruction from tiles*)
5. Post-process to yield a final segmentation mask (*Result post-processing*)

Scan reading and downsampling is done as described in *Downsampling*. Tiling is done as described in *Tiling*, with tile size of 7680 × 7680 pixels with a minimum overlap of 1024 pixels in each direction (see example in Protocol Fig. 36, Supplementary Section 6).

Input images are processed with the optimised segmentation network after the following operations are applied on the input image

1. Read image as RGB with 8-bit values in each channel
2. Zero-pad image so that both the image height and width are divisible by 16. This step is not necessary for this particular setup since we have tiles with size 7680 × 7680, but is included for making the method applicable in the general case with varying input sizes.
3. Scale image values to [0,1] by dividing by 255
4. Subtract the image from the development dataset mean (Protocol Table 12, Supplementary Section 6)
5. Divide image by development dataset standard deviation (Protocol Table 12, Supplementary Section 6)

The resulting prediction from the segmentation network is an image with one channel per output class, where only the channel corresponding to the tumour class is used further. Its values are floats where pixel value 0 indicates negative prediction and 1 indicate positive prediction. The image values are multiplied by 255 before the image is quantised to 8 bits. The padding (if any) is removed before the prediction is written as a PNG image.

### Reconstruction from tiles

The final reconstructed image *f* is computed as $f={\sum }_{i}{w}_{i}{g}_{i}$ where *f, w*_*i*_*, g*_*i*_ are *m* × *n* matrices and *i* iterates over all tiles. *g*_*i*_ represent a single tile output from the segmentation network, and has the output tile value in the tile location and value zero everywhere else. *w*_*i*_ represents a single weight tile which has values in the corresponding tile location and value zero everywhere else. *w*_*i*_ have values in [0, 1] and *g*_*i*_ have integer values in [0, 255] since they have been written as 8-bit PNG files by the segmentation network. The values of *f* are quantised to integer values by rounding with the tie-breaking rule of rounding half to even before *f* is written as PNG.

The weight tiles are constructed so that the sum weight image $s={\sum }_{i}{w}_{i}$ with shape *m* × *n* will have value 1 in all pixels. In the rest of this explanation a weight tile and image tile will refer only to the part of *w*_*i*_ and *g*_*i*_ that correspond to the location of each tile, respectively.

The tile weights are constructed in three phases, and an example result is shown in Protocol Fig. 37 (Supplementary Section 6). First, initial weight tiles are computed for each image tile. These weight tiles are weighted by distance in overlapping regions. A sum image the same size of *f* is constructed by adding all initial weight tiles *w* at their locations within this sum image. Each initial weight tile is normalised by dividing it by the tile cropped out from its location within the sum image. The next two paragraphs explain the construction of the initial weight tiles.

An initial weight tile *w* is computed as the element-wise product of four side-specific weight tiles: *w*_*t*_ weighting overlaps at the top of *w*, *w*_*b*_ weighting overlaps at the bottom of *w*, *w*_*l*_ weighting overlaps at the left of *w* and *w*_*r*_ weighting overlaps at the right of *w*.

In order to compute a side-specific weight tile, e.g. *w*_*r*_, the smallest leftmost coordinate of all overlapping tiles with a leftmost coordinate greater than the leftmost coordinate in *w* is recorded. The region between this recorded coordinate and the rightmost coordinate of *w* defines the overlapping area to the right in *w*.

All pixels in *w*_*r*_ to the left of this overlapping area are given value 1, and all other pixels are giving a value decreasing linearly with the distance from the left overlapping border $v=1-\frac{d}{1+l}$ where *v* is the result value, *d* is the distance from the left overlapping border, and *l* is the length of the overlapping region. Both *d* and *l* are measured in pixels. The procedure and weighting are similar for the other side-specific weight tiles.

### Result post-processing

Post-processing is used to transform the segmentation network output probability maps to binary foreground and background masks. The process comprises three steps

1. Smooth the probability map
2. Binarize the smoothed probability map
3. Clean the binarised mask

The merged probability map from *Reconstruction from tiles* has the same size as the 1 MPP scan image they originate from. Before further post-processing, this probability map is downsampled by five times in both horizontal and vertical directions (corresponding to the scan image at 5 MPP).

We apply smoothing of the probability map both to get a smooth segmentation boundary in the final segmentation, and to reduce the impact of noise in the post-processing. For the sake of efficiency, the probability map is further downsampled before smoothing and upsampled again after smoothing is done. The downsampling factor is set to 0.2 for both the vertical and the horizontal direction unless the resulting image has an area less than 10^6^, in which case then the image is resized to have an area of 10^6^. This threshold is arbitrarily chosen as a safeguard against very small scans. Specifically, the new height and width is found by multiplication with a factor $\max \{0.2,\sqrt{{10}^{6}/\left({hw}\right)}\}$ where *hw* is the area of the input. Then the resulting float value is floored to get an integer value. The image then undergoes median blurring with an aperture size of 9 using OpenCVs medianBlur function. Next, the result is further smoothed using OpenCVs GaussianBlur function with a kernel size of 5 × 5. Finally, the smoothed probability map is upsampled back to the original size corresponding to the 5 MPP scan image.

The smooth probability map is then dichotomised into foreground and background using a hysteresis threshold method. The lower threshold value is set to 85 (1/3 of 255) and the higher threshold value is set to 229 (≈90% of 255).

Finally, foreground regions in the mask are pruned with the following procedure. For each connected foreground region in the foreground mask, collect the values the region covers in the smooth probability map. If the 95th percentile value of this collection is greater than 229, the corresponding region is kept as foreground, else it is labelled background.

All pixels not foreground in both the foreground mask from the probability map and the foreground mask from the scan image (*Background segmentation*) are labelled as background. The resulting mask is further processed by removing small background regions and then small foreground regions, as explained in *Background segmentation* for the foreground mask.

### Performance evaluation

To measure the similarity between the reference and predicted segmentation, we use different metrics to highlight different similarities.

Since we employ the same background exclusion on both reference and prediction masks, it is of little interest to count true negative pixels in the white background area of a scan. We therefore excluded background in the performance evaluation. True negatives are therefore pixels that are marked as background in the prediction and neither as tumour nor background in the reference mask.

For simple overlap comparison, we partition the pixels based on how they overlap in the reference and predicted segmentation:

*N* = Pixel count in the image after excluding white background

*RP* = |{*x* : *x* is foreground in reference}|

*RN* = |{*x* : *x* is background in reference}|

*PP* = |{*x* : *x* is foreground in prediction}|

*PN* = |{*x* : *x* is background in prediction}|

*TP* = |{*x* : *x* is foreground in reference and prediction}|

*FN* = |{*x* : *x* is foreground in reference and background in prediction}|

*FP* = |{*x* : *x* is background in reference and foreground in prediction}|

*TN* = |{*x* : *x* is background in reference and prediction}|

These counts comprise a contingency table termed a confusion matrix (Protocol Table 13, Supplementary Section 6). We can derive different metrics from the confusion matrix to measure different features of the segmentation result. Some common metrics that are used in this work are presented in the following.

True positive rate or sensitivity or recall measures the fraction of reference foreground pixels that are correctly marked as foreground

5 $$TPR\,{=}\,\frac{{T}{P}}{{T}{P}{+}{F}{N}}$$

False negative rate measures the fraction of reference foreground pixels that are wrongly marked as background

6 $$FNR\,{=}\,\frac{{F}{N}}{{T}{P}{+}{F}{N}}$$

True negative rate or specificity measures the fraction of reference background pixels that are correctly marked as background

7 $$TNR\,{=}\,\frac{{T}{N}}{{T}{N}{+}{F}{P}}$$

False positive rate measures the fraction of reference background pixels that are wrongly marked as foreground

8 $$FPR\,{=}\,\frac{{F}{P}}{{T}{N}{+}{F}{P}}$$

Positive predictive value or precision measures the fraction of predicted foreground pixels that are correctly marked as foreground

9 $$PPV\,{=}\,\frac{{T}{P}}{{T}{P}{+}{F}{P}}$$

Negative predictive value measures the fraction of predicted background pixels that are correctly marked as background

10 $$NPV\,{=}\,\frac{{T}{N}}{{T}{N}{+}{F}{N}}$$

Informedness

11 $$BIN\,{=}\,TPR{+}TNR{-}{1}$$

Markedness

12 $$BMA\,{=}\,PPV\,{+}\,NPV\,{-}\,{1}$$

Matthew’s correlation coefficient is the geometric mean of informedness and markedness

13 $$MCC\,{=}\,\frac{TP\times TN-FN\times FP}{\sqrt{{(}{T}{P}{+}{F}{N}{)}{(}{T}{P}{+}{F}{P}{)}{(}{T}{N}{+}{F}{N}{)}{(}{T}{N}{+}{F}{P}{)}}}$$

Sørensen-Dice similarity coefficient or *F*_1_ score is the harmonic mean of the true positive rate and the positive predictive value

14 $$DSC=\frac{{2}TP}{{2}TP{+}FN{+}FP}$$

### Analyses

This study contains both planned and exploratory analyses. The planned analyses are stated in full in Protocol Section 3 (Supplementary Section 6) and summarised in this section. In addition, a set of exploratory analyses were performed post-hoc after the study protocol was fixed and validation results were ready.

### Planned primary analysis

The primary analysis evaluates the performance of the primary model in all Aperio AT2 scans in each validation cohort. The DSC was selected as the primary performance metric since it is commonly used and suitable for measuring overall segmentation quality^65–67^. The DSC equals two times the number of foreground pixels common in the predicted mask and the corresponding reference mask, divided by the sum of foreground pixels in the predicted mask and the foreground pixels in the reference mask. It ranges from 0 (no common foreground pixels) to 1 (all pixels are classified equally in the prediction and reference). Performance is reported per cohort as the cohort-average DSC with an accompanying 95% confidence interval (CI) computed using a Student’s *t* statistic.

### Planned secondary analyses

Four secondary analyses were planned. The first analysis further illuminates the performance of the primary model in the validation cohorts by computing 11 additional contingency table summary statistics (fraction of reference positives, fraction of predicted positives, and eqs. (5) to (13)). The second analysis investigates how the primary model performs in scans from NanoZoomer XR. The third analysis compares the primary model with models specialised on a single cancer type. The specialised colorectal model was trained using only the cohorts from colorectal carcinoma (DCo1, DCo2 and DCo3), and vice versa for the specialised endometrial, lung, and prostate models. Finally, the primary model is compared to replication models that are developed identically as the primary model, except with different random seeds.

### Exploratory analysis 1: correlation between segmentation performance and cohort characteristics

Associations between the resulting DSC and other data characteristics are measured using Spearman’s rank correlation coefficient, *ρ* (*Statistical analysis*). Results are presented in Supplementary Section 2.1.

### Exploratory analysis 2: per-scan performance comparison

The performance of the primary model on the Aperio AT2 scans from the validation cohorts are presented on a per-scan level to supplement the average results obtained from the pre-planned secondary analyses. For all validation cohorts, we compare the primary model on Aperio AT2 with (1) the primary model on NanoZoomer XR, (2) the first replication model on Aperio AT2, (3) the second replication model on Aperio AT2. We also compare the primary model on Aperio AT2 with the specialised models on Aperio AT2 in the validation cohorts from the cancer type they were specialised in: the colorectal model in VCo1, the endometrial model in VEn1 and VEn2, the lung model in VLu1, and the prostate model in VPr1. Results are presented in Supplementary Fig. S17.

### Exploratory analysis 3: region level performance

A region is a set of 4-connected foreground pixels in the predicted or reference segmentation mask. For each detected reference region, we locate predicted regions that are overlapping. We say that a reference region and a predicted region *correspond* if they have an intersection over union (Jaccard index) greater than 50%. This ensures that if a reference region correspond with a predicted region, it cannot correspond with any other predicted regions. Also, this guarantees that the predicted region also only corresponds with the same reference region. Note that the above definition of corresponding regions only considers single regions, which labels predicted regions that would correspond to a union of smaller reference regions as false positive, and reference regions that would correspond to a union of smaller predicted regions as false negative.

The true positive reference regions are then the set of reference regions that have a corresponding predicted region, and vice versa. A false negative reference region is a reference region not included in the set of true positive reference regions. A false positive predicted region is a predicted region not included in the set of true positive predicted regions.

Supplementary Table S9 shows pixel overlap measured with DSC between the prediction and reference when only considering true positive regions. We get a DSC for each image by adding the contingency tables for all pairs of corresponding regions in the image (we therefore only get a result for an image if this image contains at least one pair of corresponding regions). The DSC is then averaged over all images within a cohort with at least one pair of corresponding regions.

Supplementary Fig. S18 shows the distribution of regions and their size in an image. Reference regions smaller than 1600 pixels are discarded since they are artefacts of the background segmentation (Protocol Section 2.2.4, Supplementary Section 6).

### Exploratory analyses 4 and 5: performance in TCGA and development cohorts

Exploratory analysis 4 evaluates the performance of the primary model applied on WSIs from TCGA. Exploratory analysis 5 evaluates the performance of the primary model applied on WSIs from the development cohorts. Results are presented together with the main validation cohort results in Fig. 5.

### Exploratory analysis 6: subgroup analyses in bladder cohorts

We investigate the performance of the primary model in subgroups of the two bladder cohorts, VUr1 and BLCA. In BLCA, a pathologist (MP) noted for each scan whether it was likely to originate from TUR or not by considering the presence of fragmented tissue sections in the imaged glass slide. Performance was measured in pT stage groups and fragmented tissue groups with DSC averaged both over all scans and only in scans with a prediction. Results are presented in Supplementary Table S10.

### Exploratory analysis 7: pathologist intra- and inter-observer variability

We tasked pathologists Manohar Pradhan (MP) and Ljiljana Vlatkovic (LV) to annotate tumour regions in the VBr2 validation cohort. MP had already annotated the scans in this cohort, about two years prior to this second annotation round. In this section, we let *MP-1* refer to MP’s first set of annotations, and *MP-2* to his second set of annotations.

LV is a retired uropathologist currently serving as a consultant at the Institute for Cancer Genetics and Informatics, Oslo University Hospital, Norway. She holds a master’s degree in cytology from the University Hospital Centre Zagreb, Croatia, in addition to her specialisation in pathology. She has over 40 years of experience and has contributed to over 50 research papers throughout her career.

MP is a pathologist employed at the Institute for Cancer Genetics and Informatics, Oslo University Hospital. He holds a PhD in image cytometry from the University of Oslo, Norway, in addition to his specialisation in pathology. He has over 20 years of experience as a pathologist and has contributed to over 30 research papers throughout his career. MP was involved in all manual annotations of the development and validation materials used in this study (Protocol Sections 1.1 and 1.2, Supplementary Section 6).

Both were given instructions to provide a rough delineation of all tumour areas, including infiltrating tumour areas and intraductal carcinoma. In situ carcinoma, atypical ductal hyperplasia, and lobular hyperplasias were also included. These are the same instructions given to MP in his initial annotation round. In this experiment, MP and LV did not look at the existing annotations, and they did not consult each other on how to annotate if they encountered uncertainties.

Measured differences will capture where the pathologists disagree, where they chose differently in decisions on doubtful regions, and their general difference in annotation ‘style’. The result of this experiment is simply a quantification of the similarities between the annotations in this cohort, and does not give any indication of which annotation is the most ‘correct’. Although this result will give a measure of intra- and inter-observer variability, it was performed primarily to contextualise the values of the DSC.

Referring to the results in Supplementary Fig. S19, *MP-2 vs MP-1* will give an indication of the intra-observer variability with a separation of two years, while *LV vs MP-1* and *LV vs MP-2* will indicate inter-observer variability. Measured similarity with the primary automatic model presented in this study (labelled *Auto*) is also included for reference.

### Exploratory analysis 8: primary model performance in five different scanners

Slides from VCo1 were scanned on three different scanners in addition to Aperio AT2 and NanoZoomer XR: Aperio GT 450 DX (Leica Biosystems, Germany), KF-PRO-400 (KFBIO, China) and Pannoramic 1000 (3DHISTECH, Hungary)

All slides were scanned on the KF-PRO-400 scanner, two were not scanned on Aperio GT 450 DX, and an additional slide was not scanned on Pannoramic 1000. In all three cases, the reason for not scanning was that parts of the glass slide were broken.

Before scanning on the three additional scanners, 39 included tissue sections were restained because of weak staining in the original sections. The 39 glass slides with restained tissue sections were also scanned on the Aperio AT2 and NanoZoomer XR scanners. The DSC was similar between the original and the restained version in all 39 sections on both Aperio AT2 and NanoZoomer XR, except for one section that originally incorrectly produced no predicted tumour regions (see Supplementary Fig. S20).

All experiments presented in this section evaluate the 1152 slides that were scanned on all scanners, with 39 tissue sections that were restained and therefore differ from the corresponding 39 original tissue sections from VCo1 evaluated elsewhere in this study. Note that, although the same glass slides were scanned on the different scanners, they were not scanned at the same time. In general, the slides were scanned on Aperio AT2 and NanoZoomer XR in 2018, on Aperio GT 450 DX and KF-PRO-400 in 2023, and on Pannoramic 1000 in 2024.

Per-scan differences in DSC are presented in Supplementary Fig. S21 and statistics on the performance per scanner are summarised in Supplementary Table S12.

### Exploratory analysis 9: MedSAM segmentation performance in validation cohorts

The MedSAM model is applied on all validation cohorts and compared with the primary model presented in this study^22^. Whole-slide images are downscaled to a spatial resolution of 5 µm per pixel before input to MedSAM, and the resulting probability image is dichotomised with the same hysteresis thresholding used by the primary method in this study. We both evaluate MedSAM prompted with the bounding box of the tissue area, and prompted with the bounding box of the manually annotated tumour area. See results in Supplementary Section 2.7.

### Exploratory analysis 10: varying hysteresis thresholds in probability image segmentation

This experiment measures segmentation performance with varying hysteresis threshold values in the probability image segmentation. As in the original post-processing, this experiment also thresholds the smoothed probability image, but the subsequent pruning is disabled. Performance is measured with DSC between the automatic segmentation using the primary model and manual segmentation. The dataset evaluated is in Aperio AT2 scans from VUr1. Lower thresholds take values in [5%, 10%*, …*, 90%], and for each low threshold *x*, we have the higher thresholds [*x* + 5%*, x* + 10%*, …*, 95%]. See results in Supplementary Section 2.8.

### Statistical analysis

The Spearman’s rank correlation coefficient, *ρ* is computed using the Pearson’s sample correlation coefficient *r* applied on the rank of the variables. *P* values are computed using

15 $$t=r\sqrt{\frac{n-2}{1-{r}^{2}}}$$

which is approximately Student’s *t*-distributed with *n* − 2 degrees of freedom under the null hypothesis *ρ* = 0 where *n* is the number of samples. A two-sided *p* value below 0.05 was considered statistically significant. Correlation and *p* values are computed using the scipy.stats.spearmanr function with scipy version 1.10.1 and Python version 3.11.3^68^. The confidence interval is computed using that

16 $$z={\rm{arctanh}}\left(r\right)$$

is approximately normally distributed and has a standard error of approximately $1/\sqrt{n-3}$^69^. A $100\left(1-{\rm{\alpha }}\right)$% confidence interval is then

17 $${\mathrm{tanh}}\left({\mathrm{arctanh}}\left(r\right)\pm {z}_{1-{\rm{\alpha }}/2}\frac{1}{\sqrt{n-3}}\right).$$

## Supplementary information

Supplementary information

## Data Availability

The source code is made available at https://github.com/icgi/automatic-tumour-segmentation-in-WSIs.

## Data availability

Materials from TCGA can be downloaded from the TCGA Research Network (https://www.cancer.gov/tcga). Individual patient-level data from the other materials can be made available to other researchers upon reasonable request by contacting the corresponding author, subject to approval by the relevant people or review board at the institutions that provided the original data.
