# Supplementary material for: Generalisation of automatic tumour segmentation in histopathological whole-slide images across multiple cancer types
Source: NPJ Precis Oncol. 2026 Feb 4;10:107. doi: 10.1038/s41698-026-01311-6 (PMC12976075; doi:10.1038/s41698-026-01311-6)
Supplement: Supplementary file 1 — Supplementary information [file 41698_2026_1311_MOESM1_ESM.pdf]

Supplementary Information

Generalisation of automatic tumour segmentation in histopathological whole-slide images across multiple cancer types

Contents

1 Results from pre-planned analyses **1**
1.1 Primary model performance in Aperio AT2 (study protocol section 3.1 and 3.2.1) 1
1.2 Primary model performance in NanoZoomer XR (study protocol section 3.2.2) 3
1.3 Cancer type-specialised models performance (study protocol section 3.2.3) 5
1.3.1 Colorectal carcinoma 5
1.3.2 Endometrial carcinoma 7
1.3.3 Lung carcinoma 9
1.3.4 Prostate carcinoma 11
1.4 Primary model replication performance (study protocol section 3.2.4) 13
1.4.1 Replication 1 13
1.4.2 Replication 2 15

2 Results from exploratory analyses **17**
2.1 Association analyses in primary model 17
2.2 Per-scan comparison 25
2.3 Region areas 26
2.4 Bladder subgroup analysis 28
2.5 Intra- and inter-observer variability 29
2.6 Performance evaluation in five different scanners 30
2.7 Comparison with MedSAM 32
2.8 Varying hysteresis threshold in segmentation post-processing 34

3 Segmentation network optimisation **35**

4 TCGA cohorts **36**
4.1 Included scans 36
4.2 Baseline characteristics 38
4.3 WSI dimensions 40

5 Protocol amendment **42**
5.1 Protocol section 1.1.4 DEn1 — Endometrial carcinoma 42
5.2 Protocol section 1.2.8 VUr1 — Urothelial carcinoma 42
5.3 Protocol Table 5 and protocol Fig. 18 42
5.4 Protocol Figs. 27, 28, 29, 30 43

6 Study protocol **49**

List of Figures

S1 Primary model performance on Aperio AT2 scans . . . 2
S2 Primary model performance on NanoZoomer XR scans . . . 4
S3 Colorectal model performance on Aperio AT2 scans . . . 6
S4 Endometrial model performance on Aperio AT2 scans . . . 8
S5 Lung model performance on Aperio AT2 scans . . . 10
S6 Prostate model performance on Aperio AT2 scans . . . 12
S7 First primary model replication performance on Aperio AT2 scans . . . 14
S8 Second primary model replication performance on Aperio AT2 scans . . . 16
S9 Associations VCo1 . . . 17
S10 Associations VEn1 . . . 18
S11 Associations VEn2 . . . 19
S12 Associations VLu1 . . . 20
S13 Associations VPr1 . . . 21
S14 Associations VBr1 . . . 22
S15 Associations VBr2 . . . 23
S16 Associations VUr1 . . . 24
S17 Per scan comparison . . . 25
S18 Region areas . . . 27
S19 Intra- and inter-observer variability in VBr2 . . . 29
S20 Original vs restained mean difference . . . 30
S21 Scanner vs scanner mean difference . . . 31
S22 MedSAM performance in validation datasets . . . 33
S23 Varying hysteresis threshold . . . 34
S24 Segmentation network optimisation loss curve . . . 35
S25 Acquisition overview LUAD . . . 36
S26 Acquisition overview LUSC . . . 36
S27 Acquisition overview PRAD . . . 37
S28 Acquisition overview BLCA . . . 37
S29 Kaplan-Meier analysis in materials from TCGA lung carcinoma . . . 39
S30 TCGA WSI dimensions at 20× magnification . . . 40
S31 TCGA WSI objective power and µm per pixel . . . 41
S32 Kaplan-Meier analysis in materials from lung carcinoma . . . 42
S33 Scan area Aperio AT2 . . . 44
S34 Scan area NanoZoomer XR . . . 45
S35 Scan content prevalence Aperio AT2 . . . 46
S36 Scan content prevalence NanoZoomer XR . . . 47

List of Tables

S1 Primary model performance on Aperio AT2 scans 1
S2 Primary model performance on NanoZoomer XR scans 3
S3 Colorectal model performance on Aperio AT2 scans 5
S4 Endometrial model performance on Aperio AT2 scans 7
S5 Lung model performance on Aperio AT2 scans 9
S6 Prostate model performance on Aperio AT2 scans 11
S7 First primary model replication performance on Aperio AT2 scans 13
S8 Second primary model replication performance on Aperio AT2 scans 15
S9 Performance in true positive regions 26
S10 Subgroup analyses in bladder cohorts 28
S11 Intra- and inter-observer variability in VBr2 29
S12 Primary model performance on five different scanners 30
S13 MedSAM performance in validation datasets 32
S14 Best performance in VUr1 with varying hysteresis thresholds 34
S15 Baseline characteristics in materials from TCGA 38
S16 Baseline characteristics in materials from lung carcinoma 43

1 Results from pre-planned analyses

1.1 Primary model performance in Aperio AT2 (study protocol section 3.1 and 3.2.1)

Table S1: Primary model performance on Aperio AT2 scans
Data entries show mean value (95% CI)

Cohort	Prevalence	Bias
VCo1	0.3410 (0.3315 – 0.3505)	0.3682 (0.3583 – 0.3781)
VEn1	0.4924 (0.4363 – 0.5485)	0.4912 (0.4362 – 0.5462)
VEn2	0.3970 (0.3611 – 0.4328)	0.4065 (0.3708 – 0.4422)
VLu1	0.3380 (0.3208 – 0.3552)	0.3891 (0.3703 – 0.4079)
VPr1	0.1777 (0.1671 – 0.1882)	0.1775 (0.1670 – 0.1881)
VBr1	0.2278 (0.2089 – 0.2467)	0.2114 (0.1933 – 0.2295)
VBr2	0.3081 (0.2808 – 0.3355)	0.2938 (0.2674 – 0.3201)
VUr1	0.5304 (0.5004 – 0.5603)	0.2843 (0.2533 – 0.3154)
Cohort	True positive rate	False negative rate
VCo1	0.8928 (0.8852 – 0.9004)	0.1072 (0.0996 – 0.1148)
VEn1	0.9568 (0.9460 – 0.9677)	0.0432 (0.0323 – 0.0540)
VEn2	0.9553 (0.9490 – 0.9616)	0.0447 (0.0384 – 0.0510)
VLu1	0.9060 (0.8895 – 0.9224)	0.0940 (0.0776 – 0.1105)
VPr1	0.8530 (0.8420 – 0.8640)	0.1470 (0.1360 – 0.1580)
VBr1	0.8249 (0.8005 – 0.8494)	0.1751 (0.1506 – 0.1995)
VBr2	0.8779 (0.8609 – 0.8950)	0.1221 (0.1050 – 0.1391)
VUr1	0.4405 (0.4016 – 0.4794)	0.5595 (0.5206 – 0.5984)
Cohort	True negative rate	False positive rate
VCo1	0.8935 (0.8883 – 0.8987)	0.1065 (0.1013 – 0.1117)
VEn1	0.9187 (0.8887 – 0.9486)	0.0813 (0.0514 – 0.1113)
VEn2	0.9286 (0.9099 – 0.9473)	0.0714 (0.0527 – 0.0901)
VLu1	0.8760 (0.8660 – 0.8859)	0.1240 (0.1141 – 0.1340)
VPr1	0.9722 (0.9693 – 0.9750)	0.0278 (0.0250 – 0.0307)
VBr1	0.9741 (0.9689 – 0.9793)	0.0259 (0.0207 – 0.0311)
VBr2	0.9610 (0.9531 – 0.9690)	0.0390 (0.0310 – 0.0469)
VUr1	0.9443 (0.9340 – 0.9546)	0.0557 (0.0454 – 0.0660)
Cohort	Positive predictive value	Negative predictive value
VCo1	0.8138 (0.8062 – 0.8213)	0.9380 (0.9336 – 0.9423)
VEn1	0.9523 (0.9374 – 0.9672)	0.9132 (0.8783 – 0.9480)
VEn2	0.9211 (0.9055 – 0.9366)	0.9504 (0.9367 – 0.9641)
VLu1	0.7686 (0.7508 – 0.7864)	0.9640 (0.9597 – 0.9684)
VPr1	0.8492 (0.8382 – 0.8601)	0.9721 (0.9691 – 0.9750)
VBr1	0.8627 (0.8374 – 0.8880)	0.9523 (0.9442 – 0.9604)
VBr2	0.9018 (0.8845 – 0.9191)	0.9324 (0.9192 – 0.9456)
VUr1	0.6280 (0.5800 – 0.6759)	0.6139 (0.5860 – 0.6419)
Cohort	Informedness	Markedness
VCo1	0.7863 (0.7780 – 0.7947)	0.7517 (0.7432 – 0.7603)
VEn1	0.8755 (0.8441 – 0.9070)	0.8655 (0.8297 – 0.9013)
VEn2	0.8839 (0.8653 – 0.9026)	0.8715 (0.8528 – 0.8902)
VLu1	0.7819 (0.7653 – 0.7985)	0.7327 (0.7142 – 0.7511)
VPr1	0.8252 (0.8144 – 0.8360)	0.8212 (0.8104 – 0.8320)
VBr1	0.7990 (0.7745 – 0.8236)	0.8150 (0.7871 – 0.8429)
VBr2	0.8390 (0.8214 – 0.8566)	0.8342 (0.8120 – 0.8564)
VUr1	0.3848 (0.3508 – 0.4189)	0.2419 (0.1855 – 0.2983)
Cohort	Matthews corr. coeff.	Dice similarity coeff.
VCo1	0.7683 (0.7605 – 0.7761)	0.8454 (0.8386 – 0.8523)
VEn1	0.8686 (0.8349 – 0.9022)	0.9528 (0.9420 – 0.9636)
VEn2	0.8753 (0.8576 – 0.8930)	0.9340 (0.9231 – 0.9449)
VLu1	0.7551 (0.7387 – 0.7714)	0.8222 (0.8054 – 0.8391)
VPr1	0.8203 (0.8103 – 0.8304)	0.8436 (0.8333 – 0.8538)
VBr1	0.8021 (0.7782 – 0.8260)	0.8240 (0.7994 – 0.8486)
VBr2	0.8351 (0.8176 – 0.8526)	0.8816 (0.8652 – 0.8980)
VUr1	0.3691 (0.3369 – 0.4014)	0.4977 (0.4564 – 0.5390)

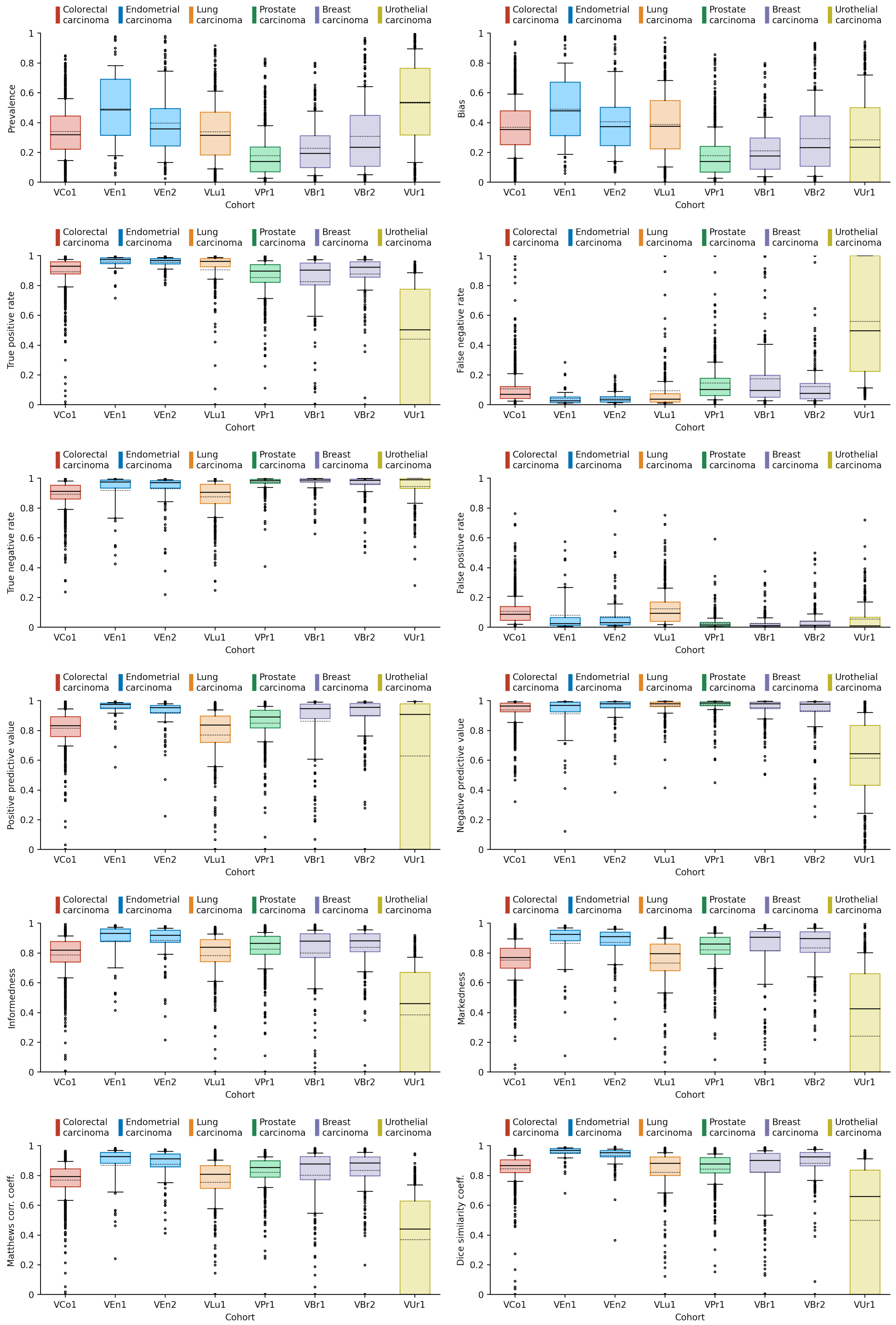

Figure S1: Primary model performance on Aperio AT2 scans
For each cohort, the plot displays the interquartile range (coloured box), mean value (perforated horizontal line), median value (solid horizontal line), the 10th and 90th percentile (whiskers), and outliers (black circles).

1.2 Primary model performance in NanoZoomer XR (study protocol section 3.2.2)

Table S2: Primary model performance on NanoZoomer XR scans
Data entries show mean value (95% CI)

Cohort	Prevalence	Bias
VCo1	0.3411 (0.3316 – 0.3505)	0.3695 (0.3596 – 0.3794)
VEn1	0.4887 (0.4328 – 0.5447)	0.4924 (0.4367 – 0.5481)
VEn2	0.3950 (0.3593 – 0.4307)	0.4044 (0.3691 – 0.4398)
VLu1	0.3363 (0.3192 – 0.3534)	0.3841 (0.3655 – 0.4026)
VPr1	0.1763 (0.1658 – 0.1868)	0.1791 (0.1686 – 0.1897)
VBr1	0.2421 (0.2229 – 0.2614)	0.2232 (0.2043 – 0.2420)
VBr2	0.3262 (0.2989 – 0.3534)	0.3126 (0.2862 – 0.3390)
VUr1	0.5292 (0.4994 – 0.5590)	0.2423 (0.2134 – 0.2712)
**Cohort**	**True positive rate**	**False negative rate**
VCo1	0.8894 (0.8815 – 0.8974)	0.1097 (0.1019 – 0.1175)
VEn1	0.9625 (0.9534 – 0.9716)	0.0375 (0.0284 – 0.0466)
VEn2	0.9555 (0.9491 – 0.9619)	0.0445 (0.0381 – 0.0509)
VLu1	0.9016 (0.8854 – 0.9179)	0.0984 (0.0821 – 0.1146)
VPr1	0.8568 (0.8454 – 0.8682)	0.1432 (0.1318 – 0.1546)
VBr1	0.8117 (0.7849 – 0.8385)	0.1883 (0.1615 – 0.2151)
VBr2	0.8696 (0.8497 – 0.8895)	0.1304 (0.1105 – 0.1503)
VUr1	0.3806 (0.3433 – 0.4178)	0.6194 (0.5822 – 0.6567)
**Cohort**	**True negative rate**	**False positive rate**
VCo1	0.8913 (0.8861 – 0.8965)	0.1087 (0.1035 – 0.1139)
VEn1	0.9131 (0.8817 – 0.9445)	0.0869 (0.0555 – 0.1183)
VEn2	0.9301 (0.9117 – 0.9484)	0.0699 (0.0516 – 0.0883)
VLu1	0.8809 (0.8715 – 0.8903)	0.1191 (0.1097 – 0.1285)
VPr1	0.9698 (0.9668 – 0.9728)	0.0302 (0.0272 – 0.0332)
VBr1	0.9712 (0.9654 – 0.9770)	0.0288 (0.0230 – 0.0346)
VBr2	0.9563 (0.9486 – 0.9640)	0.0437 (0.0360 – 0.0514)
VUr1	0.9597 (0.9511 – 0.9683)	0.0403 (0.0317 – 0.0489)
**Cohort**	**Positive predictive value**	**Negative predictive value**
VCo1	0.8101 (0.8025 – 0.8178)	0.9380 (0.9337 – 0.9423)
VEn1	0.9484 (0.9329 – 0.9639)	0.9208 (0.8894 – 0.9523)
VEn2	0.9183 (0.9015 – 0.9350)	0.9504 (0.9366 – 0.9641)
VLu1	0.7716 (0.7541 – 0.7891)	0.9624 (0.9579 – 0.9668)
VPr1	0.8369 (0.8252 – 0.8486)	0.9736 (0.9707 – 0.9764)
VBr1	0.8474 (0.8191 – 0.8756)	0.9467 (0.9380 – 0.9554)
VBr2	0.8885 (0.8682 – 0.9088)	0.9282 (0.9150 – 0.9414)
VUr1	0.6099 (0.5606 – 0.6593)	0.5905 (0.5622 – 0.6188)
**Cohort**	**Informedness**	**Markedness**
VCo1	0.7807 (0.7721 – 0.7893)	0.7481 (0.7395 – 0.7567)
VEn1	0.8756 (0.8433 – 0.9079)	0.8692 (0.8361 – 0.9023)
VEn2	0.8855 (0.8671 – 0.9039)	0.8686 (0.8491 – 0.8882)
VLu1	0.7825 (0.7663 – 0.7987)	0.7340 (0.7159 – 0.7521)
VPr1	0.8266 (0.8154 – 0.8378)	0.8105 (0.7989 – 0.8221)
VBr1	0.7829 (0.7561 – 0.8097)	0.7941 (0.7625 – 0.8257)
VBr2	0.8259 (0.8060 – 0.8457)	0.8167 (0.7924 – 0.8411)
VUr1	0.3403 (0.3070 – 0.3735)	0.2004 (0.1433 – 0.2575)
**Cohort**	**Matthews corr. coeff.**	**Dice similarity coeff.**
VCo1	0.7637 (0.7558 – 0.7715)	0.8417 (0.8346 – 0.8488)
VEn1	0.8707 (0.8386 – 0.9028)	0.9535 (0.9430 – 0.9640)
VEn2	0.8743 (0.8564 – 0.8922)	0.9320 (0.9203 – 0.9437)
VLu1	0.7559 (0.7399 – 0.7719)	0.8216 (0.8050 – 0.8382)
VPr1	0.8152 (0.8045 – 0.8259)	0.8382 (0.8272 – 0.8492)
VBr1	0.7855 (0.7589 – 0.8121)	0.8101 (0.7828 – 0.8373)
VBr2	0.8201 (0.8004 – 0.8398)	0.8700 (0.8507 – 0.8892)
VUr1	0.3328 (0.3012 – 0.3644)	0.4453 (0.4048 – 0.4858)

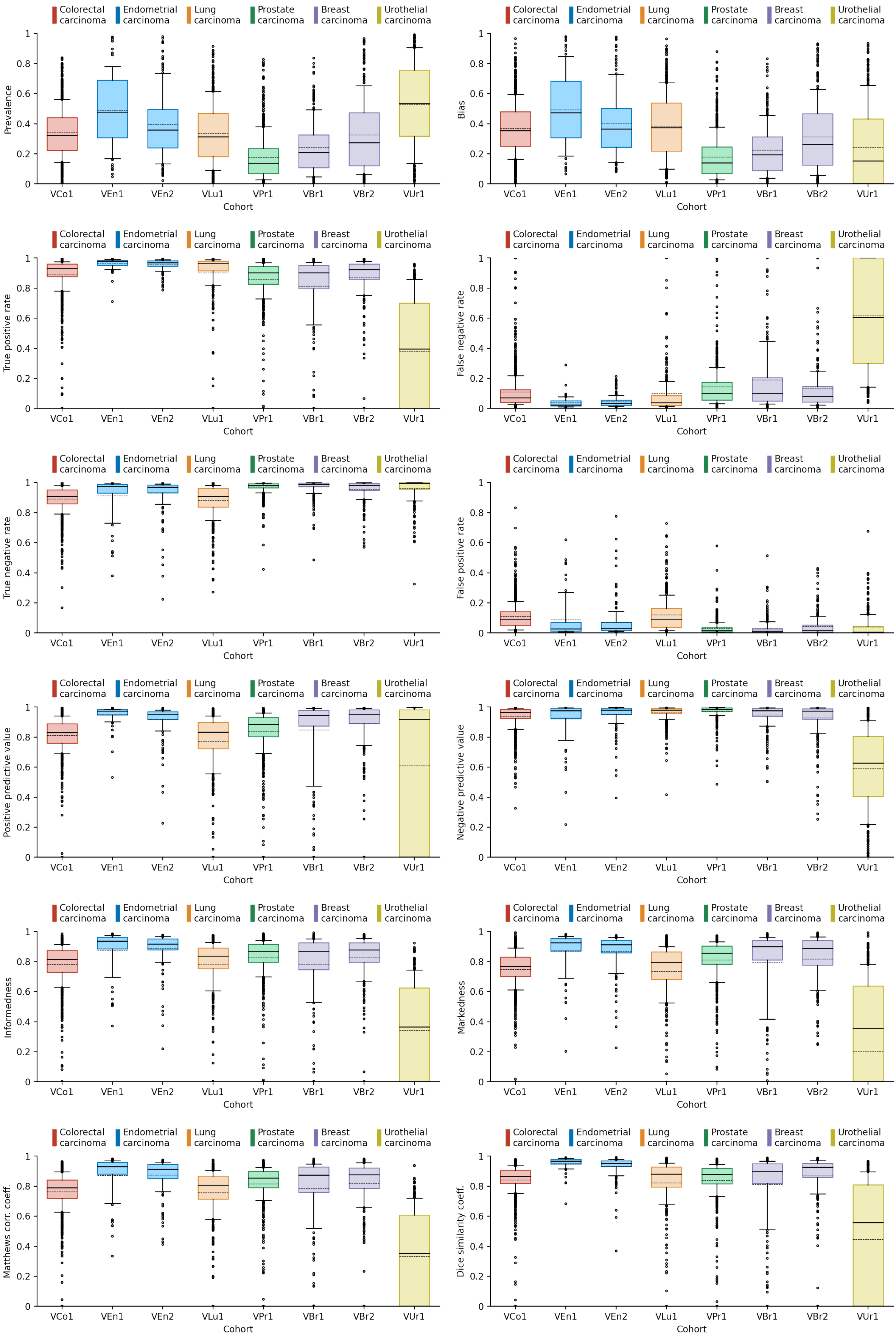

Figure S2: Primary model performance on NanoZoomer XR scans

For each cohort, the plot displays the interquartile range (coloured box), mean value (perforated horizontal line), median value (solid horizontal line), the 10th and 90th percentile (whiskers), and outliers (black circles).

1.3 Cancer type-specialised models performance (study protocol section 3.2.3)

1.3.1 Colorectal carcinoma

Table S3: Colorectal model performance on Aperio AT2 scans
Data entries show mean value (95% CI)

Cohort	Prevalence	Bias
VCo1	0.3410 (0.3315 – 0.3505)	0.3691 (0.3585 – 0.3796)
VEn1	0.4924 (0.4363 – 0.5485)	0.4511 (0.3949 – 0.5073)
VEn2	0.3970 (0.3611 – 0.4328)	0.4097 (0.3737 – 0.4457)
VLu1	0.3380 (0.3208 – 0.3552)	0.3908 (0.3666 – 0.4149)
VPr1	0.1777 (0.1671 – 0.1882)	0.5939 (0.5818 – 0.6060)
VBr1	0.2278 (0.2089 – 0.2467)	0.1952 (0.1752 – 0.2151)
VBr2	0.3081 (0.2808 – 0.3355)	0.2917 (0.2643 – 0.3191)
VUr1	0.5304 (0.5004 – 0.5603)	0.0408 (0.0260 – 0.0557)
**Cohort**	**True positive rate**	**False negative rate**
VCo1	0.8856 (0.8755 – 0.8958)	0.1144 (0.1042 – 0.1245)
VEn1	0.8684 (0.8206 – 0.9162)	0.1316 (0.0838 – 0.1794)
VEn2	0.8841 (0.8529 – 0.9153)	0.1159 (0.0847 – 0.1471)
VLu1	0.7058 (0.6734 – 0.7381)	0.2942 (0.2619 – 0.3266)
VPr1	0.9179 (0.9069 – 0.9290)	0.0821 (0.0710 – 0.0931)
VBr1	0.6776 (0.6362 – 0.7191)	0.3224 (0.2809 – 0.3638)
VBr2	0.7946 (0.7607 – 0.8285)	0.2054 (0.1715 – 0.2393)
VUr1	0.0551 (0.0368 – 0.0735)	0.9449 (0.9265 – 0.9632)
**Cohort**	**True negative rate**	**False positive rate**
VCo1	0.8932 (0.8876 – 0.8988)	0.1068 (0.1012 – 0.1124)
VEn1	0.9135 (0.8817 – 0.9454)	0.0865 (0.0546 – 0.1183)
VEn2	0.9070 (0.8897 – 0.9243)	0.0930 (0.0757 – 0.1103)
VLu1	0.7958 (0.7781 – 0.8134)	0.2042 (0.1866 – 0.2219)
VPr1	0.4906 (0.4759 – 0.5053)	0.5094 (0.4947 – 0.5241)
VBr1	0.9595 (0.9518 – 0.9672)	0.0405 (0.0328 – 0.0482)
VBr2	0.9312 (0.9189 – 0.9435)	0.0688 (0.0565 – 0.0811)
VUr1	0.9929 (0.9874 – 0.9983)	0.0071 (0.0017 – 0.0126)
**Cohort**	**Positive predictive value**	**Negative predictive value**
VCo1	0.8095 (0.8005 – 0.8186)	0.9440 (0.9397 – 0.9482)
VEn1	0.8940 (0.8482 – 0.9398)	0.8496 (0.8034 – 0.8957)
VEn2	0.8239 (0.7862 – 0.8616)	0.9215 (0.9023 – 0.9407)
VLu1	0.5768 (0.5491 – 0.6044)	0.9058 (0.8969 – 0.9147)
VPr1	0.2881 (0.2712 – 0.3050)	0.9696 (0.9651 – 0.9741)
VBr1	0.6811 (0.6393 – 0.7228)	0.9261 (0.9140 – 0.9382)
VBr2	0.7703 (0.7364 – 0.8041)	0.9141 (0.8966 – 0.9316)
VUr1	0.1184 (0.0843 – 0.1526)	0.4836 (0.4540 – 0.5132)
**Cohort**	**Informedness**	**Markedness**
VCo1	0.7788 (0.7688 – 0.7889)	0.7535 (0.7434 – 0.7636)
VEn1	0.7819 (0.7296 – 0.8343)	0.7435 (0.6723 – 0.8147)
VEn2	0.7911 (0.7590 – 0.8232)	0.7454 (0.7030 – 0.7878)
VLu1	0.5015 (0.4751 – 0.5279)	0.4825 (0.4525 – 0.5126)
VPr1	0.4086 (0.3929 – 0.4243)	0.2577 (0.2418 – 0.2736)
VBr1	0.6371 (0.5967 – 0.6775)	0.6072 (0.5592 – 0.6551)
VBr2	0.7258 (0.6931 – 0.7585)	0.6844 (0.6439 – 0.7249)
VUr1	0.0480 (0.0318 – 0.0642)	-0.3979 (-0.4420 – -0.3539)
**Cohort**	**Matthews corr. coeff.**	**Dice similarity coeff.**
VCo1	0.7659 (0.7566 – 0.7751)	0.8387 (0.8297 – 0.8477)
VEn1	0.7672 (0.7147 – 0.8197)	0.8630 (0.8153 – 0.9107)
VEn2	0.7674 (0.7335 – 0.8013)	0.8413 (0.8071 – 0.8755)
VLu1	0.5010 (0.4760 – 0.5260)	0.6150 (0.5865 – 0.6435)
VPr1	0.3094 (0.2944 – 0.3244)	0.3903 (0.3726 – 0.4080)
VBr1	0.6292 (0.5896 – 0.6688)	0.6543 (0.6139 – 0.6948)
VBr2	0.7092 (0.6770 – 0.7415)	0.7637 (0.7307 – 0.7967)
VUr1	0.0452 (0.0306 – 0.0598)	0.0705 (0.0484 – 0.0927)

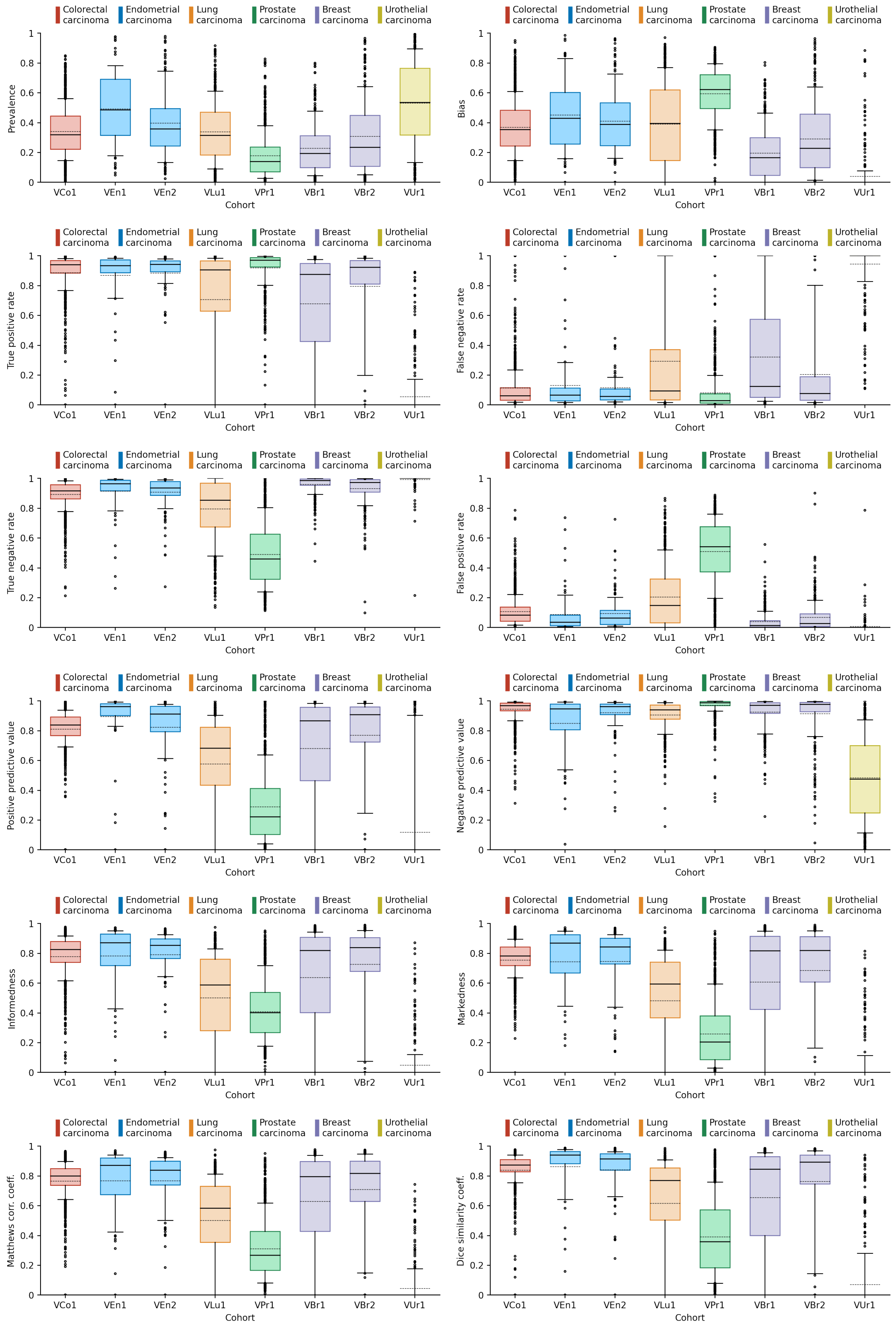

Figure S3: Colorectal model performance on Aperio AT2 scans

For each cohort, the plot displays the interquartile range (coloured box), mean value (perforated horizontal line), median value (solid horizontal line), the 10th and 90th percentile (whiskers), and outliers (black circles).

1.3.2 Endometrial carcinoma

Table S4: Endometrial model performance on Aperio AT2 scans
Data entries show mean value (95% CI)

Cohort	Prevalence	Bias
VCo1	0.3410 (0.3315 – 0.3505)	0.4541 (0.4431 – 0.4651)
VEn1	0.4924 (0.4363 – 0.5485)	0.5023 (0.4465 – 0.5582)
VEn2	0.3970 (0.3611 – 0.4328)	0.4146 (0.3788 – 0.4503)
VLu1	0.3380 (0.3208 – 0.3552)	0.6098 (0.5891 – 0.6305)
VPr1	0.1777 (0.1671 – 0.1882)	0.6396 (0.6279 – 0.6513)
VBr1	0.2278 (0.2089 – 0.2467)	0.2583 (0.2359 – 0.2806)
VBr2	0.3081 (0.2808 – 0.3355)	0.3690 (0.3395 – 0.3985)
VUr1	0.5304 (0.5004 – 0.5603)	0.3003 (0.2642 – 0.3364)
Cohort	True positive rate	False negative rate
VCo1	0.9342 (0.9261 – 0.9423)	0.0658 (0.0577 – 0.0739)
VEn1	0.9689 (0.9605 – 0.9773)	0.0311 (0.0227 – 0.0395)
VEn2	0.9676 (0.9631 – 0.9721)	0.0324 (0.0279 – 0.0369)
VLu1	0.9545 (0.9410 – 0.9681)	0.0455 (0.0319 – 0.0590)
VPr1	0.9703 (0.9639 – 0.9767)	0.0297 (0.0233 – 0.0361)
VBr1	0.8265 (0.7909 – 0.8620)	0.1735 (0.1380 – 0.2091)
VBr2	0.9221 (0.8991 – 0.9450)	0.0779 (0.0550 – 0.1009)
VUr1	0.4105 (0.3669 – 0.4541)	0.5895 (0.5459 – 0.6331)
Cohort	True negative rate	False positive rate
VCo1	0.7784 (0.7696 – 0.7871)	0.2216 (0.2129 – 0.2304)
VEn1	0.9015 (0.8666 – 0.9364)	0.0985 (0.0636 – 0.1334)
VEn2	0.9208 (0.9011 – 0.9405)	0.0792 (0.0595 – 0.0989)
VLu1	0.5569 (0.5352 – 0.5786)	0.4431 (0.4214 – 0.4648)
VPr1	0.4419 (0.4279 – 0.4560)	0.5581 (0.5440 – 0.5721)
VBr1	0.9080 (0.8941 – 0.9220)	0.0920 (0.0780 – 0.1059)
VBr2	0.8537 (0.8339 – 0.8735)	0.1463 (0.1265 – 0.1661)
VUr1	0.8594 (0.8371 – 0.8816)	0.1406 (0.1184 – 0.1629)
Cohort	Positive predictive value	Negative predictive value
VCo1	0.6883 (0.6786 – 0.6980)	0.9608 (0.9567 – 0.9650)
VEn1	0.9426 (0.9263 – 0.9589)	0.9250 (0.8912 – 0.9587)
VEn2	0.9109 (0.8949 – 0.9268)	0.9572 (0.9442 – 0.9702)
VLu1	0.5264 (0.5065 – 0.5463)	0.9761 (0.9731 – 0.9791)
VPr1	0.2769 (0.2612 – 0.2926)	0.9873 (0.9849 – 0.9897)
VBr1	0.6715 (0.6373 – 0.7057)	0.9586 (0.9474 – 0.9697)
VBr2	0.7220 (0.6938 – 0.7502)	0.9528 (0.9399 – 0.9656)
VUr1	0.4894 (0.4428 – 0.5360)	0.6085 (0.5789 – 0.6381)
Cohort	Informedness	Markedness
VCo1	0.7126 (0.7023 – 0.7228)	0.6491 (0.6387 – 0.6596)
VEn1	0.8704 (0.8351 – 0.9058)	0.8676 (0.8326 – 0.9026)
VEn2	0.8884 (0.8688 – 0.9079)	0.8680 (0.8496 – 0.8865)
VLu1	0.5114 (0.4897 – 0.5332)	0.5025 (0.4830 – 0.5220)
VPr1	0.4122 (0.3985 – 0.4259)	0.2642 (0.2493 – 0.2792)
VBr1	0.7345 (0.6997 – 0.7693)	0.6301 (0.5903 – 0.6699)
VBr2	0.7758 (0.7488 – 0.8027)	0.6748 (0.6439 – 0.7056)
VUr1	0.2699 (0.2392 – 0.3006)	0.0979 (0.0421 – 0.1537)
Cohort	Matthews corr. coeff.	Dice similarity coeff.
VCo1	0.6762 (0.6670 – 0.6854)	0.7800 (0.7712 – 0.7888)
VEn1	0.8661 (0.8314 – 0.9008)	0.9534 (0.9432 – 0.9637)
VEn2	0.8754 (0.8575 – 0.8934)	0.9345 (0.9235 – 0.9454)
VLu1	0.4915 (0.4729 – 0.5100)	0.6513 (0.6320 – 0.6706)
VPr1	0.3116 (0.2980 – 0.3251)	0.3884 (0.3712 – 0.4055)
VBr1	0.6826 (0.6492 – 0.7161)	0.7160 (0.6818 – 0.7502)
VBr2	0.7201 (0.6941 – 0.7462)	0.7917 (0.7661 – 0.8172)
VUr1	0.2651 (0.2356 – 0.2946)	0.4216 (0.3791 – 0.4641)

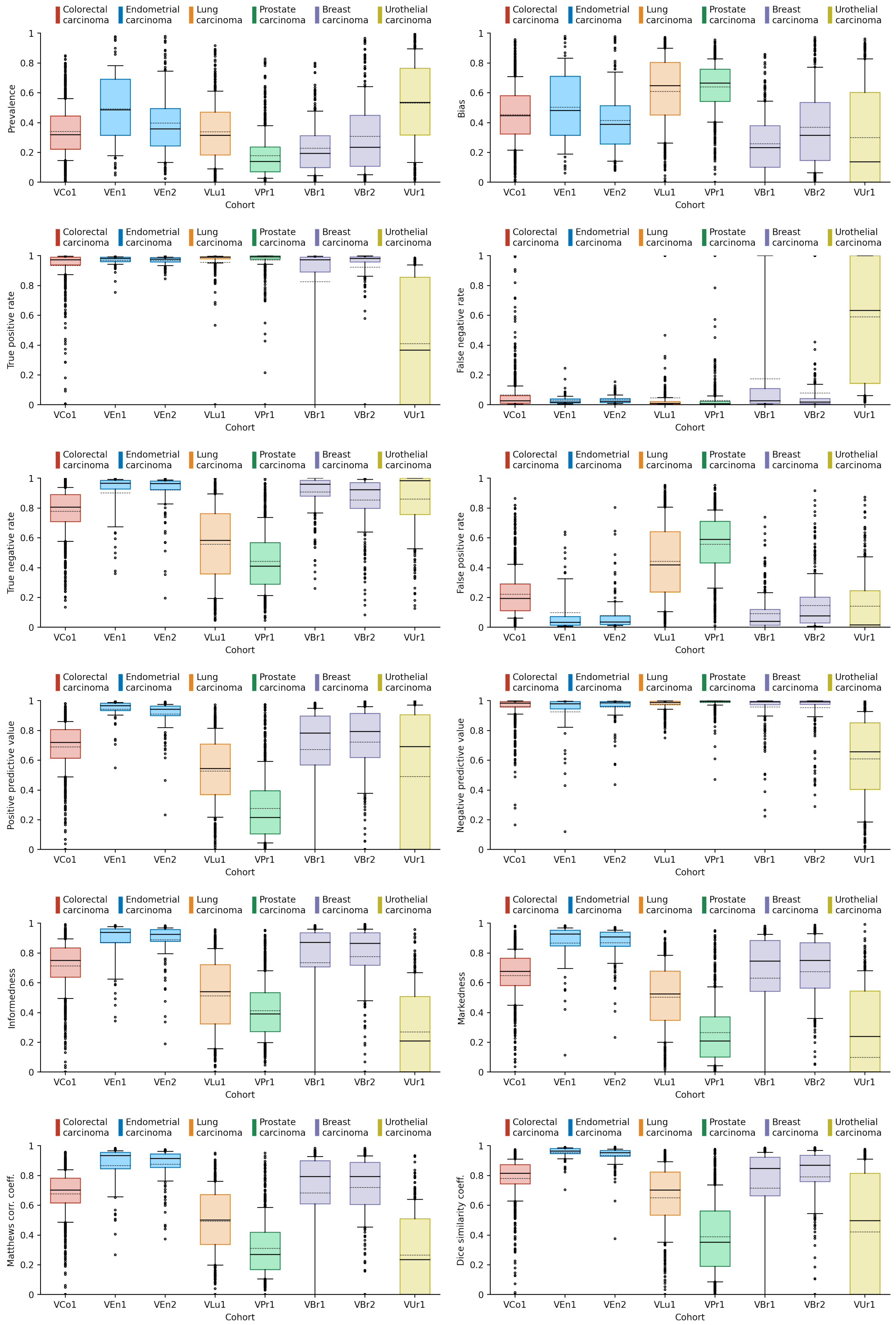

Figure S4: Endometrial model performance on Aperio AT2 scans

For each cohort, the plot displays the interquartile range (coloured box), mean value (perforated horizontal line), median value (solid horizontal line), the 10th and 90th percentile (whiskers), and outliers (black circles).

1.3.3 Lung carcinoma

Table S5: Lung model performance on Aperio AT2 scans
Data entries show mean value (95% CI)

Cohort	Prevalence	Bias
VCo1	0.3410 (0.3315 – 0.3505)	0.3841 (0.3742 – 0.3940)
VEn1	0.4924 (0.4363 – 0.5485)	0.4945 (0.4405 – 0.5484)
VEn2	0.3970 (0.3611 – 0.4328)	0.4451 (0.4110 – 0.4792)
VLu1	0.3380 (0.3208 – 0.3552)	0.3904 (0.3718 – 0.4091)
VPr1	0.1777 (0.1671 – 0.1882)	0.2360 (0.2226 – 0.2494)
VBr1	0.2278 (0.2089 – 0.2467)	0.1989 (0.1796 – 0.2183)
VBr2	0.3081 (0.2808 – 0.3355)	0.2808 (0.2544 – 0.3071)
VUr1	0.5304 (0.5004 – 0.5603)	0.3643 (0.3339 – 0.3948)
**Cohort**	**True positive rate**	**False negative rate**
VCo1	0.8697 (0.8602 – 0.8792)	0.1303 (0.1208 – 0.1398)
VEn1	0.9470 (0.9290 – 0.9650)	0.0530 (0.0350 – 0.0710)
VEn2	0.9536 (0.9454 – 0.9618)	0.0464 (0.0382 – 0.0546)
VLu1	0.9171 (0.9017 – 0.9324)	0.0829 (0.0676 – 0.0983)
VPr1	0.6463 (0.6198 – 0.6729)	0.3537 (0.3271 – 0.3802)
VBr1	0.7130 (0.6793 – 0.7468)	0.2870 (0.2532 – 0.3207)
VBr2	0.8023 (0.7750 – 0.8296)	0.1977 (0.1704 – 0.2250)
VUr1	0.5804 (0.5442 – 0.6166)	0.4196 (0.3834 – 0.4558)
**Cohort**	**True negative rate**	**False positive rate**
VCo1	0.8591 (0.8527 – 0.8655)	0.1409 (0.1345 – 0.1473)
VEn1	0.9073 (0.8799 – 0.9348)	0.0927 (0.0652 – 0.1201)
VEn2	0.8807 (0.8565 – 0.9049)	0.1193 (0.0951 – 0.1435)
VLu1	0.8758 (0.8661 – 0.8856)	0.1242 (0.1144 – 0.1339)
VPr1	0.8675 (0.8571 – 0.8779)	0.1325 (0.1221 – 0.1429)
VBr1	0.9664 (0.9569 – 0.9759)	0.0336 (0.0241 – 0.0431)
VBr2	0.9558 (0.9453 – 0.9663)	0.0442 (0.0337 – 0.0547)
VUr1	0.9320 (0.9219 – 0.9420)	0.0680 (0.0580 – 0.0781)
**Cohort**	**Positive predictive value**	**Negative predictive value**
VCo1	0.7603 (0.7509 – 0.7697)	0.9254 (0.9200 – 0.9309)
VEn1	0.9246 (0.8976 – 0.9516)	0.9058 (0.8694 – 0.9421)
VEn2	0.8478 (0.8175 – 0.8780)	0.9520 (0.9382 – 0.9657)
VLu1	0.7706 (0.7537 – 0.7875)	0.9666 (0.9623 – 0.9709)
VPr1	0.4593 (0.4351 – 0.4835)	0.9379 (0.9314 – 0.9445)
VBr1	0.7981 (0.7631 – 0.8332)	0.9311 (0.9208 – 0.9413)
VBr2	0.8640 (0.8364 – 0.8915)	0.9114 (0.8958 – 0.9271)
VUr1	0.7497 (0.7092 – 0.7903)	0.6697 (0.6426 – 0.6968)
**Cohort**	**Informedness**	**Markedness**
VCo1	0.7288 (0.7185 – 0.7391)	0.6857 (0.6757 – 0.6956)
VEn1	0.8544 (0.8212 – 0.8875)	0.8304 (0.7886 – 0.8721)
VEn2	0.8343 (0.8102 – 0.8584)	0.7997 (0.7697 – 0.8298)
VLu1	0.7929 (0.7770 – 0.8088)	0.7372 (0.7196 – 0.7547)
VPr1	0.5139 (0.4901 – 0.5376)	0.3972 (0.3724 – 0.4221)
VBr1	0.6794 (0.6459 – 0.7130)	0.7292 (0.6913 – 0.7672)
VBr2	0.7581 (0.7305 – 0.7857)	0.7754 (0.7425 – 0.8082)
VUr1	0.5124 (0.4800 – 0.5447)	0.4194 (0.3714 – 0.4674)
**Cohort**	**Matthews corr. coeff.**	**Dice similarity coeff.**
VCo1	0.7048 (0.6955 – 0.7141)	0.7972 (0.7885 – 0.8058)
VEn1	0.8386 (0.8013 – 0.8759)	0.9287 (0.9061 – 0.9512)
VEn2	0.8126 (0.7858 – 0.8394)	0.8809 (0.8560 – 0.9058)
VLu1	0.7620 (0.7466 – 0.7774)	0.8278 (0.8120 – 0.8436)
VPr1	0.4483 (0.4260 – 0.4706)	0.4906 (0.4673 – 0.5139)
VBr1	0.7021 (0.6695 – 0.7348)	0.7281 (0.6949 – 0.7614)
VBr2	0.7645 (0.7373 – 0.7917)	0.8126 (0.7853 – 0.8400)
VUr1	0.4869 (0.4555 – 0.5182)	0.6380 (0.6009 – 0.6752)

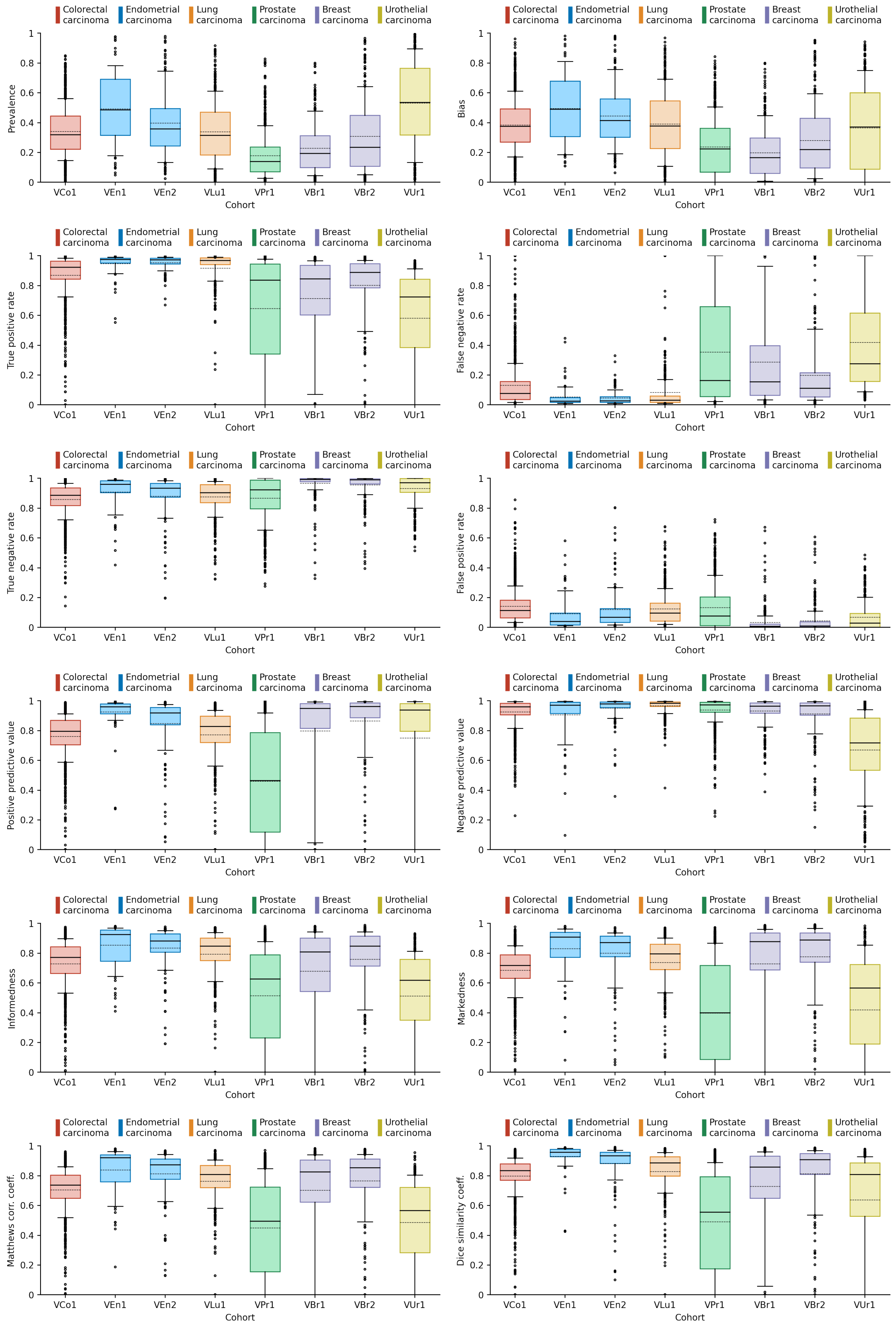

Figure S5: Lung model performance on Aperio AT2 scans

For each cohort, the plot displays the interquartile range (coloured box), mean value (perforated horizontal line), median value (solid horizontal line), the 10th and 90th percentile (whiskers), and outliers (black circles).

1.3.4 Prostate carcinoma

Table S6: Prostate model performance on Aperio AT2 scans
Data entries show mean value (95% CI)

Cohort	Prevalence	Bias
VCo1	0.3410 (0.3315 – 0.3505)	0.3655 (0.3537 – 0.3773)
VEn1	0.4924 (0.4363 – 0.5485)	0.5333 (0.4816 – 0.5850)
VEn2	0.3970 (0.3611 – 0.4328)	0.4953 (0.4540 – 0.5366)
VLu1	0.3380 (0.3208 – 0.3552)	0.4121 (0.3875 – 0.4366)
VPr1	0.1777 (0.1671 – 0.1882)	0.1732 (0.1627 – 0.1838)
VBr1	0.2278 (0.2089 – 0.2467)	0.2004 (0.1816 – 0.2191)
VBr2	0.3081 (0.2808 – 0.3355)	0.2837 (0.2573 – 0.3101)
VUr1	0.5304 (0.5004 – 0.5603)	0.0809 (0.0623 – 0.0995)
Cohort	True positive rate	False negative rate
VCo1	0.7686 (0.7506 – 0.7866)	0.2314 (0.2134 – 0.2494)
VEn1	0.8834 (0.8402 – 0.9266)	0.1166 (0.0734 – 0.1598)
VEn2	0.8232 (0.7767 – 0.8697)	0.1768 (0.1303 – 0.2233)
VLu1	0.6955 (0.6646 – 0.7264)	0.3045 (0.2736 – 0.3354)
VPr1	0.8434 (0.8336 – 0.8531)	0.1566 (0.1469 – 0.1664)
VBr1	0.7669 (0.7326 – 0.8012)	0.2331 (0.1988 – 0.2674)
VBr2	0.8401 (0.8133 – 0.8669)	0.1599 (0.1331 – 0.1867)
VUr1	0.1288 (0.1031 – 0.1546)	0.8712 (0.8454 – 0.8969)
Cohort	True negative rate	False positive rate
VCo1	0.8313 (0.8238 – 0.8388)	0.1687 (0.1612 – 0.1762)
VEn1	0.8011 (0.7482 – 0.8539)	0.1989 (0.1461 – 0.2518)
VEn2	0.7338 (0.6966 – 0.7711)	0.2662 (0.2289 – 0.3034)
VLu1	0.7514 (0.7313 – 0.7715)	0.2486 (0.2285 – 0.2687)
VPr1	0.9753 (0.9725 – 0.9781)	0.0247 (0.0219 – 0.0275)
VBr1	0.9701 (0.9649 – 0.9754)	0.0299 (0.0246 – 0.0351)
VBr2	0.9523 (0.9427 – 0.9619)	0.0477 (0.0381 – 0.0573)
VUr1	0.9871 (0.9817 – 0.9925)	0.0129 (0.0075 – 0.0183)
Cohort	Positive predictive value	Negative predictive value
VCo1	0.6430 (0.6287 – 0.6573)	0.8909 (0.8824 – 0.8994)
VEn1	0.8126 (0.7535 – 0.8716)	0.8578 (0.8086 – 0.9070)
VEn2	0.6367 (0.5877 – 0.6857)	0.8885 (0.8635 – 0.9135)
VLu1	0.5504 (0.5241 – 0.5767)	0.8887 (0.8782 – 0.8993)
VPr1	0.8717 (0.8626 – 0.8808)	0.9703 (0.9677 – 0.9730)
VBr1	0.7956 (0.7624 – 0.8288)	0.9407 (0.9293 – 0.9521)
VBr2	0.8525 (0.8274 – 0.8776)	0.9202 (0.9039 – 0.9365)
VUr1	0.3673 (0.3164 – 0.4182)	0.5071 (0.4771 – 0.5371)
Cohort	Informedness	Markedness
VCo1	0.5999 (0.5843 – 0.6156)	0.5339 (0.5156 – 0.5522)
VEn1	0.6845 (0.6257 – 0.7433)	0.6703 (0.6063 – 0.7343)
VEn2	0.5571 (0.5110 – 0.6031)	0.5252 (0.4696 – 0.5809)
VLu1	0.4469 (0.4219 – 0.4720)	0.4391 (0.4098 – 0.4684)
VPr1	0.8187 (0.8091 – 0.8283)	0.8420 (0.8330 – 0.8510)
VBr1	0.7370 (0.7036 – 0.7705)	0.7363 (0.6975 – 0.7752)
VBr2	0.7924 (0.7658 – 0.8191)	0.7727 (0.7399 – 0.8055)
VUr1	0.1159 (0.0931 – 0.1387)	-0.1256 (-0.1844 – -0.0668)
Cohort	Matthews corr. coeff.	Dice similarity coeff.
VCo1	0.5739 (0.5594 – 0.5884)	0.6767 (0.6615 – 0.6920)
VEn1	0.6719 (0.6112 – 0.7327)	0.8084 (0.7570 – 0.8599)
VEn2	0.5454 (0.5001 – 0.5907)	0.6864 (0.6405 – 0.7323)
VLu1	0.4475 (0.4237 – 0.4713)	0.5794 (0.5527 – 0.6060)
VPr1	0.8273 (0.8188 – 0.8357)	0.8498 (0.8412 – 0.8585)
VBr1	0.7381 (0.7060 – 0.7702)	0.7589 (0.7259 – 0.7919)
VBr2	0.7828 (0.7571 – 0.8086)	0.8306 (0.8049 – 0.8563)
VUr1	0.1307 (0.1073 – 0.1540)	0.1665 (0.1365 – 0.1965)

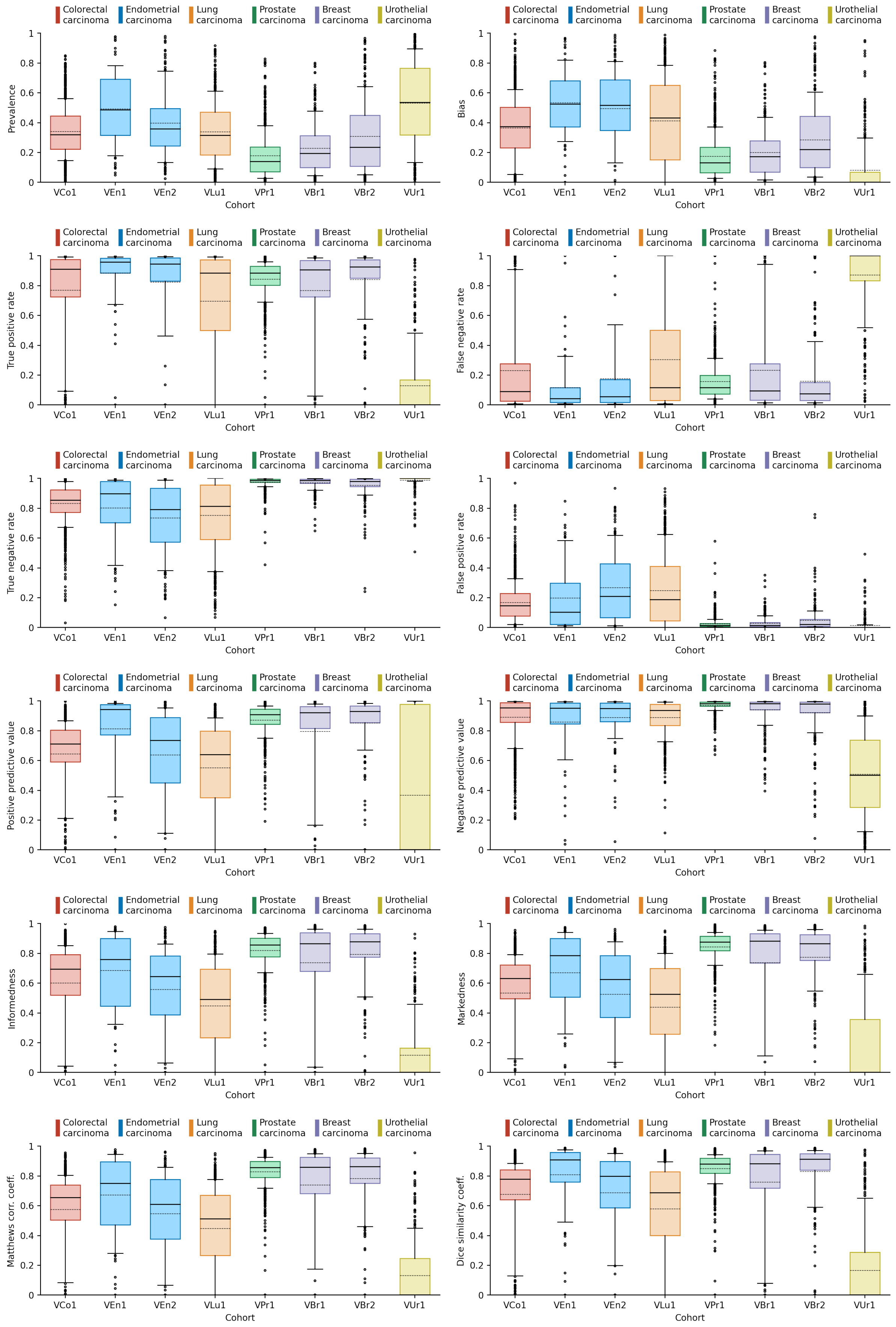

Figure S6: Prostate model performance on Aperio AT2 scans
For each cohort, the plot displays the interquartile range (coloured box), mean value (perforated horizontal line), median value (solid horizontal line), the 10th and 90th percentile (whiskers), and outliers (black circles).

1.4 Primary model replication performance (study protocol section 3.2.4)

1.4.1 Replication 1

Table S7: First primary model replication performance on Aperio AT2 scans
Data entries show mean value (95% CI)

Cohort	Prevalence	Bias
VCo1	0.3410 (0.3315 – 0.3505)	0.3746 (0.3647 – 0.3845)
VEn1	0.4924 (0.4363 – 0.5485)	0.4993 (0.4432 – 0.5553)
VEn2	0.3970 (0.3611 – 0.4328)	0.4141 (0.3782 – 0.4500)
VLu1	0.3380 (0.3208 – 0.3552)	0.3967 (0.3777 – 0.4156)
VPr1	0.1777 (0.1671 – 0.1882)	0.1820 (0.1713 – 0.1926)
VBr1	0.2278 (0.2089 – 0.2467)	0.2139 (0.1958 – 0.2320)
VBr2	0.3081 (0.2808 – 0.3355)	0.2974 (0.2709 – 0.3240)
VUr1	0.5304 (0.5004 – 0.5603)	0.2726 (0.2416 – 0.3036)
**Cohort**	**True positive rate**	**False negative rate**
VCo1	0.9015 (0.8942 – 0.9088)	0.0985 (0.0912 – 0.1058)
VEn1	0.9662 (0.9579 – 0.9745)	0.0338 (0.0255 – 0.0421)
VEn2	0.9647 (0.9595 – 0.9698)	0.0353 (0.0302 – 0.0405)
VLu1	0.9062 (0.8892 – 0.9232)	0.0938 (0.0768 – 0.1108)
VPr1	0.8673 (0.8564 – 0.8781)	0.1327 (0.1219 – 0.1436)
VBr1	0.8344 (0.8091 – 0.8597)	0.1656 (0.1403 – 0.1909)
VBr2	0.8892 (0.8721 – 0.9064)	0.1108 (0.0936 – 0.1279)
VUr1	0.4238 (0.3845 – 0.4630)	0.5762 (0.5370 – 0.6155)
**Cohort**	**True negative rate**	**False positive rate**
VCo1	0.8872 (0.8819 – 0.8924)	0.1128 (0.1076 – 0.1181)
VEn1	0.9102 (0.8782 – 0.9422)	0.0898 (0.0578 – 0.1218)
VEn2	0.9201 (0.9010 – 0.9391)	0.0799 (0.0609 – 0.0990)
VLu1	0.8645 (0.8538 – 0.8752)	0.1355 (0.1248 – 0.1462)
VPr1	0.9686 (0.9656 – 0.9716)	0.0314 (0.0284 – 0.0344)
VBr1	0.9715 (0.9658 – 0.9772)	0.0285 (0.0228 – 0.0342)
VBr2	0.9587 (0.9510 – 0.9665)	0.0413 (0.0335 – 0.0490)
VUr1	0.9402 (0.9285 – 0.9520)	0.0598 (0.0480 – 0.0715)
**Cohort**	**Positive predictive value**	**Negative predictive value**
VCo1	0.8061 (0.7986 – 0.8135)	0.9413 (0.9369 – 0.9456)
VEn1	0.9474 (0.9318 – 0.9630)	0.9265 (0.8960 – 0.9569)
VEn2	0.9112 (0.8952 – 0.9272)	0.9573 (0.9446 – 0.9701)
VLu1	0.7552 (0.7372 – 0.7733)	0.9661 (0.9620 – 0.9701)
VPr1	0.8354 (0.8246 – 0.8463)	0.9744 (0.9716 – 0.9772)
VBr1	0.8471 (0.8206 – 0.8736)	0.9532 (0.9445 – 0.9619)
VBr2	0.8896 (0.8709 – 0.9082)	0.9352 (0.9223 – 0.9481)
VUr1	0.6053 (0.5570 – 0.6536)	0.6065 (0.5779 – 0.6351)
**Cohort**	**Informedness**	**Markedness**
VCo1	0.7887 (0.7805 – 0.7968)	0.7474 (0.7390 – 0.7557)
VEn1	0.8765 (0.8437 – 0.9092)	0.8739 (0.8414 – 0.9063)
VEn2	0.8848 (0.8658 – 0.9037)	0.8685 (0.8501 – 0.8869)
VLu1	0.7707 (0.7533 – 0.7881)	0.7213 (0.7025 – 0.7401)
VPr1	0.8359 (0.8252 – 0.8466)	0.8099 (0.7991 – 0.8207)
VBr1	0.8059 (0.7807 – 0.8312)	0.8003 (0.7706 – 0.8299)
VBr2	0.8480 (0.8303 – 0.8657)	0.8248 (0.8011 – 0.8485)
VUr1	0.3640 (0.3302 – 0.3978)	0.2117 (0.1538 – 0.2697)
**Cohort**	**Matthews corr. coeff.**	**Dice similarity coeff.**
VCo1	0.7670 (0.7595 – 0.7746)	0.8453 (0.8387 – 0.8519)
VEn1	0.8735 (0.8416 – 0.9055)	0.9552 (0.9444 – 0.9660)
VEn2	0.8740 (0.8563 – 0.8917)	0.9332 (0.9221 – 0.9443)
VLu1	0.7432 (0.7264 – 0.7601)	0.8137 (0.7963 – 0.8310)
VPr1	0.8195 (0.8095 – 0.8296)	0.8429 (0.8326 – 0.8532)
VBr1	0.7994 (0.7745 – 0.8243)	0.8213 (0.7956 – 0.8470)
VBr2	0.8361 (0.8183 – 0.8538)	0.8814 (0.8645 – 0.8984)
VUr1	0.3520 (0.3200 – 0.3840)	0.4766 (0.4352 – 0.5179)

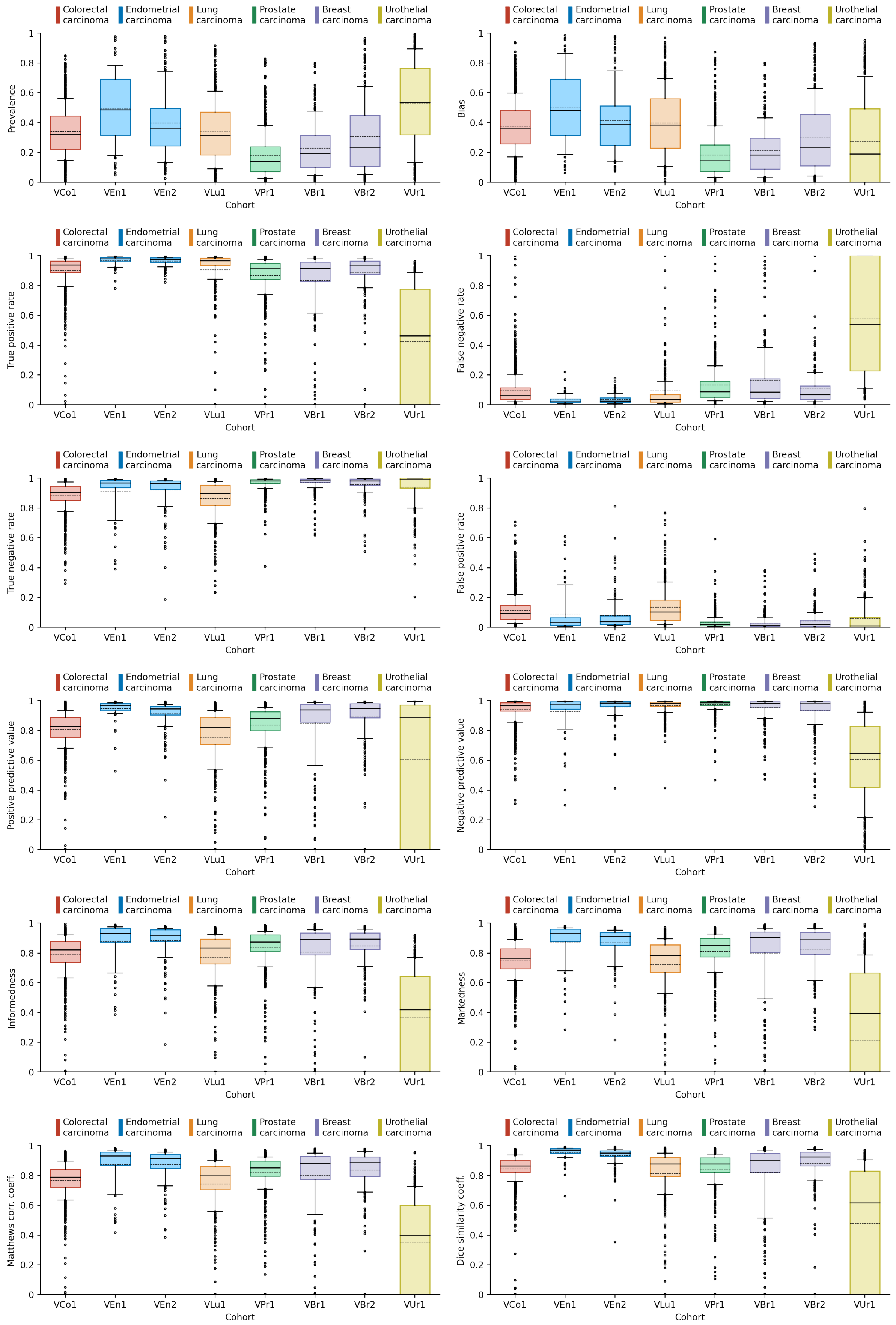

Figure S7: First primary model replication performance on Aperio AT2 scans
For each cohort, the plot displays the interquartile range (coloured box), mean value (perforated horizontal line), median value (solid horizontal line), the 10th and 90th percentile (whiskers), and outliers (black circles).

1.4.2 Replication 2

Table S8: Second primary model replication performance on Aperio AT2 scans
Data entries show mean value (95% CI)

Cohort	Prevalence	Bias
VCo1	0.3410 (0.3315 – 0.3505)	0.3744 (0.3644 – 0.3845)
VEn1	0.4924 (0.4363 – 0.5485)	0.5010 (0.4448 – 0.5573)
VEn2	0.3970 (0.3611 – 0.4328)	0.4162 (0.3800 – 0.4524)
VLu1	0.3380 (0.3208 – 0.3552)	0.4058 (0.3865 – 0.4251)
VPr1	0.1777 (0.1671 – 0.1882)	0.1803 (0.1695 – 0.1911)
VBr1	0.2278 (0.2089 – 0.2467)	0.2136 (0.1951 – 0.2320)
VBr2	0.3081 (0.2808 – 0.3355)	0.2977 (0.2708 – 0.3245)
VUr1	0.5304 (0.5004 – 0.5603)	0.2337 (0.2026 – 0.2648)
Cohort	True positive rate	False negative rate
VCo1	0.8970 (0.8886 – 0.9054)	0.1030 (0.0946 – 0.1114)
VEn1	0.9668 (0.9585 – 0.9750)	0.0332 (0.0250 – 0.0415)
VEn2	0.9665 (0.9616 – 0.9714)	0.0335 (0.0286 – 0.0384)
VLu1	0.9130 (0.8960 – 0.9301)	0.0870 (0.0699 – 0.1040)
VPr1	0.8536 (0.8414 – 0.8657)	0.1464 (0.1343 – 0.1586)
VBr1	0.8232 (0.7973 – 0.8491)	0.1768 (0.1509 – 0.2027)
VBr2	0.8825 (0.8651 – 0.9000)	0.1175 (0.1000 – 0.1349)
VUr1	0.3435 (0.3039 – 0.3832)	0.6565 (0.6168 – 0.6961)
Cohort	True negative rate	False positive rate
VCo1	0.8870 (0.8816 – 0.8924)	0.1130 (0.1076 – 0.1184)
VEn1	0.9028 (0.8675 – 0.9380)	0.0972 (0.0620 – 0.1325)
VEn2	0.9152 (0.8944 – 0.9360)	0.0848 (0.0640 – 0.1056)
VLu1	0.8518 (0.8402 – 0.8634)	0.1482 (0.1366 – 0.1598)
VPr1	0.9698 (0.9666 – 0.9730)	0.0302 (0.0270 – 0.0334)
VBr1	0.9719 (0.9660 – 0.9778)	0.0281 (0.0222 – 0.0340)
VBr2	0.9568 (0.9482 – 0.9654)	0.0432 (0.0346 – 0.0518)
VUr1	0.9428 (0.9307 – 0.9548)	0.0572 (0.0452 – 0.0693)
Cohort	Positive predictive value	Negative predictive value
VCo1	0.8022 (0.7940 – 0.8105)	0.9421 (0.9378 – 0.9464)
VEn1	0.9454 (0.9301 – 0.9607)	0.9269 (0.8959 – 0.9580)
VEn2	0.9097 (0.8936 – 0.9257)	0.9597 (0.9480 – 0.9714)
VLu1	0.7448 (0.7266 – 0.7630)	0.9700 (0.9660 – 0.9739)
VPr1	0.8402 (0.8285 – 0.8519)	0.9745 (0.9720 – 0.9771)
VBr1	0.8519 (0.8251 – 0.8788)	0.9538 (0.9458 – 0.9617)
VBr2	0.8970 (0.8791 – 0.9149)	0.9375 (0.9256 – 0.9494)
VUr1	0.5032 (0.4531 – 0.5534)	0.5826 (0.5540 – 0.6111)
Cohort	Informedness	Markedness
VCo1	0.7840 (0.7751 – 0.7930)	0.7444 (0.7351 – 0.7536)
VEn1	0.8695 (0.8338 – 0.9052)	0.8723 (0.8396 – 0.9051)
VEn2	0.8817 (0.8613 – 0.9021)	0.8694 (0.8514 – 0.8874)
VLu1	0.7648 (0.7472 – 0.7825)	0.7148 (0.6959 – 0.7337)
VPr1	0.8234 (0.8115 – 0.8352)	0.8148 (0.8032 – 0.8264)
VBr1	0.7951 (0.7694 – 0.8208)	0.8057 (0.7767 – 0.8347)
VBr2	0.8394 (0.8213 – 0.8574)	0.8345 (0.8125 – 0.8565)
VUr1	0.2863 (0.2529 – 0.3198)	0.0858 (0.0278 – 0.1438)
Cohort	Matthews corr. coeff.	Dice similarity coeff.
VCo1	0.7637 (0.7554 – 0.7720)	0.8411 (0.8335 – 0.8487)
VEn1	0.8692 (0.8355 – 0.9029)	0.9545 (0.9441 – 0.9649)
VEn2	0.8728 (0.8546 – 0.8911)	0.9333 (0.9221 – 0.9445)
VLu1	0.7366 (0.7197 – 0.7536)	0.8095 (0.7920 – 0.8269)
VPr1	0.8160 (0.8050 – 0.8270)	0.8388 (0.8275 – 0.8501)
VBr1	0.7969 (0.7715 – 0.8223)	0.8202 (0.7943 – 0.8461)
VBr2	0.8352 (0.8176 – 0.8528)	0.8804 (0.8635 – 0.8973)
VUr1	0.2751 (0.2439 – 0.3063)	0.3875 (0.3454 – 0.4295)

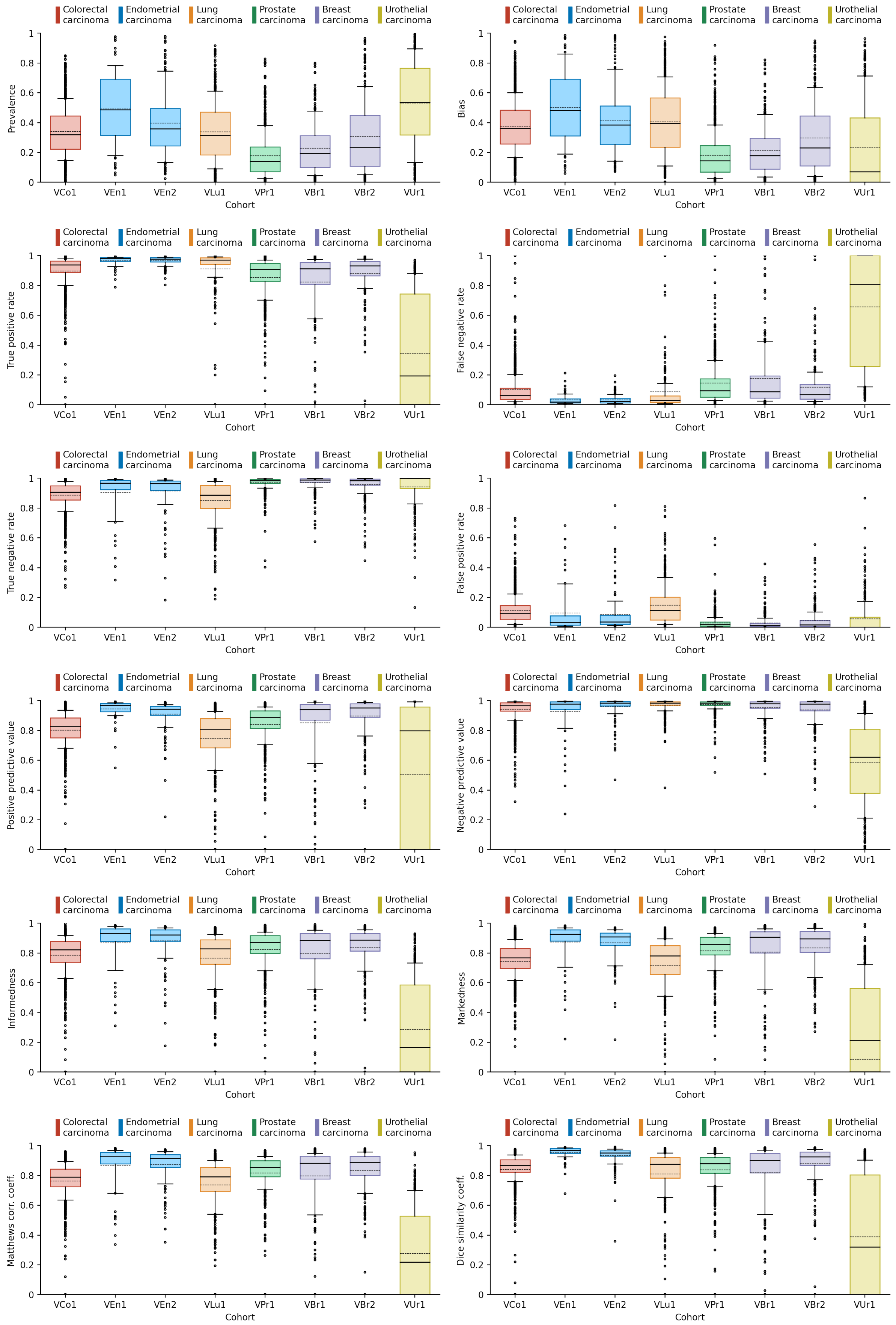

Figure S8: Second primary model replication performance on Aperio AT2 scans

For each cohort, the plot displays the interquartile range (coloured box), mean value (perforated horizontal line), median value (solid horizontal line), the 10th and 90th percentile (whiskers), and outliers (black circles).

2 Results from exploratory analyses

2.1 Association analyses in primary model

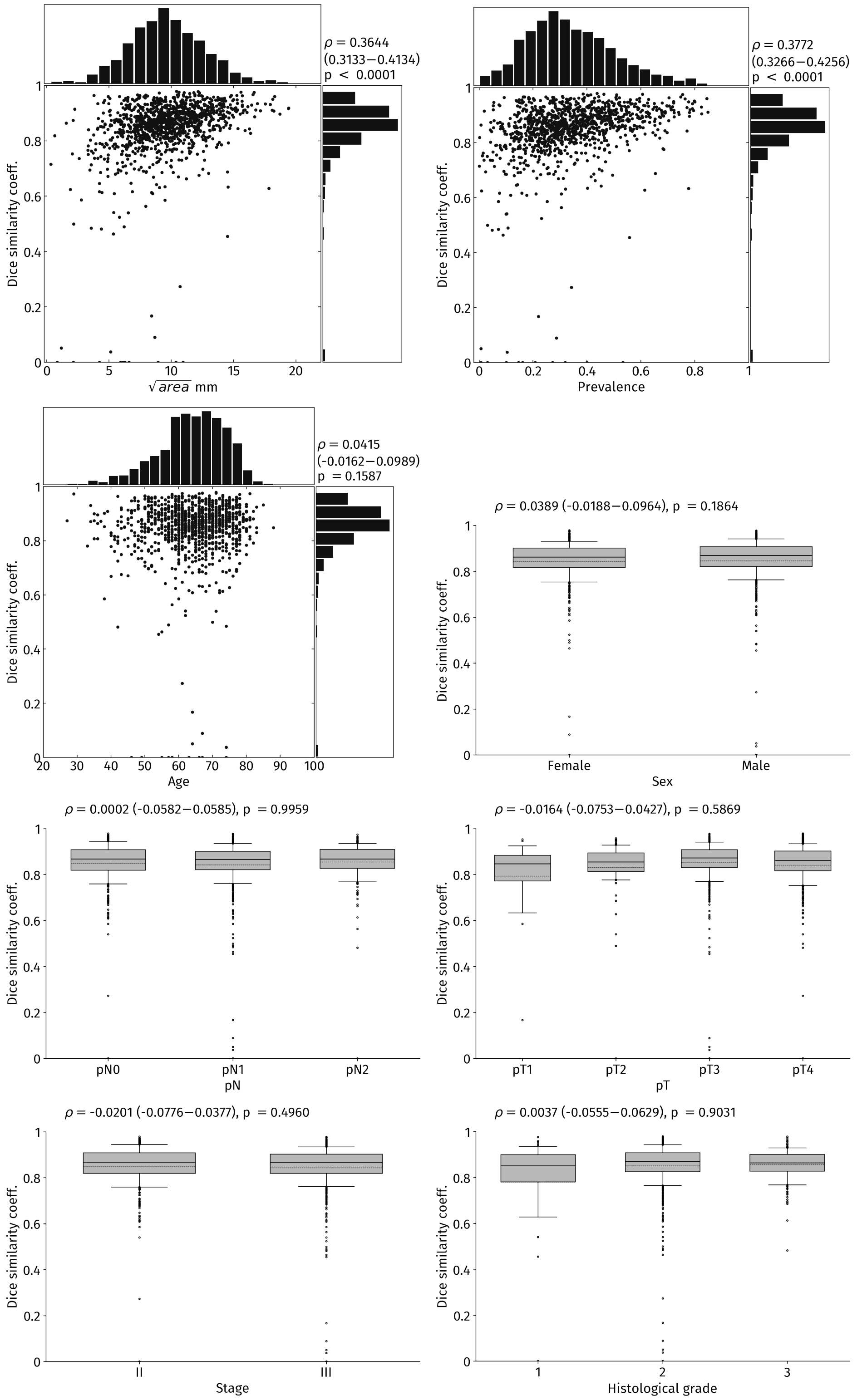

Figure S9: Associations of primary analysis result in VCo1

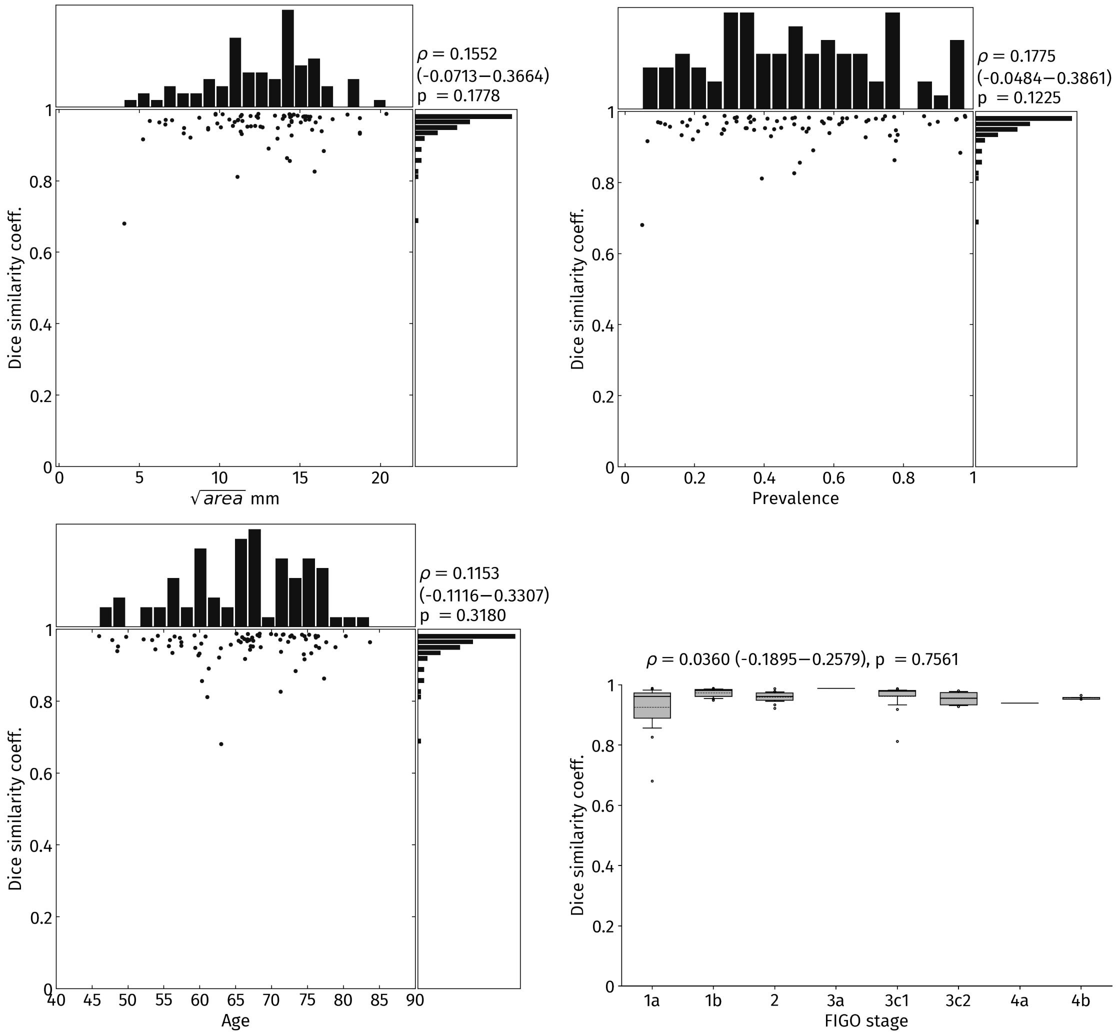

Figure S10: Associations of primary analysis result in VEn1

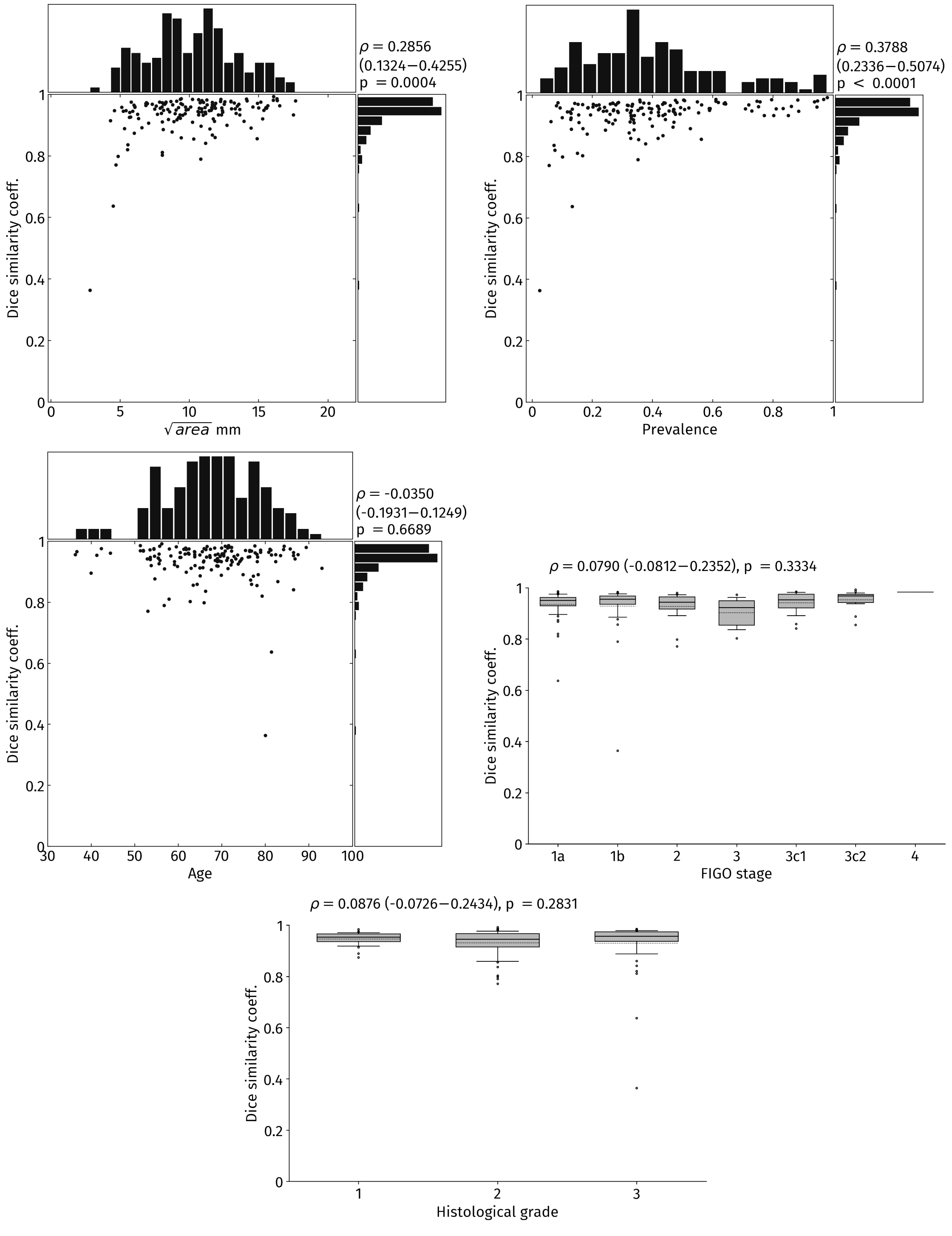

Figure S11: Associations of primary analysis result in VEn2

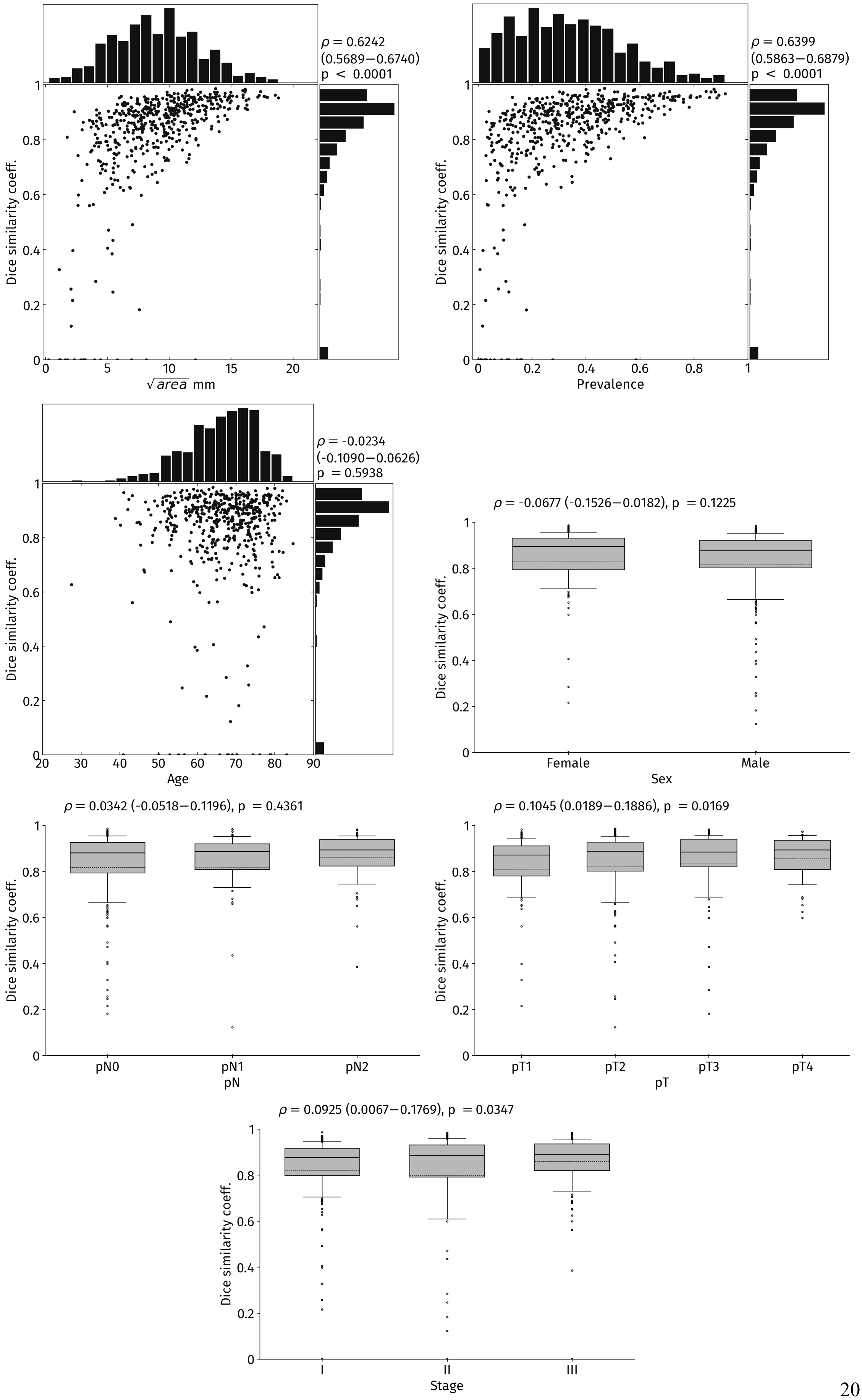

Figure S12: Associations of primary analysis result in VLu1

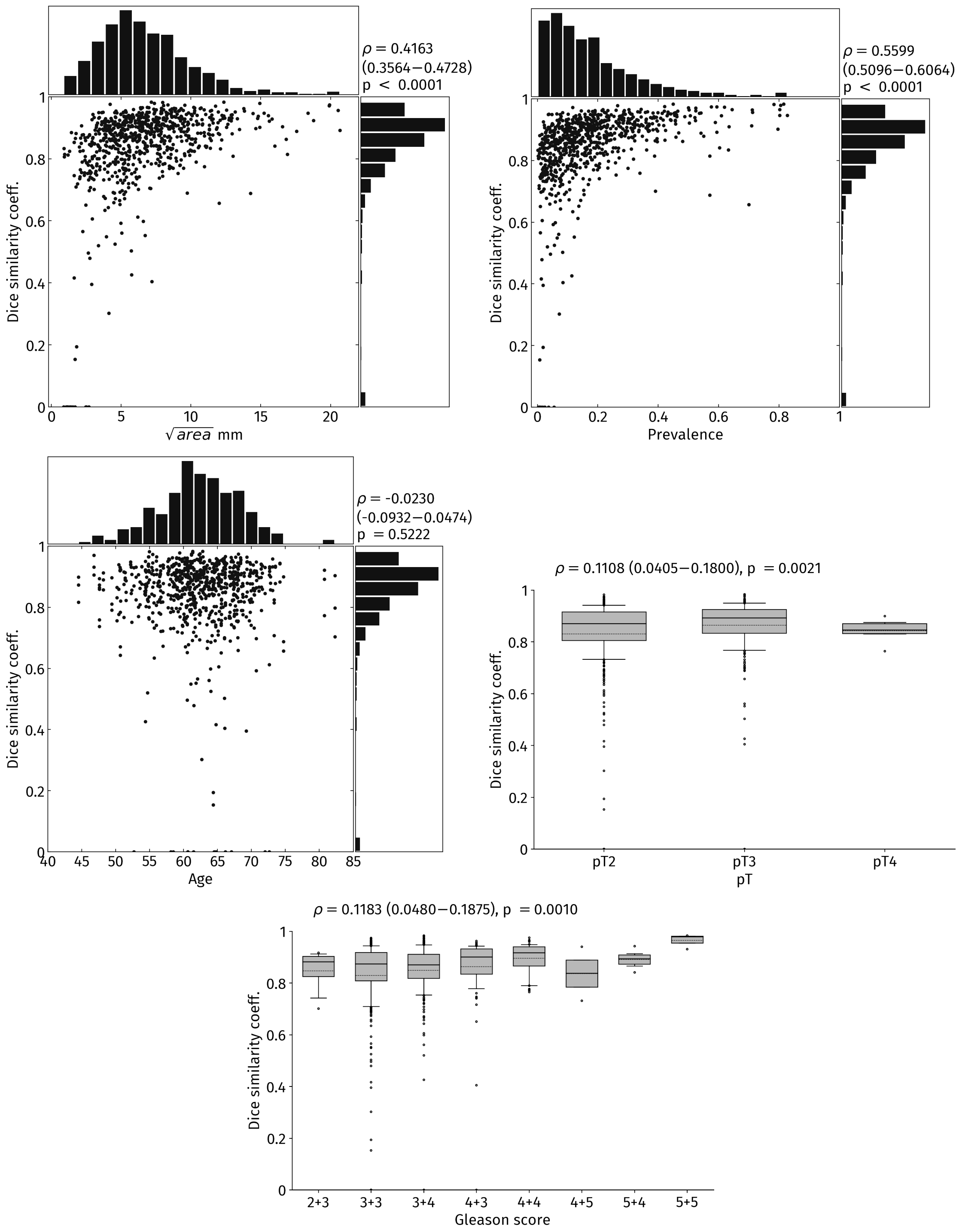

Figure S13: Associations of primary analysis result in VPr1

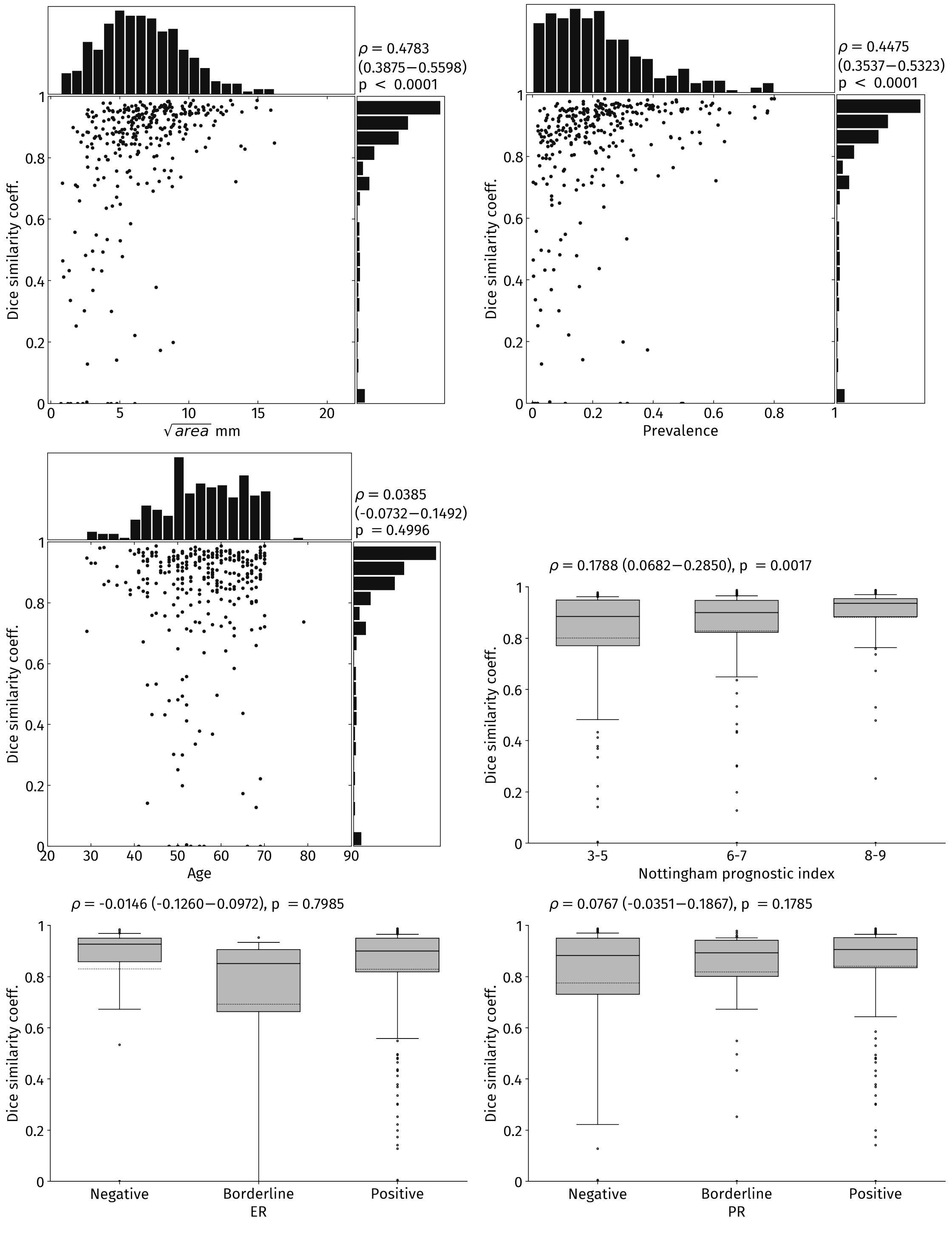

Figure S14: Associations of primary analysis result in VBr1

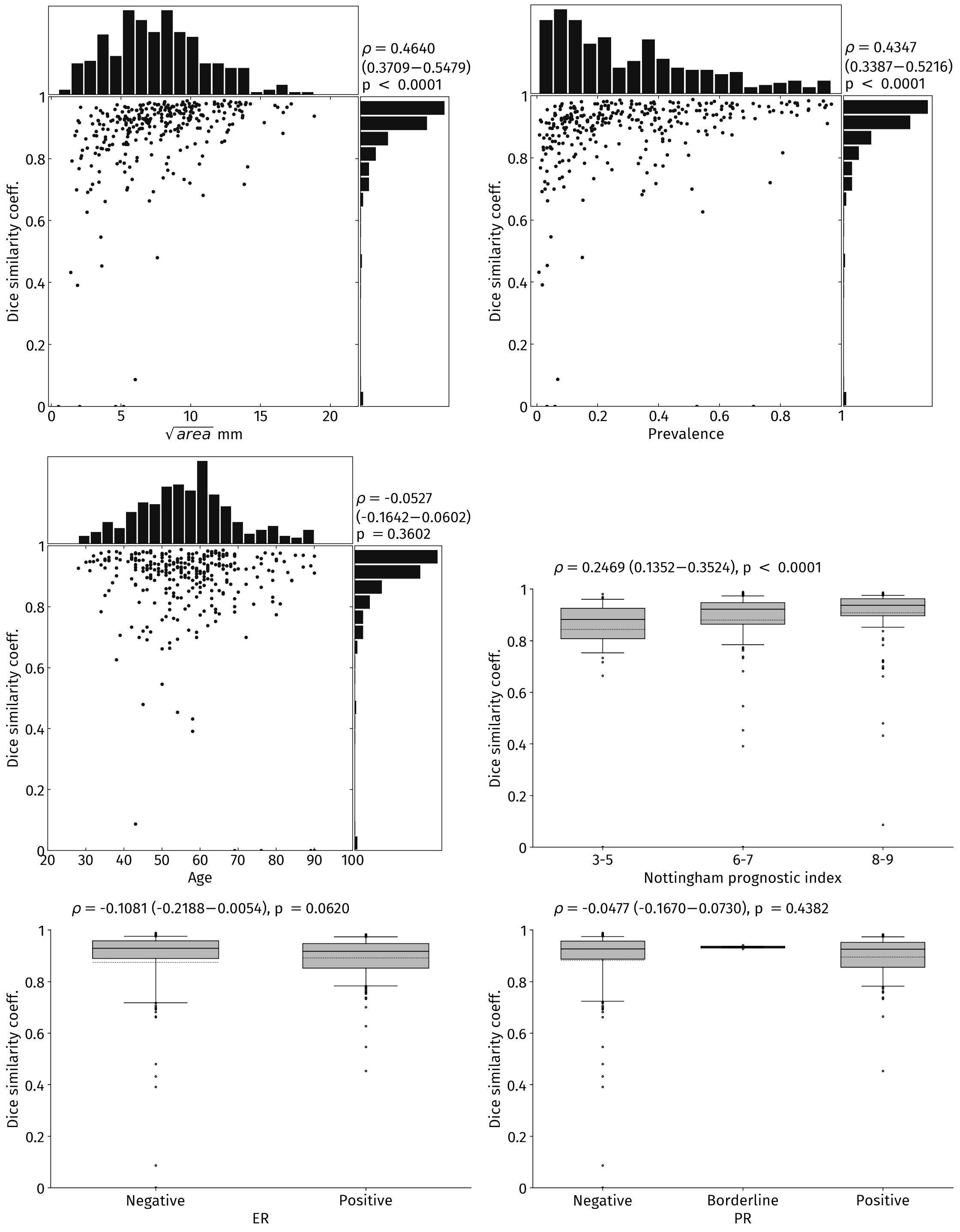

Figure S15: Associations of primary analysis result in VBr2

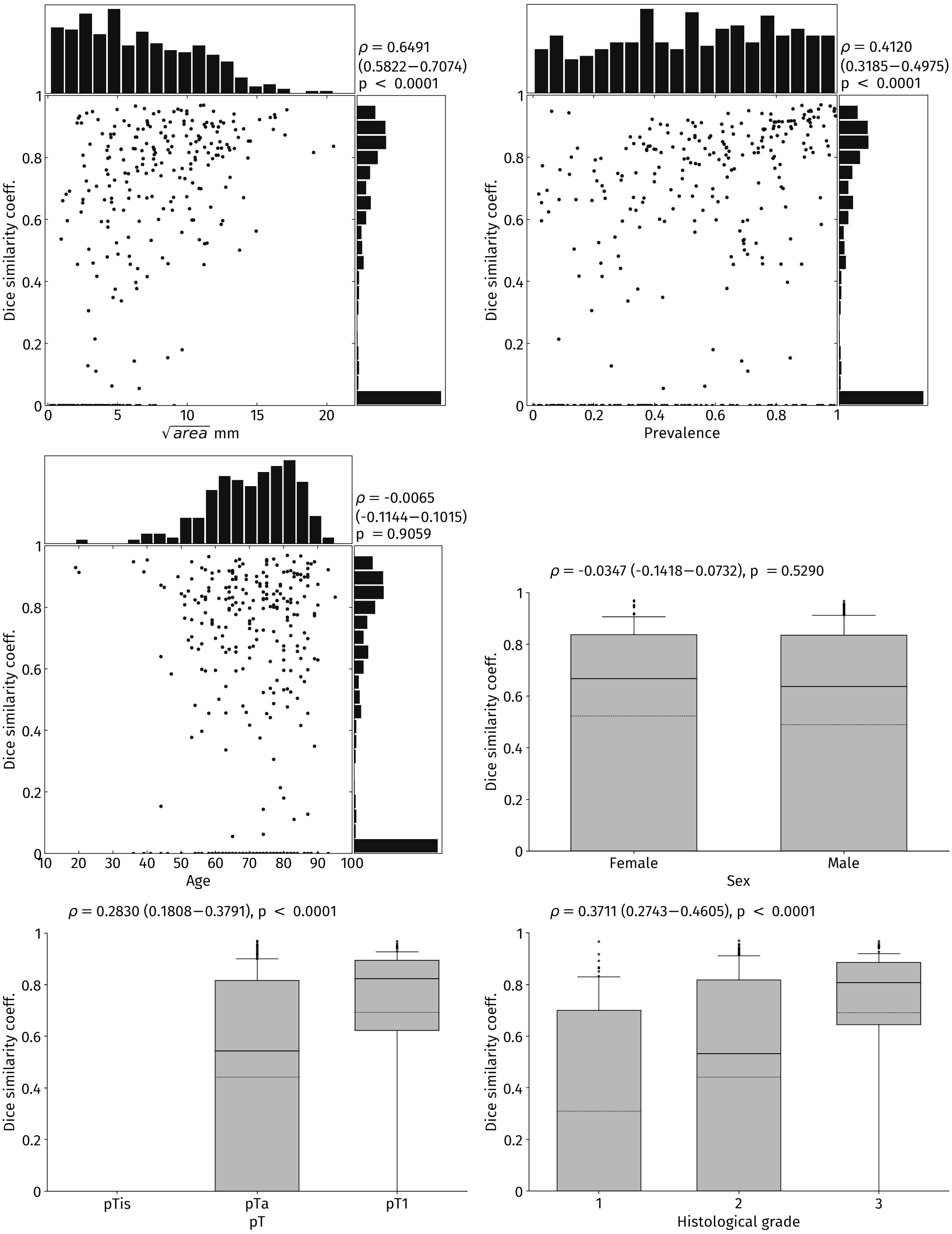

Figure S16: Associations of primary analysis result in VUr1

2.2 Per-scan comparison

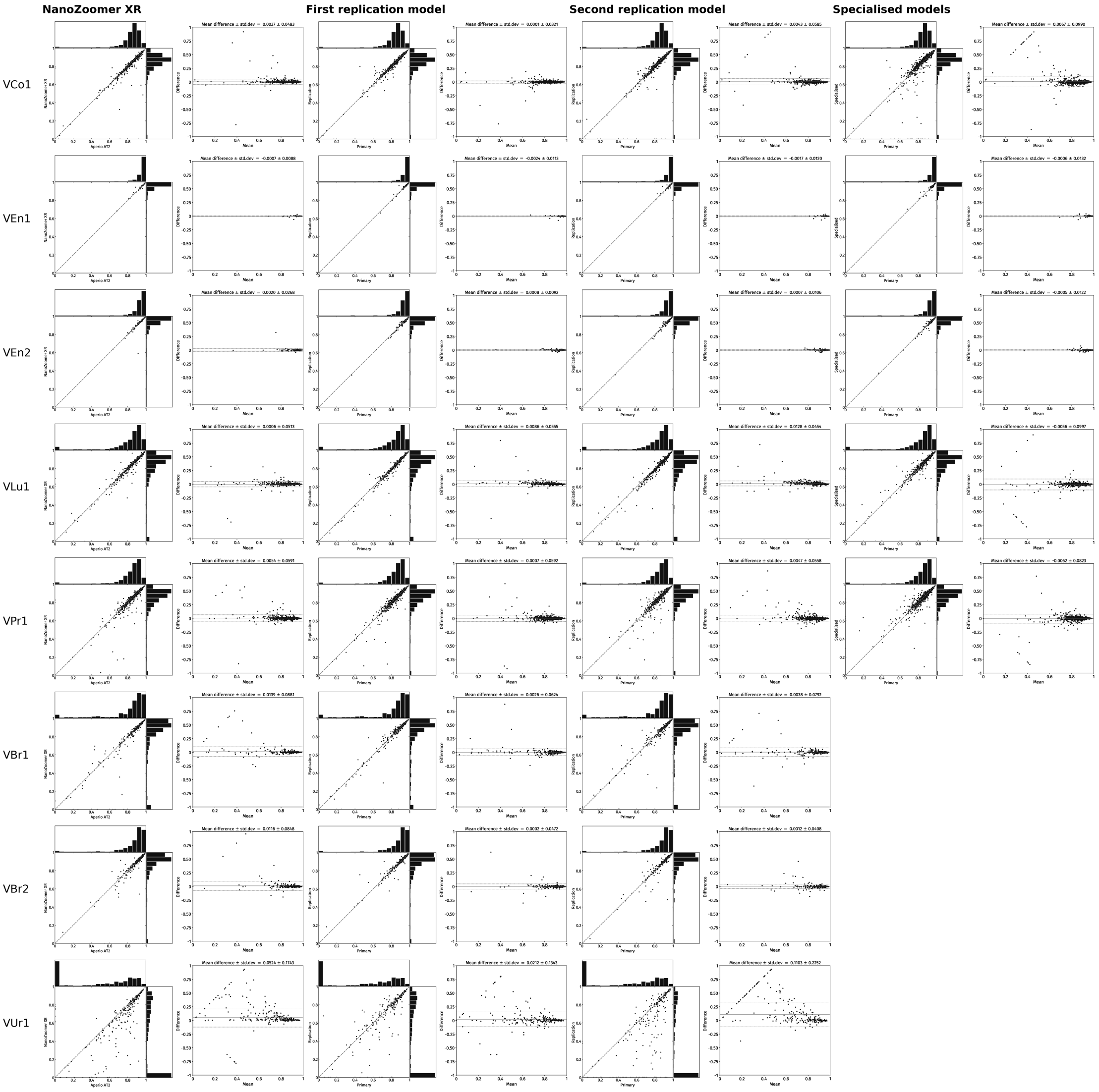

Figure S17: Per scan comparison
Per scan comparison viewed as a scatter plot (left) and mean difference plot (right) organised in columns for the primary model on Aperio AT2 vs the primary model on NanoZoomer XR, first replication model on Aperio AT2, second replication model on Aperio AT2, and the specialised models on Aperio AT2, respectively. Each scan result is the automatic vs manual segmentation measured with DSC.

2.3 Region areas

Table S9: Performance in true positive regions
Primary model evaluated on WSIs from Aperio AT2 in the validation cohorts

Cohort	Images containing true positive regions<br>Count (proportion)	Dice similarity coefficient (%)<br>Mean (95% CI)
VCo1	1058 (91.6%)	86.10 (85.71 – 86.50)
VEn1	76 (98.7%)	95.05 (93.82 – 96.27)
VEn2	150 (98.7%)	93.18 (92.22 – 94.15)
VLu1	440 (84.3%)	86.48 (85.75 – 87.22)
VPr1	731 (94.1%)	87.76 (87.29 – 88.24)
VBr1	277 (89.4%)	90.06 (89.26 – 90.86)
VBr2	295 (97.0%)	90.71 (89.94 – 91.48)
VUr1	217 (65.4%)	86.98 (86.09 – 87.87)

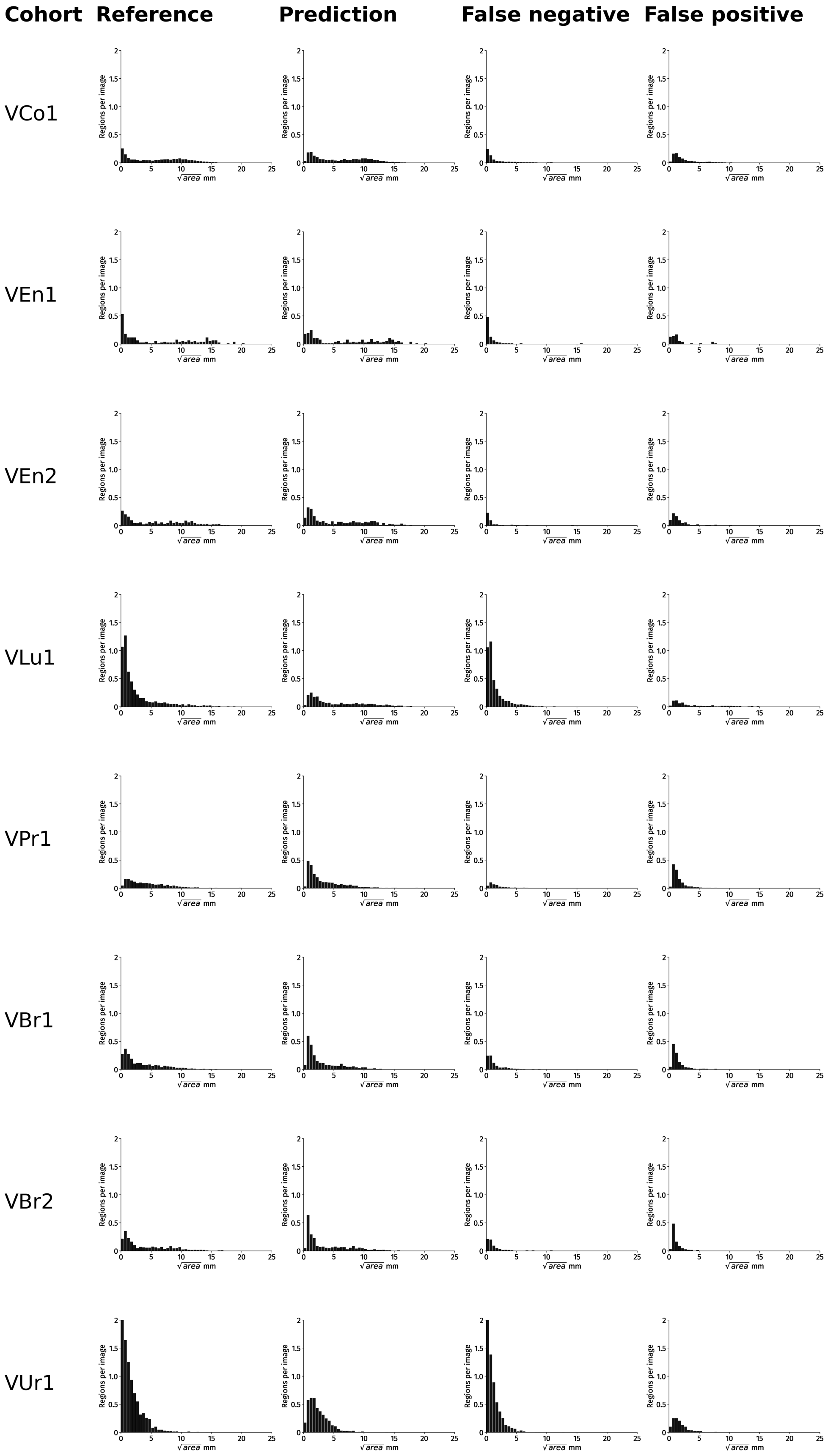

Figure S18: Region areas in Aperio AT2 WSIs from the validation cohorts

Reference regions are from the manual segmentation and predicted regions are from the automatic segmentation with the primary model.

2.4 Bladder subgroup analysis

Table S10: Subgroup analyses in bladder cohorts

Cohort	Group	All scans		Scans with predictions	
		Count	Dice similarity coefficient (%) Mean (95% CI)	Count	Dice similarity coefficient (%) Mean (95% CI)
VUr1	All scans	332	49.77 (45.64 – 53.90)	224	73.77 (71.16 – 76.39)
	pT stage				
	pTa or pTis	256	43.99 (39.24 – 48.73)	158	71.27 (67.91 – 74.63)
	pT1	76	69.27 (62.39 – 76.15)	66	79.76 (76.32 – 83.21)
BLCA	All scans	431	84.49 (82.40 – 86.59)	411	88.61 (87.42 – 89.79)
	pT stage				
	pT0	1	93.16	1	93.16
	pT1	4	91.12 (80.31 – 01.93)	4	91.12 (80.31 – 01.93)
	pT2	112	88.63 (85.86 – 91.39)	110	90.24 (88.60 – 91.87)
	pT3	203	87.62 (85.21 – 90.04)	199	89.38 (87.66 – 91.11)
	pT4	58	87.49 (81.89 – 93.08)	56	90.61 (87.01 – 94.21)
	pTx	1	79.90	1	79.90
	Missing	52	59.45 (49.74 – 69.17)	40	77.29 (73.04 – 81.55)
	Fragmented tissue				
	True	87	66.38 (59.91 – 72.86)	75	77.01 (73.41 – 80.60)
	False	342	89.05 (87.27 – 90.84)	334	91.19 (90.15 – 92.22)
	Missing	2	92.78 (87.98 – 97.58)	2	92.78 (87.98 – 97.58)

2.5 Intra- and inter-observer variability

Table S11: Intra- and inter-observer variability in VBr2

MP-1: annotations by Manohar Pradhan in the first round. *MP-2*: annotations by Manohar Pradhan in the second round. *LV*: annotations by Ljiljana Vlatkovic. *Auto*: annotations by the primary automatic segmentation model presented in this study.

Comparison	Dice similarity coefficient (%)	
	Mean (95% CI)	Median (IQR)
MP-2 vs MP-1	91.37 (90.20 – 92.54)	93.96 (90.19 – 96.85)
LV vs MP-1	74.25 (71.94 – 76.57)	79.55 (65.33 – 90.16)
Auto vs MP-1	88.16 (86.52 – 89.80)	92.45 (86.41 – 95.64)
LV vs MP-2	76.69 (74.39 – 78.99)	82.82 (67.94 – 91.40)
Auto vs MP-2	87.78 (86.05 – 89.51)	92.16 (86.37 – 95.54)
Auto vs LV	72.06 (69.63 – 74.48)	77.91 (62.76 – 88.36)

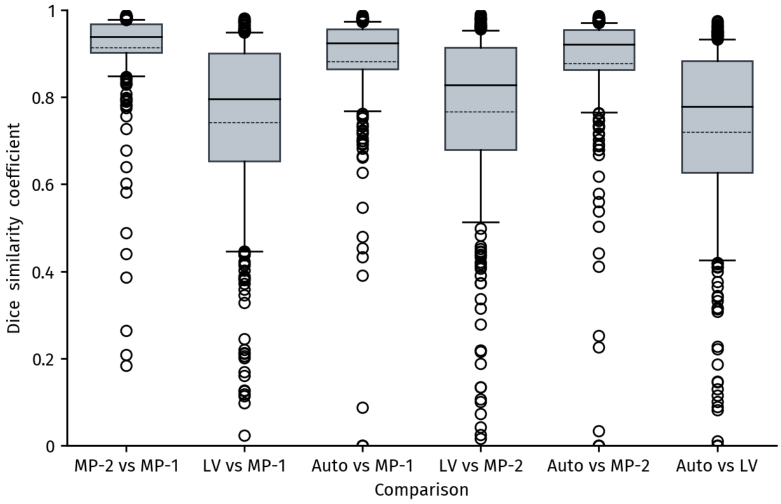

Figure S19: Intra- and inter-observer variability in VBr2

For each two observers that are compared, the plot summarises the Dice similarity coefficient with interquartile range (coloured box), mean value (perforated horizontal line), median value (solid horizontal line), the 10th and 90th percentile (whiskers), and outliers (circles). See Table S11 for label explanations.

2.6 Performance evaluation in five different scanners

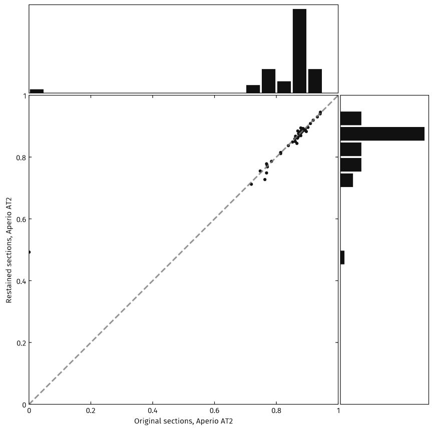

(a) Original vs restained, Aperio AT2

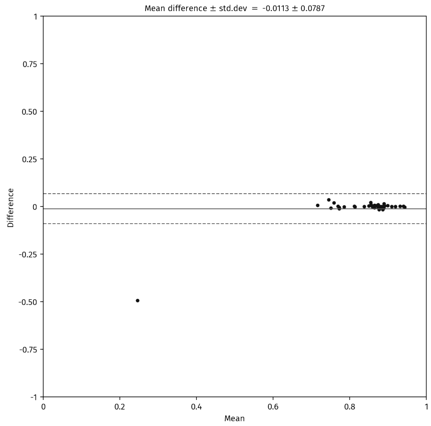

(b) Original vs restained, Aperio AT2

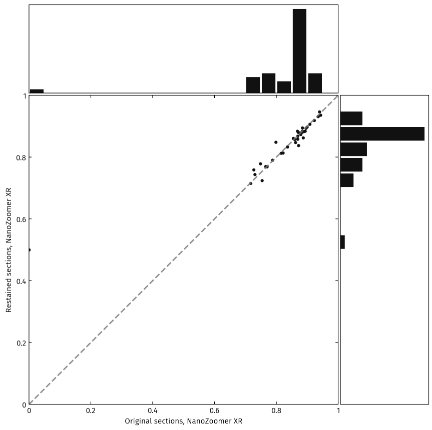

(c) Original vs restained, NanoZoomer XR

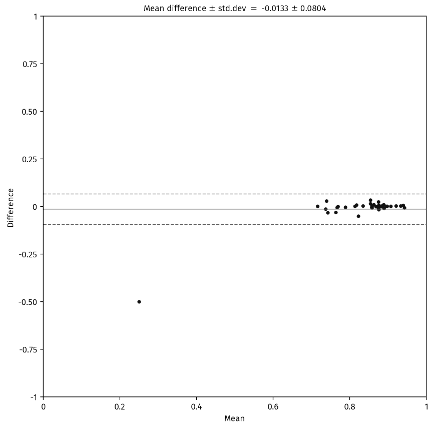

(d) Original vs restained, NanoZoomer XR

Figure S20: Original vs restained mean difference
Dice similarity coefficient of 39 tissue sections that were restained.

Table S12: Primary model performance in VCo1 scanned on five different scanners
Only slides that were successfully scanned on all five scanners were included

Scanner	Scans	Dice similarity coefficient (%) Mean (95% CI)
Aperio AT2	1 152	84.58 (83.90 – 85.26)
Aperio GT 450 DX	1 152	82.85 (81.91 – 83.80)
KF-PRO-400	1 152	84.13 (83.36 – 84.90)
NanoZoomer XR	1 152	84.21 (83.51 – 84.92)
Pannoramic 1000	1 152	83.35 (82.51 – 84.18)

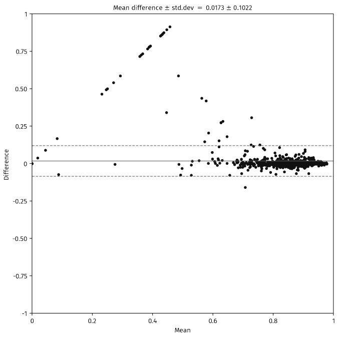

(a) Aperio AT2 vs Aperio GT 450 DX

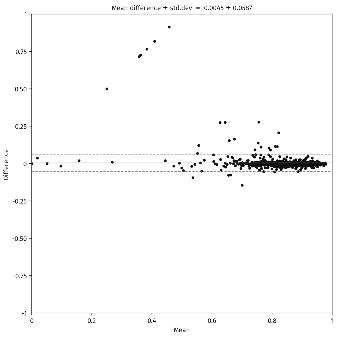

(b) Aperio AT2 vs KF-PRO-400

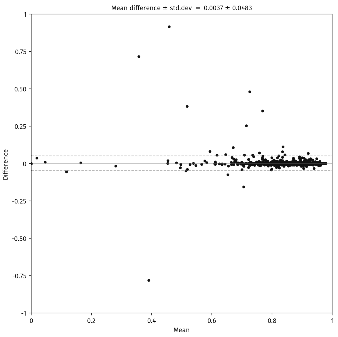

(c) Aperio AT2 vs NanoZoomer XR

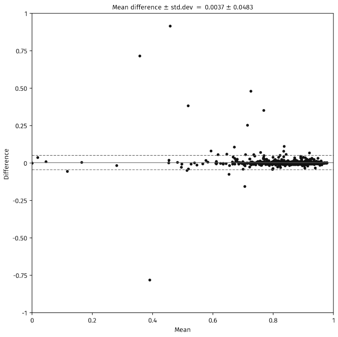

(d) Aperio AT2 vs Pannoramic 1000

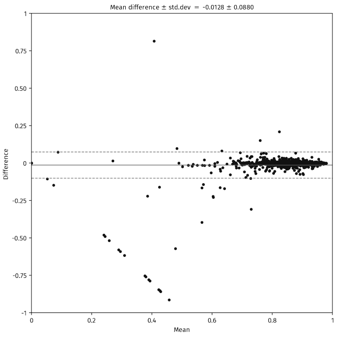

(e) Aperio GT 450 DX vs KF-PRO-400

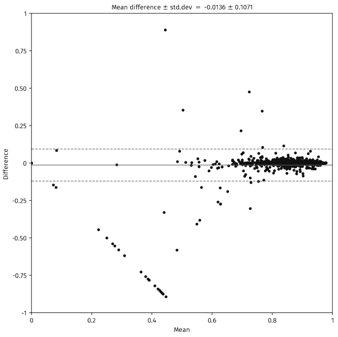

(f) Aperio GT 450 DX vs NanoZoomer XR

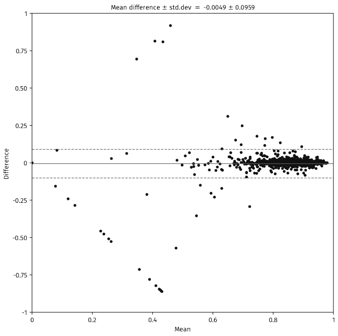

(g) Aperio GT 450 DX vs Pannoramic 1000

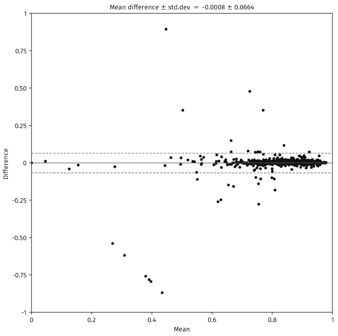

(h) KF-PRO-400 vs KF-PRO-400

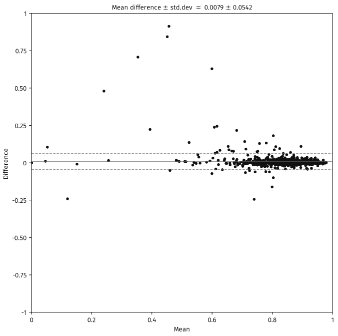

(i) KF-PRO-400 vs Pannoramic 1000

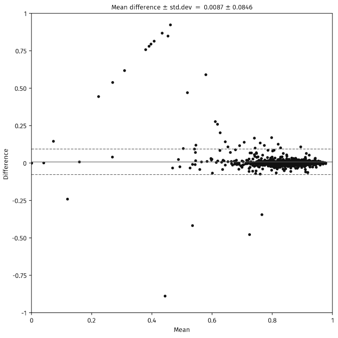

(j) NanoZoomer XR vs Pannoramic 1000

Figure S21: Scanner vs scanner mean difference
Dice similarity coefficient of 1 152 glass slides from VCo1 scanned with five different scanners. Each plot compares results from one scanner vs another, and the difference is computed as the Dice similarity coefficient of the scanner mentioned first minus the scanner mentioned last.

2.7 Comparison with MedSAM

Table S13: MedSAM performance in validation datasets
Dice similarity coefficient percent mean (95% CI) versus manual segmentation.

Cohort	Method	Aperio AT2	NanoZoomer XR
VCo1	Primary	84.54 (83.86 – 85.23)	84.17 (83.46 – 84.88)
	MedSAM-tumour	79.38 (78.69 – 80.07)	79.43 (78.72 – 80.13)
	MedSAM-tissue	48.23 (47.13 – 49.33)	48.78 (47.72 – 49.84)
	Tumour-bbox	73.78 (73.11 – 74.45)	73.71 (73.03 – 74.39)
VEn1	Primary	95.28 (94.20 – 96.36)	95.35 (94.30 – 96.40)
	MedSAM-tumour	88.95 (86.34 – 91.56)	89.75 (87.20 – 92.30)
	MedSAM-tissue	62.51 (57.25 – 67.76)	61.50 (55.95 – 67.06)
	Tumour-bbox	82.35 (79.39 – 85.31)	82.29 (79.33 – 85.26)
VEn2	Primary	93.40 (92.31 – 94.49)	93.20 (92.03 – 94.37)
	MedSAM-tumour	86.56 (85.10 – 88.02)	86.57 (85.14 – 87.99)
	MedSAM-tissue	53.12 (49.51 – 56.72)	52.69 (49.04 – 56.35)
	Tumour-bbox	78.49 (76.68 – 80.30)	78.48 (76.67 – 80.28)
VLu1	Primary	82.22 (80.54 – 83.91)	82.16 (80.50 – 83.82)
	MedSAM-tumour	72.23 (70.75 – 73.71)	72.32 (70.84 – 73.81)
	MedSAM-tissue	46.55 (44.57 – 48.52)	46.79 (44.84 – 48.73)
	Tumour-bbox	66.69 (65.28 – 68.11)	66.64 (65.22 – 68.05)
VPr1	Primary	84.36 (83.33 – 85.38)	83.82 (82.72 – 84.92)
	MedSAM-tumour	65.77 (64.19 – 67.36)	65.75 (64.17 – 67.34)
	MedSAM-tissue	27.78 (26.41 – 29.15)	27.70 (26.36 – 29.05)
	Tumour-bbox	60.09 (58.68 – 61.51)	60.05 (58.63 – 61.47)
VBr1	Primary	82.40 (79.94 – 84.86)	81.01 (78.28 – 83.73)
	MedSAM-tumour	81.36 (79.49 – 83.22)	81.16 (79.28 – 83.05)
	MedSAM-tissue	34.40 (32.08 – 36.72)	36.19 (33.86 – 38.52)
	Tumour-bbox	70.48 (68.70 – 72.26)	71.29 (69.56 – 73.03)
VBr2	Primary	88.16 (86.52 – 89.80)	87.00 (85.07 – 88.92)
	MedSAM-tumour	82.54 (80.55 – 84.53)	82.79 (80.84 – 84.75)
	MedSAM-tissue	42.49 (39.50 – 45.48)	44.60 (41.66 – 47.55)
	Tumour-bbox	72.93 (71.04 – 74.81)	73.92 (72.09 – 75.75)
VUr1	Primary	49.77 (45.64 – 53.90)	44.53 (40.48 – 48.58)
	MedSAM-tumour	74.53 (72.27 – 76.78)	74.28 (72.01 – 76.55)
	MedSAM-tissue	64.63 (61.77 – 67.49)	64.58 (61.73 – 67.42)
	Tumour-bbox	74.45 (72.28 – 76.62)	74.45 (72.27 – 76.63)

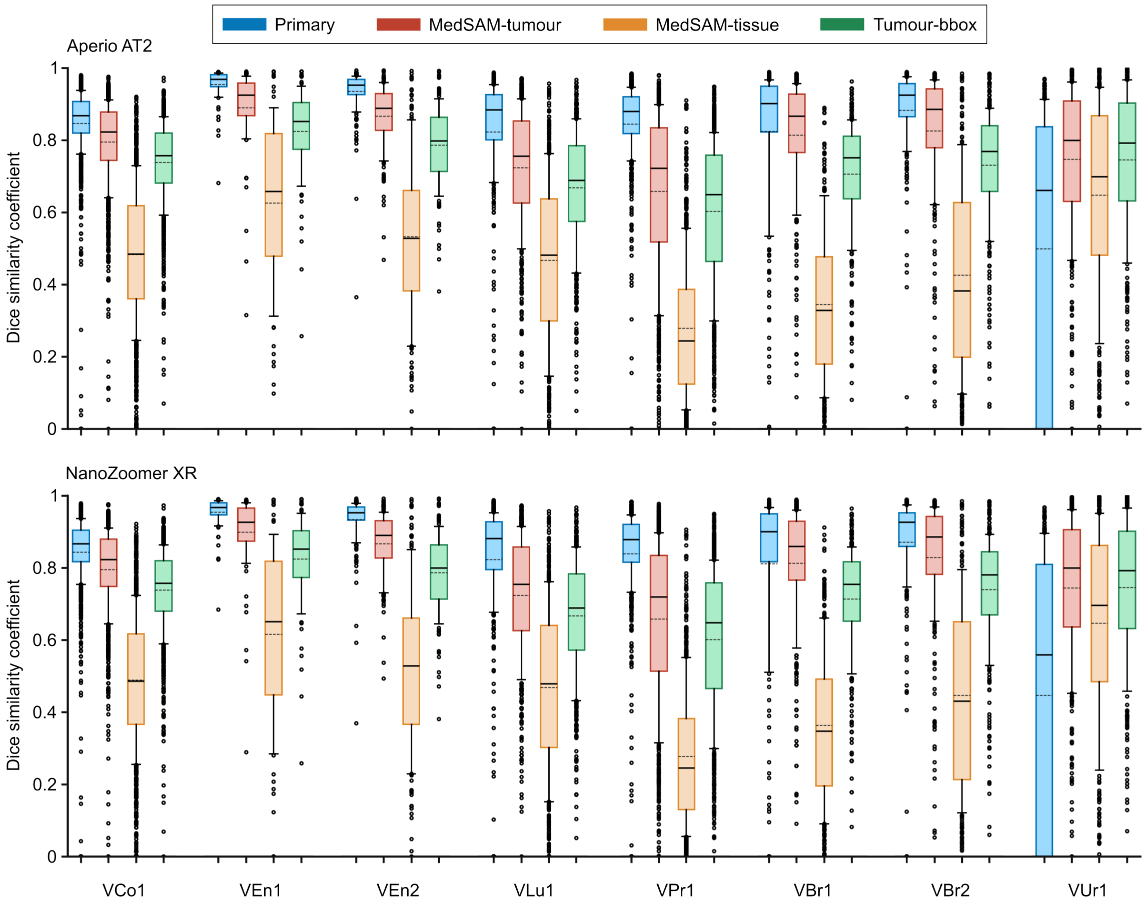

Figure S22: MedSAM performance in validation datasets
Segmentation performance in validation cohorts from Aperio AT2 (top) and NanoZoomer XR (bottom). Evaluated methods are MedSAM prompted by tumour bounding box (MedSAM-tumour), MedSAM prompted by tissue bounding box (MedSAM-tissue) and bounding boxes of manual annotations (Tumour-bbox). The result of the primary model presented in this study is included for reference (Primary). Summary statistics are given in Supplementary Table S13.

2.8 Varying hysteresis threshold in segmentation post-processing

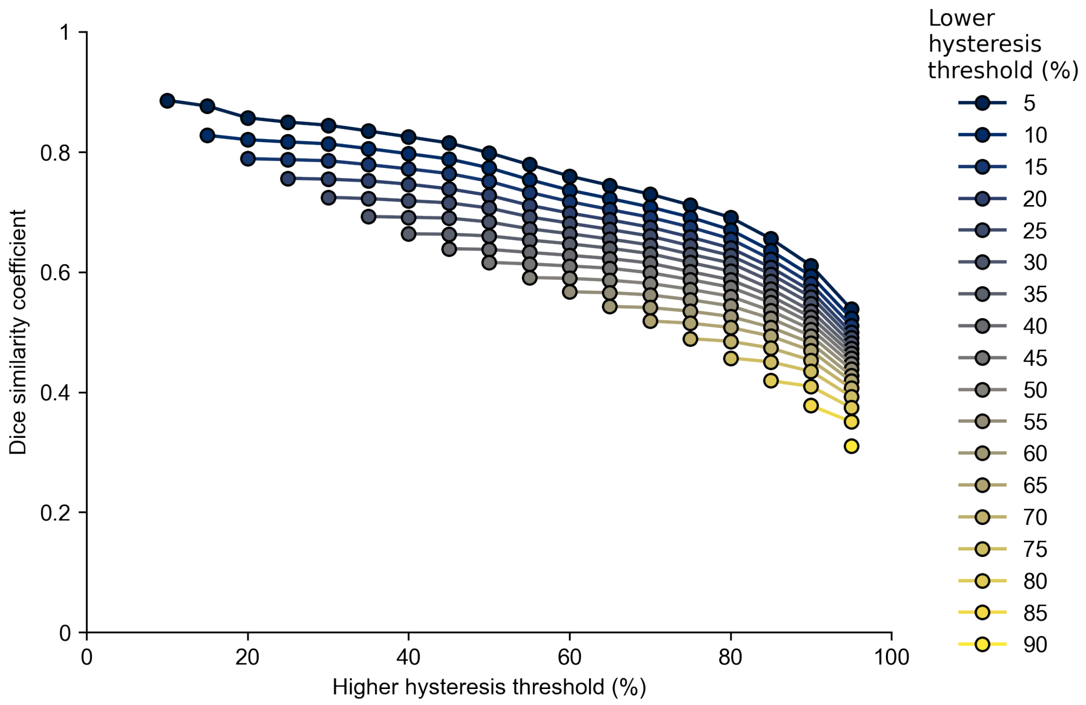

Figure S23: Varying hysteresis threshold
Comparison between automatic and manual segmentation in Aperio AT2 scans from VUr1. The various results are obtained by adjusting the lower and higher hysteresis thresholds in the segmentation post-processing. Lower thresholds are: $[5\%, 10\%, \ldots, 90\%]$. For each low threshold x, we have the higher thresholds: $[x + 5\%, x + 10\%, \ldots, 95\%]$.

Table S14: Best performance in VUr1 with varying hysteresis thresholds
Primary model evaluated on Aperio AT2 WSIs from VUr1, where the probability map is smoothed before a hysteresis threshold with lower value 5% and higher value 10% is used. These threshold values are from the run with highest mean Dice similarity coefficient value.

Name	WSI count	Mean (%)	Median (%)
True positive rate	332	84.36	95.69
False negative rate	332	15.63	04.25
True negative rate	332	99.90	99.98
False positive rate	332	00.10	00.02
Positive predictive value	332	95.42	99.98
Negative predictive value	332	85.27	93.77
Informedness	332	84.26	95.67
Markedness	332	80.69	93.45
Dice similarity coefficient	332	88.61	97.77

3 Segmentation network optimisation

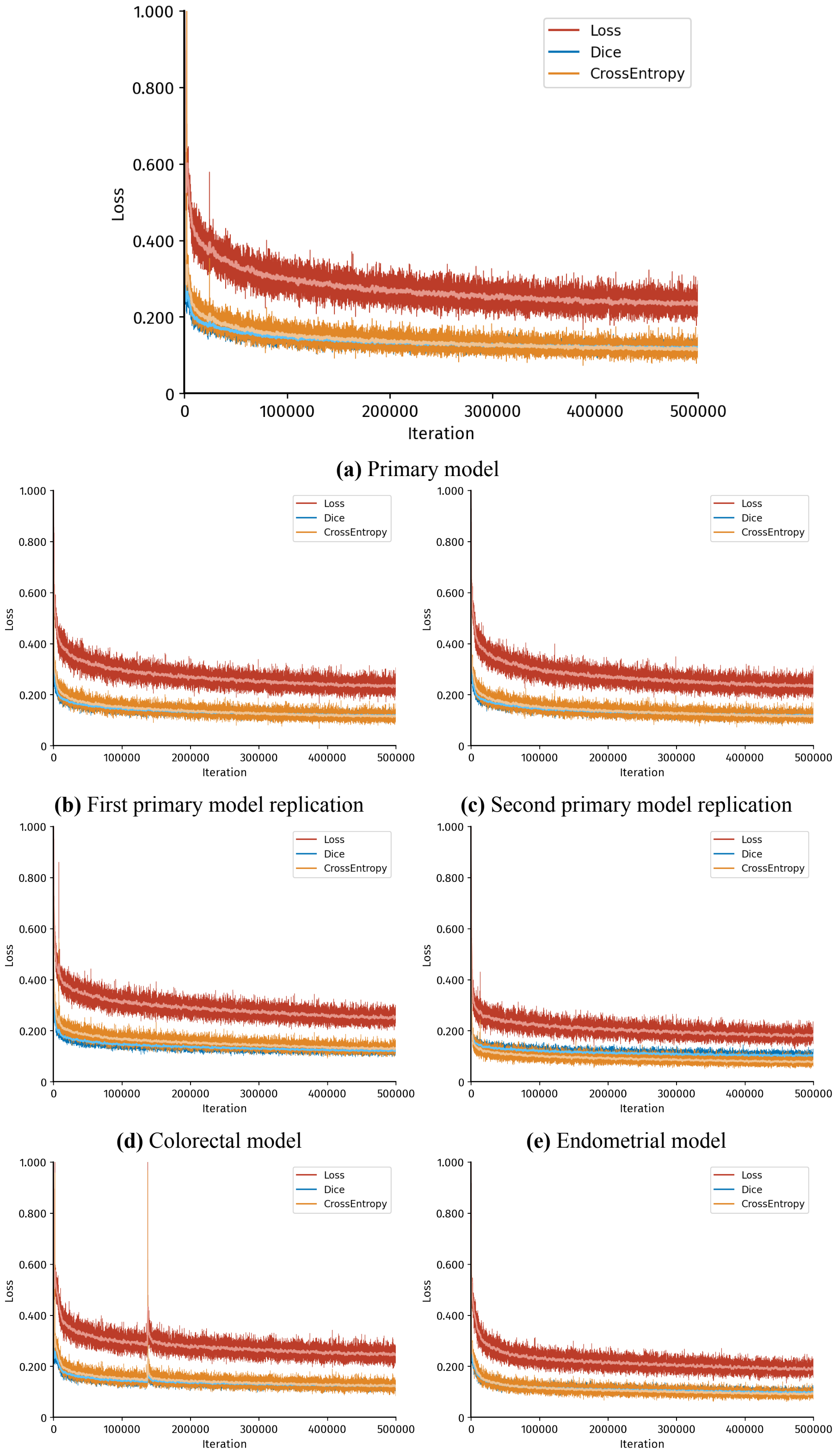

(a) Primary model

(b) First primary model replication

(c) Second primary model replication

(d) Colorectal model

(e) Endometrial model

(f) Lung model

(g) Prostate model

Figure S24: Segmentation network optimisation loss curve
Loss is the sum of *Dice* loss and *CrossEntropy* loss. Values are averaged over 20 iterations.

4 TCGA cohorts

33 TCGA cohorts were downloaded from `https://www.cancer.gov/tcga` in December 2021. Only scans from so-called *diagnostic slides* were considered. LAML did not have diagnostic slides leaving the 32 cohorts. We only made use of BLCA, LUAD, LUSC and PRAD since these were the only one we had manually annotated at the time of conducting the study.

Since we segment the scans at resolution 1 µm per pixel, we exclude all scans were the scan resolution information is not present. We note that for some scans there are an apparent disagreement between the magnification information present in the `PROPERTY_NAME_MPP_X` and `PROPERTY_NAME_MPP_Y` properties and the information from the `PROPERTY_NAME_OBJECTIVE_POWER` property (objective power at the lowest scan level. This should normally be around 0.25 µm per pixel for objective power 40×), but as we can see from the statistics presented in section 4.3, this is not always the case. This might indicate that the actual scan resolution is different from what it is stated, but we use the magnification in the `PROPERTY_NAME_MPP_*` properties, and do not exclude any scans based on this apparent discrepancy.

We do not exclude any scans based on their appearance or quality, although we find scans with attributes such as pen markings, air bubbles, dust, tissue out of focus, different stain than H&E, etc.

All scans are stored in the `.svs` format and the openslide property `PROPERTY_NAME_VENDOR` for all scans is equal to Aperio. Based on visual appearance, we find it unlikely that all scans are in fact originally scanned with Aperio, but have not excluded any scans based on this.

Clinical data presented in Table S15 and Fig. S29 are from the TCGA Pan-Cancer Clinical Data Resource which publication should be consulted when interpreting the included variables and their values.[1]

4.1 Included scans

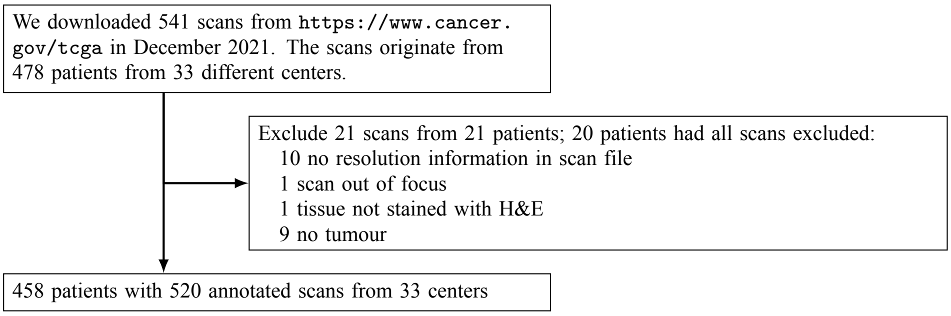

Figure S25: Flow from downloaded scans to annotated scans for the LUAD cohort

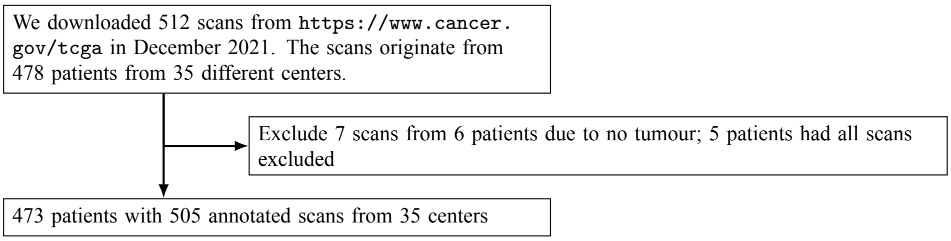

Figure S26: Flow from downloaded scans to annotated scans for the LUSC cohort

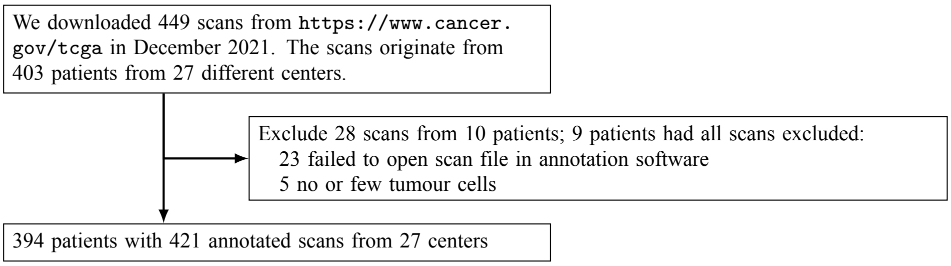

Figure S27: Flow from downloaded scans to annotated scans for the PRAD cohort

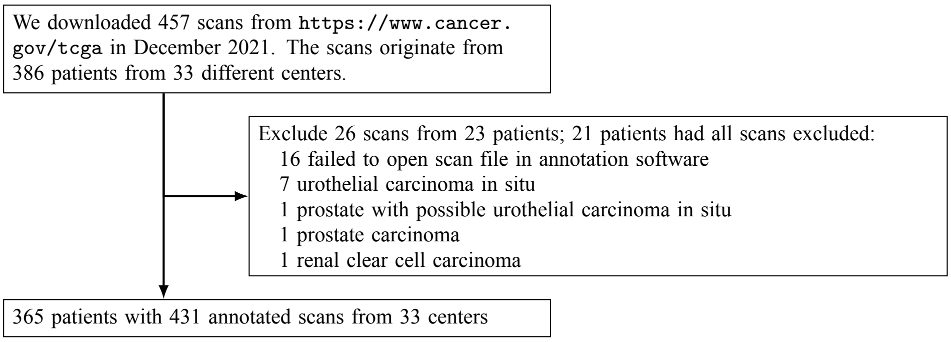

Figure S28: Flow from downloaded scans to annotated scans for the BLCA cohort

4.2 Baseline characteristics

Table S15: Baseline characteristics for included TCGA cohorts
Data are given as *median (interquartile range)* or *count (percentage)*. Time to *event* statistics are based only on patients with the respective event.

	BLCA	LUAD	LUSC	PRAD
Patient count	365	458	473	394
Age at diagnosis				
Years	69 (60 – 76)	66 (59 – 73)	68 (62 – 74)	61 (56 – 66)
Missing	0	18 (4%)	9 (2%)	0
Sex				
Female	91 (25%)	248 (54%)	119 (25%)	0
Male	274 (75%)	210 (46%)	354 (75%)	394 (100%)
Cancer-specific death				
False	242 (66%)	325 (71%)	341 (72%)	387 (98%)
True	110 (30%)	100 (22%)	83 (18%)	5 (1%)
Missing	13 (4%)	33 (7%)	49 (10%)	2 (1%)
Time to cancer-specific death				
Years	1.1 (0.6 – 1.7)	1.7 (0.9 – 2.7)	1.7 (1.0 – 2.9)	3.6 (2.2 – 5.1)
Missing	1 (1%)	2 (2%)	2 (2%)	0
Overall death				
False	202 (55%)	298 (65%)	271 (57%)	385 (98%)
True	163 (45%)	160 (35%)	202 (43%)	9 (2%)
Time to overall death				
Years	1.1 (0.6 – 1.7)	1.7 (0.8 – 2.9)	1.5 (0.8 – 3.1)	3.6 (2.0 – 6.8)
Missing	1 (1%)	3 (2%)	4 (2%)	0
New tumour event				
False	209 (57%)	272 (59%)	336 (71%)	322 (82%)
True	156 (43%)	186 (41%)	137 (29%)	72 (18%)
Time to new tumour event				
Years	0.8 (0.5 – 1.5)	1.2 (0.7 – 1.9)	1.2 (0.7 – 2.2)	1.8 (0.9 – 3.0)
Missing	0	2 (1%)	1 (1%)	0
Follow-up time				
Years	1.4 (0.9 – 2.5)	1.8 (1.2 – 3.1)	1.8 (0.9 – 3.5)	2.8 (1.8 – 4.6)
Missing	1 (<1%)	8 (2%)	6 (1%)	0
pN stage				
pN0	207 (57%)	301 (66%)	302 (64%)	276 (70%)
pN1	40 (11%)	89 (19%)	125 (26%)	54 (14%)
pN2	71 (19%)	55 (12%)	36 (8%)	0
pN3	7 (2%)	2 (<1%)	5 (1%)	0
pNx	34 (9%)	10 (2%)	5 (1%)	0
Missing	6 (2%)	1 (<1%)	0	64 (16%)
pT stage				
pT0	1 (<1%)	0	0	0
pT1	3 (1%)	157 (34%)	108 (23%)	0
pT2	108 (30%)	241 (53%)	280 (59%)	153 (39%)
pT3	174 (48%)	41 (9%)	64 (14%)	227 (58%)
pT4	51 (14%)	16 (3%)	21 (4%)	8 (2%)
pTx	1 (<1%)	3 (1%)	0	0
Missing	27 (7%)	0	0	6 (2%)
Stage				
I	2 (1%)	250 (55%)	234 (49%)	0
II	115 (32%)	113 (25%)	152 (32%)	0
III	123 (34%)	63 (14%)	77 (16%)	0
IV	123 (34%)	25 (5%)	6 (1%)	0
Missing	2 (1%)	7 (2%)	4 (1%)	394 (100%)

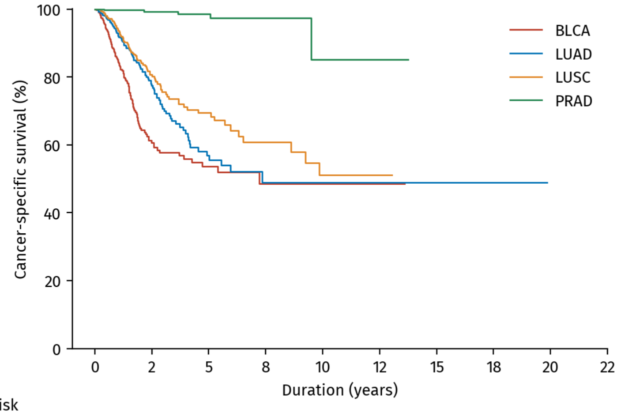

Number at risk

BLCA (missing *n*=14)	351	89	40	12	4	1	0	0	0	0
LUAD (missing *n*=40)	418	137	44	15	8	5	3	3	0	0
LUSC (missing *n*=55)	418	157	68	27	12	4	0	0	0	0
PRAD (missing *n*=2)	392	227	82	16	4	1	0	0	0	0

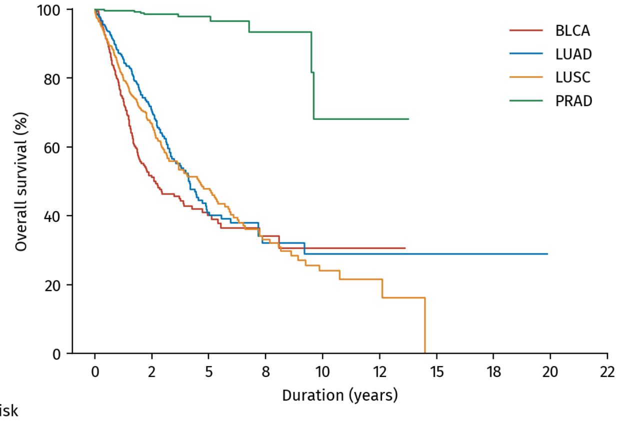

Number at risk

BLCA (missing *n*=1)	364	90	40	12	4	1	0	0	0	0
LUAD (missing *n*=8)	450	150	47	16	8	5	3	3	0	0
LUSC (missing *n*=6)	467	181	79	31	13	5	0	0	0	0
PRAD	394	228	83	16	4	1	0	0	0	0

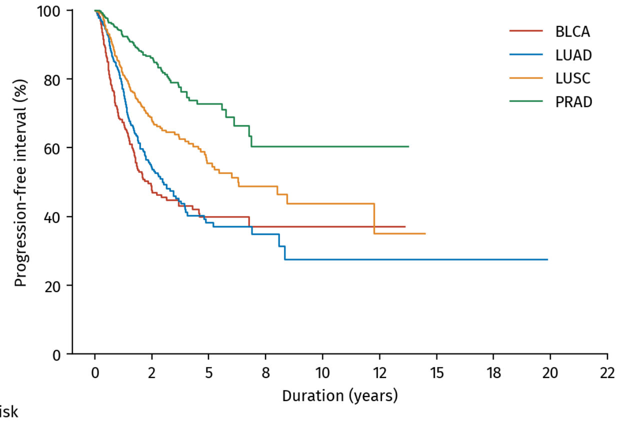

Number at risk

BLCA	365	72	31	11	4	1	0	0	0	0
LUAD (missing *n*=8)	450	109	34	12	5	4	3	3	0	0
LUSC (missing *n*=5)	468	154	64	24	10	4	0	0	0	0
PRAD	394	198	61	12	4	1	0	0	0	0

Figure S29: Kaplan-Meier analysis for included TCGA materials
Duration is years since initial diagnosis.

4.3 WSI dimensions

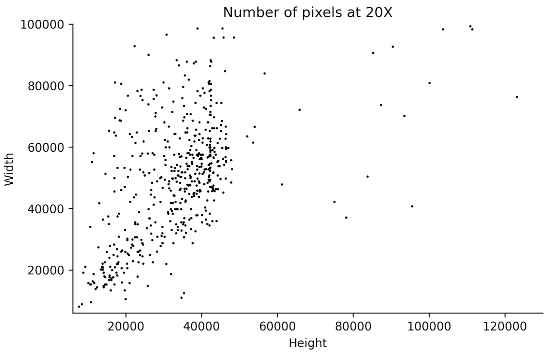

(a) TCGA LUAD

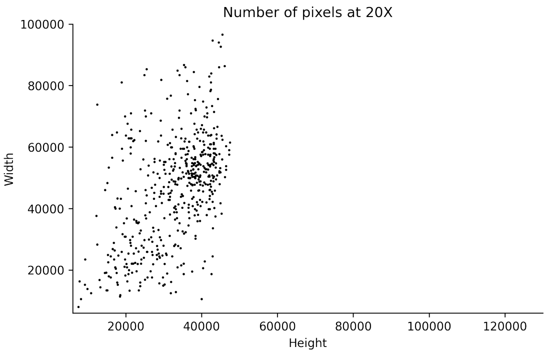

(b) TCGA LUSC

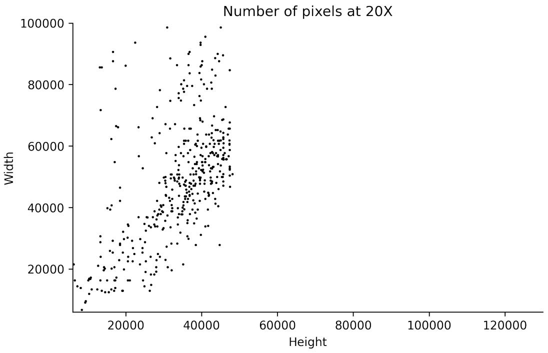

(c) TCGA PRAD

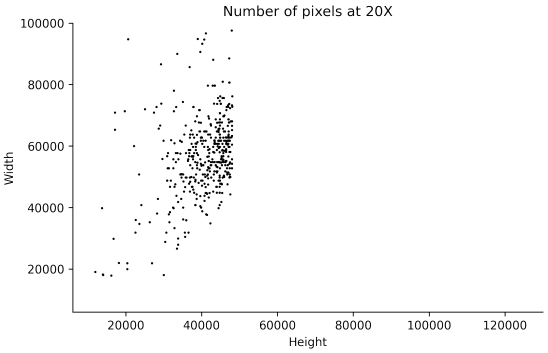

(d) TCGA BLCA

Figure S30: TCGA WSI dimensions at 20× magnification
Height and width at scan level 0 is gathered using openslide's slide property `level_dimensions[0]`. These values are scaled to 20× using openslide's `PROPERTY_NAME_OBJECTIVE_POWER`

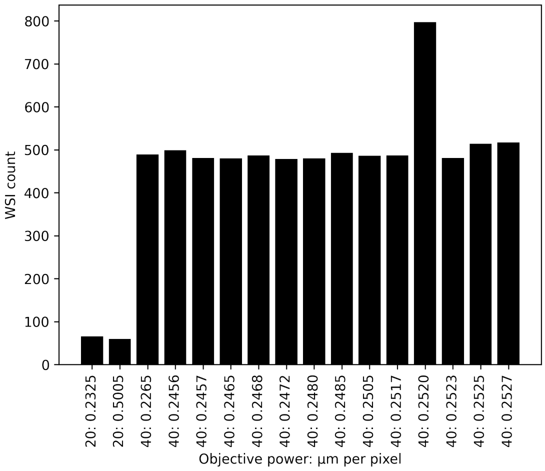

(a) TCGA LUAD

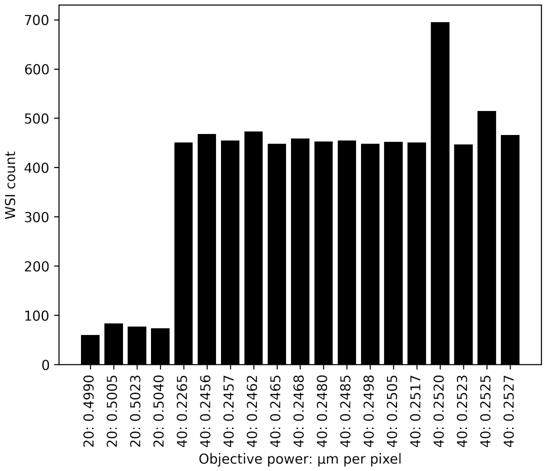

(b) TCGA LUSC

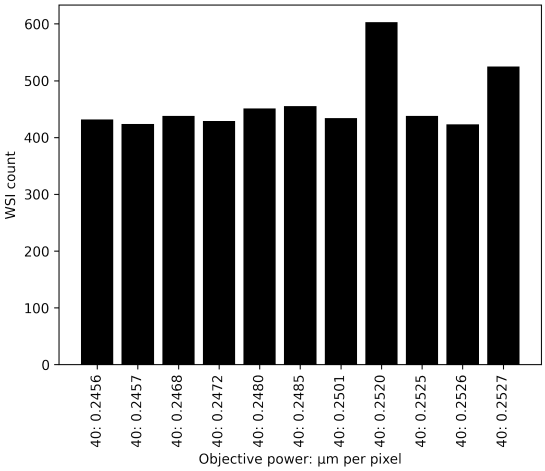

(c) TCGA PRAD

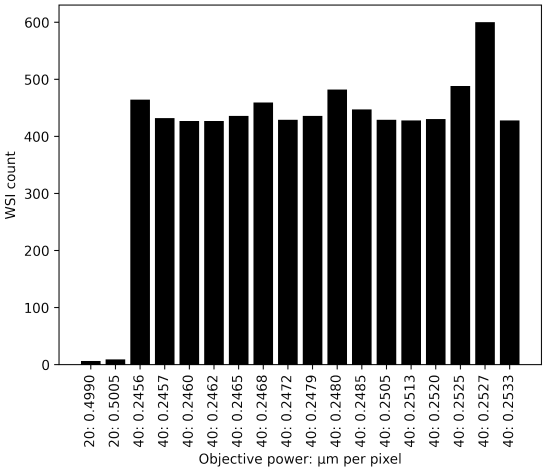

(d) TCGA BLCA

Figure S31: TCGA WSI objective power and µm per pixel

Number of WSIs with a specific *objective power: µm per pixel* combination. Objective power are from openslide's `PROPERTY_NAME_OBJECTIVE_POWER`, while µm per pixel is the average of openslide's `PROPERTY_NAME_MPP_X` and `PROPERTY_NAME_MPP_Y`.

5 Protocol amendment

A study protocol that was completed before the method validation is included in its entirety in section 6. It is included exactly how it existed at March 24, 2023, and any errors that were discovered after this date were corrected and presented in the current section rather than editing the protocol document itself.

5.1 Protocol section 1.1.4 DEn1 — Endometrial carcinoma

The first paragraph (lines 184 and 185) was left from a previous draft version and should be removed. This material is not part of the MoMaTEC study and the sentence at lines 188 and 189 should therefore be removed: *This material originates from the MoMaTEC (Molecular Markers in Treatment of Endometrial Cancer) trial (NCT number NCT00598845).[8, 9]*

5.2 Protocol section 1.2.8 VUr1 — Urothelial carcinoma

Earliest diagnosis date for patient inclusion should be corrected from 1992 to 2002. Specifically, included are all patients with non-muscle invasive urothelial carcinoma of the bladder and without upper urinary tract urothelial carcinoma with primary diagnosis date between 1.1.2002 and 1.1.2011 at Stavanger University Hospital, Norway.

5.3 Protocol Table 5 and protocol Fig. 18

Patients with small cell carcinoma were left out when summarising baseline characteristics. Corrected protocol table 5 is found in Table S16 and corrected protocol Fig. 18 is found in Fig. S32.

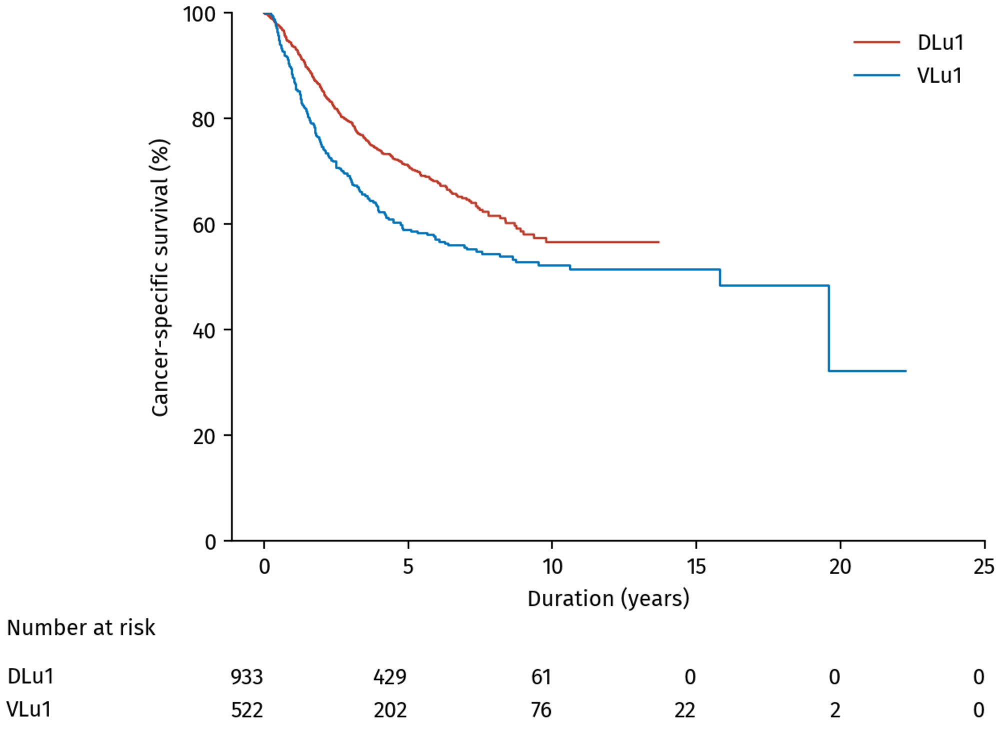

Figure S32: Kaplan-Meier analysis of survival in lung carcinoma materials. Duration is years since surgery for DLu1 and years since diagnosis for VLu1.

Table S16: Baseline characteristics in lung carcinoma cohorts. Starting point is at surgery for DLu1 and at diagnosis for VLu1.

	DLu1	VLu1
Patient count	933	522
Age		
Years	68 (62 – 73)	68 (60 – 73)
Sex		
Female	465 (50%)	168 (32%)
Male	468 (50%)	354 (68%)
Histological type		
Adenocarcinoma	521 (56%)	226 (43%)
Adenosquamous carcinoma	16 (2%)	3 (1%)
Bronchioloalveolar carcinoma	8 (1%)	0
Carcinoid	42 (5%)	0
Large cell carcinoma	29 (3%)	0
Large cell neuroendocrine carcinoma	6 (1%)	0
Salivary gland type lung carcinoma	5 (1%)	0
Small cell carcinoma	11 (1%)	0
Squamous cell carcinoma	287 (31%)	289 (55%)
Undifferentiated carcinoma	4 (<1%)	3 (1%)
Mixed	2 (<1%)	0
Other	2 (<1%)	1 (<1%)
Cancer-specific death		
False	641 (69%)	316 (61%)
True	292 (31%)	206 (39%)
Time to cancer-specific death		
Years	2.2 (1.3 – 3.8)	1.7 (0.9 – 3.2)
Follow-up time		
Years	4.6 (2.5 – 6.8)	3.6 (1.4 – 7.4)
pN stage		
pN0	676 (72%)	366 (70%)
pN1	187 (20%)	102 (20%)
pN2	70 (8%)	54 (10%)
pT stage		
pT1	328 (35%)	171 (33%)
pT2	439 (47%)	196 (38%)
pT3	137 (15%)	98 (19%)
pT4	29 (3%)	57 (11%)
Stage		
I	511 (55%)	224 (43%)
II	276 (30%)	170 (33%)
III	135 (14%)	128 (25%)
IV	11 (1%)	0

5.4 Protocol Figs. 27, 28, 29, 30

A bug in the pixel counting script swapped counts for background and non-annotated foreground. This means that protocol Figs. 27 (b), 28 (b), 29 (b), 29 (c), 30 (b) and 30 (c) are wrong. The corrected Figs. S33 to S36 should replace the erroneous protocol Figs. 27, 28, 29 and 30, respectively.

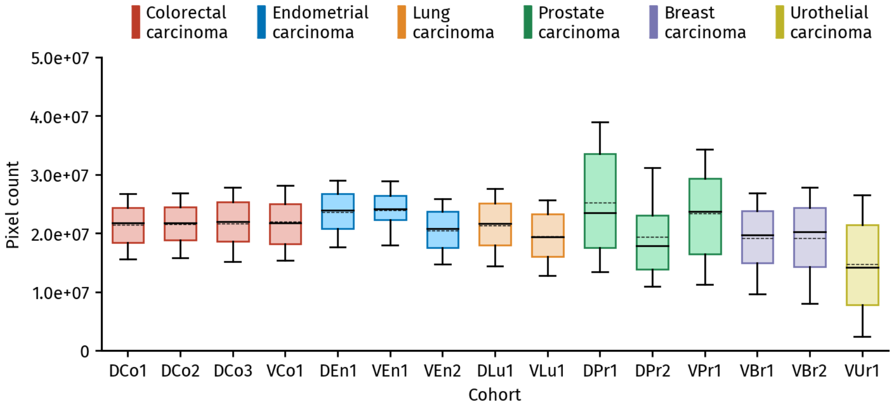

(a) Image area

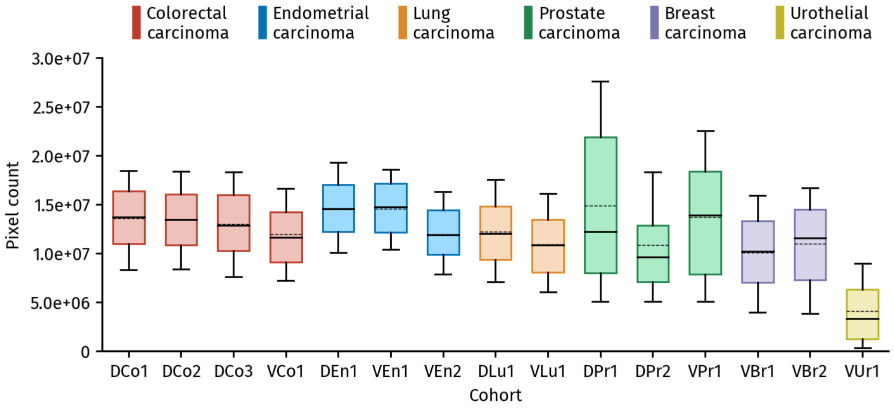

(b) Foreground area

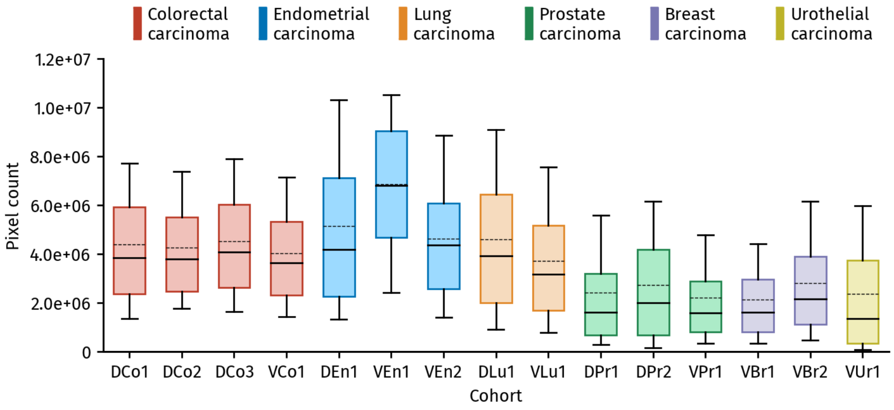

(c) Annotation area

Figure S33: Area in number of pixels at resolution 5 µm per pixel in Aperio AP2 scans. “Foreground” is foreground without annotation and “Annotation” is foreground with annotation. Background exclusion masks are applied on all images. Note the difference in vertical axis range between subplots.

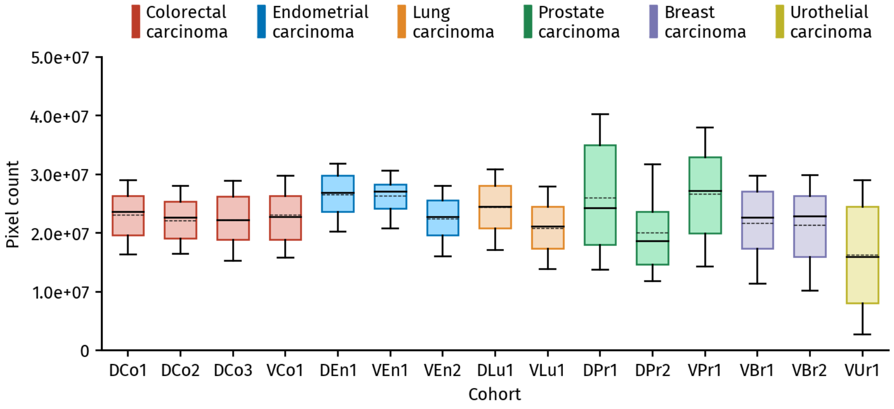

(a) Image area

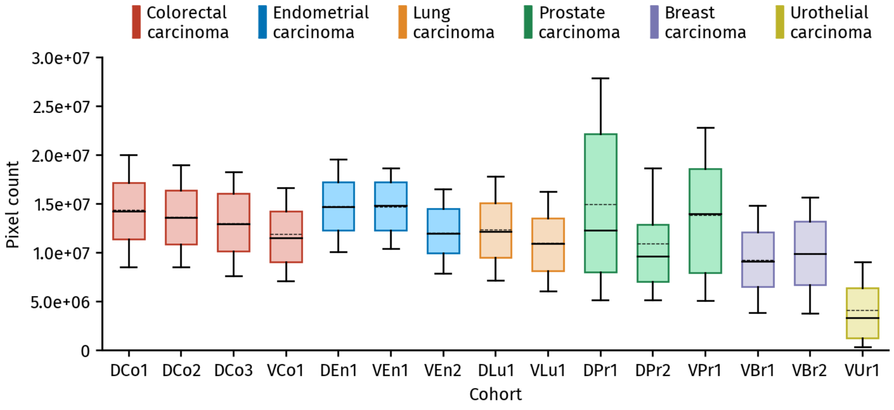

(b) Foreground area

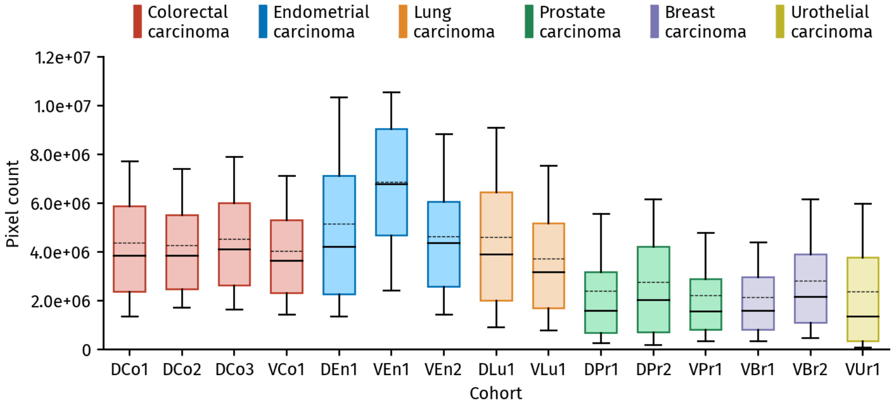

(c) Annotation area

Figure S34: Area in number of pixels at resolution 5 µm per pixel in NanoZoomer XR scans. “Foreground” is foreground without annotation and “Annotation” is foreground with annotation. Background exclusion masks are applied on all images. Note the difference in vertical axis range between subplots.

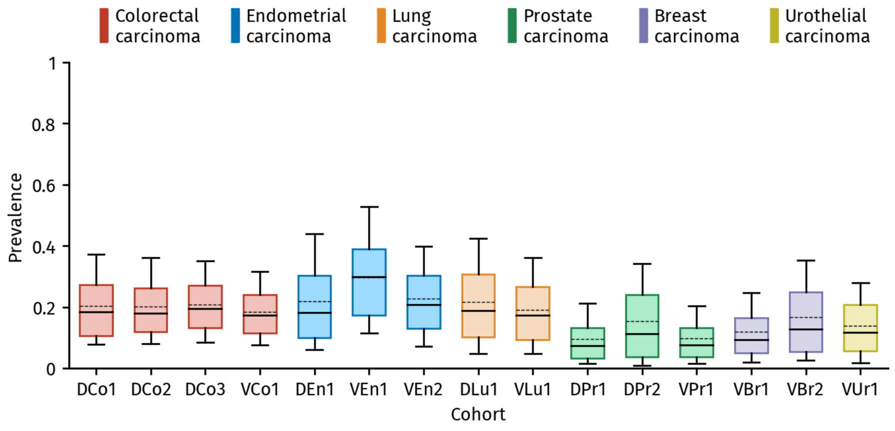

(a) Annotation prevalence in image

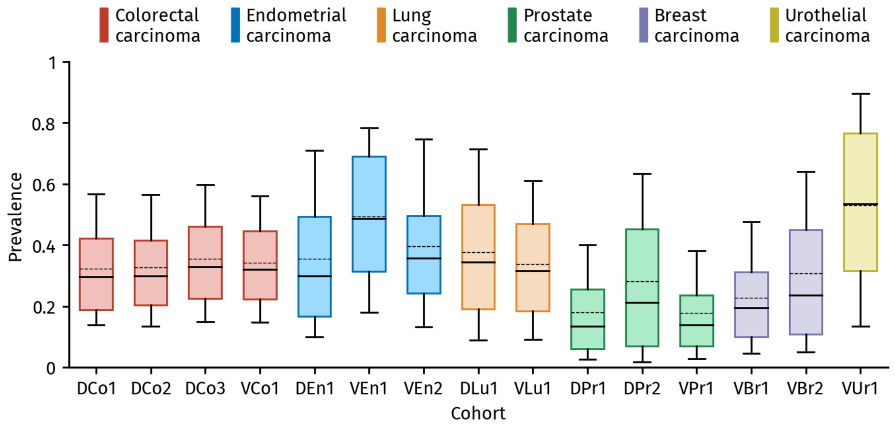

(b) Annotation prevalence in foreground

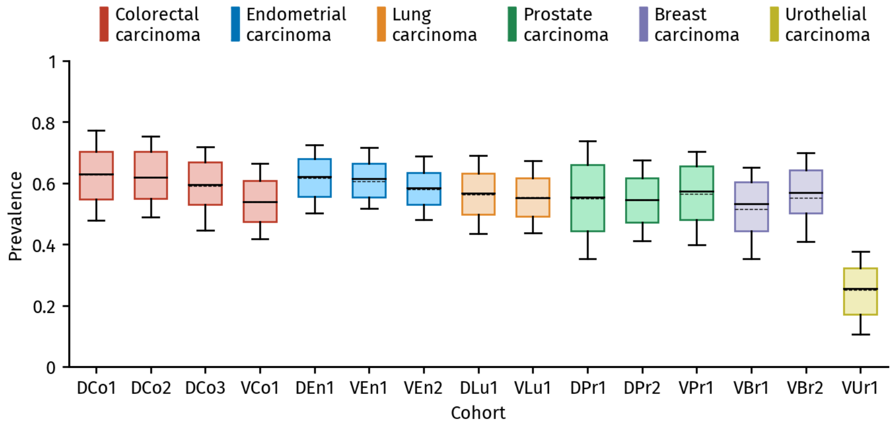

(c) Foreground prevalence in image

Figure S35: Prevalence at resolution 5 µm per pixel in Aperio AT2 scans. "Foreground" is foreground without annotation and "Annotation" is foreground with annotation. Background exclusion masks are applied on all images.

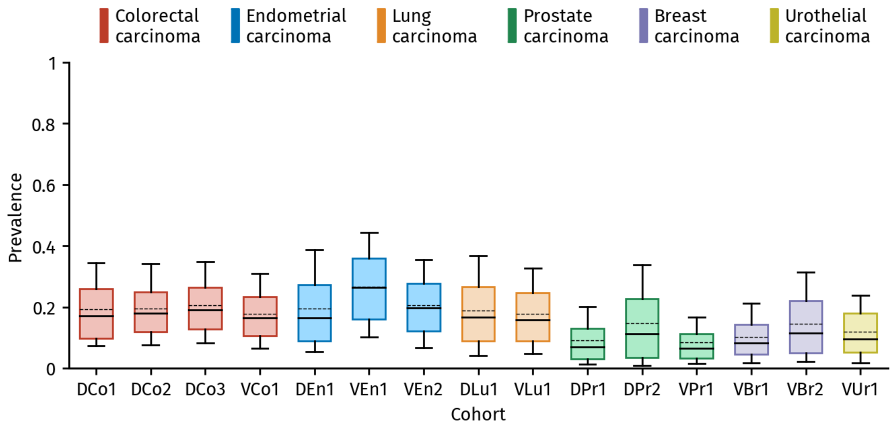

(a) Annotation prevalence in image

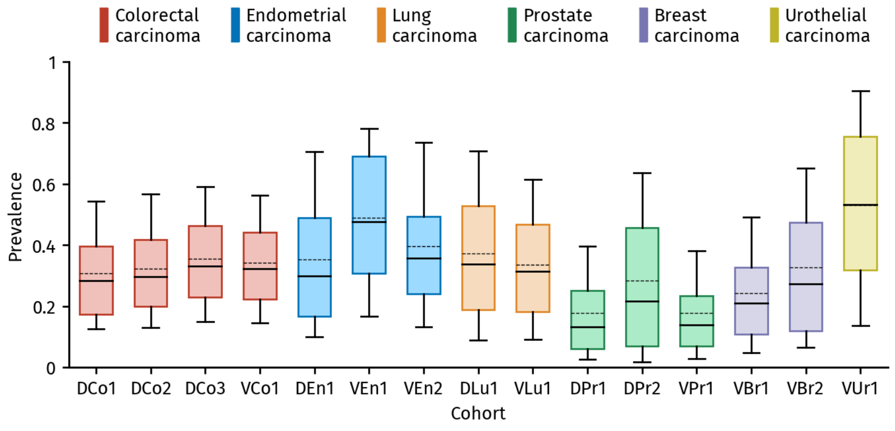

(b) Annotation prevalence in foreground

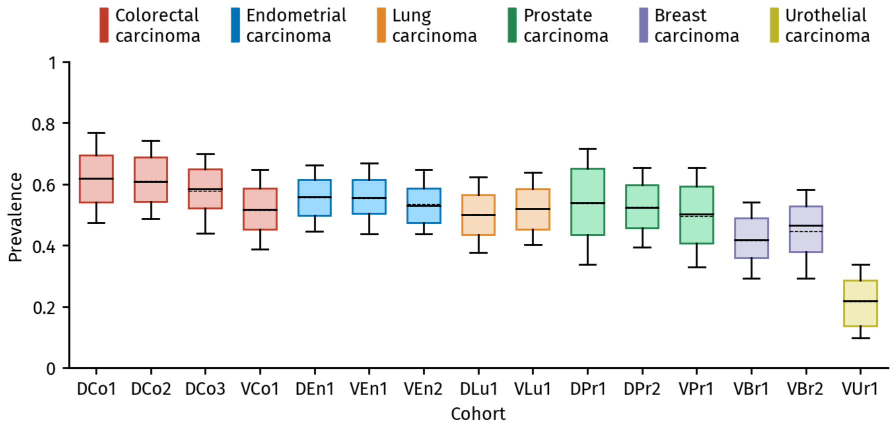

(c) Foreground prevalence in image

Figure S36: Prevalence at resolution 5 μm per pixel in NanoZoomer XR scans. “Foreground” is foreground without annotation and “Annotation” is foreground with annotation. Background exclusion masks are applied on all images.

References

1. Liu, J. *et al.* An integrated TCGA pan-cancer clinical data resource to drive high-quality survival outcome analytics. *Cell* **173,** 400–416 (2018).

6 Study protocol

Protocol for external validation of a pan cancer deep learning method for automatic tumour segmentation in digital histology images

This protocol was last modified March 24, 2023, prior to all investigations that could reveal associations between the predicted and target segmentation masks in the method validation cohorts. At that time the method validation cohorts had been scanned, annotated and tiled; baseline characteristics, colour statistics and annotation statistics had been computed; the neural network part of all methods listed in the primary and secondary analyses had been applied on all tiles, but no further processing had been performed. All of the above interactions with the validation cohorts were performed blindly and did not inform choices made in the method development.

Contents

1 Materials **1**
- 1.1 Materials for method development . . . 2
  - 1.1.1 DCo1 — Colorectal carcinoma . . . 2
  - 1.1.2 DCo2 — Colorectal carcinoma . . . 3
  - 1.1.3 DCo3 — Colorectal carcinoma . . . 4
  - 1.1.4 DEn1 — Endometrial carcinoma . . . 5
  - 1.1.5 DLu1 — Lung carcinoma . . . 7
  - 1.1.6 DPr1 — Prostate carcinoma . . . 7
  - 1.1.7 DPr2 — Prostate carcinoma . . . 8
- 1.2 Materials for method validation . . . 10
  - 1.2.1 VCo1 — Colorectal carcinoma . . . 10
  - 1.2.2 VEn1 — Endometrial carcinoma . . . 11
  - 1.2.3 VEn2 — Endometrial carcinoma . . . 12
  - 1.2.4 VLu1 — Lung carcinoma . . . 13
  - 1.2.5 VPr1 — Prostate carcinoma . . . 13
  - 1.2.6 VBr1 — Breast carcinoma . . . 14
  - 1.2.7 VBr2 — Breast carcinoma . . . 15
  - 1.2.8 VUr1 — Urothelial carcinoma . . . 16
- 1.3 Material analyses . . . 17
  - 1.3.1 Baseline characteristics . . . 17
  - 1.3.2 Survival characteristics . . . 23
  - 1.3.3 Colour statistics . . . 29

2 Methods **33**
- 2.1 Programming environment . . . 33
- 2.2 Method development . . . 33
  - 2.2.1 Downsampling . . . 34
  - 2.2.2 Tiling . . . 34
  - 2.2.3 Dataset balancing . . . 36
  - 2.2.4 Background segmentation . . . 36
  - 2.2.5 Background tile exclusion . . . 43
  - 2.2.6 Dataset augmentations . . . 45

2.2.7 Image value standardisation . . . 45
2.2.8 Segmentation network . . . 49
2.2.9 Network optimisation . . . 50
2.3 Method application . . . 51
2.3.1 Downsampling and tiling . . . 51
2.3.2 Neural network . . . 51
2.3.3 Reconstruction from tiles . . . 52
2.3.4 Result post-processing . . . 53
2.4 Performance evaluation . . . 54
2.4.1 Overlap counting . . . 54

3 Analyses **57**
3.1 Primary analysis . . . 57
3.2 Secondary analyses . . . 57
3.2.1 Different performance evaluation metrics of primary result . . . 57
3.2.2 Primary result on scans from the NanoZoomer XR . . . 57
3.2.3 Single cancer type training . . . 57
3.2.4 Primary analysis replication . . . 58

List of Figures

1 Acquisition overview DCo1 3
2 Acquisition overview DCo2 3
3 Acquisition overview DCo3 4
4 Acquisition overview DEn1 6
5 Acquisition overview DLu1 7
6 Acquisition overview DPr1 8
7 Acquisition overview DPr2 9
8 Acquisition overview VCo1 11
9 Acquisition overview VEn1 12
10 Acquisition overview VEn2 12
11 Acquisition overview VLu1 13
12 Acquisition overview VPr1 14
13 Acquisition overview VBr1 15
14 Acquisition overview VBr2 15
15 Acquisition overview VUr1 16
16 Kaplan-Meier analysis in materials from colorectal carcinoma 23
17 Kaplan-Meier analysis in materials from endometrial carcinoma 24
18 Kaplan-Meier analysis in materials from lung carcinoma 25
19 Kaplan-Meier analysis in materials from prostate carcinoma 26
20 Kaplan-Meier analysis in materials from urothelial carcinoma 27
21 Kaplan-Meier analysis in materials from urothelial carcinoma 28
22 Scan colour mean value Aperio AT2 29
23 Scan colour mean value NanoZoomer XR 30
24 Scan colour standard deviation Aperio AT2 31
25 Scan colour standard deviation NanoZoomer XR 32
26 Background exclusion example 38
27 Scan area Aperio AT2 39
28 Scan area NanoZoomer XR 40
29 Scan content prevalence Aperio AT2 41
30 Scan content prevalence NanoZoomer XR 42
31 Background exclusion statistics 44
32 Tile colour mean value 47
33 Tile colour standard deviation 48
34 Segmentation network architecture 49
35 Step length schedule 51
36 Tiling example 52
37 Overlap weight tiles 53

List of Tables

1 Data count in development cohorts . . . 2
2 Data count in validation cohorts . . . 10
3 Baseline characteristics in materials from colorectal carcinoma . . . 17
4 Baseline characteristics in materials from endometrial carcinoma . . . 18
5 Baseline characteristics in materials from lung carcinoma . . . 19
6 Baseline characteristics in materials from prostate carcinoma . . . 20
7 Baseline characteristics in materials from breast carcinoma . . . 21
8 Baseline characteristics in materials from urothelial carcinoma . . . 22
9 Python packages . . . 33
10 Annotated scan count after balancing . . . 36
11 Development set tile count . . . 43
12 Colour statistics in tiles from development set . . . 46
13 Confusion matrix . . . 55

1 Materials

Images analysed in this study are images of thin slices of resected solid tumours. Details about how these images are acquired follows below, before a per-cohort characterisation is presented.

The mass of interest is surgically removed from the patient and placed in containers with formalin for fixation. Pathologists examine the formalin-fixed specimen and regions of interest are cut into blocks and embedded in paraffin. The formalin-fixed paraffin-embedded (FFPE) blocks are sliced into thin tissue sections using a microtome and mounted on slides. Unless otherwise specified, the sections used in this study have thickness 3 µm. The tissue section is then stained with conventional haematoxylin and eosin (H&E), prepared as a tissue slide and imaged with a microscope scanner to form a so-called whole slide image (WSI).

This scan is then examined by a pathologist, and the tumour area, if any, is delineated. When the tissue slide is scanned by multiple different scanners, the digital tumour annotation is usually created on a scan from one scanner and digitally transformed to match the corresponding scan from different scanners. In these cases the transferred tumour annotation is verified by a pathologist, who corrects the annotation when necessary. If the transfer fails, the destination scan is manually annotated. To transform the annotation from the source scan to the destination scan, we first downsample the scans by a factor of 32. Then an image registration from the source image to the destination image is computed using a scale-invariant feature transform (SIFT).[1] This transformation is then used to transfer the polygons of the source annotation to the destination image.

In some of the cohorts, FFPE blocks are received at the Institute for Cancer Genetics and Informatics (ICGI), Oslo University Hospital, Norway, and sectioned, stained, scanned, and annotated by laboratory technicians. For other cohorts, we receive H&E stained tissue slides which we scan. In the remaining cohorts we receive digital scan files. What kind of material we received for each particular cohort studied will be specified in the cohort description (section 1.1 and section 1.2).

In this study, we use two scanners; the Aperio AT2 (Leica Biosystems, Germany) and the NanoZoomer XR (Hamamatsu Photonics, Japan). Digital scan files are read using the *Python* interface of the *OpenSlide C* library version 3.4.1.[2]

All cohorts are presented in section 1.1 and section 1.2 for method development and validation cohorts, respectively. Further descriptive analyses of the included materials are presented in section 1.3.

It should be noted that the materials were chiefly acquired for other projects, and not this seg-

mentation study. The exclusion reasons are therefore, in general, not consistent between materials. Also, some exclusion reasons would perhaps not have been applied if the material were acquired specifically for tumour segmentation.

Unless otherwise specified, all included scans in this study were manually annotated by pathologist Manohar Pradhan (MP) with over fifteen years of experience at the time the first cohorts in this study were annotated.

1.1 Materials for method development

20 270 scans from two scanners and 4 305 patients from 7 different cohorts were used in developing the tumour segmentation method; 3 from colorectal carcinoma, 1 from endometrial carcinoma, 1 from lung carcinoma and 2 from prostate carcinoma (protocol table 1).

Protocol Table 1: Number of patients, annotated scans and tiles in the development cohorts. Scans are from two different scanners. Tiles are produced at resolution 1 µm per pixel and have a size of 2 048 × 2 048 pixels.

Cancer type	Cohort	Patients	Scans		Tiles	
			Aperio AT2	NanoZoomer XR	Aperio AT2	NanoZoomer XR
Colorectal carcinoma	DCo1	206	206	206	28 911	30 552
	DCo2	578	578	575	81 651	82 294
	DCo3	765	765	764	108 429	109 720
	Sum	1 549	1 549	1 545	218 991	222 566
Endometrial carcinoma	DEn1	1 241	3 340	3 340	514 341	566 105
Lung carcinoma	DLu1	933	3 519	3 519	492 150	551 767
Prostate carcinoma	DPr1	328	976	976	158 001	163 527
	DPr2	254	753	753	95 015	97 867
	Sum	582	1 729	1 729	253 016	261 394
Sum		4 305	10 137	10 133	1 478 498	1 601 832

1.1.1 DCo1 — Colorectal carcinoma

224 patients with colonic adenocarcinoma and adjacent normal mucosa treated between 1988 and 2000 at Akershus University Hospital, Norway.[3, 4] Tissue blocks were received at ICGI, prepared as tissue slides and scanned. After exclusions, (see protocol figure 1) 206 annotated scans remained from both the Aperio AT2 the NanoZoomer XR scanner.

Scans from Aperio AT2 were manually annotated for tumour by a pathologist (MP), and these tumour annotations were transferred to the corresponding NanoZoomer XR scans.

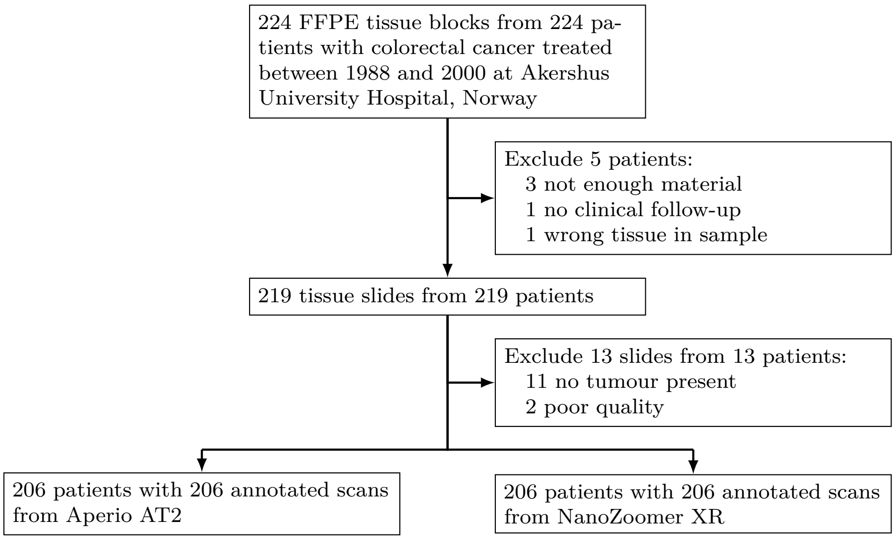

Protocol Figure 1: Flow from received blocks to annotated scans for the DCo1 cohort

1.1.2 DCo2 — Colorectal carcinoma

The material origin are patients with colorectal cancer treated between 1993 and 2003 at Aker Hospital (now part of Oslo University Hospital), Norway. 578 patients with resected tissue section and stages I, II, and III colorectal cancer that were analysed in previous studies were included.[4, 5, 6] One tissue slide per patient was prepared at ICGI, and scanned with the Aperio AT2 and NanoZoomer XR scanners. Three slides that had already been scanned with the Aperio AT2 scanner could not be scanned with the NanoZoomer XR scanner because of damaged cover glass. This resulted in 578 annotated scans from the Aperio AT2 scanner and 575 scans from the NanoZoomer XR scanner (summarised in protocol figure 2).

Scans from Aperio AT2 were manually annotated for tumour by a pathologist (MP), and these tumour annotations were transferred to the corresponding NanoZoomer XR scans.

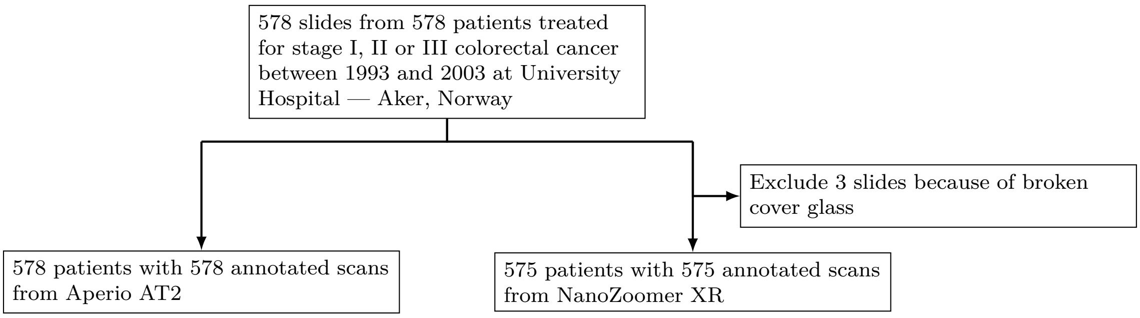

Protocol Figure 2: Flow from inclusions in previous studies (reference [5, 6]) to annotated scans for the DCo2 cohort

1.1.3 DCo3 — Colorectal carcinoma

2327 patients with histologically proven stage II or III colorectal cancer and resected primary tumour recruited from 151 hospitals in the United Kingdom between 2002 and 2004 for the *VICTOR* trial and randomly assigned to receive either rofecoxib (1167 patients) or placebo (1160 patients).[7] From 795 patients that we have included in a previous study, 795 H&E stained tissue sections were obtained at ICGI.[4] Some tissue sections where sectioned from FFPE blocks at ICGI, and some elsewhere. After exclusions as in reference [4] we had 768 scans from the Aperio AT2 scanner and 768 scans from the NanoZoomer XR scanner (see protocol figure 3). Two additional patients were excluded since their slides were of poor quality, and the scans of the new sections did not have manual annotations. One additional slide was also excluded because of no presence of tumour in the tissue section. A final scan from the NanoZoomer XR scanner was excluded since the annotation did not match the scan.

In this cohort, scans from NanoZoomer XR were manually annotated for tumour by a pathologist (MP) and these annotations were transferred to the corresponding Aperio AT2 scans.

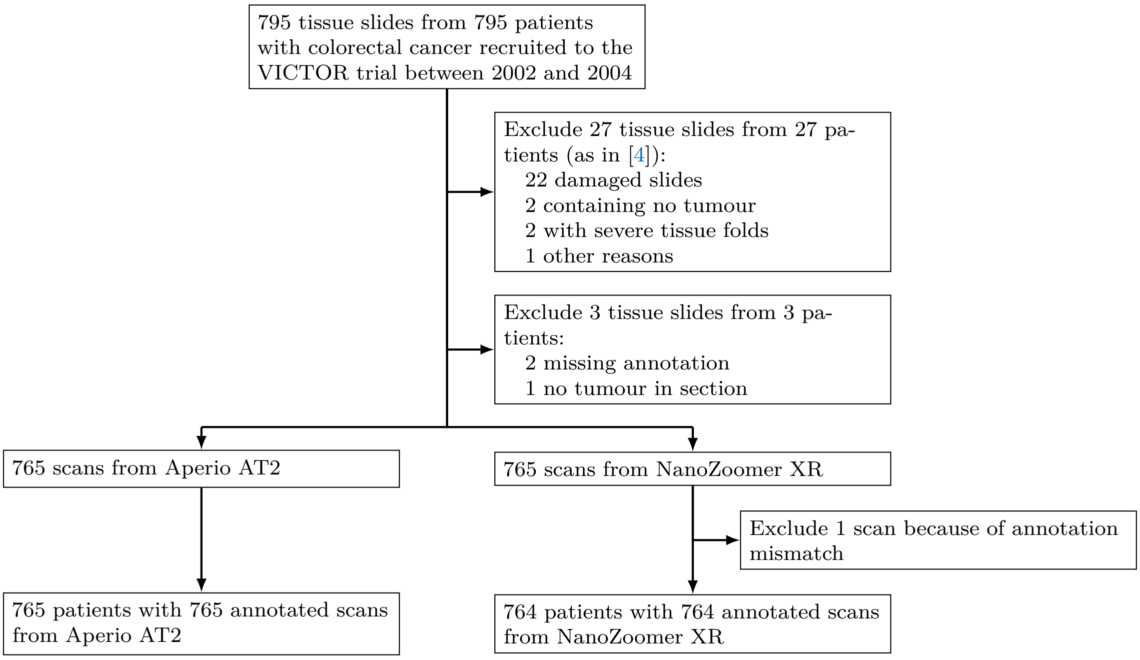

Protocol Figure 3: Flow from received slides to annotated scans for the DCo3 cohort

1.1.4 DEn1 — Endometrial carcinoma

We considered 1795 patients who underwent surgery for endometrial carcinoma between 2006 and 2018 at Oslo University Hospital, Norway.

We considered 1795 patients referred to the Department of Gynecological Oncology at Oslo University Hospital (OUH), Norway, and diagnosed *or* operated for endometrial carcinoma at OUH between 2006 and 2017. This material originates from the MoMaTEC (Molecular Markers in Treatment of Endometrial Cancer) trial (NCT number NCT00598845).[8, 9]

Note that among the 1795 patients, 11 patients (with 28 FFPE tumour blocks) had neuroendocrine tumours, which was removed from the World Health Organization classification of endometrial carcinoma between the 2014 edition and the 2020 edition.[10, 11]

FFPE blocks were collected by ICGI and prepared as tissue slides. Large sections were split and placed on two slides. After exclusions (see protocol figure 4), there remained 1229 patients with 4760 FFPE tumour blocks. At most three FFPE blocks were randomly selected from each patient, except for patients with tumours with mixed histology where all blocks were selected. This reduced the number of included blocks to 3331. 11 sections from 11 blocks were too large for a single slide and were placed on two slides. With this we have 1241 patients and 3331 blocks with 3340 annotated scans from Aperio AT2 and NanoZoomer XR.

After an update of the source of this material (after training but before validation), the following inconsistencies were noticed: 2 patients with 3 blocks each should have been excluded due to previous irradiation to a pelvic field including the uterus, 2 patients with 3 blocks each should have been excluded due to neoadjuvant treatment, 1 patient with 3 blocks was wrongly excluded due to complications after surgery. Protocol Figure 4 describe the material that was used in this study.

Scans from the NanoZoomer XR scanner were manually annotated for tumour by a pathologist (MP) and automatically transferred to the corresponding scans from the Aperio AT2 scanner.

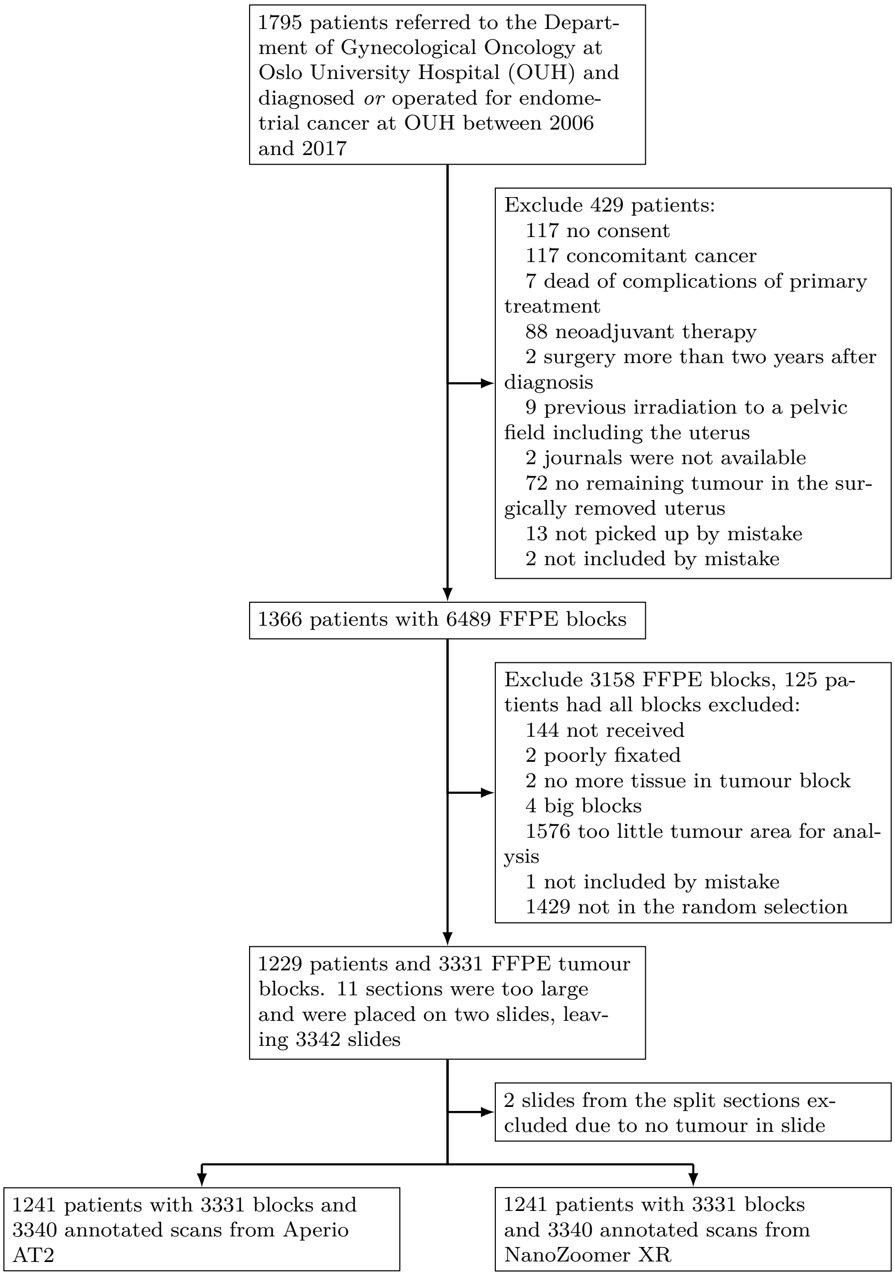

Protocol Figure 4: Flow from operated patients to annotated scans for the DEn1 cohort

1.1.5 DLu1 — Lung carcinoma

967 patients resected for primary lung cancer as part of primary treatment between March 2006 and December 2018 at Oslo University Hospital, Norway. FFPE tissue blocks were requested from all but 5 excluded patients (see protocol figure 5) from pathology departments at Oslo University Hospital. 3519 H&E slides were successfully prepared at ICGI from 3519 FFPE tissue blocks from 933 patients.

Scans from the NanoZoomer XR scanner were manually annotated by a pathologist (MP) and trained laboratory technician Jonathan Gullesen (JG). Annotations were verified and corrected if needed by a pathologist (MP). Large areas of fibrous tissue and necrosis were not included in the annotation. The manual annotations were automatically transferred to the corresponding Aperio AT2 scans.

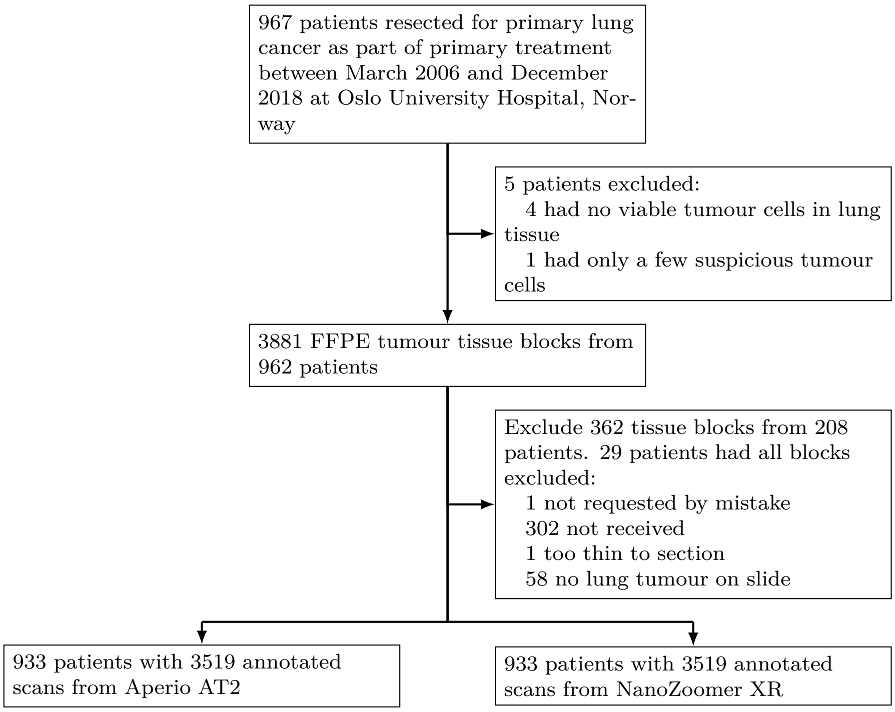

Protocol Figure 5: Flow from eligible patients to annotated scans for the DLu1 cohort

1.1.6 DPr1 — Prostate carcinoma

The cohort comprised 389 patients who underwent radical prostatectomy (RP) between 1999 and 2010 at Vestfold Hospital Trust, Norway. 61 patients were excluded: 26 for missing FFPE blocks, 6 for no tumour material and 29 patients for failing one or more FFPE block selection criteria. The

criteria were: one block with the highest Gleason score, a second block with the largest tumour area, a third block selected randomly from the remaining blocks with a tumour area $> 16\,\mathrm{mm}^2$ on a diagnostic H&E section.[12] At scan preparation, 7 blocks were excluded, and a further slide was excluded since this slide was missing when Aperio scanning was performed, leaving us with 976 annotated scans from both Aperio AT2 and NanoZoomer XR originating from 328 patients (summarised in protocol figure 6).

Tumour areas in scans from Aperio AT2 were manually annotated by a pathologist (MP) and large benign areas were avoided. These manual annotations were automatically transferred to NanoZoomer XR scans.

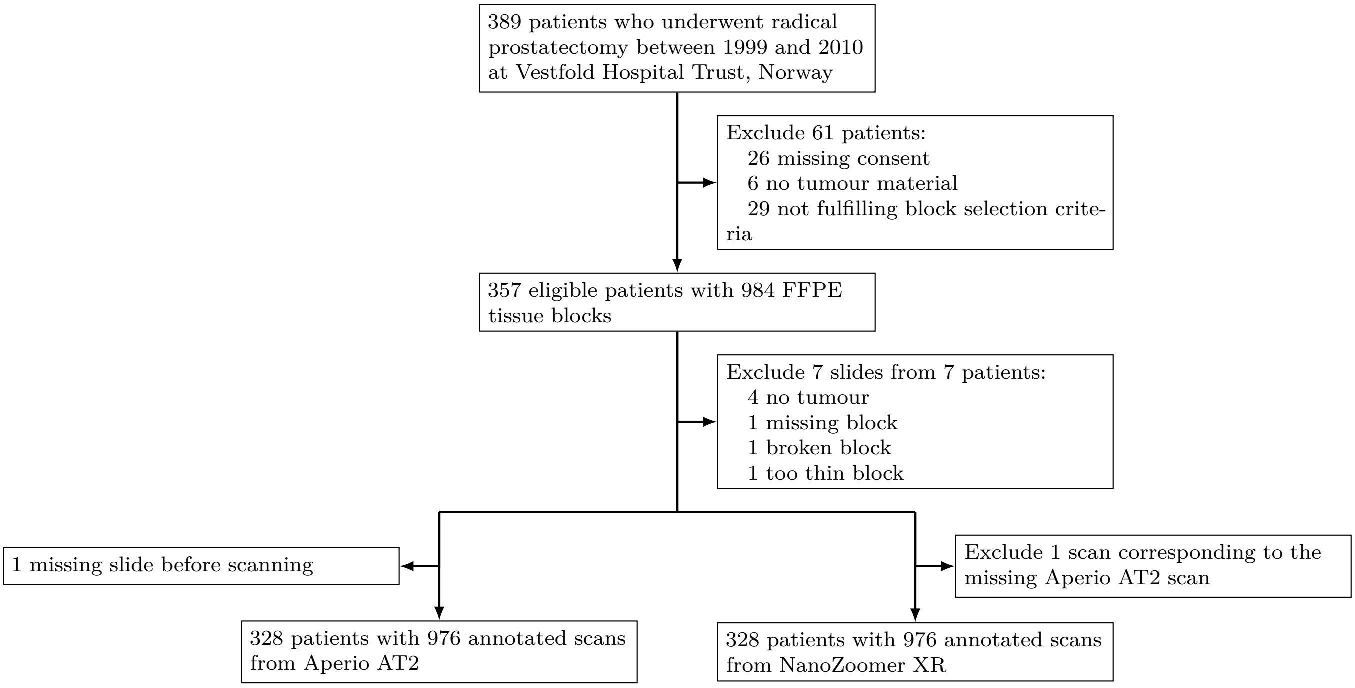

Protocol Figure 6: Flow from operated patients to annotated scans for the DPr1 cohort

1.1.7 DPr2 — Prostate carcinoma

The cohort originally comprised 317 patients who underwent RP between 1987 and 2005 at the Norwegian Radium Hospital (now part of Oslo University Hospital), Norway. Inclusion criteria for RP were preoperative absence of known metastases, age less than 75 years and life expectancy of at least 10 years. Adjuvant therapy was started in cases with elevated PSA after surgery and/or metastatic disease. All patients were operated by one surgeon (Håkon Wæhre).[13]

After exclusions (see protocol figure 7) there remained 307 eligible patients, of which 255 patients had three available tumour-containing blocks and were therefore included for further analyses. The assessment was based on the highest Gleason sum and/or previously assessed non-diploid DNA ploidy status, that had tumour areas measuring $> 4\,\mathrm{mm}^2$ on a diagnostic H&E section.[14] After further exclusions, we had 753 annotated scans from 254 patients (245 patients with 3 scans each and 9 patients with 2 scans each) from both the Aperio AT2 scanner and the NanoZoomer XR scanner.

Tumour areas were manually annotated avoiding large benign areas. 50% of the Aperio AT2 scans were manually annotated by a pathologist (MP) and automatically transferred to the corresponding NanoZoomer XR scans. The other 50% of the NanoZoomer XR scans were manually annotated by a laboratory technician (JG). Annotations were verified and corrected if needed by a pathologist (MP). These annotations were automatically transferred to the corresponding Aperio AT2 scans.

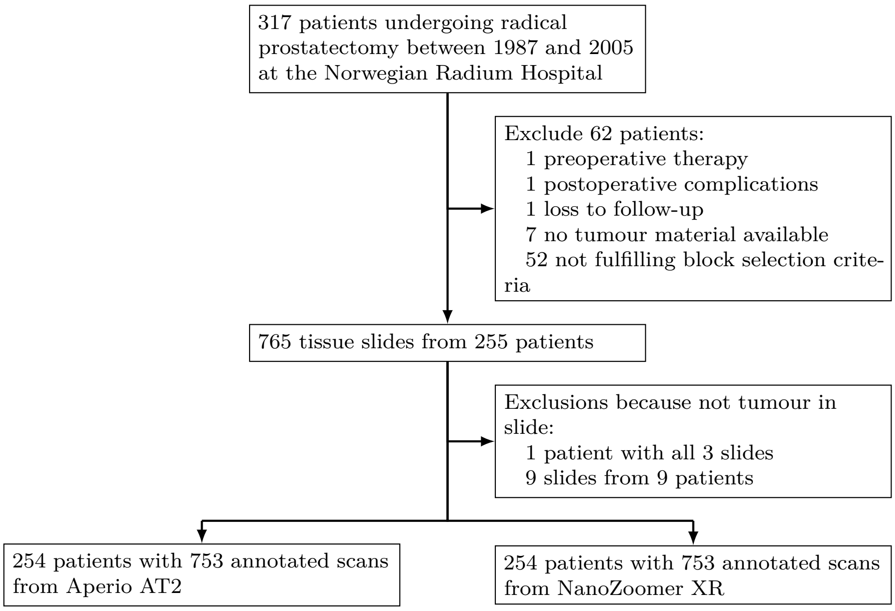

Protocol Figure 7: Flow from operated patients to annotated scans for the DPr2 cohort

1.2 Materials for method validation

7 258 scans from two scanners and 3 068 patients from 8 different cohorts were used in validating the tumour segmentation method; 1 from colorectal carcinoma, 2 from endometrial carcinoma, 1 from lung carcinoma, 1 from prostate carcinoma, 1 from bladder carcinoma and 2 from breast carcinoma (see protocol table 2).

All included scans from all validation cohorts were manually segmented prior to any investigation that could reveal correlations between predicted segmentation masks and manually segmented target masks. All validation cohorts were primarily manually segmented for purposes other than serving as validation cohorts for this project, and had already been manually segmented when this project was initiated. These manual segmentations did not inform the development of the presented segmentation method, nor did the development of the presented segmentation method inform the manual segmentation.

Protocol Table 2: Number of patients, annotated scans and tiles in the validation cohorts. Scans are from two different scanners. Tiles are produced at resolution 1 µm per pixel and have a size of 7 680 × 7 680 pixels

Cancer type	Cohort	Patients	Scans Aperio AT2	Scans NanoZoomer XR	Tiles Aperio AT2	Tiles NanoZoomer XR
Colorectal carcinoma	VCo1	1 132	1 155	1 155	17 686	18 635
Endometrial carcinoma	VEn1	77	77	77	1 279	1 408
	VEn2	132	152	152	2 179	2 383
	Sum	209	229	229	3 458	3 791
Lung carcinoma	VLu1	522	522	522	7 100	7 649
Prostate carcinoma	VPr1	259	777	777	12 568	1 4072
Breast carcinoma	VBr1	310	310	310	4 165	4 689
	VBr2	304	304	304	4 098	4 576
	Sum	614	614	614	8 263	9 265
Urothelial carcinoma	VUr1	332	332	332	3 446	3 814
Sum		3 068	3 629	3 629	52 521	57 226

1.2.1 VCo1 — Colorectal carcinoma

This cohort comprises participants in the QUASAR 2 (QUick And Simple And Reliable) trial (ISRCTN registry number ISRCTN45133151). Between 2005 and 2010, 1952 eligible patients were enrolled from 170 hospitals in seven countries (Australia, Austria, Czeck Republic, New Zealand,

Serbia, Slovenia, and the UK). The trial investigated the effect of bevacizumab on disease-free survival after potentially curative surgery of primary tumour.[15] FFPE tissue blocks were collected from 1 251 patients with either stage II or III colorectal cancer, of whom usable tissue blocks from 1 140 patients were received at ICGI.[4] After exclusions (see protocol figure 8) we had 1132 eligible patients with 1155 annotated scans from the Aperio AT2 scanner and the NanoZoomer XR scanner. Note that there were originally one section per patient, but some large sections had to be split before scanning in order to fit the cover slip.

Scans from both Aperio AT2 and NanoZoomer XR were manually annotated for tumour by a pathologist (MP).

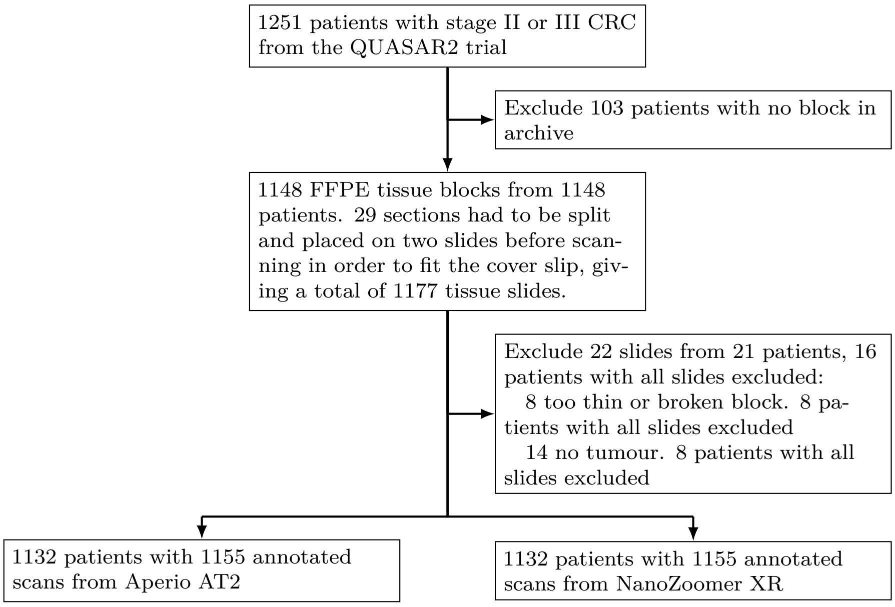

Protocol Figure 8: Flow from eligible patients to annotated scans for the VCo1 cohort

1.2.2 VEn1 — Endometrial carcinoma

95 blocks from 95 patients collected between 2001 and 2016 at Amsterdam Medical Center, The Netherlands. Six patients were excluded for clinical reasons, after which a further twelve blocks were excluded, leaving 77 patients with 77 annotated scans from both the Aperio AT2 and NanoZoomer XR scanner (see protocol figure 9).

Scans from the NanoZoomer XR scanner were manually annotated by a pathologist (MP) and transferred to the corresponding Aperio AT2 scans.

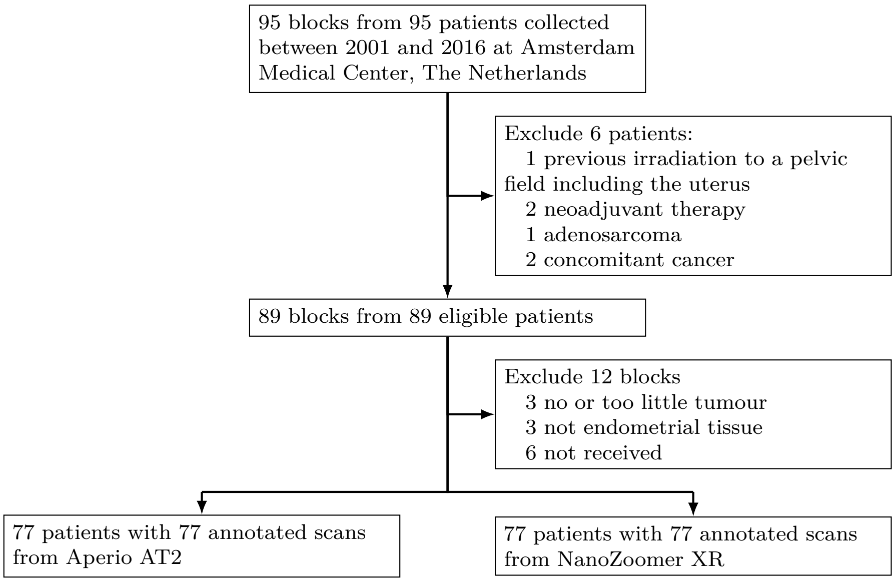

Protocol Figure 9: Flow from requested blocks to annotated scans for the VEn1 cohort

1.2.3 VEn2 — Endometrial carcinoma

177 blocks from 152 patients collected between 1999 and 2018 at Innsbruck Medical University, Austria. 19 blocks were excluded for no or too little tumour in the received block. 6 blocks were too thin to section. 14 patients had all blocks excluded for no or too little tumour, 4 patients had all blocks excluded for too thin block, and one additional patient had one block excluded for both reasons leaving no blocks left. This leaves 133 patients with 152 annotated scans from both the Aperio AT2 and NanoZoomer XR scanner (see protocol figure 10).

Scans from the NanoZoomer XR scanner were manually annotated by a pathologist (MP) and transferred to the corresponding Aperio AT2 scans.

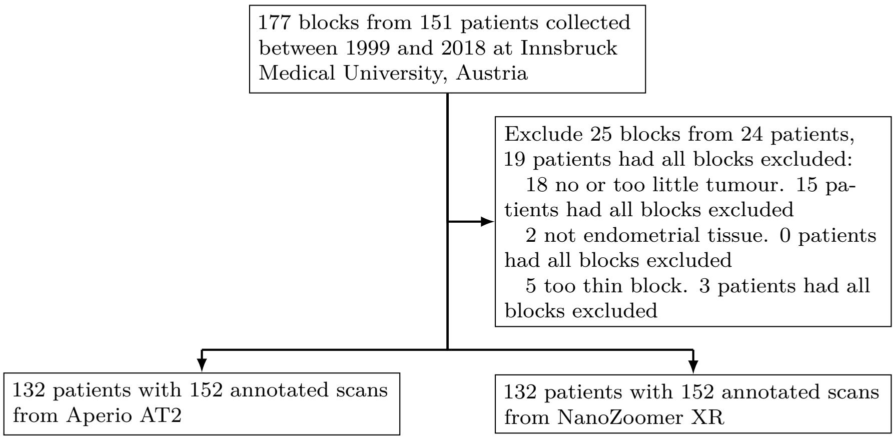

Protocol Figure 10: Flow from requested blocks to annotated scans for the VEn2 cohort

1.2.4 VLu1 — Lung carcinoma

A consecutive series of 633 patients with stage I to III non-small cell lung carcinoma (NSCLC) operated between 1990 and 2010 at the University Hospital of Northern Norway and Nordland Hospital, Norway.[16, 17] H&E stained tissue slides were received at ICGI for scanning, and after exclusions (see protocol figure 11) we had 522 eligible patients with 522 annotated scans from both the Aperio AT2 scanner and the NanoZoomer XR scanner.

NanoZoomer XR scans were manually annotated by a pathologist (MP), and they were automatically transferred to the corresponding Aperio AT2 scans. Large areas of fibrous tissue and necrosis were not annotated.

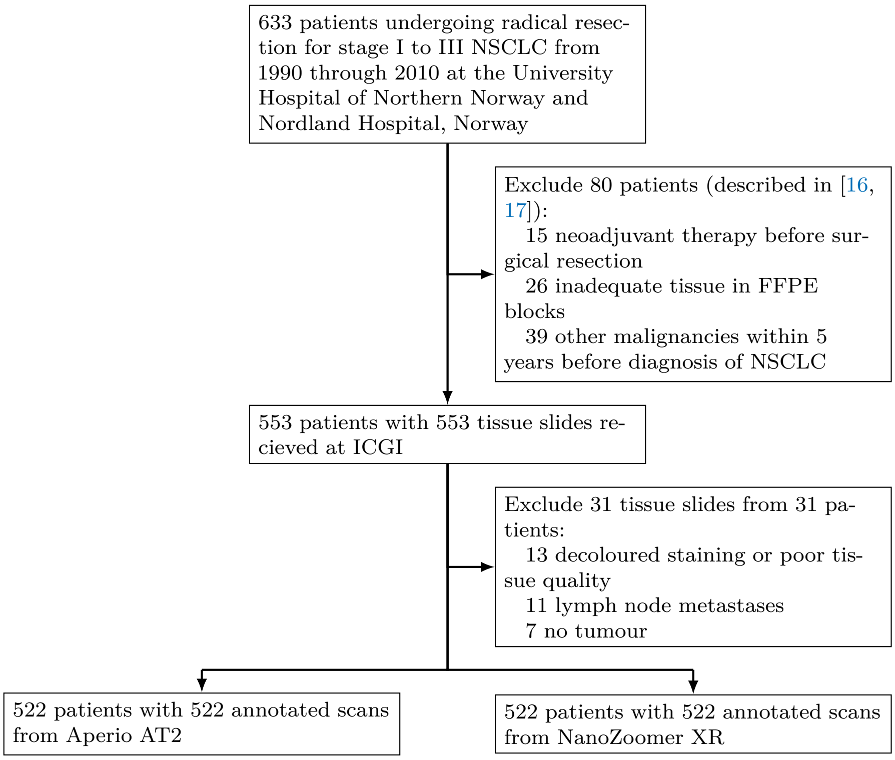

Protocol Figure 11: Flow from original study recruitment to annotated scans for the VLu1 cohort

1.2.5 VPr1 — Prostate carcinoma

The cohort comprised 287 patients who underwent RP between 2001 and 2006 at the Norwegian Radium Hospital (now a part of Oslo University Hospital), Norway. All patients were operated by one surgeon (Bjørn Brennhovd).[18]

After exclusions (see protocol figure 12), 259 eligible patients remained from which three blocks

were selected. The first and the second block represented the highest Gleason score and the largest tumour area, respectively. The third block was selected randomly from the remaining blocks with a tumour area $> 5\,\mathrm{mm}^2$ on a diagnostic H&E section.

We obtained three sections from all 259 eligible patients, resulting in 777 sections scanned on both the Aperio AT2 scanner and the NanoZoomer XR scanner.

The origin of the digital annotations are manual annotations made directly on the cover slip with a marker. These marked slides were scanned with a NanoZoomer HT (Hamamatsu Photonics, Japan) scanner. Digital annotations were generated on these scans by drawing inside the area delineated by the analog marker, this was done either manually or automatically. The resulting digital annotations were transferred to scans from the Aperio AT2 scanner before they were manually verified and potentially corrected by a pathologist (MP). Finally, the verified annotations were transferred to scans from the NanoZoomer XR scanner. Large areas of benign epithelium and stroma were not annotated.

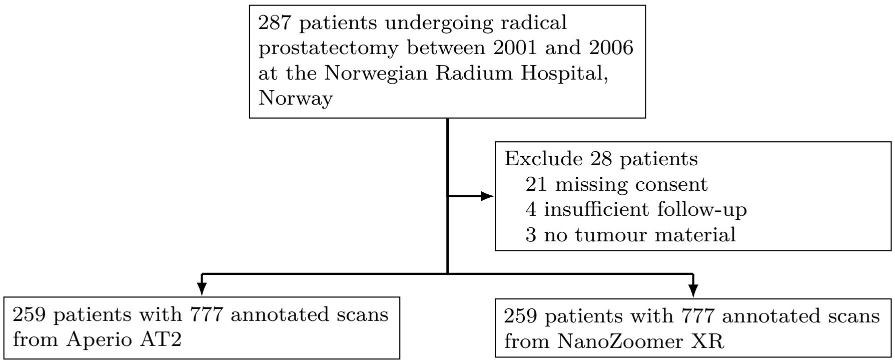

Protocol Figure 12: Flow from operated patients to annotated scans for the VPr1 cohort

1.2.6 VBr1 — Breast carcinoma

This cohort comprises 348 patients registered with breast cancer between 1990 and 1998 at Stavanger University Hospital, Norway.[19, 20, 21, 22] 320 H&E stained tissue sections prepared as slides from 320 patients were received at ICGI and scanned. After exclusions (see protocol figure 13) we were left with 310 slides from 310 patients scanned on both the Aperio AT2 scanner and the NanoZoomer XR scanner.

Scans from the Aperio AT2 scanner were manually annotated by a pathologist (MP) and transferred to the corresponding NanoZoomer XR scans. Both infiltrating tumour areas and intraductal carcinoma were annotated.

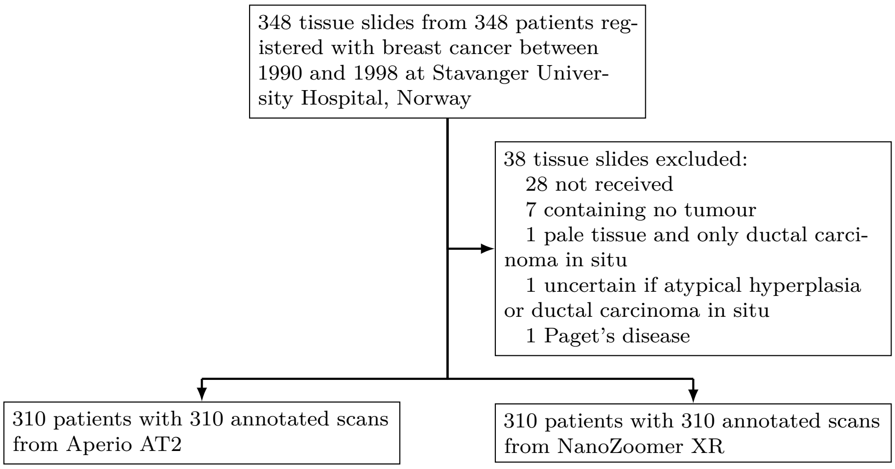

Protocol Figure 13: Flow from requested tissue slides to annotated scans for the VBr1 cohort

1.2.7 VBr2 — Breast carcinoma

This cohort comprises 339 patients registered with breast cancer between 2000 and 2004 at Stavanger University Hospital, Norway.[23] 315 H&E stained tissue sections prepared as slides from 315 patients were received at ICGI and scanned. After exclusions (see protocol figure 14) we were left with 304 slides from 304 patients scanned on both the Aperio AT2 scanner and the NanoZoomer XR scanner.

Scans from the Aperio AT2 scanner were manually annotated by a pathologist (MP), and annotations were transferred to the corresponding NanoZoomer XR scans. Both infiltrating tumour areas and intraductal carcinoma were annotated.

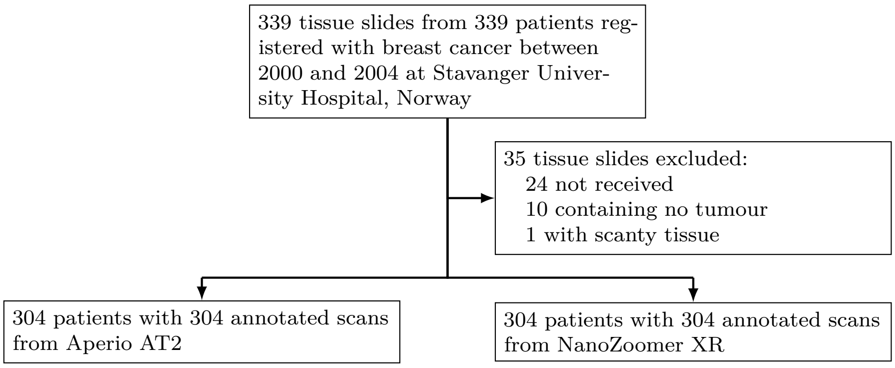

Protocol Figure 14: Flow from requested tissue slides to annotated scans for the VBr2 cohort

1.2.8 VUr1 — Urothelial carcinoma

357 patients diagnosed with urothelial carcinoma of the bladder between 1992 and 2010 at Stavanger University Hospital, Norway.[24, 25] From this, 333 slides from 333 patients were received at ICGI and scanned on the Aperio AT2 and the NanoZoomer XR scanner. 10 slides contained two parallel tissue sections, in which case only the best tissue section of the two parallels was scanned. One scan was excluded since it contained squamous epithelium and soft tissue but no tumour tissue, leaving us with 332 scans from 332 patients (see protocol figure 15).

All Aperio AT2 scans were manually annotated by a pathologist (MP), and the annotations were transferred to the NanoZoomer XR scanner. Urothelial tumours with or without infiltration were annotated.

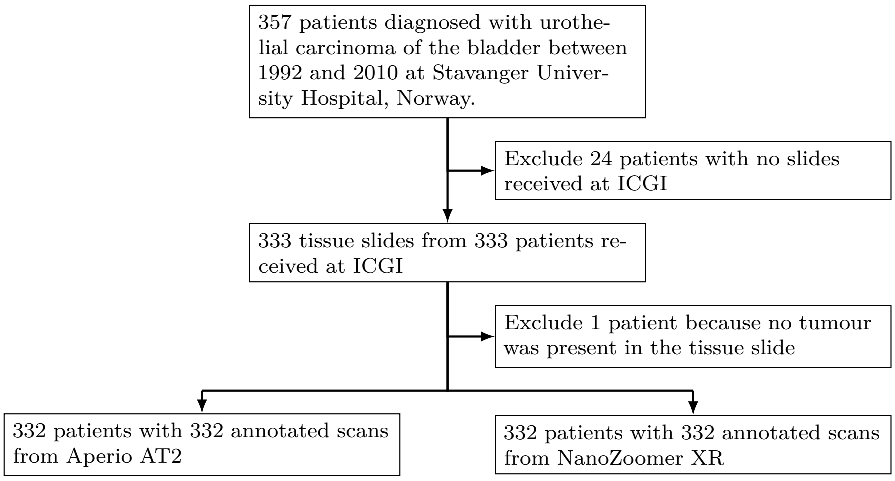

Protocol Figure 15: Flow from eligible patients to annotated scans for the VUr1 cohort

1.3 Material analyses

1.3.1 Baseline characteristics

Baseline characteristics for all cohorts grouped by cancer type are given in protocol tables 3 to 8. Data are given as *median (interquartile range)* or *count (percentage)*. Time to *event* statistics are based only on patients with the respective event. "Age" is age at a given starting point, which is the same starting point as time to event durations and follow-up time durations starts from. This starting point can vary between cohorts and will be specified.

Protocol Table 3: Baseline characteristics in colorectal carcinoma cohorts. Starting point is at randomisation for VCo1 and at surgery for the other cohorts.

	DCo1	DCo2	DCo3	VCo1
Patient count	206	578	765	1132
Age				
Years	71 (61 – 78)	73 (63 – 79)	64 (58 – 71)	65 (59 – 71)
Missing	2 (1%)	0	0	0
Sex				
Female	106 (51%)	290 (50%)	270 (35%)	480 (42%)
Male	98 (48%)	288 (50%)	495 (65%)	652 (58%)
Missing	2 (1%)	0	0	0
Cancer-specific death				
False	144 (70%)	377 (65%)	645 (84%)	961 (85%)
True	60 (29%)	201 (35%)	120 (16%)	157 (14%)
Missing	2 (1%)	0	0	14 (1%)
Time to cancer-specific death				
Years	0.7 (0.3 – 2.3)	2.8 (1.7 – 5.1)	3.0 (2.2 – 4.1)	2.7 (1.7 – 3.6)
Follow-up time				
Years	3.5 (1.0 – 5.0)	6.6 (2.9 – 9.4)	5.1 (4.1 – 5.8)	4.6 (3.3 – 5.1)
Missing	2 (1%)	0	0	0
pT stage				
pT1	2 (1%)	27 (5%)	12 (2%)	18 (2%)
pT2	24 (12%)	103 (18%)	52 (7%)	71 (6%)
pT3	164 (80%)	414 (72%)	527 (69%)	588 (52%)
pT4	13 (6%)	34 (6%)	150 (20%)	404 (36%)
Missing	3 (1%)	0	24 (3%)	51 (5%)
pN stage				
pN0	128 (62%)	388 (67%)	367 (48%)	406 (36%)
pN1	60 (29%)	152 (26%)	260 (34%)	515 (45%)
pN2	14 (7%)	37 (6%)	113 (15%)	185 (16%)
Missing	4 (2%)	1 (<1%)	25 (3%)	26 (2%)
Stage				
I	8 (4%)	112 (19%)	0	0
II	99 (48%)	277 (48%)	379 (50%)	406 (36%)
III	52 (25%)	189 (33%)	386 (50%)	726 (64%)
IV	45 (22%)	0	0	0
Missing	2 (1%)	0	0	0
Histological grade				
1	9 (4%)	58 (10%)	64 (8%)	46 (4%)
2	173 (84%)	452 (78%)	608 (79%)	855 (76%)
3	21 (10%)	63 (11%)	76 (10%)	173 (15%)
Missing	3 (1%)	5 (1%)	17 (2%)	58 (5%)

Protocol Table 4: Baseline characteristics in endometrial carcinoma cohorts. Starting point is at surgery.

	DEn1	VEn1	VEn2
Patient count	1241	77	132
Age			
Years	68 (61 – 76)	67 (60 – 73)	68 (61 – 75)
Missing	5 (<1%)	0	0
Histological type			
Adenosquamous carcinoma	0	2 (3%)	0
Carcinosarcoma	83 (7%)	17 (22%)	4 (3%)
Clear cell carcinoma	35 (3%)	14 (18%)	4 (3%)
Endometrioid carcinoma	825 (66%)	11 (14%)	98 (74%)
Mucinous carcinoma	7 (1%)	0	0
Neuroendocrine carcinoma	11 (1%)	0	0
Papillary serous carcinoma	133 (11%)	31 (40%)	24 (18%)
Squamous cell carcinoma	0	2 (3%)	0
Mixed with clear cell or papillary serous carcinoma	67 (5%)	0	0
Mixed without clear cell or papillary serous carcinoma	53 (4%)	0	0
Undifferentiated carcinoma	14 (1%)	0	2 (2%)
Unclassifiable	9 (1%)	0	0
Missing	4 (<1%)	0	0
Cancer-specific death			
False	974 (78%)	56 (73%)	115 (87%)
True	263 (21%)	21 (27%)	17 (13%)
Missing	4 (<1%)	0	0
Time to cancer-specific death			
Years	2.5 (1.4 – 4.2)	1.9 (1.3 – 3.4)	3.7 (2.7 – 5.8)
Overall death			
False	756 (61%)	41 (53%)	94 (71%)
True	481 (39%)	36 (47%)	38 (29%)
Missing	4 (<1%)	0	0
Time to overall death			
Years	3.7 (1.8 – 6.7)	2.0 (1.3 – 3.4)	5.0 (2.7 – 8.3)
Recurrence			
False	896 (72%)	54 (70%)	105 (80%)
True	341 (27%)	23 (30%)	27 (20%)
Missing	4 (<1%)	0	0
Time to recurrence			
Years	1.3 (0.7 – 2.4)	1.2 (1.0 – 1.5)	1.2 (0.8 – 2.6)
Follow-up time			
Years	7.6 (5.0 – 11.8)	3.6 (2.5 – 4.7)	4.5 (2.0 – 8.4)
Missing	4 (<1%)	0	0
FIGO stage			
1a	548 (44%)	20 (26%)	48 (36%)
1b	296 (24%)	12 (16%)	32 (24%)
2	79 (6%)	14 (18%)	19 (14%)
3	0	0	8 (6%)
3a	28 (2%)	1 (1%)	0
3b	12 (1%)	0	0
3c1	106 (9%)	18 (23%)	15 (11%)
3c2	72 (6%)	8 (10%)	9 (7%)
4	0	0	1 (1%)
4a	0	1 (1%)	0
4b	96 (8%)	3 (4%)	0
Missing	4 (<1%)	0	0
Histological grade			
1	467 (38%)	0	26 (20%)
2	286 (23%)	1 (1%)	64 (48%)
3	179 (14%)	75 (97%)	42 (32%)
Missing	309 (25%)	1 (1%)	0

Protocol Table 5: Baseline characteristics in lung carcinoma cohorts. Starting point is at surgery for DLu1 and at diagnosis for VLu1.

	DLu1	VLu1
Patient count	933	522
Age		
Years	68 (62 – 73)	68 (60 – 73)
Missing	11 (1%)	0
Sex		
Female	460 (49%)	168 (32%)
Male	462 (50%)	354 (68%)
Missing	11 (1%)	0
Histological type		
Adenocarcinoma	521 (56%)	226 (43%)
Adenosquamous carcinoma	16 (2%)	3 (1%)
Bronchioloalveolar carcinoma	8 (1%)	0
Carcinoid	42 (5%)	0
Large cell carcinoma	29 (3%)	0
Large cell neuroendocrine carcinoma	6 (1%)	0
Salivary gland type lung carcinoma	5 (1%)	0
Squamous cell carcinoma	287 (31%)	289 (55%)
Undifferentiated carcinoma	4 (<1%)	3 (1%)
Mixed	2 (<1%)	0
Other	2 (<1%)	1 (<1%)
Missing	11 (1%)	0
Cancer-specific death		
False	635 (68%)	316 (61%)
True	287 (31%)	206 (39%)
Missing	11 (1%)	0
Time to cancer-specific death		
Years	2.2 (1.2 – 3.7)	1.7 (0.9 – 3.2)
Follow-up time		
Years	4.6 (2.5 – 6.8)	3.6 (1.4 – 7.4)
Missing	11 (1%)	0
pT stage		
pT1	323 (35%)	171 (33%)
pT2	433 (46%)	196 (38%)
pT3	137 (15%)	98 (19%)
pT4	29 (3%)	57 (11%)
Missing	11 (1%)	0
pN stage		
pN0	671 (72%)	366 (70%)
pN1	184 (20%)	102 (20%)
pN2	67 (7%)	54 (10%)
Missing	11 (1%)	0
Stage		
I	507 (54%)	224 (43%)
II	273 (29%)	170 (33%)
III	132 (14%)	128 (25%)
IV	10 (1%)	0
Missing	11 (1%)	0

Protocol Table 6: Baseline characteristics in prostate carcinoma cohorts. Starting point is at surgery.

	DPr1	DPr2	VPr1
Patient count	328	254	259
Age			
Years	64 (61 – 68)	62 (58 – 67)	62 (59 – 66)
Missing	0	1 (<1%)	0
Overall death			
False	257 (78%)	176 (69%)	200 (77%)
True	71 (22%)	77 (30%)	59 (23%)
Missing	0	1 (<1%)	0
Time to overall death			
Years	8.6 (6.3 – 12.3)	9.6 (5.7 – 12.8)	9.8 (7.2 – 13.5)
Biochemical recurrence			
False	215 (66%)	98 (39%)	188 (73%)
True	113 (34%)	155 (61%)	71 (27%)
Missing	0	1 (<1%)	0
Time to biochemical recurrence			
Years	0.8 (0.0 – 3.9)	2.9 (1.5 – 5.3)	3.4 (1.2 – 5.6)
Follow-up time			
Years	11.0 (8.8 – 13.9)	11.2 (7.7 – 14.4)	9.6 (8.5 – 12.3)
Missing	0	1 (<1%)	0
pT stage			
pT2	178 (54%)	54 (21%)	159 (61%)
pT3	136 (41%)	168 (66%)	96 (37%)
pT4	0	26 (10%)	2 (1%)
pTx	14 (4%)	4 (2%)	0
Missing	0	2 (1%)	2 (1%)
Gleason grade			
2+3	0	0	3 (1%)
3+3	63 (19%)	11 (4%)	126 (49%)
3+4	147 (45%)	92 (36%)	83 (32%)
3+5	1 (<1%)	3 (1%)	0
4+3	88 (27%)	77 (30%)	25 (10%)
4+4	13 (4%)	41 (16%)	15 (6%)
4+5	4 (1%)	26 (10%)	1 (<1%)
5+4	1 (<1%)	3 (1%)	2 (1%)
5+5	0	0	1 (<1%)
Missing	11 (3%)	1 (<1%)	3 (1%)

Protocol Table 7: Baseline characteristics in breast carcinoma cohorts. Starting point is at diagnosis.

	VBr1	VBr2
Patient count	310	304
Age		
Years	56 (50 – 64)	57 (47 – 64)
Histological Type		
Invasive ductal carcinoma	235 (76%)	284 (93%)
Invasive lobular carcinoma	20 (6%)	6 (2%)
Lobular carcinoma in citu	24 (8%)	0
Medullary carcinoma	5 (2%)	8 (3%)
Tubular carcinoma	12 (4%)	0
Other	13 (4%)	6 (2%)
Missing	1 (<1%)	0
Distant metastases		
False	255 (82%)	220 (72%)
True	55 (18%)	74 (24%)
Missing	0	10 (3%)
Time to distant metastases		
Years	5.3 (2.6 – 11.7)	1.5 (0.7 – 3.3)
Local recurrence		
False	279 (90%)	274 (90%)
True	31 (10%)	20 (7%)
Missing	0	10 (3%)
Time to local recurrence		
Years	9.3 (4.3 – 16.5)	8.3 (2.8 – 13.3)
Follow-up time distant metastases		
Years	14.9 (8.4 – 21.2)	12.4 (4.0 – 15.2)
Missing	0	10 (3%)
Follow-up time local recurrence		
Years	13.3 (6.2 – 21.0)	11.9 (3.8 – 15.1)
Missing	0	11 (4%)
Oestrogen recetor (ER)		
Negative	40 (13%)	153 (50%)
Borderline	11 (4%)	0
Positive	258 (83%)	146 (48%)
Missing	1 (<1%)	5 (2%)
Progesterone receptor (PR)		
Negative	60 (19%)	166 (55%)
Borderline	55 (18%)	2 (1%)
Positive	194 (63%)	98 (32%)
Missing	1 (<1%)	38 (12%)
Lymph node status		
Negative	310 (100%)	216 (71%)
Positive	0	53 (17%)
Missing	0	35 (12%)
Nottingham prognostic index		
3–5	109 (35%)	39 (13%)
6–7	134 (43%)	115 (38%)
8–9	64 (21%)	134 (44%)
Missing	3 (1%)	16 (5%)

Protocol Table 8: Baseline characteristics in urothelial carcinoma cohort. Starting point is at diagnosis.

	VUr1
Patient count	332
Age	
Years	72 (62 – 80)
Missing	2 (1%)
Sex	
Female	84 (25%)
Male	248 (75%)
Recurrence	
False	173 (52%)
True	159 (48%)
Time to recurrence	
Years	1.2 (0.6 – 2.0)
Stage progression	
False	307 (92%)
True	25 (8%)
Time to stage progression	
Years	2.0 (0.7 – 3.0)
Follow-up recurrence	
Years	5.8 (2.6 – 8.2)
Follow-up stage progression	
Years	7.2 (4.4 – 10.1)
pT stage	
pTa	255 (77%)
pTis	1 (<1%)
pT1	76 (23%)
Histological grade	
1	65 (20%)
2	155 (47%)
3	111 (33%)
Missing	1 (<1%)
Metastasis	
False	321 (97%)
True	11 (3%)
Multifocal	
False	197 (59%)
True	107 (32%)
Missing	28 (8%)

1.3.2 Survival characteristics

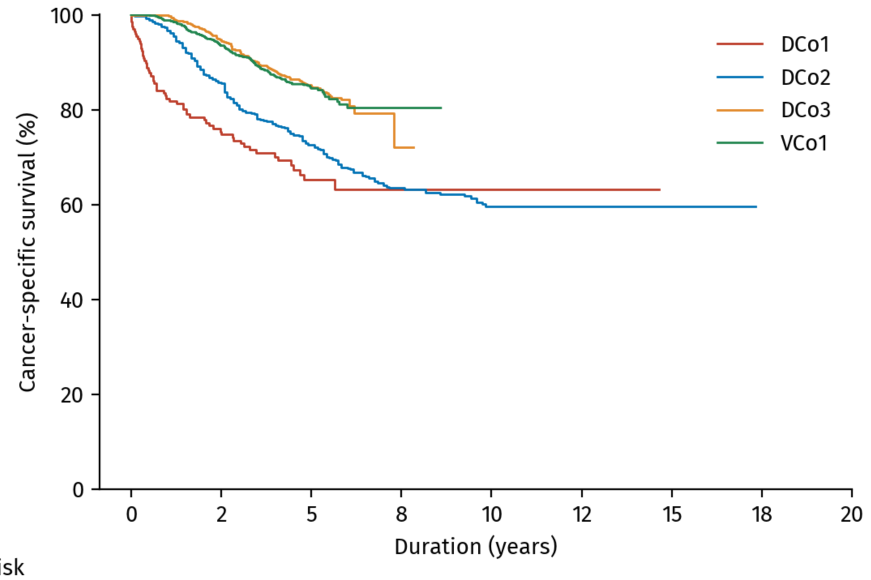

Number at risk

DCo1 (missing *n*=2)	204	122	50	10	5	5	0	0	0
DCo2	578	467	360	231	123	55	10	0	0
DCo3	765	689	424	4	0	0	0	0	0
VCo1 (missing *n*=14)	1118	989	425	8	0	0	0	0	0

Protocol Figure 16: Kaplan-Meier analysis of survival in colorectal carcinoma materials. Duration is years since randomisation for VCo1 and years since surgery for the other cohorts.

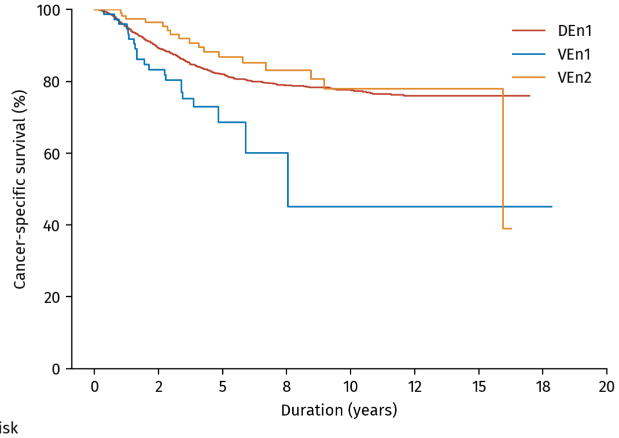

Number at risk

	0	2	5	8	10	12	15	18	20
DEn1 (missing *n*=4)	1237	1068	929	622	432	248	86	0	0
VEn1	77	57	13	4	3	2	1	1	0
VEn2	132	91	60	37	18	7	3	0	0

(a) Cancer-specific survival

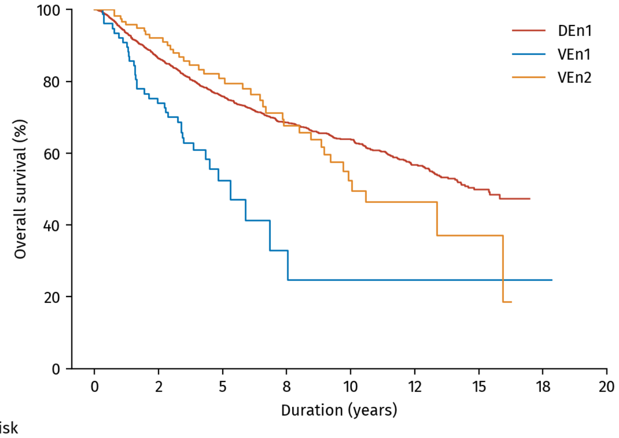

Number at risk

	0	2	5	8	10	12	15	18	20
DEn1 (missing *n*=4)	1237	1068	929	622	432	248	86	0	0
VEn1	77	57	13	4	3	2	1	1	0
VEn2	132	91	60	37	18	7	3	0	0

(b) Overall survival

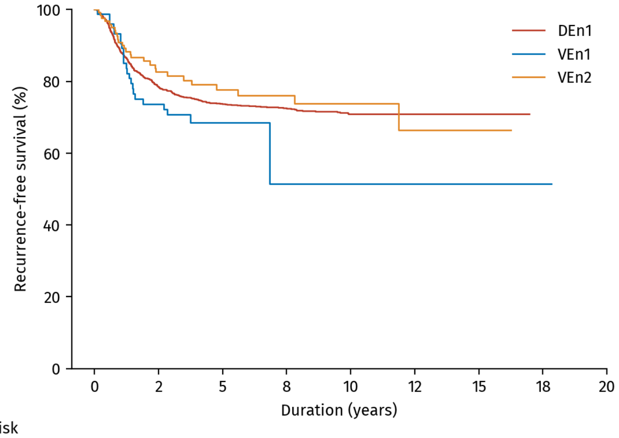

Number at risk

	0	2	5	8	10	12	15	18	20
DEn1 (missing *n*=4)	1237	938	831	564	390	227	77	0	0
VEn1	77	49	12	3	3	2	1	1	0
VEn2	132	78	53	34	17	6	2	0	0

(c) Recurrence-free survival

Protocol Figure 17: Kaplan-Meier analysis of survival in endometrial carcinoma materials. Duration is years since surgery.

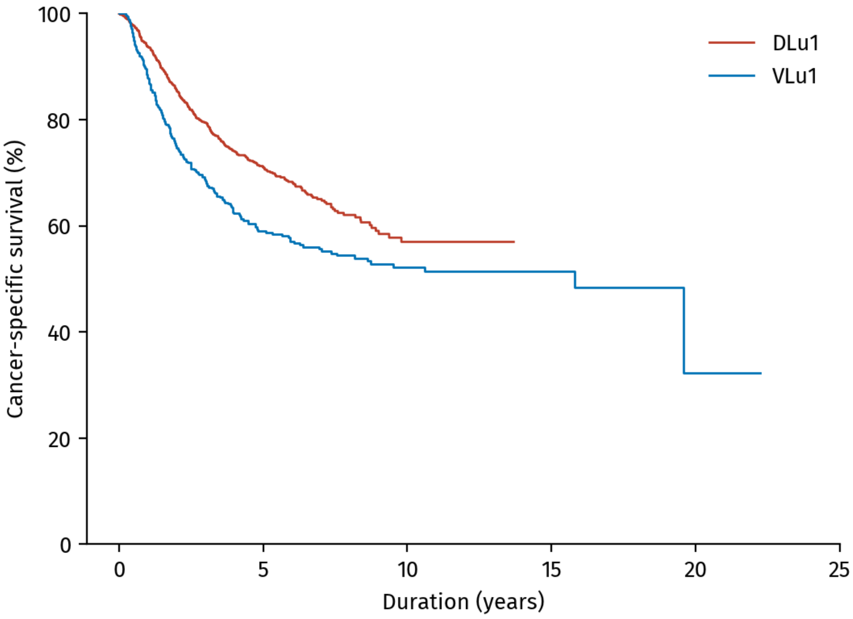

Number at risk

DLu1 (missing *n*=11)	922	424	60	0	0	0
VLu1	522	202	76	22	2	0

Protocol Figure 18: Kaplan-Meier analysis of survival in lung carcinoma materials. Duration is years since surgery for DLu1 and years since diagnosis for VLu1.

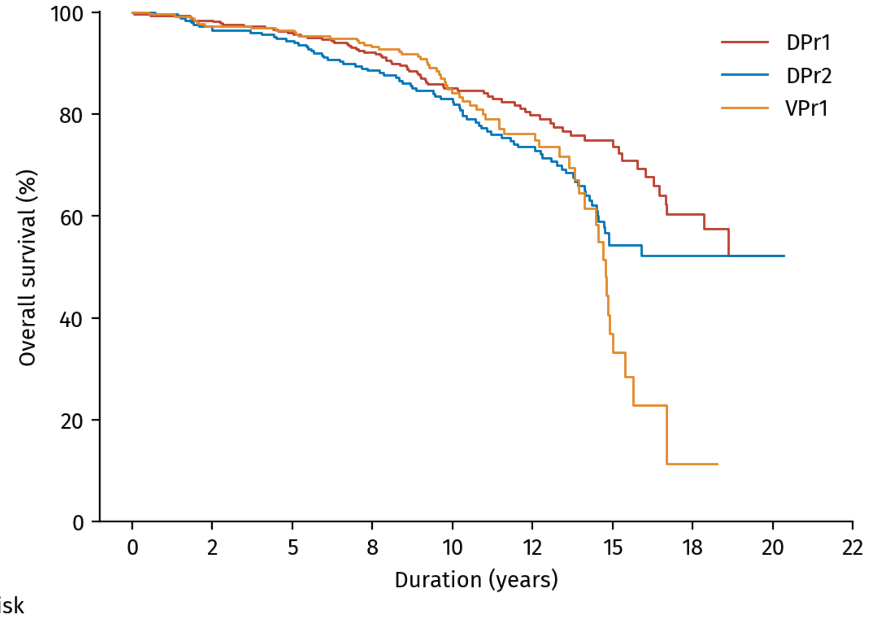

Number at risk

DPr1	328	319	307	286	195	115	61	26	2	0
DPr2 (missing *n*=1)	253	244	233	192	150	108	45	12	1	0
VPr1	259	249	242	224	113	64	10	1	0	0

(a) Overall survival

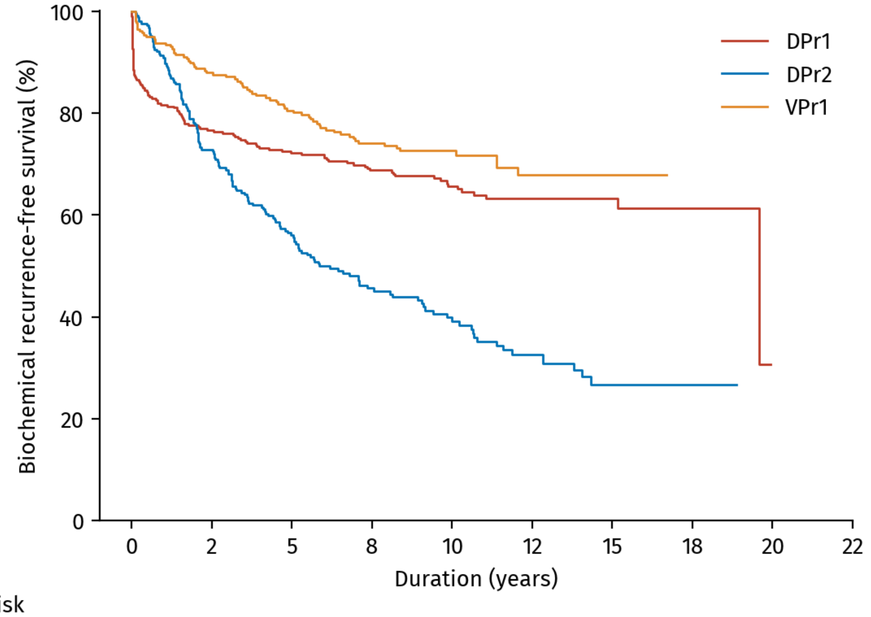

Number at risk

DPr1	328	245	220	195	119	69	37	14	0	0
DPr2 (missing *n*=1)	253	180	131	85	54	37	13	4	0	0
VPr1	259	218	193	165	80	40	4	0	0	0

(b) Biochemical recurrence-free survival

Protocol Figure 19: Kaplan-Meier analysis of survival in prostate carcinoma materials. Duration is years since surgery.

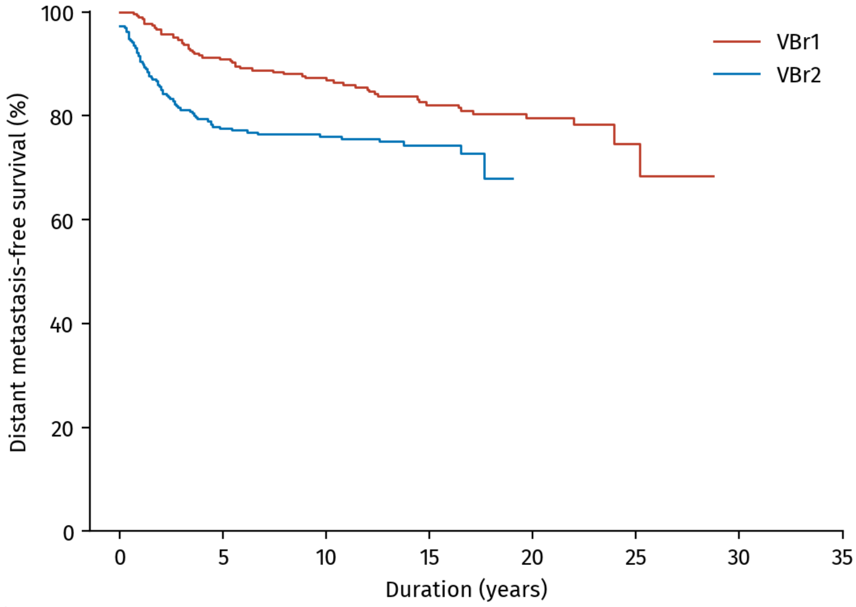

Number at risk

VBr1	310	261	206	154	94	14	0	0
VBr2 (missing n=10)	294	212	174	82	0	0	0	0

(a) Distant metastases-free survival

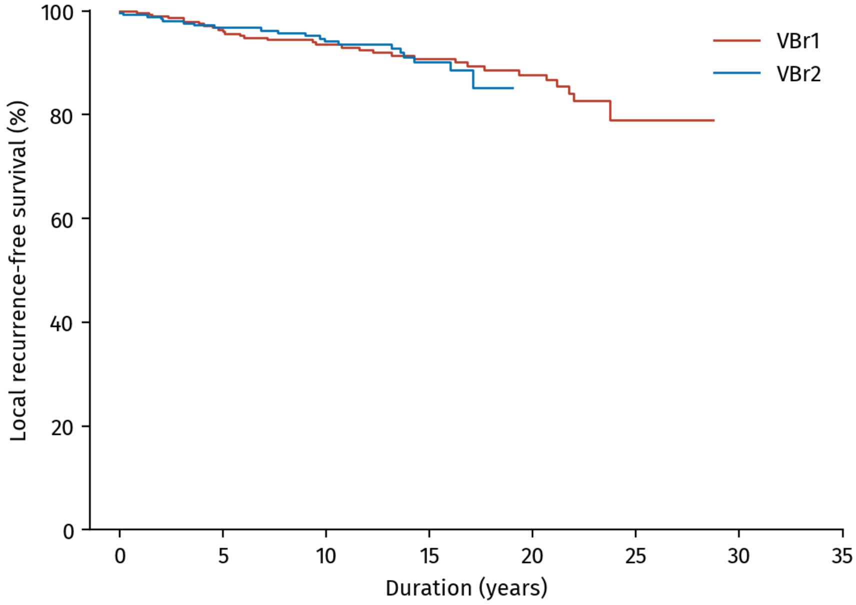

Number at risk

VBr1	310	252	195	144	89	13	0	0
VBr2 (missing n=11)	293	207	169	77	0	0	0	0

(b) Local recurrence-free survival

Protocol Figure 20: Kaplan-Meier analysis of survival in breast carcinoma materials. Duration is years since diagnosis. Note different follow-up times between the different event types.

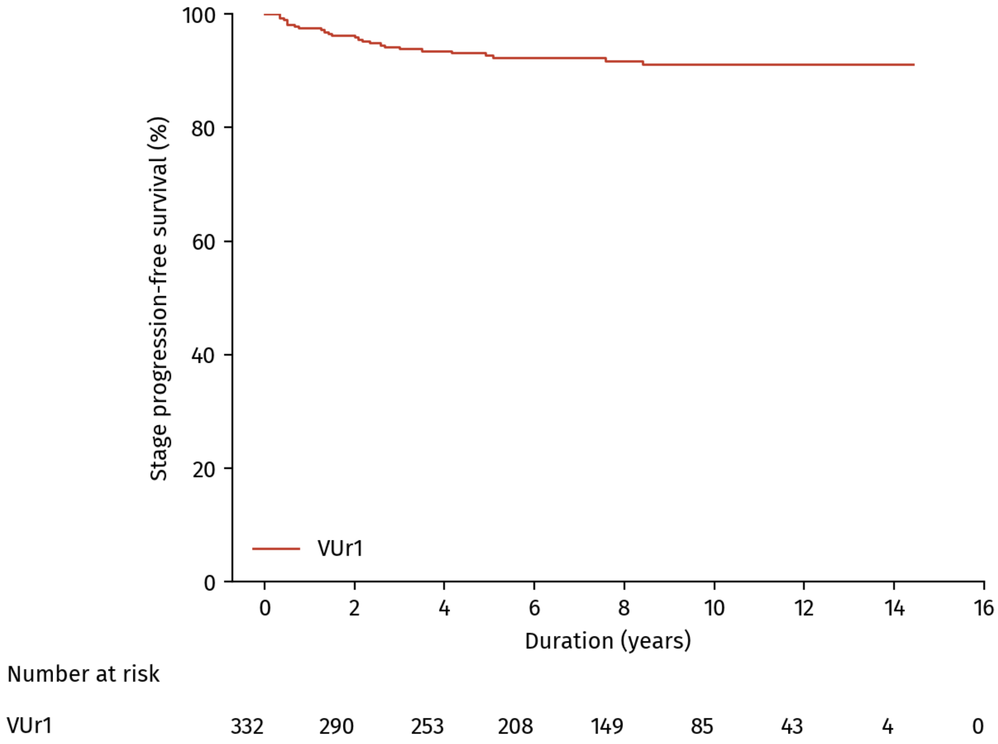

(a) Stage progression-free survival

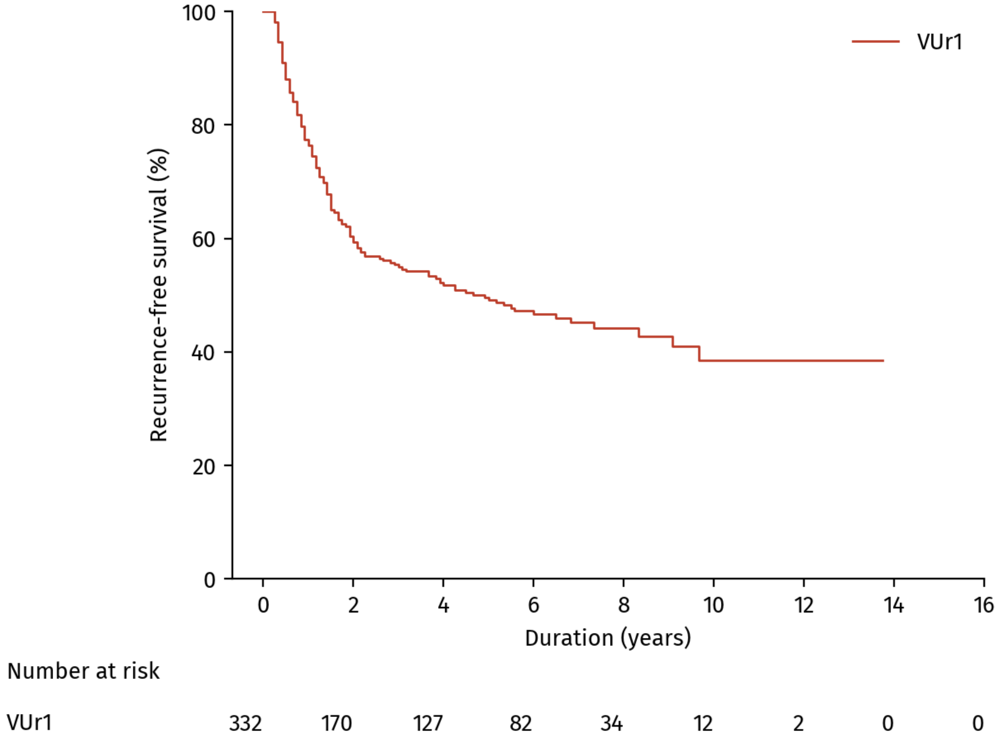

(b) Recurrence-free survival

Protocol Figure 21: Kaplan-Meier analysis of survival in urothelial carcinoma material. Duration is years since diagnosis. Note different follow-up times between the event types.

1.3.3 Colour statistics

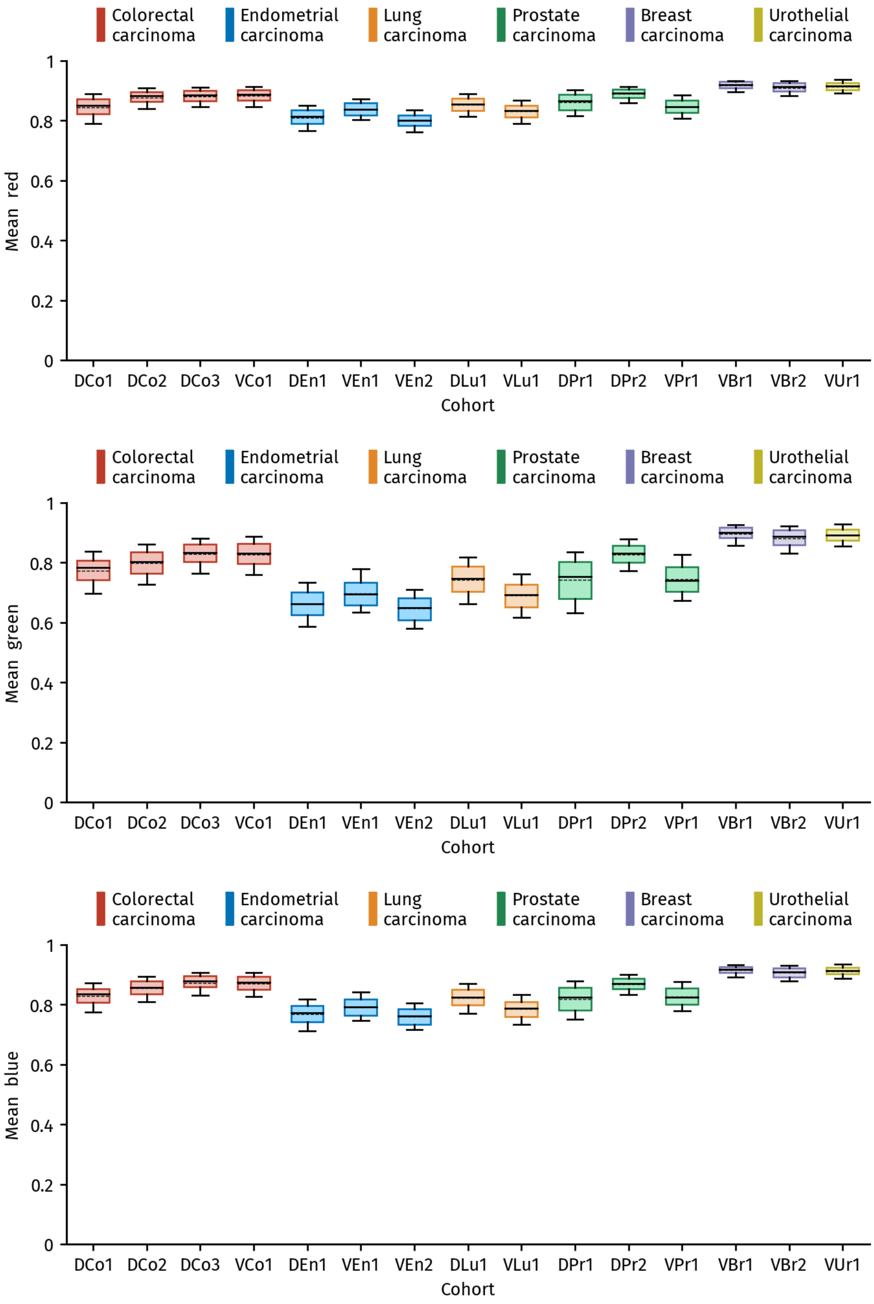

Protocol Figure 22: Colour channel mean value in full Aperio AT2 scans downscaled to 5 µm per pixel

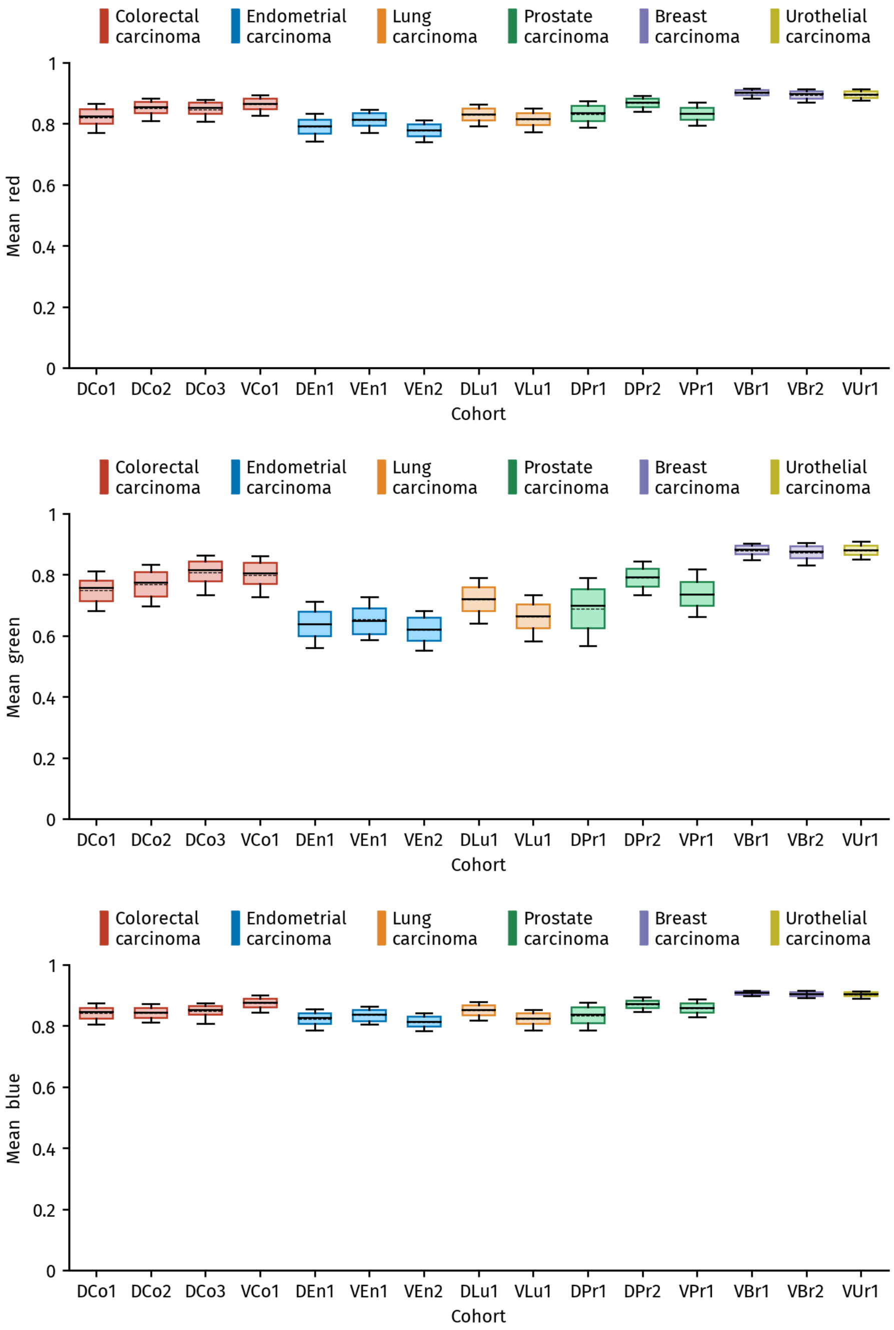

Protocol Figure 23: Colour channel mean value in full NanoZoomer XR scans downscaled to 5 µm per pixel

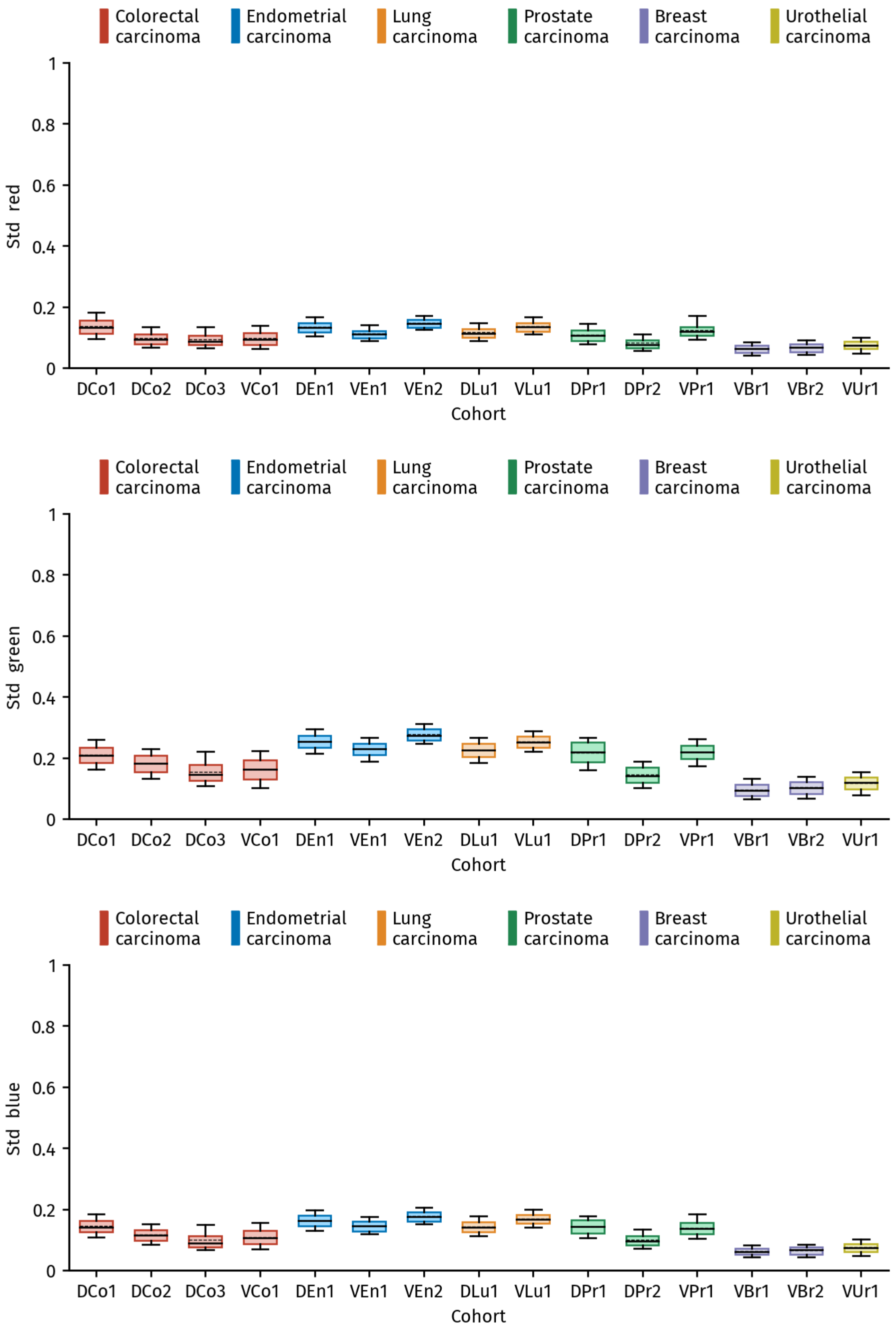

Protocol Figure 24: Colour channel standard deviation in full Aperio AT2 scans downscaled to 5 µm per pixel

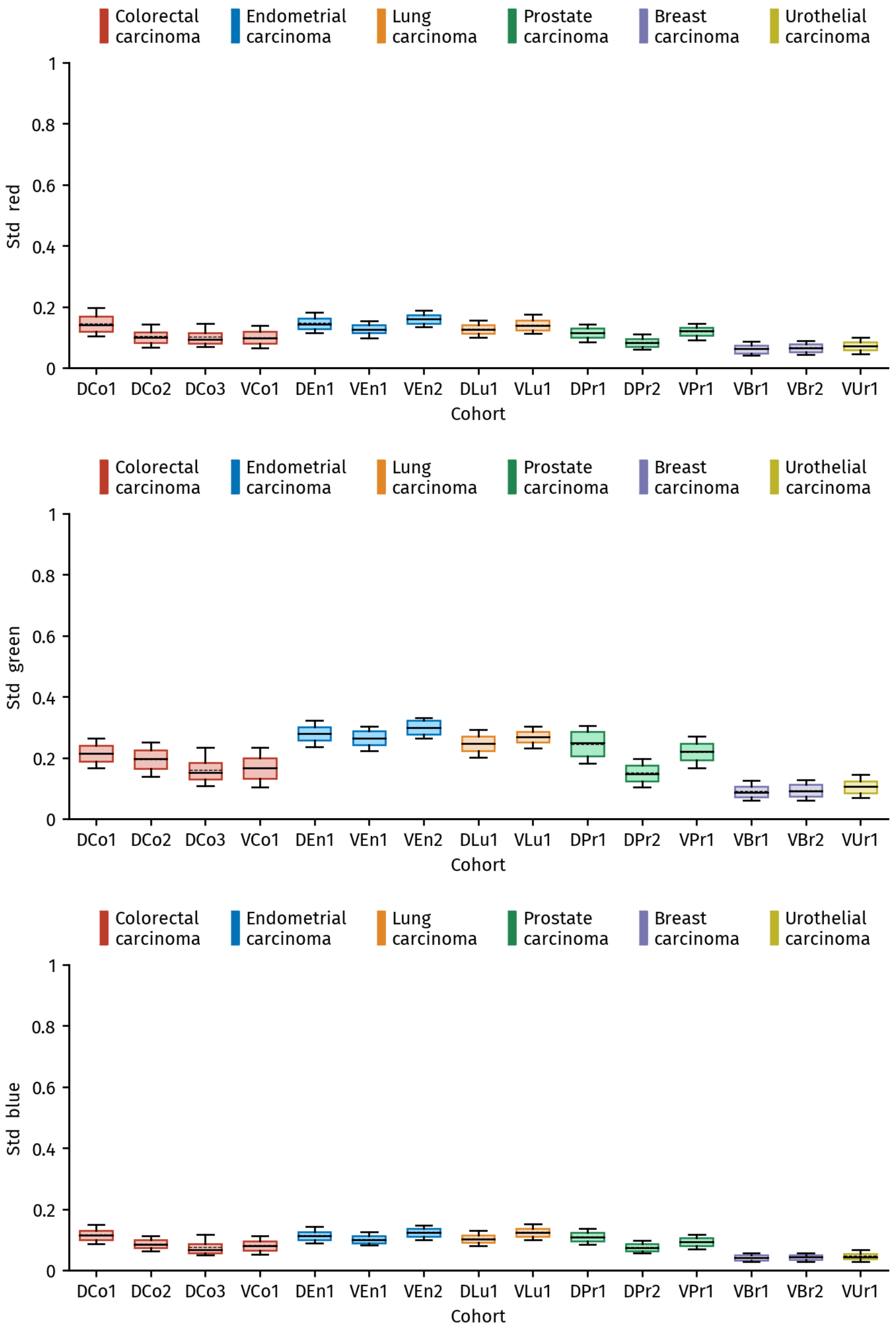

Protocol Figure 25: Colour channel standard deviation in full NanoZoomer XR scans downscaled to 5 µm per pixel

2 Methods

This section give a detailed explanation of how the segmentation method was developed and how it is applied. The software and hardware development environment is described in section 2.1. Steps necessary for preparing the method are described in section 2.2 while the segmentation method itself is described in section 2.3. How we evaluate the performance of the segmentation result is explained in section 2.4.

2.1 Programming environment

Most programs used in this project are implemented in the *Python* programming language. For method validation, programs were run in a *Docker container* based on the `pytorch/pytorch:1.11.0-cuda11.3-cudnn8-runtime` image. The network optimisation was run in a Docker container based on the image `nvcr.io/nvidia/pytorch:22.02-py3`. Additional python packages used are listed in protocol table 9.

Protocol Table 9: Python packages and their versions

Package	Version
`albumentations`	`1.1.0`
`matplotlib`	`3.5.1`
`opencv_python_headless`	`4.5.5.64`
`openslide_python`	`1.1.2`
`pandas`	`1.4.1`
`segmentation_models_pytorch`	`0.2.1`
`scikit-image`	`0.19.2`
`timm`	`0.4.12`
`toml`	`0.10.2`
`torchinfo`	`1.6.3`

Segmentation network processing was done on graphical processing units (GPUs). We used an Nvidia DGX machine with 8 A100 40 GB SXM GPUs, driver version 470.57.02 and CUDA version 11.4 for the network optimisation. For the validation, we used computers with Nvidia Titan RTX 24 GB GPU cards with driver version 465.19.01 and CUDA version 11.3.

2.2 Method development

The segmentation method use a convolutional neural network which needs to be optimised to this particular task of tumour segmentation. All necessary steps needed for preparation are described in this section and are summarised next:

1. Read input scans and downsample them to resolution 1 µm per pixel (section 2.2.1)
2. Partition each scan into tiles with 2 048 × 2 048 pixels (section 2.2.2)
3. Balance the development dataset (section 2.2.3)
4. Exclude background tiles (section 2.2.5)
5. Augment the development dataset (section 2.2.6)
6. Standardise input images (section 2.2.7)
7. Optimise the segmentation network (section 2.2.8)

2.2.1 Downsampling

Each scan used in this study is downsampled to a target resolution of 1 µm per pixel (MPP). For reference, the highest resolution of many scans is labelled 40× magnification which corresponds to about 0.25 MPP depending on the scanner vendor and model. As an example, we have scans from Aperio AT2 with a 40× magnification with resolution 0.2530 MPP and scans from NanoZoomer XR with a 40× magnification with resolution 0.2267 MPP (rounded to four decimal places).

The target downsampling factor is found by dividing the target MPP by the MPP at the highest resolution level of the scan (*level 0*). The level 0 MPP is accessed from the scan by `OpenSlide` using the `PROPERTY_NAME_MPP_X` and `PROPERTY_NAME_MPP_Y`. In case the directional level 0 MPP are different*, the target downsampling factor will also be different in the two directions. If these two properties are not available in the scan, it is not included in the study.

For neural network optimisation and application, we read tile regions from the scan file one by one rather than the entire scan. Each tile is read from the scan at the pyramid level with a corresponding downsampling factor smaller than or equal to the target downsampling factor (or the smallest of the two directional target downsampling factors if they are different). Unless the target downsampling factor is equal to the reading downsampling factor, the size of the read tile will be larger than the target size. The enlarged tile is therefore downsampled to the target size so that the resulting resolution is equal to the target resolution. Downsampling to a target size (instead of to a target factor) also ensures that the resulting tile has the exact height and width we desire (and not e.g. off-by-one due to rounding). This final resizing is performed using `OpenCV`s `resize` function with the `INTER_AREA` interpolation option. This ensures that no upsampling is performed, but may result in tiles being read from the scan at different resolutions depending on the scanner model and settings.

For background exclusion, performance evaluation and display purposes, we use the downsampled scan as a single image, and in these cases the scan is downsampled to a resolution of 5 MPP (about 2× magnification). Extracting the image from the scan file is done as for the tiles explained in the previous paragraph, with the exception that the target resolution is different and that the entire scan is read all at once in stead of in smaller regions.

2.2.2 Tiling

Since the downscaled scans are too large to process at a resolution of 1 MPP, they are partitioned into a set of tiles. The horizontal and vertical spatial dimensions are split in the same way, and the procedure for computing tile start and end coordinates is listed as python code in protocol listing 1.

The scan is partitioned into overlapping tiles if the scan dimension is not an integer multiple of the tile dimension and the minimum overlap is not specified to be 0. The amount of overlap is equal between all tile columns in the horizontal direction, except for between the rightmost tile columns which may overlap more, so that the rightmost tile column aligns with the right scan boundary. The same is true in the vertical direction where tile rows overlap with the same amount except perhaps for between the bottommost tile rows. With the procedure shown in protocol listing 1, we can also specify the minimum number of overlapping pixels along a dimension.

*In this study, none of the included scans had different directional level 0 MPP

```python
from typing import List, Tuple
import numpy as np


def find_overlap(full_size: int, part_size: int, min_overlap: int) -> Tuple[float, int]:
    assert full_size > part_size, "The part is larger than the whole"
    num_parts = int(np.ceil(full_size / part_size))
    overlap = (part_size * num_parts - full_size) / (num_parts - 1)
    if overlap < min_overlap:
        assert part_size > min_overlap, "Part size must be greater than minimal overlap"
        num_parts = int(np.ceil((full_size - min_overlap) / (part_size - min_overlap)))
        overlap = (part_size * num_parts - full_size) / (num_parts - 1)
    return overlap, num_parts


def partition(full_size: int, part_size: int, min_overlap: int) -> List[range]:
    """
    Divide a full line into parts where the line have size full_size and the parts have
    size part_size (except when full_size < part_size).

    Return a list of part start (inclusive) and stop (exclusive) points on the full line
    """
    ranges: List[range] = []
    if full_size > part_size:
        overlap, num_parts = find_overlap(full_size, part_size, min_overlap)
        fractional_part = overlap - np.floor(overlap)
        num_ceils = int(np.floor(num_parts * fractional_part))
        for k in range(num_parts):
            if k <= num_ceils:
                int_overlap = int(np.ceil(overlap))
            else:
                int_overlap = int(np.floor(overlap))
            if k == 0:
                start = 0
            else:
                start = ranges[k - 1].stop - int_overlap
            ranges.append(range(start, start + part_size))
    else:
        ranges.append(range(0, full_size))
    return ranges
```

Protocol Listing 1: Divide with overlap

Tiles used for network optimisation have a target spatial dimension of $2\,048 \times 2\,048$ pixels and are sampled from the scan with a minimum overlap of 0 pixels. Tiles used for network inference have a target spatial dimension of $7\,680 \times 7\,680$ pixels with a minimum overlap of $1\,024$ pixels.

Scan tiles are written as `jpg` files with 95% quality while annotation mask tiles are written as `png` files. Full scans at 5 MPP are written as `png` files.

2.2.3 Dataset balancing

The development set was balanced w.r.t. cancer type by oversampling the minority groups on a tissue slide level. Tissue slides were selected multiple times at random without replacement so that no slides were selected $n+1$ times before all slides had been selected n times. This resulted in 3 519 sections sampled from each cancer type (the same number of sections included in lung carcinoma, which was the majority group). Counting scans from both scanners, the result was 7 030 scans from colorectal carcinoma and 7 038 scans from each of the other cancer types. See protocol table 10 for an overview of the number of scans for each cohort. Note that since the selection was done on a slide level, and cohorts DCo2 and DCo3 had fewer NanoZoomer XR scans than Aperio AT2, there are slightly fewer scans from NanoZoomer XR than from Aperio AT2 in protocol table 10 for these two cohorts.

Protocol Table 10: Number of annotated scans in the training cohorts after balancing with oversampling

Cancer type	Cohort	Scans	
		Aperio AT2	NanoZoomer XR
Colorectal carcinoma	DCo1	471	471
	DCo2	1 309	1 303
	DCo3	1 739	1 737
	Sum	3 519	3 511
Endometrial carcinoma	DEn1	3 519	3 519
Lung carcinoma	DLu1	3 519	3 519
Prostate carcinoma	DPr1	1 981	1 981
	DPr2	1 538	1 538
	Sum	3 519	3 519
Sum		14 076	14 068

2.2.4 Background segmentation

A simple method is employed to segment the white background in an image from the rest. This background mask is used to alter both predicted and reference segmentation masks. This is useful when large background regions are inside the annotated region (one example being holes from *tissue microarray* acquisition) without being manually annotated as background. These regions are clearly not cancerous tissue, and should not be annotated as such neither by the reference nor by the prediction.

Note that this segmentation is quite sensitive in that it will mark most tissue as foreground, also adipose tissue that is often left out when applying threshold methods based on image brightness or saturation or similar. But it may also include artefacts such as pen markings, air bubbles, dust, glass cracks, etc. But since the mask is used to exclude white background tiles used in training, it can be an advantage that foreground elements other than tissue is included. The method with the stated parameter values assumes images of H&E-stained tissue with 5 MPP resolution.

Canny edge detection is performed on the input colour image, using the `OpenCV Canny` implementation.[26] We use a 3×3 *Sobel* filter for the gradient computation, and thresholds of 10 and 50 for the lower and upper thresholds in the hysteresis. This produce a mask with lots of foreground pixels in regions with structure and lots of background pixels in homogeneous regions.

This foreground mask is refined by first removing small background regions. The mask first

undergoes morphological closing (`openCV morphologyEx`) with a square 9×9 structure element before background regions with an area smaller than 10 000 pixels are filled in with the function `remove_small_holes` from the `scikit-image` python library. An area of 10 000 pixels at 5 MPP resolution corresponds to a square region of $0.5\,\text{mm} \times 0.5\,\text{mm}$.

Finally, small foreground regions are removed from the mask. Morphological opening is applied on the mask using the `openCV` function `morphologyEx` with the same 9×9 structure element before foreground regions with an area smaller than 1 600 pixels are erased using the function `remove_small_objects` from the `scikit-image` python library. An area of 1 600 pixels corresponds to a square region of $0.2\,\text{mm} \times 0.2\,\text{mm}$ at 5 MPP resolution.

This method is simple to implement, very robust, and quite fast, spending around one second per image on a single CPU core on consumer-grade hardware. An example of a downscaled scan from colorectal carcinoma scanned with Aperio AT2 and manually annotated is shown in protocol figure 26.

With this we can classify every pixel as either white background, foreground without annotation and foreground with annotation. This content classification is summarised for all scans in all cohorts used in this study in protocol figures 27 to 30.

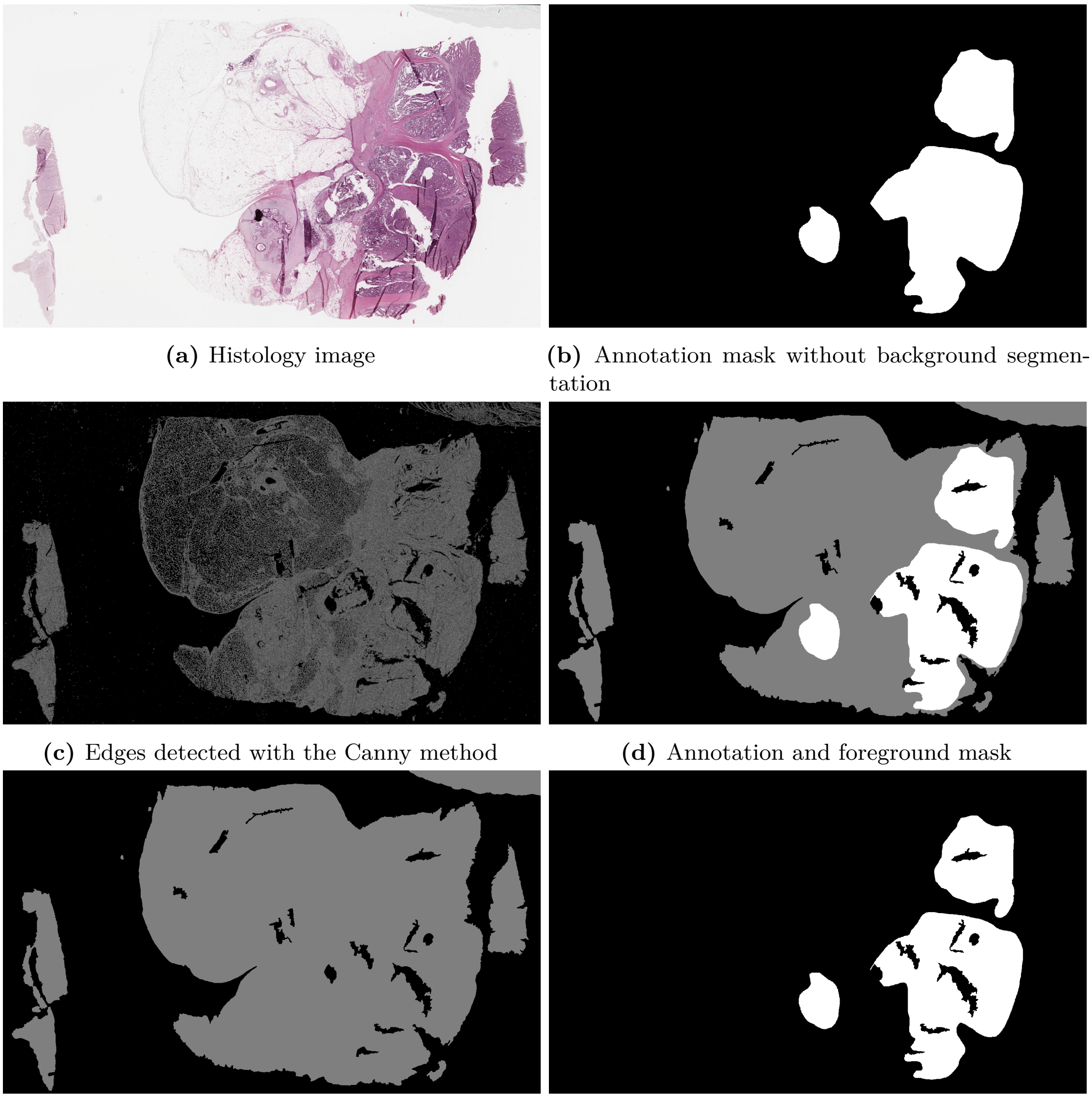

(a) Histology image
(b) Annotation mask without background segmentation
(c) Edges detected with the Canny method
(d) Annotation and foreground mask
(e) Foreground mask
(f) Annotation mask after removing background

Protocol Figure 26: Scan and segmentation mask with background (black), foreground (gray) and tumour annotation (white)

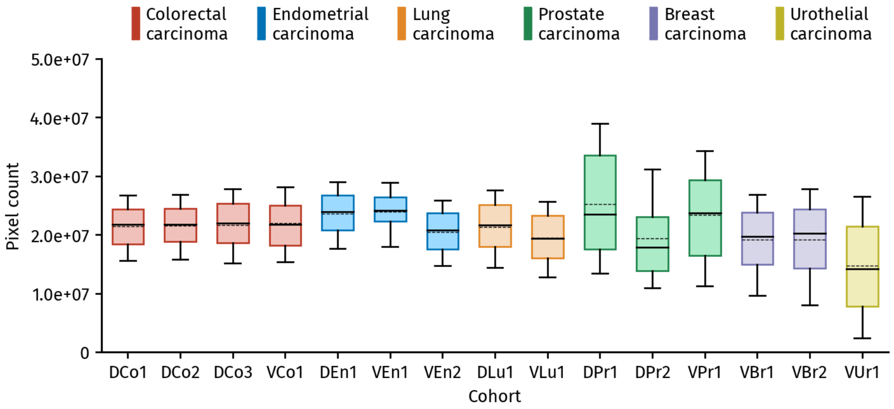

(a) Image area

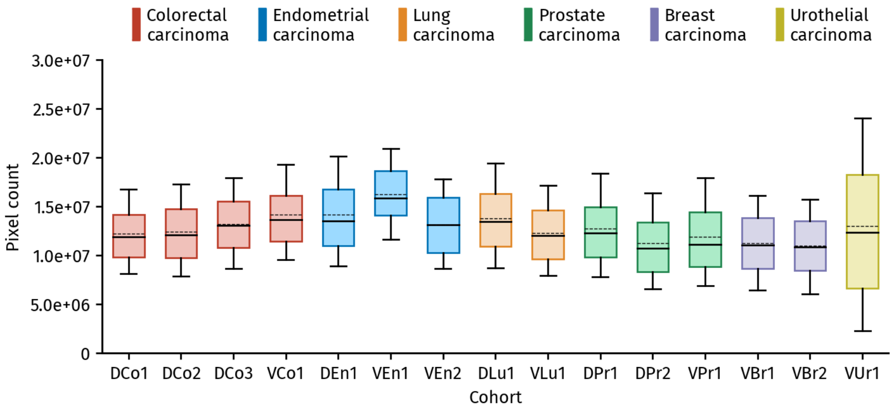

(b) Foreground area

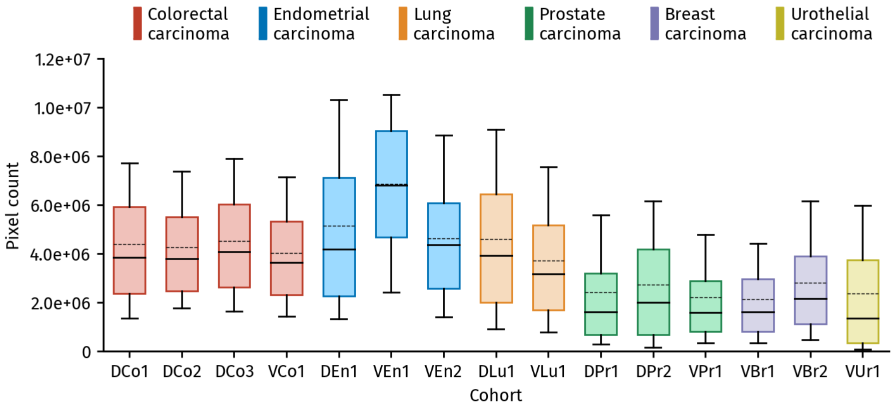

(c) Annotation area

Protocol Figure 27: Area in number of pixels at resolution 5 µm per pixel in Aperio AP2 scans. "Foreground" is foreground without annotation and "Annotation" is foreground with annotation. Background exclusion masks are applied on all images. Note the difference in vertical axis range between subplots.

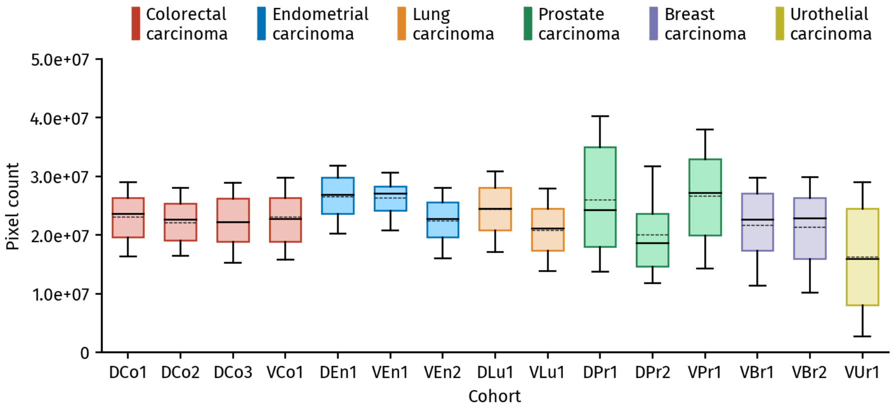

(a) Image area

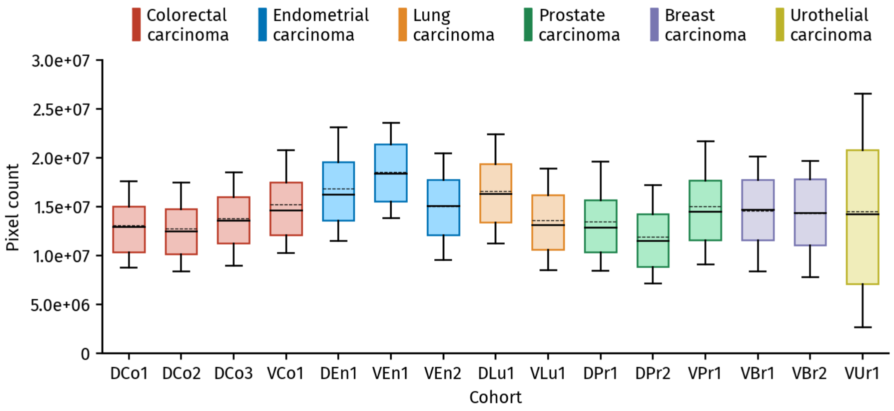

(b) Foreground area

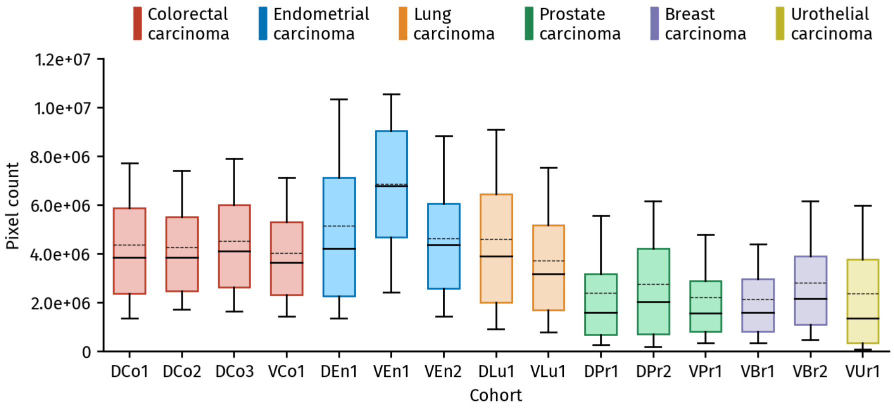

(c) Annotation area

Protocol Figure 28: Area in number of pixels at resolution 5 µm per pixel in NanoZoomer XR scans. "Foreground" is foreground without annotation and "Annotation" is foreground with annotation. Background exclusion masks are applied on all images. Note the difference in vertical axis range between subplots.

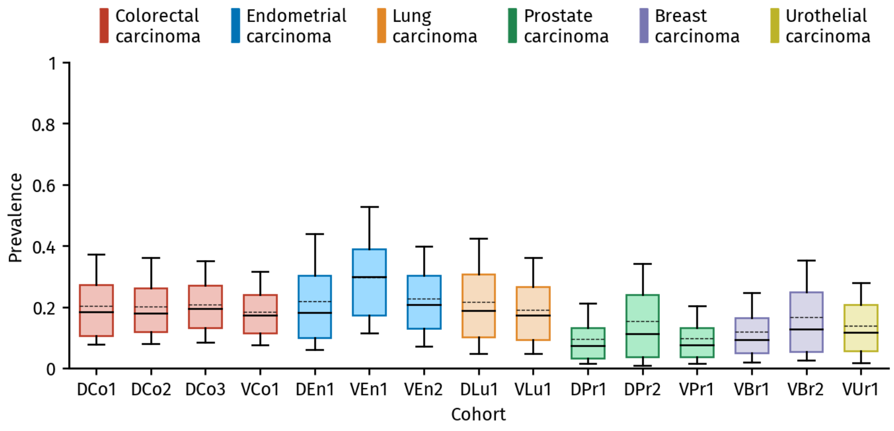

(a) Annotation prevalence in image

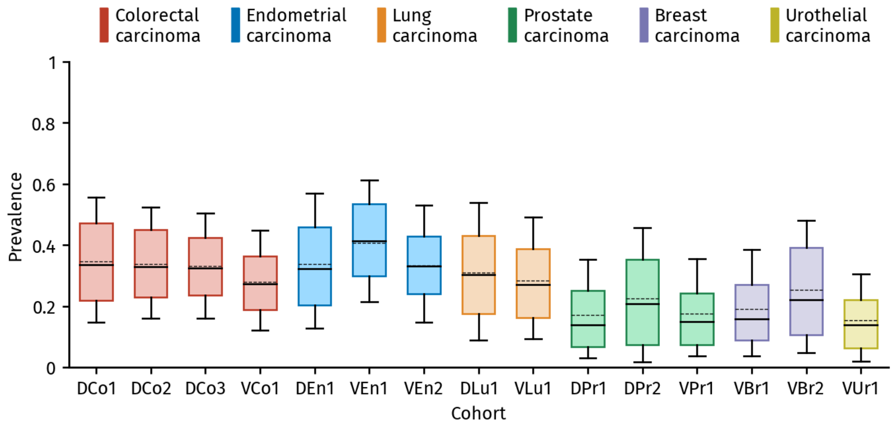

(b) Annotation prevalence in foreground

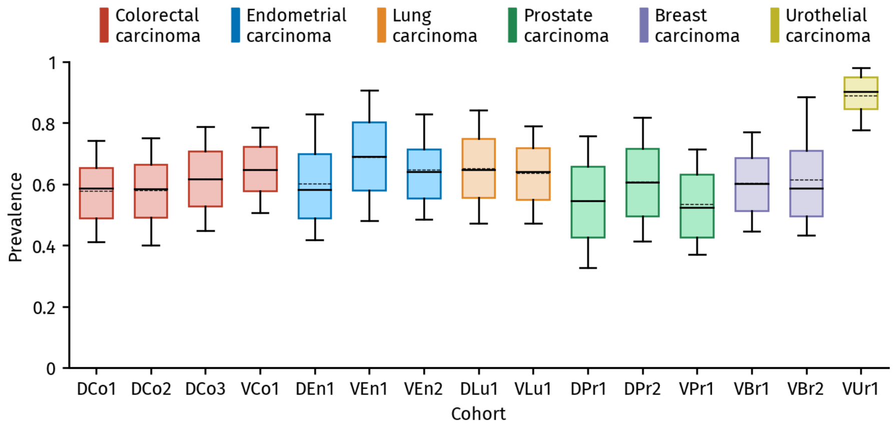

(c) Foreground prevalence in image

Protocol Figure 29: Prevalence at resolution 5 µm per pixel in Aperio AT2 scans. "Foreground" is foreground without annotation and "Annotation" is foreground with annotation. Background exclusion masks are applied on all images.

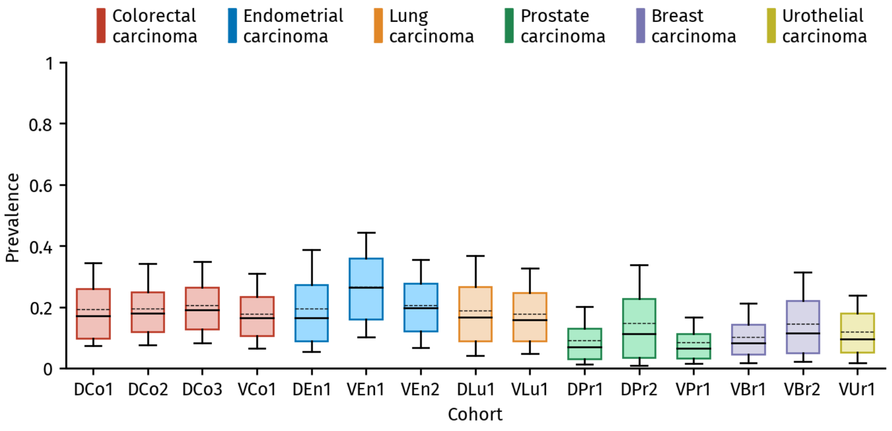

(a) Annotation prevalence in image

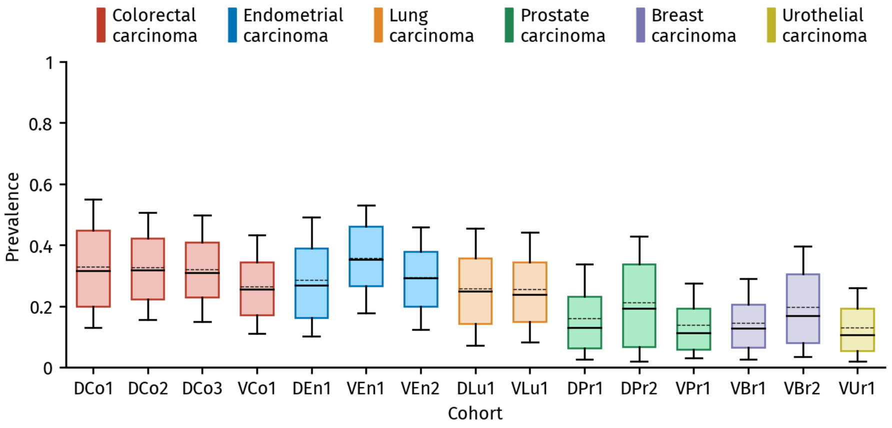

(b) Annotation prevalence in foreground

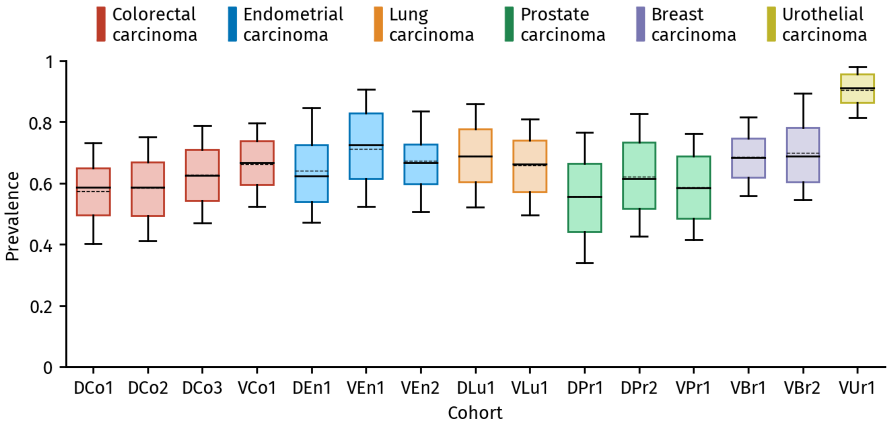

(c) Foreground prevalence in image

Protocol Figure 30: Prevalence at resolution 5 µm per pixel in NanoZoomer XR scans. "Foreground" is foreground without annotation and "Annotation" is foreground with annotation. Background exclusion masks are applied on all images.

2.2.5 Background tile exclusion

Tiles containing too much white background are removed from the development set. The background segmentation is performed on 5 MPP full images as described in section 2.2.4 and transferred to the 1 MPP tiles. Specifically, we include all tiles that contain some tumour annotated regions, and for those that don't, we keep those with a background fraction smaller than 50%.

In total this reduces the number of unique tiles from 3 080 330 to 2 144 651 or from 4 233 081 to 2 902 032 non-unique tiles in the balanced dataset (see protocol table 11 and protocol figure 31).

Protocol Table 11: Number of unique tiles in development cohorts before and after background exclusion

Cohort	Aperio AT2			NanoZoomer XR			Both scanners		
	Tiles before	Tiles after	%	Tiles before	Tiles after	%	Tiles before	Tiles after	%
DCo1	28 911	19 429	67.20	30 552	20 245	66.26	59 463	39 674	66.72
DCo2	81 651	54 356	66.57	82 294	55 104	66.96	163 945	109 460	66.77
DCo3	108 429	76 511	70.56	109 720	78 609	71.65	218 149	155 120	71.10
DEn1	514 341	340 922	66.28	566 105	394 211	69.64	1 080 446	735 133	68.04
DLu1	492 150	364 029	73.97	551 767	422 192	76.52	1 043 917	786 221	75.31
DPr1	158 001	91 707	58.04	163 527	96 500	59.01	321 528	188 207	58.54
DPr2	95 015	63 855	67.21	97 867	66 981	68.44	192 882	130 836	67.83
Sum	1 478 498	1 010 809	68.37	1 601 832	1 133 842	70.78	3 080 330	2 144 651	69.62

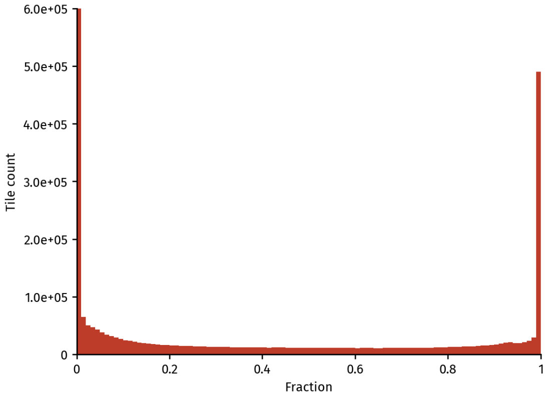

(a) Background before

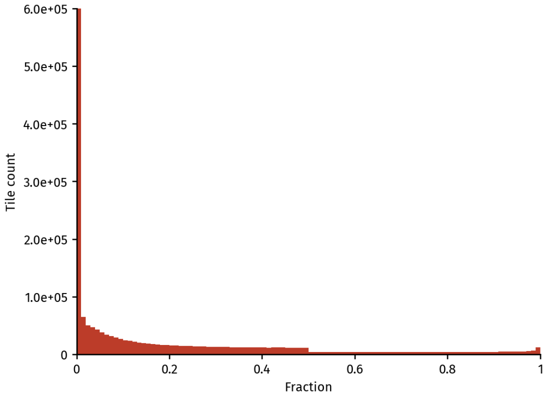

(b) Background after

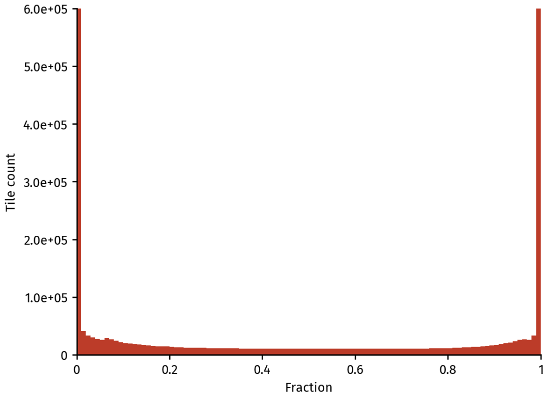

(c) Non-annotated foreground before

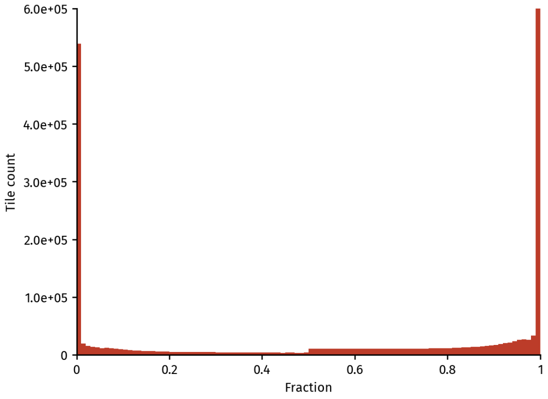

(d) Non-annotated foreground after

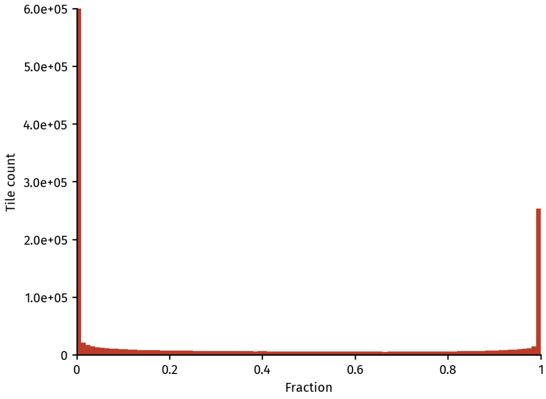

(e) Annotated foreground before

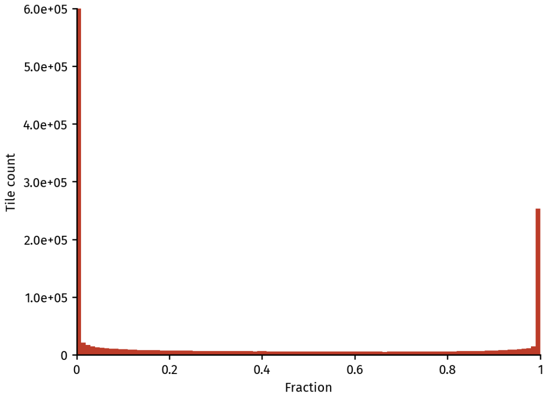

(f) Annotated foreground after

Protocol Figure 31: Distribution of number of tiles with a certain fraction of either background (top row) non-annotated foreground (middle row) and annotated foreground (bottom row) before (left column) and after (right column) background exclusion.

2.2.6 Dataset augmentations

Image tiles are read as *RGB* with 8 bits values per channel, cast to 32 bits floating point values, and then preprocessed before they enter the segmentation network. We artificially augment the training dataset by distorting images using the `albumentations` library.[27] The operations are listed in protocol listing 2 in the order they are applied. Note that while the tiles are sampled at a size of 2 048 × 2 048 pixels, they are cropped to a size of 1 536 × 1 536 pixels before they enter the network.

Image distortions are only applied during network optimisation, and not when the fixed network is applied.

```
HorizontalFlip(p=0.5)
RandomRotate90(p=0.5)
PadIfNeeded(min_height=1536, min_width=1536, border_mode=cv2.BORDER_CONSTANT, value=0)
RandomCrop(height=1536, width=1536)
RandomBrightnessContrast(
  brightness_limit=[-0.2, 0.2], contrast_limit=[-0.2, 0.2], brightness_by_max=True, p=1
)
HueSaturationValue(
  hue_shift_limit=[-26, 26], sat_shift_limit=[-26, 26], val_shift_limit=[-26, 26], p=1
)
```

Protocol Listing 2: Image distortions

2.2.7 Image value standardisation

Before the image enters the network, the image values are divided by 255 before the image is centred around the development dataset mean value and scaled with the development dataset standard deviation. This standardisation is applied both during network optimisation and inference.

The dataset mean value for an image channel is computed as

$$\begin{aligned}\mu &= \frac{1}{m}\sum_{i=1}^{m}\mu_i \\ &= \frac{1}{m}\sum_{i=1}^{m}\frac{1}{n_i}\sum_{j=1}^{n_i}x_{ij}\end{aligned}$$

where x_{ij} is the value at pixel j in image i for the image channel and μ_i is the mean value in image i. n_i is the number of pixels in image i, and m is the number of images in the dataset. Similarly, the dataset variance for a single channel is estimated as

$$\begin{aligned}\sigma^2 &= \frac{1}{m}\sum_{i=1}^{m}\sigma_i^2 \\ &= \frac{1}{m}\sum_{i=1}^{m}\frac{1}{n_i-1}\sum_{j=1}^{n_i}(x_{ij}-\mu_i)^2.\end{aligned}$$

We use $\sigma = \sqrt{\sigma^2}$ as the estimate for the dataset standard deviation. For both estimates μ and σ, the final result is divided by 255 before it is applied.

When applied on all unique 2 048 × 2 048-sized tiles in the development dataset at resolution 1 MPP without distortions, we get the result shown in protocol table 12 and protocol figures 32

and 33. Colour mean and standard deviation distributions for the all scans at resolution 5 MPP are shown in protocol figures 22 to 25.

Protocol Table 12: Colour statistics for all unique tiles in the development set. Here, all 8-bit integer colour channel values are cast to 32 bit floating point values before the per-image statistics are computed. These values are then averaged over all tiles and the result is divided by 255.

Colour channel	Mean value	Standard deviation
Red	0.8297992	0.1051075
Green	0.7106879	0.1543867
Blue	0.8241846	0.0991757

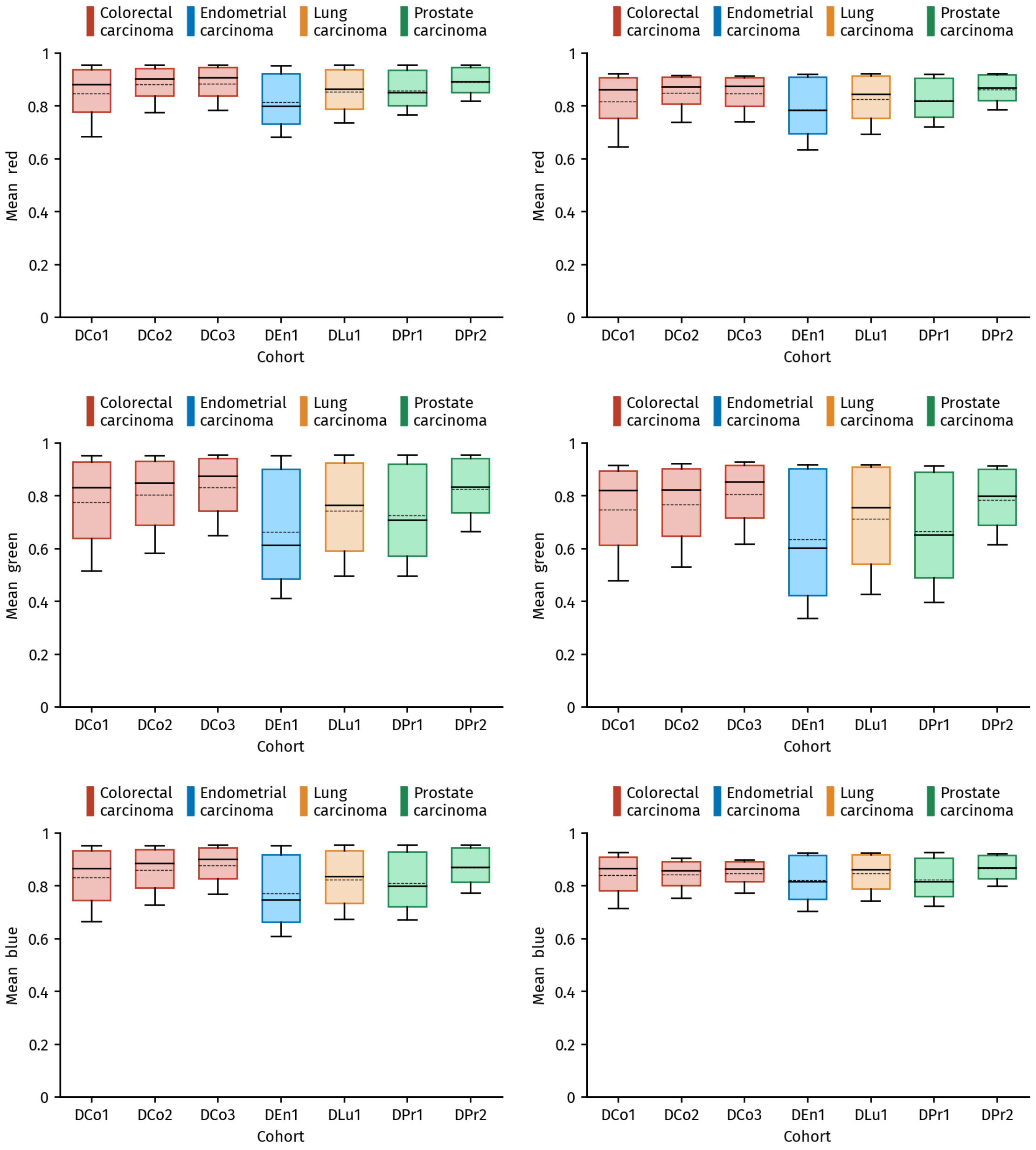

Protocol Figure 32: Colour channel mean value for all unique tiles in the development set. Tiles have resolution 1 MPP and a size of 2048 × 2048 pixels. Aperio AT2 in the left column and NanoZoomer XR in the right column.

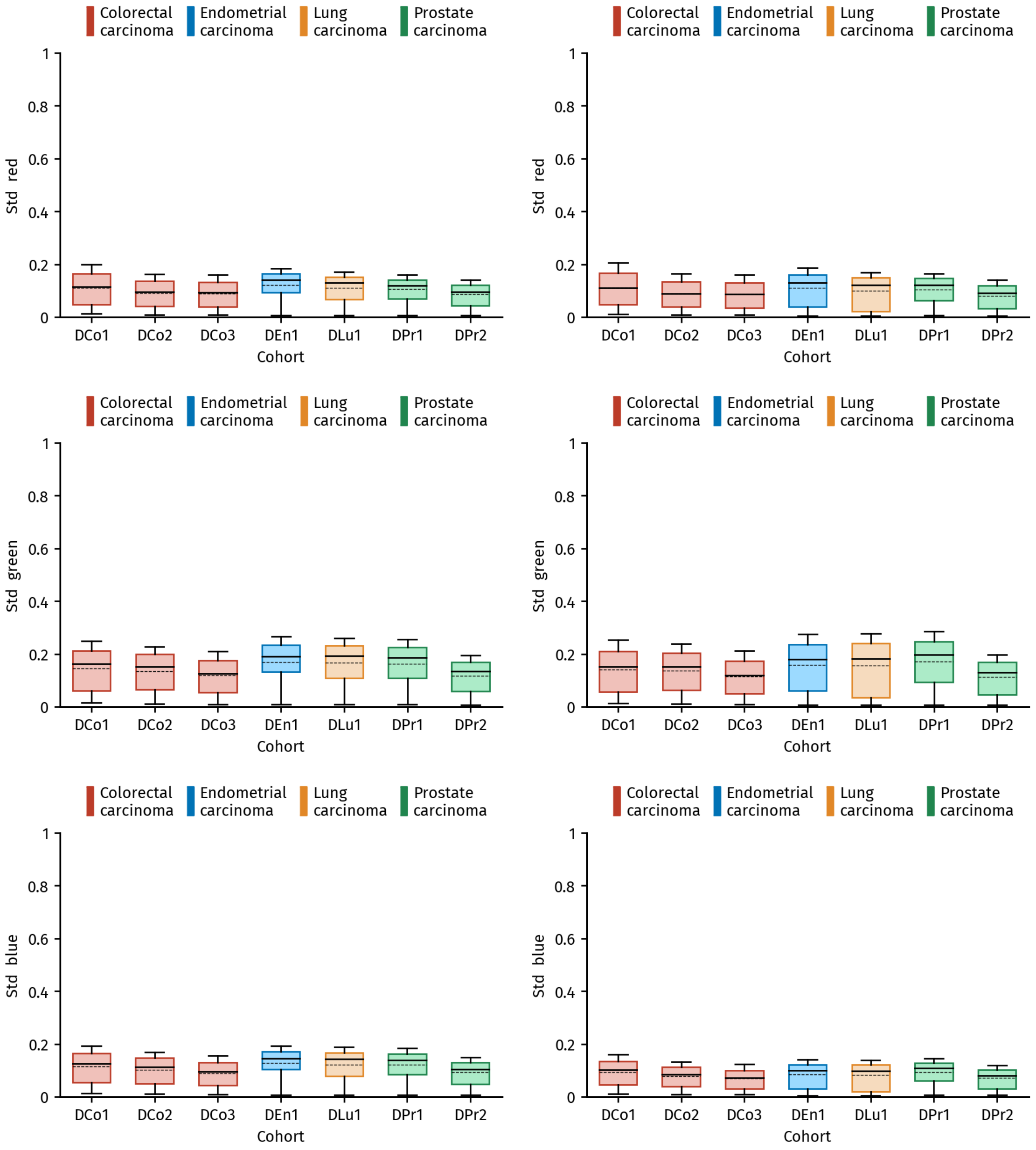

Protocol Figure 33: Colour channel standard deviation for all unique tiles in the development set. Tiles have resolution 1 MPP and a size of 2048 × 2048 pixels. Aperio AT2 in the left column and NanoZoomer XR in the right column.

2.2.8 Segmentation network

The segmentation network is an encoder-decoder network developed using the `PyTorch v1.11` machine learning framework.[28]

For the encoder we used a *Normalising-free Network* (NFNet), a modern classification network designed to achieve state-of-the-art performance without using batch normalisation.[29, 30] More specifically, we use the `eca_nfnet_l3` implementation provided by the `timm version 0.4.12` package.[31]

This implementation differ from the one described by Brock and colleagues in that it has 4, 8, 24, and 12 blocks for the four stages, respectively. The *Squeeze and Excitation* module is replaced by the *Efficient Channel Attention* module.[32, 33]. It also use *SiLu* activation functions instead of *GeLu*.[34].

The decoder is the decoder from the *DeepLabV3+* segmentation network, and the implementation is from the `segmentation_models_pytorch` python package.[35, 36] We modified the DeeplLabV3+ decoder to be free of batch normalization, following the NFNet encoder. To achieve this, we simply replaced every batch norm layer with a group norm layer with groups of size 8.[37]

The network consist in total of 73 472 403 adjustable parameters to be optimised (computed by `torchinfo`). An overview of the architecture can be seen in protocol figure 34.

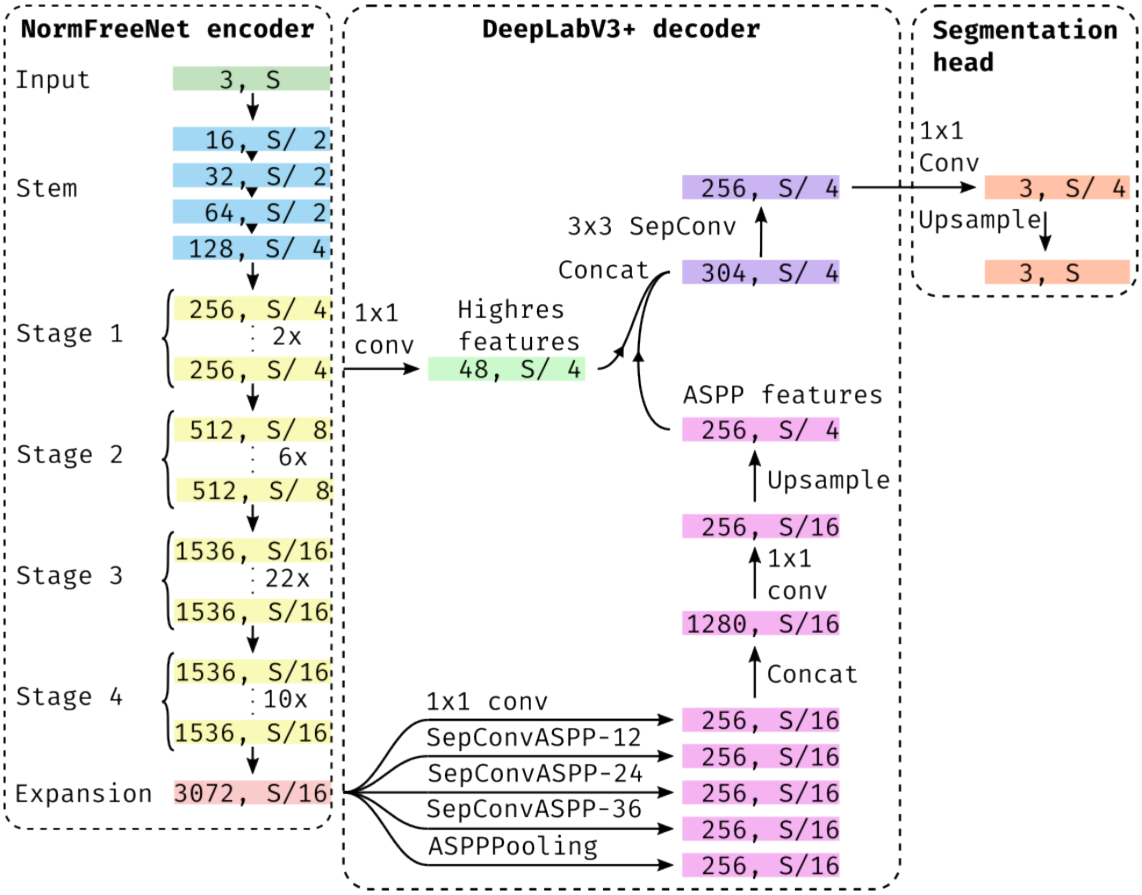

Protocol Figure 34: Segmentation network architecture. Each coloured block represent an element of the network and is described with two symbols separate by a comma. The first is the number of output channels and the second is the spatial size relative to the input size S. The first block has three channels since we use RGB-images, while the last block has three channels since we predict three classes.

2.2.9 Network optimisation

Below we describe how the network was optimised, but in general we follow the procedure described in by Brock and colleagues in their NFNet paper, with some exceptions.[29] We do *not* use *Adaptive Gradient Clipping* as we did not see any benefit for it in our case, perhaps because of our small batch size (24 images). We also do not use moving averages of the model parameters.

The objective is to minimise the difference between the output of the segmentation network and the reference segmentation by iteratively modifying the adjustable parameters of the segmentation network. The difference to be minimised is captured by the loss function $l = l_1 + l_2$, where l_1 is the so-called *Dice-loss* (`DiceLoss` from `segmentation_models_pytorch` with `mode="multiclass"`), and l_2 is a so-called *top-90 Cross Entropy* loss function. The top-90 Cross Entropy at a particular step is computed by first computing the per-pixel cross entropy for all pixels in the mini batch of this step and then averaging the cross entropy value over pixels in the top 90 percentile. That is, when computing the mean cross entropy, we are ignoring 10% of pixels with the lowest cross entropy value.

We predict three classes, and the reference is segmented into background, non-annotated foreground, and tumour-annotated foreground. We also experimented with using just two classes, tumour-annotated foreground and everything else, but we did not notice any important difference in performance.

The convolution weights in the encoder are initialised with *normal* initialisation while the biases are initialised to zero.

$$X \sim \mathcal{N}(0, \sigma^2), \text{ where } \sigma = \sqrt{\frac{1}{c_i hw}}.$$

The convolution weights and biases used in the decoder and segmentation head are initialised with *uniform* initialisation

$$X \sim \mathcal{U}(-a, a), \text{ where } a = \sqrt{\frac{1}{c_i hw}}.$$

In the above equations, $c_i hw$ is the volume of the input feature maps in the convolutional layer (number of input channels times the height times the width), often called *fan in*.[38]

At each iteration (or step), the adjustable network parameters are updated according to the *Stochastic Gradient Descent* optimisation method with *Nesterov momentum* 0.9.[39] The optimisation is regularised with a *weight decay* value of 2×10^{-5} with the exceptions described by Brock and colleagues.[29]

A batch of 24 images is randomly selected without replacement from the development dataset and processed at each step. When the dataset is exhausted we say that an *epoch* is complete, and the selection is reset. The whole batch is processed by the segmentation network before the output is compared with the corresponding reference segmentation batch with the objective function. The batch of 24 is distributed on 8 GPUs with 3 tiles per GPU using `pytorchs` `DistributedDataParallel`

The step length is initialised to 1.0×10^{-4} and incremented by 1.0×10^{-4} every 10th step until step 1 000 when the step length has reached 1.0×10^{-2}. After this warm up period, the step length follows a cosine annealing schedule until termination (see protocol figure 35).[40]

The optimisation is carried out for 500 000 steps (or 4.14 epochs) before termination. Since we have 2 902 032 tiles in the dataset and 24 tiles per batch, we have 120 918 steps per epoch. The model at step 500 000 is selected as the model used in the segmentation method.

We employ *Automatic mixed precision* both during optimisation of the network and when applying it. This is provided by the `torch.cuda.amp` module in the `pytorch` python package.

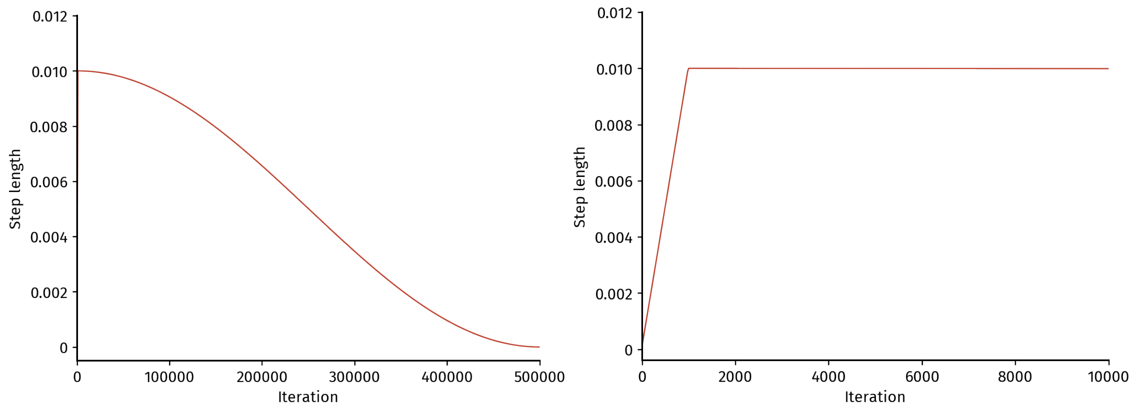

Protocol Figure 35: Step length with respect to optimisation iteration. Left panel shows all 500 000 steps while the right panel only shows the first 10 000 steps.

2.3 Method application

Application of the method on a single input scan can be summarised as

1. Read the input scan at 1 MPP resolution (section 2.3.1)
2. Partition the downsampled scan into overlapping tiles (section 2.3.1)
3. Apply the optimised segmentation network on each tile (section 2.3.2)
4. Construct a probability image from the segmentation network tiles (section 2.3.3)
5. Post-process to yield a final segmentation mask (section 2.3.4)

2.3.1 Downsampling and tiling

Scan reading and downsampling is done as described in section 2.2.1. Tiling is done as described in section 2.2.2, with tile size of $7\,680 \times 7\,680$ pixels with a minimum overlap of 1 024 pixels in each direction (see example in protocol figure 36).

2.3.2 Neural network

Input images are processed with the optimised segmentation network after the following operations are applied on the input image

1. Read image as RGB with 8-bit values in each channel
2. Zero-pad image so that both the image height and width are divisible by 16. This step is not necessary for this particular setup since we have tiles with size $7\,680 \times 7\,680$, but is included for making the method applicable in the general case with varying input sizes.
3. Scale image values to $(0, 1)$ by dividing by 255
4. Subtract image by development dataset mean (protocol table 12)
5. Divide image by development dataset standard deviation (protocol table 12)

The resulting prediction from the segmentation network is an image with one channel per output class, where only the channel corresponding to the tumour class is used further. Its values are floats where pixel value 0 indicates negative prediction and 1 indicate positive prediction. The image values are multiplied by 255 before the image is quantised to 8 bits. The padding (if any) is removed before the prediction is written as a `png` image.

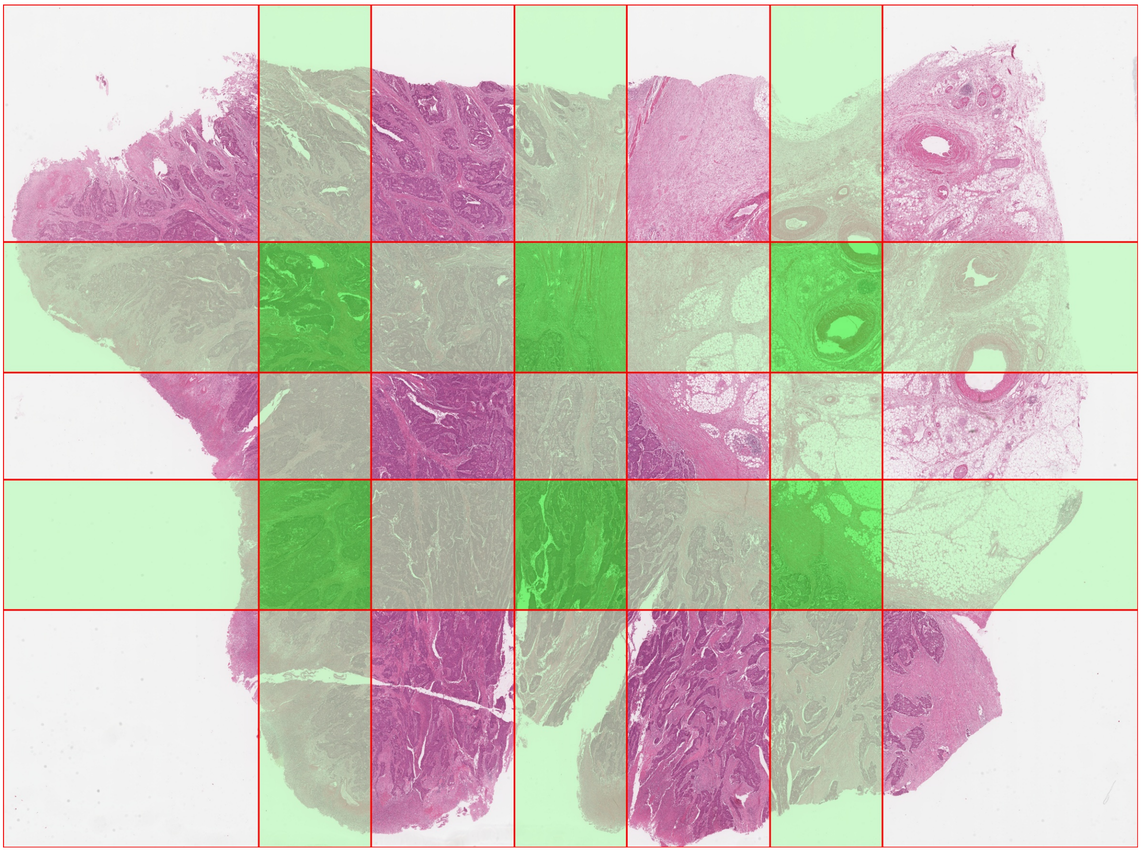

Protocol Figure 36: Tiling with size 7 680 × 7 680 pixels and minimum overlap of 1 024 pixels at resolution 1 MPP. Red shows the tile contour. Tile interiors are shown in green with opacity increasing with the number of overlapping tiles: transparent for no overlapping, light green for two overlapping, and darker green for four overlapping tiles.

2.3.3 Reconstruction from tiles

The final reconstructed image f is computed as $f = \sum_i w_i g_i$ where f, w_i, g_i are $m \times n$ matrices and i iterate over all tiles. g_i represent a single tile output from the segmentation network, and has the output tile value in the tile location and value zero everywhere else. w_i represent a single weight tile which has values in the corresponding tile location and value zero everywhere else. w_i have values in $[0, 1]$ and g_i have integer values in $[0, 255]$ since they have been written as 8-bit `png` files by the segmentation network. The values of f are quantised to integer values by rounding with the tie-breaking rule of rounding half to even before f is written as `png`.

The weight tiles are constructed so that the sum weight image $s = \sum_i w_i$ with shape $m \times n$ will have value 1 in all pixels. In the rest of this explanation a *weight tile* and *image tile* will refer only to the part of w_i and g_i that correspond to the location of each tile, respectively.

The tile weights are constructed in three phases, and an example result is shown in protocol figure 37. First, initial weight tiles are computed for each image tile. These weight tiles are weighted by distance in overlapping regions. A sum image the same size of f is constructed by adding all initial weight tiles w at their locations within this sum image. Each initial weight tile is normalised by dividing it by the tile cropped out from its location within the sum image. The next two paragraphs explain the construction of the initial weight tiles.

An initial weight tile w is computed as the element-wise product of four side-specific weight tiles: w_t weighting overlaps at the top of w, w_b weighting overlaps at the bottom of w, w_l weighting

overlaps at the left of w and w_r weighting overlaps at the right of w.

In order to compute a side-specific weight tile, e.g. w_r, the smallest leftmost coordinate of all overlapping tiles with a leftmost coordinate greater than the leftmost coordinate in w is recorded. The region between this recorded coordinate and the rightmost coordinate of w defines the overlapping area to the right in w. All pixels in w_r to the left of this overlapping area are given value 1, and all other pixels are giving a value decreasing linearly with the distance from the left overlapping border: $v = 1 - \frac{d}{1+l}$ where v is the result value, d is the distance from the left overlapping border, and l is the length of the overlapping region. Both d and l are measured in pixels. The procedure and weighting is similar for the other side-specific weight tiles.

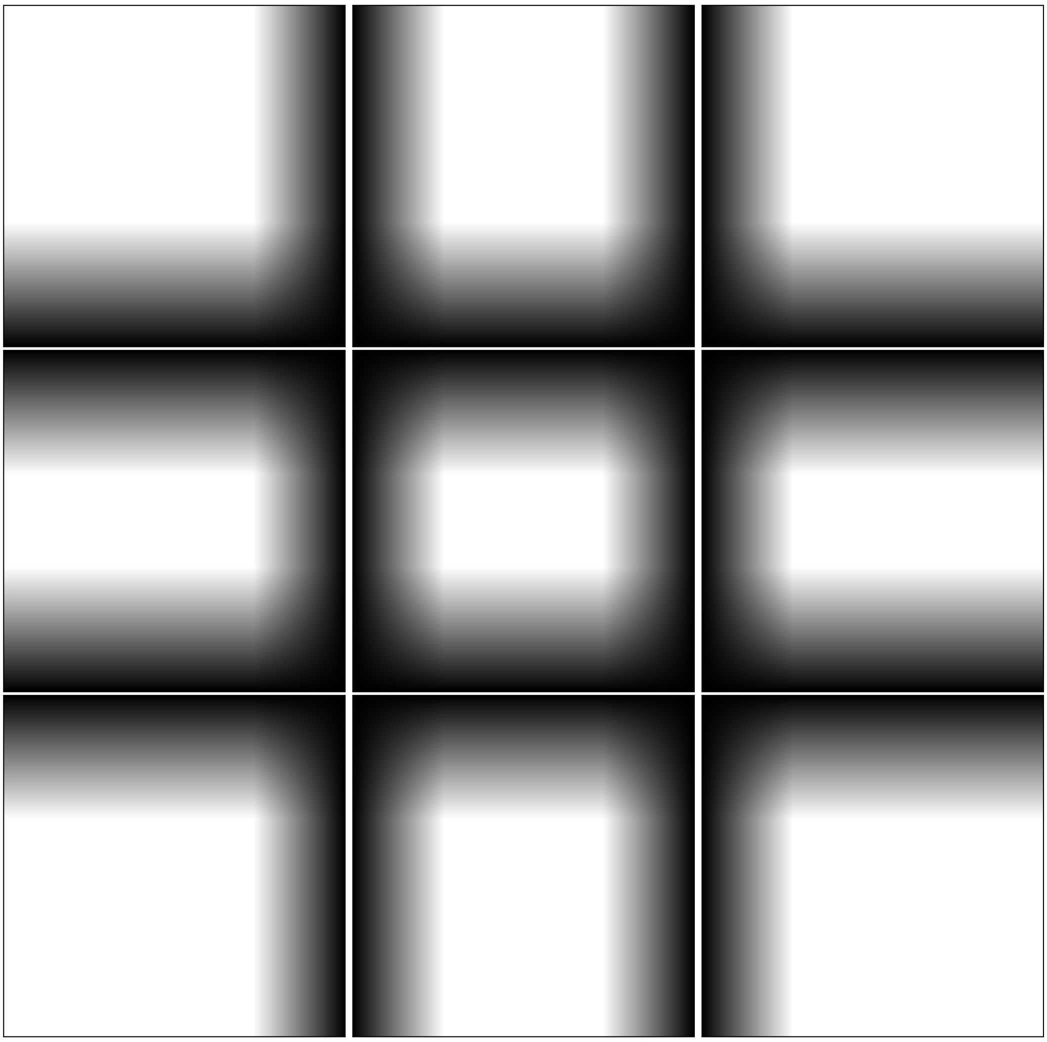

Protocol Figure 37: Weight tiles for merging overlapping tiles. The top left tile is overlapping with the tile to its right, the tile below, and to the tile in the middle. The centre tile is overlapping with all other tiles. The weight tile sub-images are arranged as their corresponding tiles. Note that frames are added in the above figure for clarity, but they are not present in the weight tiles.

2.3.4 Result post-processing

Post-processing is used to transform the segmentation network output probability maps to binary foreground and background masks. The process comprise three steps

1. Smooth the probability map
2. Binarise the smoothed probability map
3. Clean the binarised mask

The merged probability map from section 2.3.3 has the same size as the 1 MPP scan image they originate from. Before further post-processing, this probability map is downsampled by five times in both horizontal and vertical directions (corresponding to the scan image at 5 MPP).

We apply smoothing of the probability map both to get a smooth segmentation boundary in the final segmentation, and to reduce the impact of noise in the post-processing. For the sake of efficiency, the probability map is further downsampled before smoothing and upsampled again after smoothing is done. The downsampling factor is set to 0.2 for both the vertical and the horisontal direction unless the resulting image has an area less than 10^6, in which case then the image is resized to have an area of 10^6. This threshold is arbitrarily chosen as a safeguard against very small scans. Specifically, the new height and width is found by multiplication with a factor $\max\{0.2, \sqrt{10^6/(hw)}\}$ where hw is the area of the input. Then the resulting float value is floored to get an integer value. The image then undergoes median blurring with an aperture size of 9 using `OpenCVs` `medianBlur` function. Next, the result is further smoothed using `OpenCVs` `GaussianBlur` function with a kernel size of 5×5. Finally, the smoothed probability map is upsampled back to the original size corresponding to the 5 MPP scan image.

The smooth probability map is then dichotomised into foreground and background using a hysteresis threshold method. The lower threshold value is set to 85 (1/3 of 255) and the higher threshold value is set to 229 ($\approx 90\%$ of 255).

Finally, foreground regions in the mask are pruned with the following procedure. For each connected foreground region in the foreground mask, collect the values the region cover in the smooth probability map. If the 95th percentile value of this collection is greater than 229, the corresponding region is kept as foreground, else it is labelled background.

All pixels not foreground in both the foreground mask from the probability map and the foreground mask from the scan image (section 2.2.4) are labelled as background. The resulting mask is further processed by removing small background regions and then small foreground regions as explained in section 2.2.4 for the foreground mask.

2.4 Performance evaluation

To measure the similarity between the reference and predicted segmentation, we use different metrics to highlight different similarities.

Since we employ the same background exclusion on both reference and prediction masks, it is of little interest to count true negative pixels in the white background area of a scan. We therefore excluded background in the performance evaluation. True negatives are therefore pixels that are marked as background in the prediction and neither as tumour nor background in the reference mask.

2.4.1 Overlap counting

For simple overlap comparison, we partition the pixels based on how they overlap in the reference and predicted segmentation:

Protocol Table 13: Confusion matrix

		Prediction		
		Background	Foreground	
Reference	Background	TN	FP	RN
	Foreground	FN	TP	RP
		PN	PP	N

$$N = \text{Pixel count in the image after excluding white background}$$

$$RP = |\{x : x \text{ is foreground in reference}\}|$$

$$RN = |\{x : x \text{ is background in reference}\}|$$

$$PP = |\{x : x \text{ is foreground in prediction}\}|$$

$$PN = |\{x : x \text{ is background in prediction}\}|$$

$$TP = |\{x : x \text{ is foreground in reference and prediction}\}|$$

$$FN = |\{x : x \text{ is foreground in reference and background in prediction}\}|$$

$$FP = |\{x : x \text{ is background in reference and foreground in prediction}\}|$$

$$TN = |\{x : x \text{ is background in reference and prediction}\}|$$

These counts comprise a contingency table termed a *confusion matrix* (protocol table 13).

We can derive different metrics from the confusion matrix to measure different features of the segmentation result. Some common metrics that are used in this work are presented in the following.

True positive rate or sensitivity or recall measures the fraction of reference foreground pixels that are correctly marked as foreground

$$TPR = \frac{TP}{TP + FN} \tag{1}$$

False negative rate measures the fraction of reference foreground pixels that are wrongly marked as background

$$FNR = \frac{FN}{TP + FN} \tag{2}$$

True negative rate or specificity measures the fraction of reference background pixels that are correctly marked as background

$$TNR = \frac{TN}{TN + FP} \tag{3}$$

False positive rate measures the fraction of reference background pixels that are wrongly marked as foreground

$$FPR = \frac{FP}{TN + FP} \tag{4}$$

Positive predictive value or precision measures the fraction of predicted foreground pixels that are correctly marked as foreground

$$PPV = \frac{TP}{TP + FP} \tag{5}$$

Negative predictive value measures the fraction of predicted background pixels that are correctly marked as background

$$NPV = \frac{TN}{TN + FN} \tag{6}$$

Informedness

$$BIN = TPR + TNR - 1 \tag{7}$$

Markedness

$$BMA = PPV + NPV - 1 \tag{8}$$

Matthew's correlation coefficient is the geometric mean of informedness and markedness

$$MCC = \frac{TP \times TN - FN \times FP}{\sqrt{(TP + FN)(TP + FP)(TN + FN)(TN + FP)}} \tag{9}$$

Sørensen-Dice similarity coefficient or F_1 score is the harmonic mean of the true positive rate and the positive predictive value

$$DSC = \frac{2TP}{2TP + FN + FP} \tag{10}$$

3 Analyses

3.1 Primary analysis

The primary analysis of this study is the performance assessment of an automatic method tasked to segment tumour regions from non-tumour regions in WSIs of H&E-stained tissue sections known to contain cancerous regions. The performance is evaluated against manual segmentations in the validation cohorts described in section 1.2 using only scans from the Aperio AT2 scanner.

The single segmentation method is as described in section 2 and developed using images from cohorts described in section 1.1.

The primary analysis of each validation cohort described in section 1.2 is the segmentation method's performance measured using the Dice similarity coefficient (eq. (10)) averaged over the images in the cohort with associated 95% confidence interval. The results will also be presented as a box plot showing mean value (which is the primary metric), median value, interquartile range, whiskers and outliers for each cohort.

3.2 Secondary analyses

3.2.1 Different performance evaluation metrics of primary result

In the corresponding manner as done in the primary analysis for the Dice similarity coefficient, report the following segmentation performance evaluation metrics:

- Prevalence (amount of reference positive / total)
- Bias (amount of predicted positive / total)
- True positive rate (eq. (1))
- False negative rate (eq. (2))
- True negative rate (eq. (3))
- False positive rate (eq. (4))
- Positive predictive value (eq. (5))
- Negative predictive value (eq. (6))
- Informedness (eq. (7))
- Markedness (eq. (8))
- Matthews correlation coefficient (eq. (9))

3.2.2 Primary result on scans from the NanoZoomer XR

Repeat the primary analysis and the analysis in section 3.2.1 but on scans from NanoZoomer XR instead of Aperio AT2.

3.2.3 Single cancer type training

Repeat the primary analysis and the analysis in section 3.2.1 on methods that are developed exactly as the method analysed in the primary analysis except that the methods only have been trained on a subset of the original training set. The original training set is partitioned into one subset per cancer type, comprising scans only from that cancer type. Therefore, four segmentation methods are analysed, one for each of the cancer types

- Colorectal carcinoma
- Endometrial carcinoma

- Lung carcinoma
- Prostate carcinoma

All four additional segmentation methods will be analysed on all validation cohorts.

3.2.4 Primary analysis replication

Repeat the primary analysis and the analysis in section 3.2.1 on methods trained exactly as the one in the primary analysis, except for different random seed which will affect the neural network parameter initialisation and the image input pipeline. Both two additional methods will be analysed on all validation cohorts.

References

[1] David G Lowe. Object recognition from local scale-invariant features. In *Proceedings of the seventh IEEE international conference on computer vision*, volume 2, pages 1150–1157. Ieee, 1999.

[2] Adam Goode, Benjamin Gilbert, Jan Harkes, Drazen Jukic, and Mahadev Satyanarayanan. Openslide: A vendor-neutral software foundation for digital pathology. *Journal of pathology informatics*, 4, 2013.

[3] J Bondi, A Husdal, G Bukholm, JM Nesland, A Bakka, and IRK Bukholm. Expression and gene amplification of primary (a, b1, d1, d3, and e) and secondary (c and h) cyclins in colon adenocarcinomas and correlation with patient outcome. *Journal of clinical pathology*, 58(5):509–514, 2005.

[4] Ole-Johan Skrede, Sepp De Raedt, Andreas Kleppe, Tarjei S Hveem, Knut Liestøl, John Maddison, Hanne A Askautrud, Manohar Pradhan, John Arne Nesheim, Fritz Albregtsen, et al. Deep learning for prediction of colorectal cancer outcome: a discovery and validation study. *The Lancet*, 395(10221):350–360, 2020.

[5] MA Merok, T Ahlquist, EC Røyrvik, KF Tufteland, M Hektoen, OH Sjo, T Mala, A Svindland, RA Lothe, and A Nesbakken. Microsatellite instability has a positive prognostic impact on stage ii colorectal cancer after complete resection: results from a large, consecutive norwegian series. *Annals of Oncology*, 24(5):1274–1282, 2013.

[6] TS Hveem, MA Merok, ME Pretorius, M Novelli, MS Bævre, OH Sjo, N Clinch, K Liestøl, A Svindland, RA Lothe, et al. Prognostic impact of genomic instability in colorectal cancer. *British journal of cancer*, 110(8):2159–2164, 2014.

[7] David J Kerr, Janet A Dunn, Michael J Langman, Justine L Smith, Rachel SJ Midgley, Andrew Stanley, Joanne C Stokes, Patrick Julier, Claire Iveson, Ravi Duvvuri, et al. Rofecoxib and cardiovascular adverse events in adjuvant treatment of colorectal cancer. *New England Journal of Medicine*, 357(4):360–369, 2007.

[8] Jone Trovik, Elisabeth Wik, Henrica MJ Werner, Camilla Krakstad, Harald Helland, Ingrid Vandenput, Tormund S Njolstad, Ingunn M Stefansson, Janusz Marcickiewicz, Solveig Tingulstad, et al. Hormone receptor loss in endometrial carcinoma curettage predicts lymph node metastasis and poor outcome in prospective multicentre trial. *European journal of cancer*, 49(16):3431–3441, 2013.

[9] Tarjei S Hveem, Tormund S Njølstad, Birgitte Nielsen, Rolf Anders Syvertsen, John Arne Nesheim, Marna L Kjæreng, Wanja Kildal, Manohar Pradhan, Janusz Marcickiewicz, Solveig Tingulstad, et al. Changes in chromatin structure in curettage specimens identifies high-risk patients in endometrial cancer. *Cancer Epidemiology and Prevention Biomarkers*, 26(1):61–67, 2017.

[10] Robert J. Kurman, Maria Luisa Carcangiu, C. Simon Herrington, and Robert H. Young. *WHO Classification of Tumours of Female Reproductive Organs*. International Agency for Research on Cancer, Lyon, 4 edition, 2014.

[11] WHO Classification of Tumours Editorial Board. *WHO Classification of Tumours; Female Genital Tumours*. International Agency for Research on Cancer, Lyon, 5 edition, 2020.

[12] Jonathan I Epstein, Lars Egevad, Mahul B Amin, Brett Delahunt, John R Srigley, and Peter A Humphrey. The 2014 international society of urological pathology (isup) consensus conference on gleason grading of prostatic carcinoma. *The American journal of surgical pathology*, 40(2):244–252, 2016.

[13] Håkon Wæhre, Ljiljana Vlatkovic, Milada Cvancarova, Elisabeth Paus, Sophie D Fosså, and Håvard E Danielsen. Fifteen-year mortality after radical prostatectomy: Which factors are available for patient counselling? *Scandinavian Journal of Urology*, 48(2):123–130, 2014.

[14] Karolina Cyll, Elin Ersvær, Ljiljana Vlatkovic, Manohar Pradhan, Wanja Kildal, Marte Avranden Kjær, Andreas Kleppe, Tarjei S Hveem, Birgitte Carlsen, Silje Gill, et al. Tumour heterogeneity poses a significant challenge to cancer biomarker research. *British journal of cancer*, 117(3):367–375, 2017.

[15] Rachel S Kerr, Sharon Love, Eva Segelov, Elaine Johnstone, Beverly Falcon, Peter Hewett, Andrew Weaver, David Church, Claire Scudder, Sarah Pearson, et al. Adjuvant capecitabine plus bevacizumab versus capecitabine alone in patients with colorectal cancer (quasar 2): an open-label, randomised phase 3 trial. *The Lancet Oncology*, 17(11):1543–1557, 2016.

[16] Sigurd M Hald, Mehrdad Rakaee, Inigo Martinez, Elin Richardsen, Samer Al-Saad, Erna-Elise Paulsen, Egil Støre Blix, Thomas Kilvaer, Sigve Andersen, Lill-Tove Busund, et al. Lag-3 in non–small-cell lung cancer: expression in primary tumors and metastatic lymph nodes is associated with improved survival. *Clinical lung cancer*, 19(3):249–259, 2018.

[17] Mehrdad Rakaee, Lill-Tove Rasmussen Busund, Simin Jamaly, Erna-Elise Paulsen, Elin Richardsen, Sigve Andersen, Samer Al-Saad, Roy M Bremnes, Tom Donnem, and Thomas K Kilvaer. Prognostic value of macrophage phenotypes in resectable non–small cell lung cancer assessed by multiplex immunohistochemistry. *Neoplasia*, 21(3):282–293, 2019.

[18] Karolina Cyll, Andreas Kleppe, Joakim Kalsnes, Ljiljana Vlatkovic, Manohar Pradhan, Wanja Kildal, Kari Anne R Tobin, Trine M Reine, Håkon Wæhre, Bjørn Brennhovd, et al. Pten and dna ploidy status by machine learning in prostate cancer. *Cancers*, 13(17):4291, 2021.

[19] Ivar Skaland, Emiel AM Janssen, Einar Gudlaugsson, Lydia Hui Ru Guo, and Jan Baak. The prognostic value of the proliferation marker phosphohistone h3 (pph3) in luminal, basal-like and triple negative phenotype invasive lymph node-negative breast cancer. *Analytical Cellular Pathology*, 31(4):261–271, 2009.

[20] Ivar Skaland, Emiel AM Janssen, Einar Gudlaugsson, Jan Klos, Kjell H Kjellevold, Håvard Søiland, and Jan Baak. Validating the prognostic value of proliferation measured by phosphohistone h3 (pph3) in invasive lymph node-negative breast cancer patients less than 71 years of age. *Breast cancer research and treatment*, 114(1):39–45, 2009.

[21] Kristin Jonsdottir, Hui Zhang, Darshni Jhagroe, Ivar Skaland, Aida Slewa, Benny Björkblom, Eleanor T Coffey, Einar Gudlaugsson, Rune Smaaland, Emiel AM Janssen, et al. The prognostic value of marcks-like 1 in lymph node-negative breast cancer. *Breast cancer research and treatment*, 135(2):381–390, 2012.

[22] Kristin Jonsdottir, Susanne R Janssen, Fabiana C Da Rosa, Einar Gudlaugsson, Ivar Skaland, Jan PA Baak, and Emiel AM Janssen. Validation of expression patterns for nine mirnas in 204 lymph-node negative breast cancers. *PloS one*, 7(11):e48692, 2012.

[23] Nina Gran Egeland, Marie Austdal, Bianca van Diermen-Hidle, Emma Rewcastle, Einar G Gudlaugsson, Jan PA Baak, Ivar Skaland, Emiel AM Janssen, and Kristin Jonsdottir. Validation study of marcksl1 as a prognostic factor in lymph node-negative breast cancer patients. *PloS one*, 14(3):e0212527, 2019.

[24] Vebjørn Kvikstad, Ok Målfrid Mangrud, Einar Gudlaugsson, Ingvild Dalen, Hans Espeland, Jan Baak, and Emiel AM Janssen. Prognostic value and reproducibility of different microscopic characteristics in the who grading systems for pta and pt1 urinary bladder urothelial carcinomas. *Diagnostic pathology*, 14(1):1–8, 2019.

[25] Melinda Lillesand, Vebjørn Kvikstad, Ok Målfrid Mangrud, Einar Gudlaugsson, Bianca van Diermen-Hidle, Ivar Skaland, Jan PA Baak, and Emiel AM Janssen. Mitotic activity index and cd25+ lymphocytes predict risk of stage progression in non-muscle invasive bladder cancer. *Plos one*, 15(6):e0233676, 2020.

[26] John Canny. A computational approach to edge detection. *IEEE Transactions on pattern analysis and machine intelligence*, pages 679–698, 1986.

[27] Alexander Buslaev, Vladimir I. Iglovikov, Eugene Khvedchenya, Alex Parinov, Mikhail Druzhinin, and Alexandr A. Kalinin. Albumentations: Fast and flexible image augmentations. *Information*, 11(2), 2020.

[28] Adam Paszke, Sam Gross, Francisco Massa, Adam Lerer, James Bradbury, Gregory Chanan, Trevor Killeen, Zeming Lin, Natalia Gimelshein, Luca Antiga, Alban Desmaison, Andreas Kopf, Edward Yang, Zachary DeVito, Martin Raison, Alykhan Tejani, Sasank Chilamkurthy, Benoit Steiner, Lu Fang, Junjie Bai, and Soumith Chintala. Pytorch: An imperative style, high-performance deep learning library. In H. Wallach, H. Larochelle, A. Beygelzimer, F. d'Alché-Buc, E. Fox, and R. Garnett, editors, *Advances in Neural Information Processing Systems 32*, pages 8024–8035. Curran Associates, Inc., 2019.

[29] Andrew Brock, Soham De, Samuel L. Smith, and Karen Simonyan. High-performance large-scale image recognition without normalization. *arXiv preprint arXiv:2102.06171*, 2021.

[30] Andrew Brock, Soham De, and Samuel L. Smith. Characterizing signal propagation to close the performance gap in unnormalized resnets. In *9th International Conference on Learning Representations, ICLR*, 2021.

[31] Ross Wightman. Pytorch image models. https://github.com/rwightman/pytorch-image-models, 2019.

[32] Jie Hu, Li Shen, and Gang Sun. Squeeze-and-excitation networks. In *Proceedings of the IEEE conference on computer vision and pattern recognition*, pages 7132–7141, 2018.

[33] Qilong Wang, Banggu Wu, Pengfei Zhu, Peihua Li, Wangmeng Zuo, and Qinghua Hu. Eca-net: Efficient channel attention for deep convolutional neural networks. In *2020 IEEE/CVF Conference on Computer Vision and Pattern Recognition (CVPR)*, pages 11531–11539, 2020.

[34] Dan Hendrycks and Kevin Gimpel. Gaussian error linear units (gelus). *arXiv preprint arXiv:1606.08415*, 2016.

[35] Liang-Chieh Chen, Yukun Zhu, George Papandreou, Florian Schroff, and Hartwig Adam. Encoder-decoder with atrous separable convolution for semantic image segmentation. In *Proceedings of the European conference on computer vision (ECCV)*, pages 801–818, 2018.

[36] Pavel Yakubovskiy. Segmentation models pytorch. https://github.com/qubvel/segmentation_models.pytorch, 2020.

[37] Yuxin Wu and Kaiming He. Group normalization. In *Proceedings of the European conference on computer vision (ECCV)*, pages 3–19, 2018.

[38] Kaiming He, Xiangyu Zhang, Shaoqing Ren, and Jian Sun. Delving deep into rectifiers: Surpassing human-level performance on imagenet classification. In *Proceedings of the IEEE international conference on computer vision*, pages 1026–1034, 2015.

[39] Ilya Sutskever, James Martens, George Dahl, and Geoffrey Hinton. On the importance of initialization and momentum in deep learning. In *International conference on machine learning*, pages 1139–1147. PMLR, 2013.

[40] Ilya Loshchilov and Frank Hutter. Sgdr: Stochastic gradient descent with warm restarts. *arXiv preprint arXiv:1608.03983*, 2016.
